# An intersectionality-based policy analysis of COVID-19 measures and women informal food vendors in Kisumu, Kenya

**DOI:** 10.1371/journal.pone.0359620

**Published:** 2026-10-01

**Authors:** Elizabeth Opiyo Onyango, Joyce Kiplagat, Rebecca Gokiert, Patrick Mbullo Owuor

**Affiliations:** 1 School of Public Health University of Alberta, Edmonton, Alberta, Canada; 2 Department of Anthropology, Wayne State University, Detroit, United States of America; 3 Pamoja Community-Based Organization, Kisumu, Kenya; Middle Tennessee State University Jennings A Jones College of Business, UNITED STATES OF AMERICA

## Abstract

**Introduction:**

Women working as informal food vendors in Kisumu faced precarious work conditions that worsened during the COVID-19 pandemic. Despite playing a vital role in urban household food security, they were excluded from policy decisions, which were largely confined to technical experts and national government officials.

**Methods:**

Using the Intersectionality-Based Policy Analysis (IBPA) framework, this study examined the engagement of women informal food vendors and the disparities in COVID-19 control policies. Between July 10, 2024, and October 15, 2024, we conducted a qualitative study using key informant interviews (n = 20), in-depth interviews (n = 20), four focus group discussions (n = 40; 10 participants each), and a purposive review of four policy documents. Participants were recruited via purposive sampling in collaboration with Pamoja Community Based Organisation, and data were analyzed thematically guided by the IBPA principles.

**Results:**

The findings indicate that excluding women vendors from decision-making led to gender-insensitive policies that worsened existing inequalities by overlooking the vendors’ diverse social identities. Furthermore, inconsistent policy implementation increased gender-based vulnerabilities, leaving these women exposed to harassment, extortion, and physical assault.

**Discussion:**

To prepare for future emergencies, governments must establish inclusive, gender-responsive policy frameworks that prioritize the perspectives of informal food vendors, thereby safeguarding sustainable urban food systems. Specifically, municipal, county, and national authorities should create consultative mechanisms to ensure women informal food vendors actively participate in decision-making processes. Such integration will foster gender-sensitive policy development and keep vendors’ lived experiences central to urban food governance. Ultimately, this research demonstrates the value of applying an intersectional policy analysis framework to health research, advancing equity and social justice by strengthening the participation of marginalized groups in emergency response and regulatory planning.

## Introduction

In early 2020, the World Health Organization (WHO) declared COVID-19 a global pandemic, prompting urgent and comprehensive public health interventions [1]. Governments worldwide responded with mandatory containment measures, such as lockdowns and curfews [2], alongside safety protocols—including mask-wearing, hand hygiene, and physical distancing—to mitigate viral transmission [3]. These control measures, combined with the pandemic’s broader impact on global trade, severely disrupted both formal and informal economies [4–6]. Within the informal sector, these policies produced disparate social and economic outcomes for men and women [7], thereby exposing and exacerbating existing socioeconomic and cultural inequalities, particularly in urban centers [8–13].

In cities of the Global South, women disproportionately shouldered the burdens of the pandemic and its associated control measures. Women food vendors, in particular, faced systemic challenges—including reduced market hours, restricted movement, disrupted food supply chains, and increased household and caregiving responsibilities—which resulted in significant income loss and heightened food insecurity [11,14–16]. The impact was especially severe for women-led informal food businesses that rely on direct social interaction and close physical proximity with clientele—operational necessities that were severely curtailed by pandemic protocols [9]. Ultimately, these measures exacerbated existing inequalities because policy frameworks failed to account for the specific socio-cultural and economic realities of these women [10,13].

Moreover, the lack of gender-sensitive policy measures created unique challenges for women throughout the pandemic [17]. Women informal food vendors encountered significant obstacles in accessing financial assistance from both public and private institutions [18,19]. In the African context, these challenges were further compounded by deep-seated socio-cultural norms and patriarchal structures that often limit women’s financial autonomy and their ability to provide the assets required for loan collateral [15,20].

Across most cities in the Global South, particularly secondary cities, women are overrepresented in the informal sector, with a heavy concentration in food vending [18]. These secondary cities serve as critical regional, administrative, and economic hubs characterized by rapid urbanization and strong urban-rural linkages [21]. In Kisumu, Kenya—a major secondary city—women constitute approximately 60% of all informal traders, predominantly operating food businesses [22,23]. These women-led enterprises are crucial for supporting urban household food security and livelihoods [10,15]. However, pandemic response policies overlooked the distinct vulnerabilities of women in informal food sectors. This omission fueled an increase in gender-based and intimate partner violence while undermining women’s ability to sustain their businesses and contribute to the local economy [14,24–26]. Compounding these challenges, heightened unpaid care and domestic responsibilities during the pandemic further restricted women from fully engaging in their economic activities [27].

However, research on gender-specific gaps in COVID-19 control policies remains limited, particularly regarding the impact on women-led informal food businesses in secondary cities of the Global South [28]. Most existing literature focuses on the broader informal sector in major metropolitan hubs such as Nairobi, Kampala, Karachi, West Bengal, and Johannesburg [14,29–33]. Previous analyses in Durban, Nairobi, and Kampala indicate that women in the urban informal economy were systematically excluded from COVID-19 policy processes [14,33,34]. Additionally, studies in Karachi demonstrate that lockdown mandates intensified gendered inequalities—increasing domestic violence and caregiving burdens—even as women remained essential to household food security [29]. Similarly, research in West Bengal suggests that women informal workers had little influence on pandemic policy decisions [30]. Overall, as emphasized by Martínez and Young [35], there is a critical need for more research focused specifically on how the pandemic affected women in the informal food sector within secondary cities.

Secondary cities act as vital conduits for social, economic, and cultural exchange between rural and urban areas, yet they often face marginalization, particularly during emergencies [15]. For example, COVID-19 policies in Kisumu City were centralized in Nairobi, excluding the specific realities of vulnerable populations in secondary cities, especially women.

Kisumu, the economic hub for western Kenya, relies heavily on informal trade [23]. Women own over 60% of these businesses, with most residing in informal settlements and belonging to the Luo or Luhya communities [15]. In these communities, deeply rooted patriarchal systems often diminish the social standing of women, who are traditionally assigned domestic and caregiving duties. Pandemic containment measures exacerbated these challenges for women-led businesses, as the closure of schools and increased medical needs intensified caregiving burdens [10,15]. Furthermore, male-dominated leadership and systemic power imbalances marginalized female perspectives from the informal food sector during pandemic response planning

Against this backdrop, this study examines how COVID-19 containment policies impacted women-led informal food businesses in Kisumu. Specifically, it explores how centralized governance and entrenched gender norms hindered operational resilience and excluded female perspectives from policy formulation. By analyzing the gendered disparities faced by female vendors, this research highlights how women entrepreneurs were overlooked in pandemic control strategies within secondary cities of the Global South. Ultimately, elucidating these gendered dimensions is essential for advocating for equitable policy frameworks that safeguard the food security, nutrition, and economic stability of urban households during emergencies.

### The Intersectionality-Based Policy Analysis (IBPA) framework

This study adopted the Intersectionality-Based Policy Analysis (IBPA) framework [36] to investigate how power dynamics and systemic structures contributed to the marginalization of women food vendors in secondary cities during the COVID-19 pandemic. The IBPA framework extends beyond viewing gender as an isolated factor, examining how intersecting identities and regulatory control measures shape social locations and systemic inequities.

We applied seven of the eight IBPA principles: equity, power, intersecting categories, multi-level analysis, reflexivity, social justice, and diverse knowledges [36]. These principles (Fig 1) guided the analysis of policy documents, key informant interviews, focus group discussions, and in-depth interviews, as well as the interpretation of the findings.

**Fig 1:**
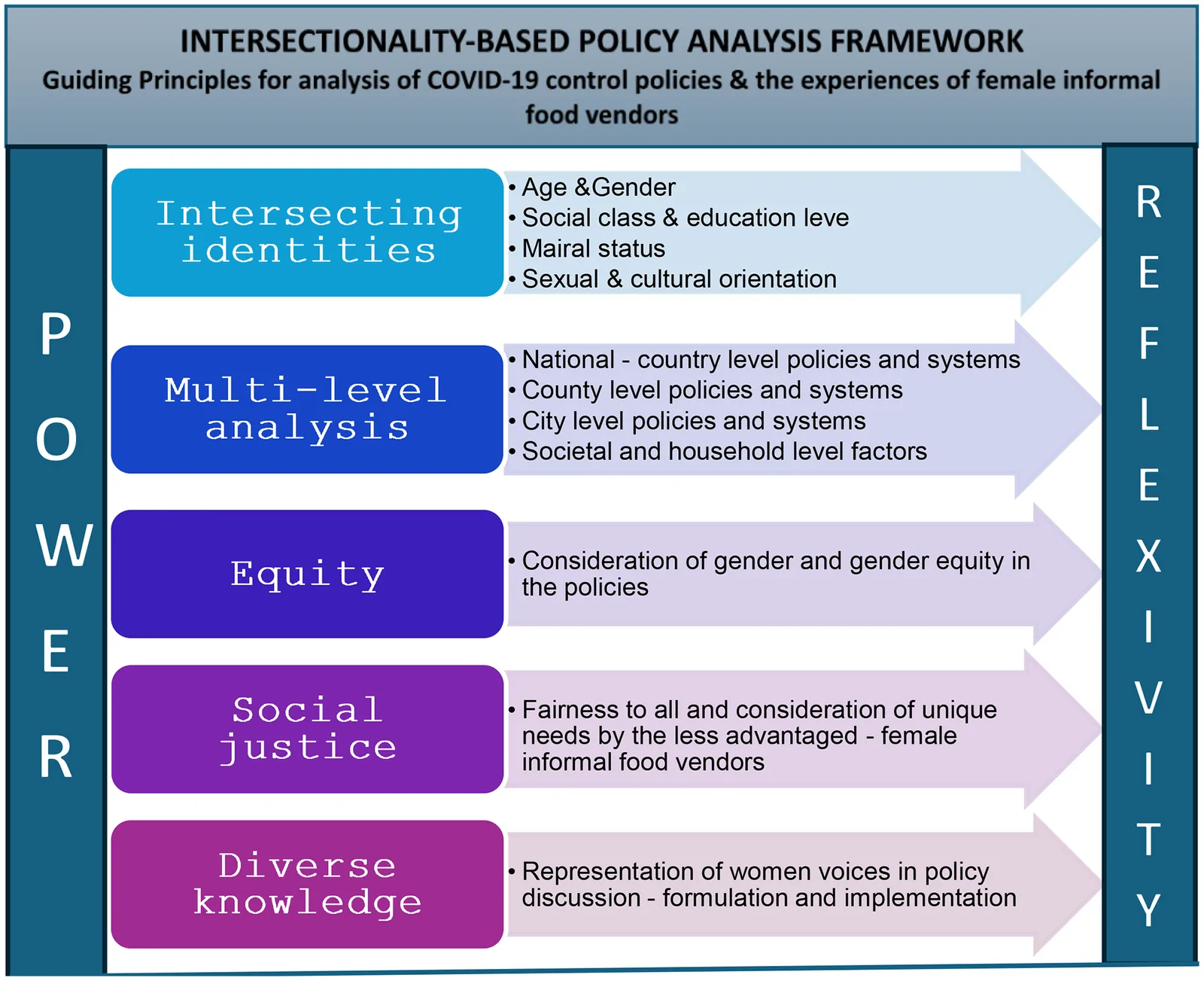
The conceptual framework for the IBPA guiding principles for analysis of COVID-19 control policies & the experiences of female informal food vendors.

The principle of intersecting categories was used to examine how overlapping social identities—including gender, age, sexual orientation, marital status, education, culture, and socioeconomic status—interacted with pandemic response strategies to deepen vendor marginalization. Furthermore, the analysis assessed whether policy design acknowledged the heterogeneous identities and unique needs of informal food vendors. Applying this intersectional lens illuminated how social positions influenced women’s experiences under pandemic policies and exposed systemic inequities in policy design, dissemination, and interpretation [37].

By integrating the principles of power and multi-level analysis, we highlighted how overlapping power dynamics either enabled inclusion or reinforced systemic inequities for female vendors. In Kenya—where healthcare delivery is devolved to county and municipal authorities while legislative mandates remain centralized—complex hierarchies of power emerge. Managing a public health crisis within this devolved context required a multisectoral approach that prioritized meaningful engagement with marginalized populations, including informal food vendors.

In addition, our analytical framework combined a gender equity lens with the principle of social justice to evaluate gender inclusivity within COVID-19 control measures across varying levels of governance. Applying the principle of diverse knowledges enabled us to interrogate whether women vendors were included in policy development and how structural power influenced the integration of their perspectives. While prior research shows that governments broadly neglected gender-specific needs during pandemic responses [38], using the IBPA framework allowed this study to look beyond gender alone to evaluate intersecting identities, power structures, social justice, and lived experiences, while maintaining analytical self-reflexivity. The eighth principle—time and space—was excluded, as this study focused on specific, localized policy dynamics during a defined time frame.

## Methods

### Study setting

This study was conducted in Kisumu City, Kisumu County, western Kenya. The city’s proximity to Lake Victoria, the world’s second-largest freshwater lake, provides significant economic benefits through fishing, trade, and transport [23]. Following Kenya’s 2010 constitutional promulgation, the country transitioned to a devolved governance system comprising 47 counties [39]. Within this framework, the national government is responsible for health policy formulation, while county governments manage the implementation and delivery of health services [39].

Kisumu’s dominant economic activities encompass crop and livestock production, fisheries, trade, manufacturing, construction, and mining [40,41]. With a population of approximately 600,000, Kisumu has experienced significant migration from nearby rural regions due to rapid urbanization [42]. Approximately 64% of the city’s residents live in informal settlements characterized by high levels of poverty [23]. These settlements include Manyatta A, Manyatta B, Obunga, Bandani, Kaloleni, Nyalenda A, Nyalenda B, and Kibos [42]. Additionally, the informal food sector thrives in the city, with most residents relying on it for food rather than formal supermarkets [23,43].

We selected Kisumu City as the study site due to its vibrant informal food sector and the significant representation of women vendors in the urban food space. This makes it a relevant case study for understanding the gendered implications of COVID-19 policies in secondary cities of the Global South [23]. The study focused specifically on markets located within the municipality’s informal settlements, including Manyatta, Nyalenda, Obunga, Nyawita, and Bandani.

### Study design

Conducted between July 10 and October 15, 2024, this cross-sectional study investigated the nuanced experiences of informal food vendors, focusing on gendered dimensions and shortcomings in COVID-19 policy responses. The study employed a mixed-methods approach, combining a review of four primary national pandemic policies with qualitative data collection from vendors and key informants in Kisumu City. These policies, formulated by the national government and implemented by county and city authorities, included: Public Health (COVID-19 General Public Safety) [44]; Public Order (State Curfew) [45]; Public Health (COVID-19 Restriction of Movement of Persons and Related Measures) Rules [46]; and Public Health (Prevention, Control and Suppression Of COVID-19) Rules [47]. Primary data was generated through Key Informant Interviews (KIIs), In-Depth Interviews (IDIs), and Focus Group Discussions (FGDs) with relevant stakeholders and informal food vendors across various markets and informal settlements within Kisumu City.

### Selection of participants

#### Policy document sampling.

We purposefully selected four (n = 4) policy documents for critical review: the Public Health (COVID-19 General Public Safety) [44]; Public Order (State Curfew) [45]; Public Health (COVID-19 Restriction of Movement of Persons and Related Measures) Rules [46]; and the Public Health (Prevention, Control and Suppression Of COVID-19) Rules [47]. Inclusion criteria required that these policies: (1) targeted COVID-19 containment in Kisumu City at either the national or county level, (2) were implemented within Kisumu County, and (3) significantly impacted the informal food sector. Table 1 details these selected COVID-19 control policies.

**Table 1:** Selected COVID-19 policy documents for review.

Policy document	Relevance to the study
1. Public Health (COVID-19 General Public Safety) Rules, 2020 [44]	These policies mandated specific health protocols—such as physical distancing, mandatory mask-wearing, sanitation measures, COVID-19 screening, and prohibitions on public gatherings—which significantly disrupted the operations of informal food vendors.
2. Public Order (State Curfew) Order, No. 36 of 2020 [45]	Imposition of a 7:00 pm to 5:00am curfew, which affected business-operating hours.
3. Public Health (COVID-19 Restriction of Movement of Persons and Related Measures) Rules, 2020 [46]	Set restriction of movement of people into and out of an infected area, curtailing the supply of goods, traders and customers into markets.
4. Public Health (Prevention, Control and Suppression Of COVID-19) Rules, 2020 [47]	These regulations outlined the powers granted to medical and clinical officers for COVID-19 enforcement—including conducting searches, inspections, and decontaminations, as well as closing premises for non-compliance—which ultimately led to quarantines, reduced business profitability, and the forced closure of many premises operated by women vendors.

### Participant sampling

Participants were recruited in collaboration with the Pamoja Community Based Organisation (Pamoja CBO) in Kisumu. Leveraging the organization’s extensive local network, we utilized purposive and snowball sampling to identify participants.

Between July 10 and October 15, 2024, we recruited 80 participants for this cross-sectional study, drawing from five primary markets in Kisumu: Manyatta, Nyalenda, Obunga, Nyawita, and Bandani. Our sample included female informal food vendors, select male vendors, and key informants with direct professional experience in the informal food sector. In collaboration with the Pamoja Community Based Organisation (Pamoja CBO), we used purposive and snowball sampling to identify participants. Market representatives facilitated contact, inviting potential participants to information sessions where the study’s objectives were explained. After obtaining informed consent, 80 participants were enrolled across in-depth interviews (IDIs, *n* = 20), key informant interviews (KIIs, *n* = 20), and focus group discussions (FGDs, *n* = 40).

Participants were recruited from five key markets across Kisumu City (see Table 2 for distribution). Eligibility criteria required participants to have resided in Kisumu City for at least five years, operated an informal food business both during and after the pandemic, and provided informed consent for individual interviews or focus group discussions.

**Table 2:** Demographic characteristics of study participants.

Characteristics	All (n=80) Frequency (%)	KIIs (n=20) Frequency (%)	IDIs (n=20) Frequency (%)	4 FGD (n=40) Frequency (%)
**Education**				
Primary or less	42 (52.5)	2 (10)	14 (70)	26 (65)
Secondary	17 (21.2)	3 (15)	4 (20)	10 (25)
Tertiary	21 (26.3)	15 (75)	2 (10)	4 (10)
**Marital status**				
Single	10 (12.5)	0	5 (25)	5 (12.5)
Married	49 (61.2)	14 (70)	12 (60)	23 (57.5)
Divorced/separated/widowed	21 (26.3)	6 (30)	3 (15)	12 (30)
**Age categories**				
18-29 years	8 (10)	0	4 (20)	4 (10)
30-49 years	58 (72.5)	14 (70)	14 (70)	30 (75)
50+ years	14 (17.5)	6 (30)	2 (10)	6 (15)
Age range Mean age (SD)	25-65 40.3 (8.5)	34-48	25-65	26-59
Gender				
Female	67 (83.8)	11 (55)	16 (80)	40 (100)
Male	13 (16.2)	9 (45)	4 (20)	0
**Informal markets**				
Manyatta	19 (23.8)	3 (15)	6 (30)	10 (50)
Nyalenda	18 (22.5)	3 (15)	5 (25)	10 (50)
Obunga	16 (20)	2 (10)	4 (20)	10 (50)
Nyanwita	10 (12.5)	2 (10)	3 (15)	6 (30)
Bandani	8 (12.5)	2 (10)	2 (10)	4 (20)
Other Key informants	8 (10)	8 (80)	0	0

Twenty key informants (*n* = 20) were selected from the five study markets, as well as relevant government departments and community organizations (see Table 2 for distribution), with at least one to two participants per market. To be eligible, key informants had to: [1] be market leaders or representatives of informal food vendors; [2] represent city government departments, community organizations, microfinance initiatives, or community health programs; and [3] provide informed consent for a one-on-one interview. Those who did not meet these criteria were excluded.

### Data collection techniques

#### Policy document analysis.

We analyzed four key COVID-19 policy documents retrieved from the Kenya Law Reports portal: the Public Health (General Public Safety) Rules [44], Public Health Order (State Curfew Order) [45], Public Health (COVID-19 Restriction of Movement of Persons and Related Measures) Rules [46], and Public Health (Prevention, Control and Suppression Of COVID-19) Rules [47]. This review evaluated whether these mitigation strategies addressed the specific needs of female informal food vendors. Guided by the Intersectionality-Based Policy Analysis (IBPA) framework, we examined how these policies considered vendors’ intersecting social identities and sociocultural factors. This analysis aimed to assess the gender-responsiveness of the interventions and identify policy gaps impacting women-led informal food businesses.

### Primary data collection

Research assistants trained in qualitative methodology and fluent in English, Swahili, and Luo conducted participant recruitment and facilitated face-to-face interviews. The language of interaction was based on participant preference; most key informants chose English, while most IDI and FGD participants preferred Swahili or Luo. All interviews were audio-recorded with participant consent. Interviews conducted in English were transcribed verbatim, while those in Swahili or Luo were translated into English during transcription. Finally, research assistants and two authors (PMO & EOO)—all fluent in the three languages—reviewed all transcripts to ensure accuracy.

Semi-structured interview guides were developed to facilitate KIIs, IDIs, and FGDs. These instruments were designed to elicit discussions on key topics relevant to the experiences of women vendors during and after the implementation of COVID-19 pandemic policies. We explored the gendered considerations within the policies and the engagement of women vendors in the formulation and implementation of such policies. We probed for specific experiences with the implementation of COVID-19 control measures and the direct and indirect impacts on women-led businesses. Data collection continued until theoretical saturation was reached—the point at which no new ideas or themes emerged from the interviews. The research team maintained reflexivity throughout the process, and field notes maintained by research assistants were incorporated into the data analysis. Following each interview, research assistants summarized key insights and conducted daily debriefing meetings with the broader research team.

***Key informant interviews:*** We conducted *n* = 20 face-to-face key informant interviews (KIIs), each lasting 30–60 minutes, with a diverse group of stakeholders, including representatives from the County Government of Kisumu, market and women’s group leaders, and personnel from community lending and microfinance organizations, as well as community health promoters. These participants—including compliance experts, enforcement officers, revenue collectors, and municipal officials—provided in-depth insights into policy formulation and implementation, the impacts of these regulations on women informal food vendors, and the support provided by various entities during and after the pandemic.

***In-depth interviews*** (*n* = 20) were conducted with both female and male food vendors who operated businesses during and after the COVID-19 pandemic. Male participants were included to provide comparative insights into the experiences of female vendors under pandemic control measures. Recruitment was facilitated through our community partner, the Pamoja Community Based Organisation, and local market leadership. Following information sessions, interested vendors provided informed consent for face-to-face interviews, which were held at locations of their choosing. These interviews explored the impact of COVID-19 policies on participants’ business operations, household and caregiving responsibilities, interactions with authorities, food security, well-being, and adopted coping mechanisms. All interviews were audio-recorded, transcribed, and translated to facilitate analysis.

***Focus group discussions:*** We conducted four focus group discussions (FGDs) with a total of *n* = 40 women informal food vendors. Participants were recruited in collaboration with our community partner and women market leaders, who organized information sessions to explain the study and obtain informed consent. Discussions were held in community halls near the markets, providing a safe space for vendors to reflect collectively on how COVID-19 policies impacted their social and economic lives. Participants discussed their experiences with enforcement measures—including curfews, movement restrictions, and market closures—and explored the gendered effects of these policies. Furthermore, they offered recommendations for future policy reforms to mitigate the negative impacts of top-down, non-consultative decision-making.

### Data analysis

Primary qualitative data (IDIs, KIIs, and FGDs) were all audio-recorded following receipt of informed consent from all the participants. While KIIs were conducted in English and transcribed verbatim, IDIs and FGDs were primarily facilitated in Swahili or Luo, subsequently requiring translation into English and transcription for analysis. The transcription and translation were conducted by the local research assistants who facilitated the interviews and the discussion and were reviewed by the authors PMO and EOO who are fluent in English, Swahili, and Luo. Comprehensive data sets—comprising transcripts, policy documents, field notes, reflexive journals, and interview summaries—were imported into NVivo (QSR International, version 15) for systematic coding, management, and analysis.

### Policy document analysis

The analysis of the four policy documents was guided by seven core principles of the Intersectionality-Based Policy Analysis (IBPA) framework: power, equity, intersecting categories, multi-level analysis, social justice, diverse knowledges, and reflexivity [36]. We applied Braun and Clarke’s [48] reflexive thematic analysis through an iterative process of deductive and inductive coding to identify both pre-existing and emergent themes [49,50]. The predefined themes aligned with IBPA principles, encompassing power dynamics, intersecting identities, social justice, equity, multi-level analysis and reflexivity. The theme codes, transcripts, field notes, interview summaries, and the post-pandemic recovery policies were then imported and organized in NVivo QSR International, version 15, for line-by-line coding. To ensure rigor and trustworthiness, an independent peer researcher (GR) cross-validated the thematic coding for consistency and accuracy. This process of peer checking allowed for multiple interpretations and understanding of the experiences of women informal food vendors and the identification of gaps within the policy documents [51]. This triangulation of evidence—encompassing policy documents, key informant narratives, and vendor data—facilitated a multi-layered understanding of women’s experiences and policy gaps.

### Primary data analysis

Primary data from the KIIs, IDIs, and FGDs were analyzed inductively and deductively, guided by the IBPA framework and Braun and Clarke’s reflexive thematic analysis approach [36,48]. The research team developed initial themes and codes through prolonged data immersion, including transcribing and exhaustively reviewing the interview recordings. These codes were then organized into themes and sub-themes to create a refined code set. All data were imported into NVivo (QSR International, version 15) for systematic coding. Participant perspectives were illustrated using direct quotes [52]. To ensure analytical rigor, an independent peer researcher verified the thematic coding and consistency. Finally, findings were triangulated across primary data and policy documents to enhance the study’s credibility.

### Quality and rigor

To ensure the rigor of this study, we employed several methodological strategies. Data triangulation was achieved by synthesizing evidence from multiple sources, including policy document analysis and primary data from KIIs, FGDs, and IDIs. By integrating policy analysis with qualitative findings, we successfully triangulated theoretical frameworks with lived experience perspectives. Furthermore, we employed purposive sampling to select respondents with relevant expertise and experience in COVID-19 policy, governance, and informal food vending [53]. Finally, local research assistants from Pamoja CBO supported data collection, and all research team members (EOO, RG, and PMO) engaged in regular peer debriefing sessions to ensure consistency throughout the collection, analysis, and interpretation phases.

### Ethical considerations

Ethics approval was granted by the University of Alberta Research Ethics Review Board (Protocol: Pro00138854) and the AMREF Kenya Research Ethics Board (ESRC P1670/2024). Verbal consent was obtained from participants who were unable to provide written consent. All participants provided informed consent (verbal or written) and received an honorarium of CAD $10 (KES 1,000) for their participation.

## Results

The results are presented in two sections: first, a summary of participant socio-demographic characteristics; second, a thematic analysis integrating policy documents with qualitative findings.

### Socio-demographic characteristics of study participants

The qualitative study participants comprised 67 (83.8%) women and 13 (16.2%) men food vendors. Participants ranged in age from 25 to 65 years (mean = 40.3, SD = 8.5). Over half (52.5%) of the participants had primary-level education or less, while one-quarter (25.3%) held tertiary qualifications education (college, diploma and university). Most participants (61.2%) were married, had at least one child, and lived with extended family, indicating additional care responsibilities during the pandemic. For most, informal trading was the primary income source, highlighting their dependence on food vending to sustain their livelihoods and households (Table 2).

Participants managed diverse food-related enterprises, including the sale of fresh produce, cereals (e.g., maize, sorghum, and rice), prepared meals, meat products (e.g., beef, pork, poultry, and fish), and various beverages. They operated from a variety of structures, ranging from makeshift platforms and hand-carts to permanent stalls and small hotels. While many vendors lacked formal licenses or permits, most paid daily fees to the county government. To manage financial constraints and sustain operations, a significant proportion of participants maintained memberships in market associations or informal savings groups (*chamas*), which facilitated access to credit.

### Key thematic areas

Our analysis of COVID-19 control policies and qualitative data identified four key themes that highlight how informal food vendors experienced these measures, as well as the related sociocultural and gender disparities. These themes are: (1) vendor engagement in policy decision-making; (2) inequities in policy enforcement; (3) policy blind spots regarding vendors’ intersecting identities; and (4) policy fragmentation. The following section details these themes, supported by participant quotes and policy excerpts.

1. Lack of engagement of informal food vendors in policy decision making

Using the IBPA framework’s multi-level analysis principle, our review of policy documents and qualitative data revealed that informal food vendors were systematically excluded from policy decision-making, resulting in a structural omission of their voices and lived experiences. Health and hygiene protocols—such as masking, sanitization, curfews, social distancing, and market closures—were implemented without prior consultation. Many participants reported that these top-down mandates imposed unanticipated expenses, a sentiment reflected in the following accounts:

*According to me since it caught us unaware, we were forced and remember more expenses came with it because you must buy a mask and a sanitizer. When your water was used up you must go and buy some so that your clients would wash their hands, so we never did it because of flexibility but we were forced to them.* ***(FGD-01-P3).***

*We were also forced to put on masks and without the masks, we could not come to the market, there were times when we were told to close our shops and stay at home yet most of us depended on business, so it was so hard and us business people of (Okony Welo)* ***(KII-05).***

Additionally, women traders were excluded from policy discussions despite their membership in market associations and informal savings groups (chamas). These community-based groups pool member contributions on a regular basis to provide savings and lending services—a vital resource given that most informal traders lack access to formal financial institutions. Beyond supporting business recovery and growth, these groups possess rich local knowledge regarding food vending, financial management, food security, mobility, and caregiving. Such insights could have informed policy formulation to ensure vendors’ roles and needs were adequately addressed. A majority of participants reported active membership in these market groups:

*We saw to it that being together is good. Even if you own a business, it will not be enough and there was a need for a group, where we have a merry-go-round and welfare, among other things, since we also have children that need to go to school as well. Together we can achieve a lot. So, we have such things in our project; merry-go-round, welfare, and table banking.* ***(KII-01).***

The exclusion from decision-making left many women vendors unprepared to meet new regulatory requirements. For example, mandatory physical distancing protocols significantly reduced the operational capacity of food businesses, forcing vendors to reorganize their premises. These challenges, confirmed by both key informant interviews and vendor accounts, highlight the practical difficulties caused by top-down policy implementation.

*First, you know, the informal or the small-scale businesses, you find that somebody even has just a small table and maybe they are able to accommodate even only four people at a time in their business... Because of the* *COVID-19 regulations, they could not even hold four at a go because of the spacing..****. (KII-15_County Nursing Officer).***

Similarly, the vendors also narrated how the county government of Kisumu increased the market licensing fees during the pandemic without consulting the traders:

*As for the abrupt increase in 500 shillings’ payment, they should consult with the public before rushing to a decision. Whenever they want to implement something in the market, they should first come to talk to our leaders … and a decision to be made. There ought to be an agreement.* ***(FGD-02).***

In illustrating the top-down approach frequently adopted in the development and enforcement of public health orders, a review of the policy environment during the pandemic revealed systemic barriers to the engagement of informal food vendors, particularly women business owners. Technical experts and government officials formulated policies at the national level without consulting vendors or vendor associations. These regulations were subsequently implemented at the county level. The excerpt below shows how power structures shaped policy processes:

*The Cabinet Secretary may, by notice in the Gazette and in a newspaper with a wide circulation, put in place such measures as may from time to time become necessary to contain and curb the further spread of* *COVID-19.* ***Public Health Act Public Health (Prevention, Control and Suppression of Covid-19) Rules, 2020, Rule 14.***

2. Inequities in policy enforcement

Guided by the IBPA principle of equity and social justice, our findings reveal that the enforcement of curfews and movement restrictions further entrenched gendered power imbalances, with vulnerable populations disproportionately bearing the burden. Enforcement, involving national and county authorities—including police, municipal officers, and public health officials—created uneven power dynamics. Throughout the pandemic, women vendors reported widespread inequitable treatment, extortion, physical assault, and coercion by these authorities. Participants often described being threatened with arrest unless they paid bribes, as illustrated by the following account:

*Concerning the police too, there were times when I used to go to the lake early in the morning, the police caught me on the road, and they arrested me and ordered me to pay five thousand shillings (5 dollars). I told them to take me to court, but they insisted that they need money, but I just told them to take me to the court of which when they did, I was only fined five hundred shillings (5 dollars)  ....* ***(1D1-09)***

Women vendors recounted how they faced abuse at their places of businesses from the municipal officers and the police, who ate, confiscated or destroyed their food items. Some women reported being physically abused from trying to escape arrest, while others shared how the police took food items from their stalls without paying.

*At that time, I would make sales as someone would come and make a purchase in a hurry when police would find that you have food items, they consume and on that particular day that they ate my pineapples, they also took carrier bags which I sold and carried some pineapples in them* ***(IDI-10).***

Furthermore, strict top-down enforcement imposed severe penalties that were difficult for informal vendors to navigate. Labeling these businesses as “informal” turned the marketplace into a contested space, where the high cost of non-compliance proved devastating for women vendors who relied on these operations for their livelihoods and to support urban household food security.

*Police kept throwing teargas at us and there was I day I fell in a terrace that was flooded since it had rained and the water swept away my fingerlings together with the cash that I had wrapped in a polythene paper. It was not easy but too hard* ***(FGD-02).***

The punitive nature of the control measures and the regulations are also reflected within the regulations as the fines could push small-scale businesses such as the food vendors out of business.

*Except as otherwise provided in these Rules, any person who contravenes the provisions of these Rules shall be liable to a fine not exceeding twenty thousand shillings (200 dollars), or to imprisonment for a term not exceeding six months, or to both. —* ***Public Health Act Public Health (Prevention, Control and Suppression of Covid-19) Rules, 2020, Rule 15 (1).***

3. Policy blind spots to intersecting identities of the informal food vendors

Drawing on the IBPA principle of *intersecting categories* [36]—which emphasizes how overlapping factors shape individual experiences—this analysis highlights the unique challenges faced by women informal food vendors. Pandemic policies consistently overlooked these vendors’ diverse social identities, including differences in age, socio-economic status, education, marital status, and cultural background. Instead, policies assumed informal food traders were a homogenous group, a bias evident in both the official policy language and the vendors’ lived experiences.

*Every person who is in a Public Place shall—(b) wear a facemask that covers the mouth and nose. —* ***The Public Health (Covid-19 General Public Safety) Rules, 2020, Rule 3 (1) (b).***

*Every organisation, business entity or any other trader, whether in an open-air market, enclosed premises or any public place shall—(a) provide a handwashing station with soap and water or an alcohol based sanitizer approved by the Kenya Bureau of Standards for use at the entrance of its premises—* ***The Public Health (Covid-19 General Public Safety) Rules, 2020, Rule 3(2) (a).***

Health safety protocols, such as mandatory masking and handwashing, assumed that individuals across all genders possessed equal financial and physical capacity to comply. However, women vendors reported having to independently purchase masks, water, and sanitizing supplies daily without government support. These policies overlooked the financial hardship faced by women in the informal sector, whose income and operations were already severely constrained by curfews, reduced operating hours, and broader supply chain disruptions. As several women vendors described:

*We were forced to  ... buy hand-washing items so it forced us leaders to buy water tanks for people to wash their hands when they came to shop/market. We were also forced to put on masks and without the masks, we could not come to the market, there were times when we were told to close down our shops and stay at home yet most of us depended on business,  ..it was hard  ....* ***(KII-05).***

Furthermore, these policies failed to account for prevailing domestic social dynamics. A significant number of the interviewed vendors were the sole providers for their households; consequently, the decline in business profitability directly compromised their ability to secure basic necessities for themselves and their dependents. Using the IBPA framework, we can see how gender intersected with other social determinants—such as marital status, employment informality, and socioeconomic standing—to exacerbate the systemic inequalities faced by female informal vendors, as illustrated in the following Key Informant Interview (KII) narrative:

*…either they are selling greens, they are selling tomatoes, they are selling the onions, some of them are doing the chips/fries business, some of them are doing the fish business... So, when COVID came about, all these businesses ceased. You find these are young women, they are mostly eighteen to twenty-four years of age, pregnant, parenting, and they did not have any other source of income.  ...They live hand to mouth through these businesses....* ***(KII-11).***

Analysis indicated that prevailing societal norms within the study area assigned primary household and caregiving responsibilities to women, while men provided minimal support. However, policy frameworks failed to acknowledge the additional burdens sustained by women vendors. Participants noted that this caregiving burden was particularly pronounced for single or unmarried women with children or ailing family members. Consequently, a majority of women reported the need to modify business operations and working hours to accommodate these substantial, unpaid domestic obligation.

*During corona for someone like me who have kids and had no one to help, it forced me to wake up early  ...to wash, and clean the house in the morning.  ...There are things that I have in the house like chicken and other domestic animals that I have to take care of as well....* ***(IDI-09).***

Additionally, climate-related events compounded the risks for the women in the informal food sector during the pandemic. For instance, in Kisumu, the COVID-19 pandemic overlapped with climate risk of flooding, with vendors losing their goods amidst the lockdowns, adding to their psychological burden.

*So, when water got into my house, I recently had taken a loan and stocked some items, water then got into my house with the items in there; wheat flour, charcoal in bulk. Flood got in when I was not around and it even covered the seats* ***(FGD-01).***

4. Policy fragmentation

Guided by the IBPA principles of power and multi-level analysis, we examined how governance structures shaped policy outcomes for the study population. Although the national government in Nairobi formulated public health policies, county authorities were responsible for implementing them. Our findings highlight enforcement inconsistencies, as Kisumu County government officials mandated market closures while the National Police Service enforced curfew orders, with both entities operating independently.

*During COVID, the police were enforcing the law, and the traders were not coping with them because of things like curfew. They felt like they were being denied a right and means of generating their earnings* ***(KII-03).***

The overlapping policy interventions created an avenue for extortion and harassment of the women vendors:

*The police too knew that when they go to a certain place, traders did not have a license so they would come and harass me a bit and I would be forced to give them something for them to leave because you also have your target* ***. (KII-06).***

Additionally, the curfew rules required business not to operate after 7:00 PM, while the restriction of movement rules allowed for the transportation of food commodities outside curfew hours.

*This Order shall apply during the hours of darkness between seven o’clock in the evening and five o’clock in the morning with effect from the 27th of March 2020 and shall remain in effect for a period of thirty days thereof—* ***The Public Order (State Curfew) Order, 2020, Order 3***

*During the restriction period, there shall be no movement of persons into or out of an infected area, either by road, rail or air, unless the movement into or out of an infected area is for the purpose of — (a) transportation of food and farm produce for commercial purposes including rice, beans, maize, potatoes, cassava, cabbages, kale, spinach, tomatoes, onions, bananas, eggs...* ***Public Health (Covid-19 Restriction of Movement of Persons and Related Measures) Rules, 2020, Rule 4 (1) (a).***

## Discussion

This study examined the sociocultural and gender disparities in the formulation and implementation of COVID-19 mitigation policies and their impact on women in the informal food vending sector. The findings reveal a top-down approach that restricted the engagement of women vendors. Policies were fragmented across governance levels, exacerbating inequities and overlooking the diverse, intersecting identities of vendors. Women were systematically excluded from decision-making due to structures that delegate service delivery to male-dominated county governments, coupled with local systemic barriers that reinforce power hierarchies. Our research identified critical obstacles to women’s participation in Kisumu’s COVID-19 policy process, including crisis-related time constraints, sociocultural and gender inequities, limited information access, and a reliance on technocratic expertise. These findings echo Martínez and Young [35], who observed that prioritizing technical experts often excludes street vendors from policy development. Furthermore, deep-seated patriarchal norms continue to impede women’s political and leadership involvement; despite constitutional affirmative action, policy and community leadership remain predominantly male-dominated. These structural barriers perpetuate gender hierarchies, marginalizing women across all levels of governance. Consequently, this lack of representation hindered the development of policies responsive to the specific needs of women informal vendors, ultimately intensifying existing socioeconomic disparities [54].

Vendors consistently described this exclusion using the phrase “forced on us,” highlighting their marginalization and the absence of prior consultation on policy initiatives. Such exclusionary practices may contradict Article 174(c) of the Constitution of Kenya, which mandates public participation in decision-making processes [55]. While research shows that the political empowerment of women in pandemic policy-making improves gender-sensitive health and economic outcomes [56,57], the approach in Kisumu—and similar cities in the Global South, such as Rangpur in Bangladesh, and Rusizi and Rubavu in Rwanda—remained highly centralized under the national Ministry of Health. Consequently, the development and implementation of mitigation measures relied solely on technical experts and government officials, omitting essential community engagement, gender-responsive planning, and feedback mechanisms for women in the informal food economy.

Our findings demonstrate that COVID-19 policies reinforced existing inequalities by ignoring the diverse, intersecting identities and lived realities of women informal food vendors. These enforcement practices highlight how gendered power relations influenced the application of pandemic measures within informal urban economies. Solymári et al. [58] similarly noted that the brutality inherent in enforcement efforts within Kenyan informal settlements often infringed upon fundamental human rights. In Kisumu, this was exacerbated by a male-dominated county enforcement team, which contributed to biased policy application [59]. This enforcement culture increased the risk of violence and physical abuse at business sites, threatening vendors’ health, safety, and economic autonomy. Such patterns of harassment and excessive force, including instances where vendors reported paying bribes of up to five thousand shillings to avoid arrest, underscore how entrenched power structures directly undermined the security and livelihoods of women in the informal food sector.

Similarly, research by Wandera et al. [60] in Nairobi documents that women in the informal workforce were subjected to physical abuse by police and municipal officers while enforcing curfews, suggesting a consistent pattern of adverse COVID-19 policy impacts on women vendors in both primary and secondary urban centers. These findings are corroborated by Kiaka et al. [43], who highlight that the pandemic exacerbated bribery and harassment against informal vendors across the Global South. This ongoing exposure to violence and extortion restricted the agency of women to provide urban household food security. Consequently, the current study underscores the necessity for gender-responsive governance during public health emergencies, ensuring that policies actively include and address the specific experiences of this marginalized yet essential segment of the urban population.

Our study provides further evidence of the lack of an intersectional perspective in the formulation of COVID-19 policies. Informal traders in Kisumu represent a heterogeneous group with diverse social identities—including gender, age, socio-economic status, cultural background, marital status, ability, and religious affiliation. However, pandemic mitigation strategies employed a ‘blanket’ approach that failed to account for the specific needs and diverse identities of these vendors. Similar findings were reported by Dutta et al. [61] in India, where COVID-19 lockdown policies adopted a one-size-fits-all model that overlooked existing gender-based differences.

These policy interventions failed to account for contextual factors in low- and middle-income countries (LMICs), such as pre-existing inequalities like patriarchal structures and gender-based discrimination faced by women informal workers. Before the COVID-19 pandemic, these women already encountered compounded challenges, including business instability, gender-based violence, discrimination, economic marginalization, and sexual harassment. Policies lacking gender-sensitive considerations exacerbated these inequalities, creating additional burdens for women vendors and hindering their post-pandemic resilience. While acknowledging the pandemic’s urgency and the necessity of immediate public health measures, we argue that governments must balance this urgency with the inclusive, equitable engagement of women in policy decision-making to mitigate unintended negative impacts.

Women faced societal pressures to assume additional caregiving responsibilities at the expense of their paid employment. Significantly, this study highlights critical concerns regarding the undervaluation of women’s unpaid domestic and caregiving labor. Data from the UNDP-UN Women COVID-19 Global Gender Response Tracker indicate that fewer than one-third of COVID-19 policy measures in Kenya incorporated a gender perspective, and none adequately addressed unpaid care work [62,63]. Our findings contribute to this discourse by illustrating how these policy gaps specifically impacted women in secondary cities.

Furthermore, the rapid influx of new COVID-19 regulations, compounded by strict enforcement and the use of force, overwhelmed vendors and induced ‘compliance fatigue.’ This aligns with findings by Martínez and Young [35], which demonstrate that excluding vulnerable groups from policy formulation and implementation undermines effectiveness and exacerbates marginalization. Such evidence underscores the need for future emergency policy to prioritize the meaningful engagement of all stakeholders to ensure equitable outcomes. This approach is consistent with the principle of ‘nothing for us without us’ [64], which advocates for the substantive participation of vulnerable groups in both policy design and execution.

Furthermore, the rapid influx of new COVID-19 regulations, compounded by strict enforcement and the use of force, overwhelmed vendors and induced ‘compliance fatigue.’ This aligns with findings by Martínez and Young [35], which demonstrate that excluding vulnerable groups from policy formulation and implementation undermines effectiveness and exacerbates marginalization. Such evidence underscores the need for future emergency policy to prioritize the meaningful engagement of all stakeholders to ensure equitable outcomes. This approach is consistent with the principle of ‘nothing for us without us’ [64], which advocates for the substantive participation of vulnerable groups in both policy design and execution.

This study has several limitations that warrant consideration. First, the cross-sectional design limited data collection to the period between July 10 and October 15, 2024. As this took place years after the pandemic, participant recall of specific details may be affected. Second, the geographic focus on a single county may limit nationwide generalizability; however, these findings are likely transferable to urban centers with characteristics comparable to Kisumu City. Third, the study was confined to the informal food economy, which may restrict generalizability to other informal sectors. Furthermore, reliance on self-reported data may introduce social desirability bias, particularly regarding reflections on pandemic-era challenges and policy disparities. Despite these limitations, the methodological approach aligns with existing scholarship. These findings offer significant insights into the gendered dynamics of the informal food sector in secondary cities across the Global South.

## Conclusions

In conclusion, this study makes significant theoretical, methodological, and practical contributions to the literature on urban informal economies during public health emergencies. Theoretically, by utilizing the IBPA framework, this research advances intersectionality-informed policy analysis. It reveals how blanket COVID-19 mitigation policies failed to account for the heterogeneous, intersecting identities of women food vendors, reinforcing pre-existing socio-economic and cultural disparities. By documenting these gendered experiences of exclusion in both policy formulation and enforcement, the study provides a critical empirical basis for shifting governance toward gender-responsive, inclusive frameworks that prioritize the substantive engagement of marginalized populations.

While the COVID-19 pandemic necessitated immediate public health interventions, our findings emphasize the imperative for inclusive policy-making processes. Governments should adopt gender-responsive governance by formally representing women vendors on emergency advisory committees and mandating consultation with community partners before implementing market restrictions. Through such deliberate, participatory initiatives, governments can better accommodate social diversity, respond to localized needs, and safeguard the vital contributions of informal vendors to urban food security.

Methodologically, this study demonstrates the value of integrating the IBPA framework with policy document reviews and qualitative evidence. This approach highlights how policies intersect with lived experiences, underscoring the importance of qualitative data in capturing the human impact of policy, particularly regarding socioeconomic and gender disparities in secondary cities. Substantively, our research indicates that pandemic containment measures were enforced via top-down methodologies that marginalized female informal food vendors by failing to address their unique needs and intersecting identities. Furthermore, regulatory measures—including curfews, stay-at-home mandates, and physical distancing protocols—exacerbated pre-existing power imbalances and gender-based vulnerabilities.

To address these gaps, policy interventions must recognize the unique roles of women informal food vendors by incorporating targeted economic support, such as financial assistance, flexible licensing, and emergency income protection. Implementing flexible market regulations and tailored communication strategies during public health emergencies will further enhance gender responsiveness and mitigate structural inequalities. Future research should build on this analysis through comparative studies across secondary cities to evaluate how intersectional inequalities shape the experiences of informal food vendors under different governance frameworks.
