# Supplementary material for: An intersectionality-based policy analysis of COVID-19 measures and women informal food vendors in Kisumu, Kenya
Source: PLoS One. 2026 Oct 1;21(10):e0359620. doi: 10.1371/journal.pone.0359620 (PMC13630232; doi:10.1371/journal.pone.0359620)
Supplement: Supplementary file 1 [file pone.0359620.s002.docx]

**Response to Editor and reviewers comments**

**NB: Highlighted in yellow are the edited/revised sections in the paper.**

**NB: Highlighted in blue are the editor/reviewers comments and suggested edits**

**NB: Highlighted in gray are our responses to the suggestions/comments**

1. When submitting your revision, we need you to address these additional requirements.

Please ensure that your manuscript meets PLOS ONE's style requirements, including those for file naming. The PLOS ONE style templates can be found at

<https://journals.plos.org/plosone/s/file?id=wjVg/PLOSOne_formatting_sample_main_body.pdf> and

[https://journals.plos.org/plosone/s/file?id=ba62/PLOSOne_formatting_sample_title_authors_affiliations.pdf](https://track.editorialmanager.com/CL0/https:%2F%2Fjournals.plos.org%2Fplosone%2Fs%2Ffile%3Fid=ba62%2FPLOSOne_formatting_sample_title_authors_affiliations.pdf/1/010f019b43f817ea-9d18d8dd-247e-4089-9d3a-4a384c351f9b-000000/dq7qz4edAkcVmmzZu8KIwKkHrBchNCY15L8KSVmBgfo=242)

 Checked and edits incorporated

2. Please include a complete copy of PLOS’ questionnaire on inclusivity in global research in your revised manuscript. Our policy for research in this area aims to improve transparency in the reporting of research performed outside of researchers’ own country or community. The policy applies to researchers who have travelled to a different country to conduct research, research with Indigenous populations or their lands, and research on cultural artefacts. The questionnaire can also be requested at the journal’s discretion for any other submissions, even if these conditions are not met.  Please find more information on the policy and a link to download a blank copy of the questionnaire here: <https://journals.plos.org/plosone/s/best-practices-in-research-reporting>. Please upload a completed version of your questionnaire as Supporting Information when you resubmit your manuscript.

- **Included as an attachment!**

3. PLOS requires an ORCID iD for the corresponding author in Editorial Manager on papers submitted after December 6th, 2016. Please ensure that you have an ORCID iD and that it is validated in Editorial Manager. To do this, go to ‘Update my Information’ (in the upper left-hand corner of the main menu), and click on the Fetch/Validate link next to the ORCID field. This will take you to the ORCID site and allow you to create a new iD or authenticate a pre-existing iD in Editorial Manager.

**ORCID iD**

- Joyce Kiplagat - No account
- Patrick Mbullo Owuor - https://orcid.org/0000-0002-4550-2667
- Rebecca Gokiert - <https://orcid.org/0000-0002-7472-9545>
- Elizabeth Opiyo Onyango - <https://orcid.org/0000-0002-2736-0108>

4. Thank you for stating the following financial disclosure:
   This research was funded by SSHRC-IDG Grant # 430-2023-00856, and a sub-grant from the Social Sciences and Humanities Research Council of Canada (SSHRC grant no. 895-2021-1004) from Wilfrid Laurier University. Please state what role the funders took in the study. If the funders had no role, please state: "The funders had no role in study design, data collection and analysis, decision to publish, or preparation of the manuscript."
   If this statement is not correct you must amend it as needed.
   Please include this amended Role of Funder statement in your cover letter; we will change the online submission form on your behalf.

**"The funders had no role in study design, data collection and analysis, decision to publish, or preparation of the manuscript."**

5. Please expand the acronym “SSHRC-IDG” (as indicated in your financial disclosure) so that it states the name of your funders in full.
   This information should be included in your cover letter; we will change the online submission form on your behalf.

**Social Science and Humanities Council – Insight Development Grant (SSHRC –IDG). Is spelt out in the financial disclosure section.**

6. Thank you for stating the following in the Competing Interests section:
   The authors declare no conflicts of interest with this paper’s research, authorship, and/or publication.

   Please confirm that this does not alter your adherence to all PLOS ONE policies on sharing data and materials, by including the following statement: "This does not alter our adherence to PLOS ONE policies on sharing data and materials.” (as detailed online in our guide for authors <http://journals.plos.org/plosone/s/competing-interests>). If there are restrictions on sharing of data and/or materials, please state these. Please note that we cannot proceed with consideration of your article until this information has been declared.

   Please include your updated Competing Interests statement in your cover letter; we will change the online submission form on your behalf.

**The statement is included! Thank you!**

7. In the online submission form, you indicated that data supporting reported results in this study are hoisted on the University of Alberta Google Drive and are not publicly available currently but are available on request.

All PLOS journals now require all data underlying the findings described in their manuscript to be freely available to other researchers, either a. In a public repository, b. Within the manuscript itself, or c. Uploaded as supplementary information.
This policy applies to all data except where public deposition would breach compliance with the protocol approved by your research ethics board. If your data cannot be made publicly available for ethical or legal reasons (e.g., public availability would compromise patient privacy), please explain your reasons on resubmission and your exemption request will be escalated for approval.

**Vcjk;lx]mlnData Availability Statement:** Data supporting reported results in this study are hosted on the University Of Alberta Google Drive and are available on request. This is because of the REB approval in which participants were assured that the raw data will not be publicly available and will only be accessed by the research team.

8. Please amend your authorship list in your manuscript file to include all authors.

**Noted!**

9. Please amend your list of authors on the manuscript to ensure that each author is linked to an affiliation. Authors’ affiliations should reflect the institution where the work was done (if authors moved subsequently, you can also list the new affiliation stating “current affiliation:….” as necessary).

**Amended!**

10. **Please include captions for your Supporting Information files at the end of your manuscript, and update any in-text citations to match accordingly. Please see our Supporting Information guidelines for more information:** [**http://journals.plos.org/plosone/s/supporting-information**](http://journals.plos.org/plosone/s/supporting-information)**.**

**Done!**

11. 11. Please remove all personal information, ensure that the data shared are in accordance with participant consent, and re-upload a fully anonymized data set. Note: spreadsheet columns with personal information must be removed and not hidden as all hidden columns will appear in the published file. Additional guidance on preparing raw data for publication can be found in our Data Policy (<https://journals.plos.org/plosone/s/data-availability#loc-human-research-participant-data-and-other-sensitive-data>) and in the following article: <http://www.bmj.com/content/340/bmj.c181.long>.

The data in this study is available but give the REB approval in which we assured the study participants that the raw data will only be available to the research team and only the summaries and de-identified quotes would be shared publicly in project report and other publication, we are unable to share the raw data publicly.

12. If the reviewer comments include a recommendation to cite specific previously published works, please review and evaluate these publications to determine whether they are relevant and should be cited. There is no requirement to cite these works unless the editor has indicated otherwise.

**Noted!**

13. Please review your reference list to ensure that it is complete and correct. If you have cited papers that have been retracted, please include the rationale for doing so in the manuscript text, or remove these references and replace them with relevant current references. Any changes to the reference list should be mentioned in the rebuttal letter that accompanies your revised manuscript. If you need to cite a retracted article, indicate the article’s retracted status in the References list and also include a citation and full reference for the retraction notice.

**Noted! And revised accordingly!**

**Additional Editor Comments:**

The manuscript requires a little bit of work to put it in a better form for publication, this work is majorly some additions to the body of the literature of the paper, to make it more engaging and also improve its structure. Writers are encouraged to adhere to the recent review comments and make the necessary adjustments.

**Well noted and addressed as shown bellow!**

Reviewers' comments:

Reviewer's Responses to Questions

**Comments to the Author**

1. Is the manuscript technically sound, and do the data support the conclusions?

The manuscript must describe a technically sound piece of scientific research with data that supports the conclusions. Experiments must have been conducted rigorously, with appropriate controls, replication, and sample sizes. The conclusions must be drawn appropriately based on the data presented.

Reviewer #1: Yes

Reviewer #2: Yes

2. Has the statistical analysis been performed appropriately and rigorously?

Reviewer #1: N/A

Reviewer #2: N/A

3. Have the authors made all data underlying the findings in their manuscript fully available?

The [PLOS Data policy](http://track.editorialmanager.com/CL0/http:%2F%2Fwww.plosone.org%2Fstatic%2Fpolicies.action%23sharing/1/010f019b43f817ea-9d18d8dd-247e-4089-9d3a-4a384c351f9b-000000/dBah7TzIqXBopw0uG9G6e5-GseuqlXAZkVmlfNfIzus=242) requires authors to make all data underlying the findings described in their manuscript fully available without restriction, with rare exception (please refer to the Data Availability Statement in the manuscript PDF file). The data should be provided as part of the manuscript or its supporting information, or deposited to a public repository. For example, in addition to summary statistics, the data points behind means, medians and variance measures should be available. If there are restrictions on publicly sharing data—e.g. participant privacy or use of data from a third party—those must be specified.

Reviewer #1: Yes

Reviewer #2: Yes

4. Is the manuscript presented in an intelligible fashion and written in standard English?

PLOS ONE does not copyedit accepted manuscripts, so the language in submitted articles must be clear, correct, and unambiguous. Any typographical or grammatical errors should be corrected at revision, so please note any specific errors here.

Reviewer #1: Yes

Reviewer #2: Yes

5. Review Comments to the Author

Please use the space provided to explain your answers to the questions above. You may also include additional comments for the author, including concerns about dual publication, research ethics, or publication ethics. (Please upload your review as an attachment if it exceeds 20,000 characters)

Reviewer #1: Reviewer’s Comments

This is a well-written, relevant, and timely manuscript that provides valuable insights into how COVID-19 control measures intersected with gender and informality in the Global South, focusing on women informal food vendors in Kisumu, Kenya. The authors make an important contribution by applying the Intersectionality-Based Policy Analysis (IBPA) framework to an under-researched context, i.e. secondary African cities.

The authors can consider the following comments to improve their work:

1. **In the introduction Clarify how the IBPA framework adds explanatory power compared to other gender-policy frameworks. Also, include a brief statement of objectives or research questions at the end of the introduction section for clearer reader orientation.**

Thank you for this valuable feedback. We have incorporated this and a section of the introduction now reads as follows:

.....

To explore how COVID-19 control policies accounted for and addressed the unique challenges and needs of women working in informal food businesses and whether their perspectives were considered in policy formulation and implementation in Kisumu City, this study adopted the intersectionality-based policy analysis framework (IBPA) (Hankivsky et al., 2014). The study utilized the seven principles of the IBPA framework including equity, power, intersecting categories, multi-level analysis, reflexivity, social justice, and diverse knowledges (Hankivsky et al., 2014). These guided the analysis of the COVID-19-related policies, key informant interviews, focus group discussions and in-depth interview transcripts, as well as the interpretation of the findings

2. **Also consider adding a concise literature review section just after the introduction that synthesizes global/African evidence on COVID-19, gender, and informality, intersectionality-based policy analyses, and prior IBPA applications to anchor the study’s concepts and contributions. Emphasize the documented gaps in secondary cities and in women vendors’ participation to justify Kisumu’s focus and show how your IBPA approach advances this literature.**

I appreciate this feedback. We have incorporated additional context literature to strengthen the introduction and create some foundation for the IBP framework and the subsequent sections of the paper. The introduction now reads as follows:

**Introduction**

The COVID-19 pandemic had devastating effects on global trade and markets, which affected both the formal and informal economies (Alon et al., 2022; Alon et al., 2020; Mishra & Rampal, 2020). Following the WHO declaration of the global pandemic, governments across the world imposed mandatory containment measures such as lockdowns and curfews (Koh, 2020). Additionally, safety protocols such as the use of face masks, handwashing, sanitizing, and physical distancing were introduced to curb the spread of coronavirus (Mwakisha et al., 2024). The pandemic control policies triggered events that shaped social and economic outcomes differently for men and women engaged in the informal economy (Kabeer et al., 2021).

As pointed out in most COVID-19 related literature, the pandemic exposed and exacerbated socioeconomic and cultural inequalities in most urban populations with women bearing a disproportionate burden (Finn, 2023; Finn & Kobayashi, 2020; Odhiambo et al., 2025; Onyango et al., 2021; Sharifi & Khavarian-Garmsir, 2020; Ukala et al., 2020). Women food vendors experienced varied challenges including reduced market hours, restricted movement, disruption of food supply chains, increased household and caregiving responsibilities, loss of income, and food insecurity (Afifu et al., 2021; Kimani-Murage et al., 2022). This is because most of the women-led informal food businesses rely on rich social interactions and close proximity engagements and yet these were restricted with the pandemic control measures (Finn & Kobayashi, 2020). The measures deepened existing inequalities due to the lack of consideration of socio-cultural and economic challenges faced by women (Odhiambo et al., 2025; Ukala et al., 2020). Additionally, lack of consideration of gender inequities in policy responses presented unique problems for women during the pandemic (UN Women, 2024). Economically, women informal food vendors faced challenges in accessing financial support from government and financial institutions (Donga & Chimucheka, 2024; Sinyolo et al., 2022). In the African context, these were particularly pronounced due to embedded socio-cultural practices and the dominance of patriarchy, which undermined women’s possession and authority over assets that could act as collateral in financial access (Fletschner & Kenney, 2014; Kiplagat et al., 2025).

In most urban centers of the Global South, women are over-represented in the informal economy, particularly within the food sector (Donga & Chimucheka, 2024). In Kisumu City for example, women make up to 60% of all the informal traders, majorly engaged in food vending (Kisumu, 2020; Opiyo et al., 2018). Yet, the COVID-19 policies did not consider the unique challenges of women in the informal food sectors leading to in increased gender and intimate partner violence, hindering the contributions of women-led informal food businesses in supporting urban food needs and livelihoods (Afifu et al., 2021; John et al., 2021; Roever & Skinner, 2016; Smith, 2023). Simultaneously, increased unpaid care work and household responsibilities impeded women’s full engagement in paid work (Wenham et al., 2020).

In secondary cities in the global South such as Kisumu City, the experiences with the pandemic control measures were not any different, yet limited studies have explored gender-specific gaps in COVID-19 control policies in such contexts. In particular, the impacts of the control measures on women-led informal food businesses in secondary cities remains an underexplored research area (Malou, 2024). Most existing studies have focused on the broader informal sector in major cities of the global South, including Nairobi, Kampala, Karachi, West Bengal, and Johannesburg (Afifu et al., 2021; Anwar et al., 2023; Mondal & Chakraborty, 2022; Pinchoff et al., 2021; Rwafa-Ponela et al., 2022; Suubi et al., 2022). Policy analyses on COVID-19 in Durban, Nairobi and Kampala showed that women in the urban informal economy were excluded from COVID-19 policy processes (Afifu et al., 2021; Skinner et al., 2021; Suubi et al., 2022). In Karachi, the findings of an intersectional analysis revealed that lockdowns resulted in gendered inequalities with women experiencing increased domestic violence and care responsibilities while also maintaining provisioning role to their households (Anwar et al., 2023). In West Benghal, Mondal and Chackraborty (2022) argued that women informal workers had limited influence on policy decisions during the pandemic. As Martínez and Young (2022) note, there is a dearth of research in secondary cities on the impacts of pandemic-related policies on women in the informal food sector.

Secondary cities have unique roles in fostering urban-rural linkages within the social, economic, and cultural spheres. In terms of policy development, these cities are often characterized by informality and are marginalized, especially during emergencies (Kiplagat et al., 2025). In the case of Kisumu City, COVID-19 policies were formulated at the national government level in Nairobi, a primary city, excluding the lived realities of vulnerable populations in secondary cities, particularly women.

Kisumu City is the economic hub for western Kenya and the city’s economy is largely driven by informal trade (Opiyo et al., 2018). Over 60% of the informal businesses in the city are female owned with majority living in the informal settlements and identifying as being from the Luo or the Luhya speaking communities (Kiplagat et al., 2025). In these communities, gender roles and responsibilities are well-defined with care work and household chore relegated to women and girls who are less-valued due to patriarchy. With the pandemic control measure, the women-led businesses in the city were significantly impacted as additional care responsibilities emerged with closure of schools and sick family members requiring care (Kiplagat et al., 2025; Odhiambo et al., 2025). Additionally, with the existing power imbalance between the males and females in the communities and the over representation of males in decision-making positions, the female voices, particularly those in the informal food sector were less likely to be reflected within the pandemic control measures.

This paper extends and enables our understanding of the extent to which the perspectives of women business-owners were considered in the formulation and implementation of the pandemic control policies in small-to-medium sized cities in the global South. Understanding the gendered dimensions of the COVID-19 policies on women informal food vendors secondary cities is key in recommending inclusive policy responses that supports the food security needs and livelihoods of urban households in policy formulation, and more so, in times of emergencies.

3. **The IBPA framework is described well, but a short conceptual figure or table summarizing the seven principles and how they guided analysis would improve clarity.**

Thanks for this suggestion! A conceptual framework for the IBPA framework guiding principles to the study is included in the paper. A section of the introduction now reads as follows and with the conceptual framework reflected below.

………

The study adopted the intersectionality-based policy analysis framework (IBPA), utilizing the principles of equity, power, intersecting categories, multi-level analysis, reflexivity, social justice, and diverse knowledges (Hankivsky et al., 2014). These seven IBPA framework principles (See Figure 1 below) guided the analysis of the COVID-19-related policies, key informant interviews, focus group discussions and in-depth interview data as well as the interpretation of the findings. We applied the principle of intersecting categories to explore their various overlapping social identities, such as gender, age, sexual orientation, and marital status, level of education, cultural orientation, and socio-economic status. The intersectional lens was meant to identify how the social locations influenced the women experiences with the policies and understand how inequalities are shaped by how policies are designed, disseminated and understood (Kanenberg & Leal, 2019).

Considering the principle of power and multi-level analysis, we sought to highlight how overlapping power structures fostered inclusion or reinforced inequality for the informal women food vendors. The gender equity lens and the principle of social justice was also applied to understand the gender inclusivity of the COVID-19 policies while considering the overlapping layers of power in policy processes. Furthermore, by applying the principle of diverse knowledge, the framework enabled us to interrogate whether the voices of women in informal food vending were integrated in policy development and how power dynamics shaped the inclusion or exclusion of their diverse perspectives in policy decision making. Whilst studies in other jurisdictions showed that governments overlooked gender-specific needs of women in COVID-19 policy responses (Chitando, 2020), the IBPA framework allowed for the advancing of the principle of social justice. The principles were applied to the analysis by the authors while acknowledging self-reflexivity. However, the IBPA principle of time and space, which analyses the dynamics of policy inequalities over time and across historical and geographical spaces, was not utilized in the study. This is because the research focussed on the gendered COVID-19 policy dynamics at a specified time and place.

**INTERSECTIONALITY-BASED POLICY ANALYSIS FRAMEWORK**

**Guiding Principles for analysis of COVID-19 control policies & the experiences of female informal food vendors**

**REFLEXIVITY**

**POWER**

Intersecting identities

Age &Gender

Social class & education leve

Mairal status

Sexual & cultural orientation

Multi-level analysis

National - country level policies and systems

County level policies and systems

City level policies and systems

Societal and household level factors

Equity

Consideration of gender and gender equity in the policies

Social justice

Fairness to all and consideration of unique needs by the less advantaged - female informal food vendors

Diverse knowledge

Representation of women voices in policy discussion - formulation and implementation

**Figure 1: The conceptual framework for the IBPA guiding principles for analysis of COVID-19 control policies & the experiences of female informal food vendors**

4. **The discussion convincingly interprets findings but could more explicitly link empirical themes to IBPA dimensions (power, equity, social justice, etc.). In the discussion also strengthen the comparative insight by briefly noting how Kisumu’s experience relates to other secondary cities.**

We appreciate this valuable feedback. We have revised the discussion to explicitly link the themes to the IBPA principles the guided our interpretation of the findings and linked the experiences of Kisumu City to other secondary cities. The discussion now reads as follows:

This study aimed to explore the sociocultural and gender gaps in the formulation and implementation of the COVID-19 pandemic control policies and the experiences of women informal food vendors in Kisumu City, Kenya. The study adopted the IBPA fragment to reveal the prevailing gaps within the policies and to incorporate and intersectionality lens in the assessment of the policies and the lived experience narratives from the vendors and key informants. Seven key IBPA principles of power, intersecting identities, multilevel analysis, equity, social justice, multiple knowledges, and reflexivity guided our critical analysis of the data – the policy document and the narratives from the study participants.

Our analysis of the data uncovered that women informal food vendors in Kisumu City were not engaged in the formulation and implementation of the COVID-19 policies. Women vendor narratives alluded to this exclusion, while using the term ‘forced on us’ to show non-participation and lack of prior information of COVID-19 policy processes. This contravenes the provision of the Constitution of Kenya Article 174 (c) which gives powers of self-governance to the people to enhance their participation in making decisions of issues affecting them (Kenya., 2013). Previous research has shown that political empowerment of women and their participation in COVID-19 policy processes produced better gendered outcomes in the health and economic spheres (Abras et al., 2021; Huang & Lin, 2022). However, in the case of Kisumu City and many other cities in the Global South, policy formulation during the pandemic was undertaken at the national government through the Ministry of Health and in collaboration with technical experts and government officials. From our qualitative research, the policy texts did not allude to any community engagement mechanisms and gender considerations. Neither was there any kind of feedback channels provided for the public, and especially women, specifically those in informal food vending.

We identified several key barriers to the limited engagement of women in COVID-19 policy process in Kisumu, such as time limitations and the urgency of response to the crisis, unavailability of information on the need to participate, and the use of technocrats specialized in health, governance and policy matters. Our findings align with previous work by Martínez and Young (2022), who noted that the use of technical expertise in policy development by governments limited the participation of street vendors in decision-making. Another barrier to participation was the societal culture of patriarchy that impeded women participation in politics and key decision-making roles. Although Kenya’s constitution stipulates an affirmative action and creates the position of County Women Representatives, men often dominate the policy discussion tables and community level leadership (Kenyatta, 2023). These structural barriers have consistently fostered women exclusion and gender hierarchies in policy formulation and decision-making both at different levels of government and at societal level (Kenyatta, 2023). It is worth noting that this exclusion affected the responsiveness of the policies to the lived realities of women informal food vendors, worsening the existing social and economic inequalities (Women. & Work., 2023).

Furthermore, our study findings indicate that power dynamics influenced the enforcement of the COVID-19 policies. In that regard, women vendors expressed their experiences with assault, harassment and extortion by law enforcement officers. Similarly, (Solymári et al., 2022) indicated that the brutality that came with COVID-19 policy enforcement in Kenyan slum dwellings infringed on fundamental human rights. This comes against the backdrop of another study conducted in Kisumu City, where outcomes indicated that the county enforcement team was male-dominated, contributing to a biased enforcement of regulations (Loo et al., 2021). The male-dominance in enforcement predisposes women to violence and physical abuse at their places of business, which is a health and safety risk.

Similarly, in Nairobi, Wandera and colleagues (2023) indicated that women informal workers faced physical abuse from police and municipal officers during curfew implementation. This shows a commonality of the COVID-19 policy experiences by women vendors in major cities and secondary cities. This exposure to harassment and assault curtailed women’s agency in undertaking urban household food provision and fending for their families during a pandemic. Our study also provides evidence of the lack of an intersectional approach in the design of the COVID-19 policies. As in the case of the City of Cali, Colombia (Martínez & Young, 2022), informal traders in Kisumu are a heterogeneous group, of different genders, ages, socio-economic status, cultural backgrounds, religion, marital status, abilities, sexual orientation, among others. However, the policies employed a blanket policy approach and failed to consider the different settings and intersecting social identities of the vendors. This was found by Dutta et al. (Dutta et al., 2021) in India, where COVID-19 lockdown policies adopted a one-size-fits-all approach and overlooked existing gender differences.

Other findings suggest that there is need to enhance social protection mechanisms for women in the informal sector to safeguard them from gender-related exploitations (Wandera et al., 2023). The use of excessive force to enforce policies reveal how structures of power impacted the gendered experiences of women informal food vendors. Our research therefore corroborates the findings of Kiaka and colleagues (Kiaka et al., 2021) who highlighted that the COVID-19 pandemic intensified bribery and harassment in cities in the Global South.

The overlapping social locations of the women food vendors in our study highlight the diversity in ways that our study participants experienced the pandemic. The policy interventions did not consider the contextual factors such as the prevailing socio-economic situation in secondary cities in LMICs, and the pre-existing inequalities faced by women informal workers such as the issues of patriarchy and gender inequality. Women vendors were already facing compounded challenges, including the precarity of their businesses and work, gender-based violence, discrimination, economic marginalization, and sexual harassment before the occurrence COVID-19 pandemic. The lack of gendered considerations in the policies worsened the prevailing inequalities creating an additional burden to the women vendors and influencing their resilience post-pandemic.

Women faced societal expectations to take up extra caregiving roles at the expense of paid work. Notably, findings from this study raise key questions regarding the value of unpaid household and caregiving duties undertaken by women. According to the United Nation’s Development Program-UN Women COVID-19 Global Gender Response Tracker, fewer than a third of the COVID-19 policy measures implemented in Kenya applied a gender perspective, with none accounting for unpaid care work (UN Women, 2021, 2022). Our findings extend this work by uncovering the experiences of women to the gender gaps in policies in localized settings of secondary cities.

This study established that governance structures in policy formulation and implementation continued to worsen the existing gender inequalities during and post pandemic. The policies were formulated at the national government level and implemented at the county and city levels. This did not take into account the lived realities that existed in the local level of governance. In the context of IBPA principle of equity, this scenario reveals how power hierarchies shape policy decision making. We noted that women were organized into groups at the market level for collective bargaining, psychosocial and mental wellbeing, and networks for financial support. However, the representatives of these women groups were not engaged in policy processes. In their own words, the women vendors felt that the policies were “forced” on them, and they incurred additional costs in complying while also experiencing reduced business profitability. The cost of purchasing masks and sanitizers, cost of licensing and permits, compounded with the punitive penalties and fines for non-compliance further strained the women vendors economically.

Additionally, the COVID-19 policy measures added to the long list of regulations that the traders needed to comply with, and this had the possibility to cause “compliance fatigue” among the vendors, considering the strict enforcement and use of force by the enforcement officers. This is congruent to the findings of Martínez and Young (2022) that revealed that the exclusion of vulnerable groups in policy formulation and implementation hinders the effectiveness of policies and exacerbates marginalization. This evidence could benefit future policy decisions regarding the meaningful engagement of all stakeholders for inclusive and more equitable policy options, particularly during emergencies. As Cattapan et al., (2020) states, collaborative policy development should be guided by the principle of “nothing for us without us”, which fosters the meaningful participation of vulnerable groups in policy design and implementation.

5. **The limitations section is honest, but you might add a note on potential recall bias mitigation strategies (e.g., triangulation). Or maybe invert that last paragraph of the paper into areas of further research rather than being too honest with limitations, which might dilute the essence of this paper.**

We have revised the last paragraphs of the discussion section, which now reads as follows:

Additionally, the COVID-19 policy measures added to the long list of regulations that the traders needed to comply with, and this had the possibility to cause “compliance fatigue” among the vendors, considering the strict enforcement and use of force by the enforcement officers. This is congruent to the findings of Martínez and Young (2022) that revealed that the exclusion of vulnerable groups in policy formulation and implementation hinders the effectiveness of policies and exacerbates marginalization. This evidence could benefit future policy decisions regarding the meaningful engagement of all stakeholders for inclusive and more equitable policy options, particularly during emergencies. As Cattapan et al., (2020) states, collaborative policy development should be guided by the principle of “nothing for us without us”, which fosters the meaningful participation of vulnerable groups in policy design and implementation.

This study is not without limitations and it is important to recognize its limits. The choice of a cross-sectional study design meant that data was collected at a specific point in time, and in this case, between July 10, 2024 and October 15, 2024. The period during which data was collected was a few years after the COVID-19 pandemic, hence participants’ recollections could have omitted important details that could enrich the study findings. Additionally, this study only focused on one county out of the 47 counties in Kenya, and the outcome may not be generalized to the rest of the counties. However, the findings may be transferable to cities with similar characteristic like Kisumu City. Additionally, this study focused specifically on the informal food economy limiting the generalizability of the findings to other sectors of the informal economy. Lastly, the study relied on self reported data, which may be prone to social desirability, especially with reference to personalized experiences of the pandemic control measures, the impacts of the pandemic control measures on women-led informal food businesses, and the social and gender gaps in policies. Previous studies have adopted similar approaches where participants have shared their lived experiences with the COVID-19 control measures. Our findings are therefore comparable with those of past studies and this study makes important contributions to research on gendered dynamics of the informal food sector in secondary cities of the Global South.

6. **Include a separate conclusion section that explicitly summarizes the paper’s unique contributions to theory, methodology, and practice. The authors should clearly state how the study advances the application of the Intersectionality-Based Policy Analysis (IBPA) framework in African urban contexts, contributes to gender-responsive policy discourse in secondary cities, and provides actionable insights for inclusive pandemic and post-crisis policymaking**

Thanks for this excellent suggestion. We have separated and revised the conclusion section which now reads as follow:

**Conclusions**

In conclusion, this study revealed that the pandemic control measures were formulated and implemented following a top-down approach and that women informal food vendors who were directly impacted by the measures were not engaged in any of the processes. As such, the policies neither considered nor incorporated the unique needs and the diverse intersecting identities reflected within the informal food vendors’ population in secondary cities. Power imbalance and gender related vulnerabilities were reinforced and exacerbated with the control measures such as curfews, stay home orders, and social distancing regulations. While we acknowledge that the pandemic was a relatively new and urgent event that required immediate action through enforcement of public health orders inform of the control measures, we argue balancing of urgency with inclusive and equitable engagement of women by governments in public policy decision-making in times of emergencies is necessary to minimize the unintended impacts. This would enhances the gender responsiveness of the interventions and minimizes inequalities (Wenham et al., 2020). Policy interventions should be developed with the involvement of marginalized groups to account for the existing diversities and respond to their unique needs. In the case of women informal food vendors, future emergency policies should be designed guided by a gendered lens to recognize their unique roles and needs, and ensure they continue to play the vital role of urban household food security.

7. **The paper also need a final proof reading after revision. consider for example some details on Ethics not added on…pg….291 – 294 [Ethics approval for the study was granted by 290 the University of ...... Research Ethics Review Board (Protocol: Pro00138854) and ….a Research Ethics Board (ESRC P1670/2024). We also obtained written consent from all the participants engaged in the study. All participants received an honorarium of CAD $10 (KES 1000), to honor their time and contributions to the study.**

We have proof read the paper and corrected any editorial areas. The missing information in the paper are for the purpose of blinding as was requested by the editor.

8. **Also check areas still highlighted in Yellow.**

The areas highlighted in yellow are the sections that have been edited/revised following the recommendations of the editors and the reviewers.

**Responses to Reviewer #2**

1. **Reviewer #2: There is a significant time period between when COVID policies impacted the interviewees, and when the fieldwork was conducted. How might this time difference impacted them and their recall? Has it enabled time for respondents to reflect on their exclusion from policy? Worth addressing this point and reflecting on it in the paper.**

Thanks for this great observation. We have reflected on this in the study limitations section. The section now reads as follows:

This study is not without limitations and it is important to recognize its limits. The choice of a cross-sectional study design meant that data was collected at a specific point in time, and in this case, between July 10, 2024 and October 15, 2024. The period during which data was collected was a few years after the COVID-19 pandemic, hence participants’ recollections could have omitted important details that could enrich the study findings. Additionally, this study only focused on one county out of the 47 counties in Kenya, and the outcome may not be generalized to the rest of the counties. However, the findings may be transferable to cities with similar characteristic like Kisumu City. Additionally, this study focused specifically on the informal food economy limiting the generalizability of the findings to other sectors of the informal economy. Lastly, the study relied on self reported data, which may be prone to social desirability, especially with reference to personalized experiences of the pandemic control measures, the impacts of the pandemic control measures on women-led informal food businesses, and the social and gender gaps in policies. Previous studies have adopted similar approaches where participants have shared their lived experiences with the COVID-19 control measures. Our findings are therefore comparable with those of past studies and this study makes important contributions to research on gendered dynamics of the informal food sector in secondary cities of the Global South.

2. **I like the methodology, and applaud the efforts of the author/s. The 'key informant interviews (n=20),182 (2) focus group discussions (n= 40) 10 women-only participants across four focus groups, and183 (3) in-depth interview participants (n=20, four men and 16 women)' represents a solid undertaking. Good use of quotes in the results.**

Thanks for your positive feedback. We have added details in the methods section to enhance transparency and replicability. The methods section now read as follows:

**Methods**

**Study setting**

This study was conducted in Kisumu City, located within the county of Kisumu in western Kenya. It’s proximity to the Lake Victoria, world’s second largest freshwater lake, provides economic benefits of fishing resources, trade, and transport (Opiyo et al., 2018). Kenya has a devolved system of government comprising 47 counties, which was established after the promulgation of the constitution in 2010 (Chau, 2021). In the health sector, the national government oversees policy formulation while the county governments manage the delivery of health services (Chau, 2021). The dominant economic activities carried out in Kisumu include crop and livestock farming, fisheries, trade, manufacturing, construction, and mining (County Government of Kisumu, 2020; Owuor et al., 2024). Kisumu City has an approximate population of 600,000 inhabitants (KNBS, 2020) and has experienced an influx of people from the neighboring rural areas due to its rapidly urbanizing potential (Simiyu et al., 2019). Approximately 64% of the city’s inhabitants reside within the informal settlements (UN-Habitat, 2020), which experience high levels of poverty (Opiyo et al., 2018). These informal settlements include Manyatta A, Manyatta B, Obunga, Bandani, Kaloleni, Nyalenda A, Nyalenda B and Kibos (Simiyu et al., 2019). Additionally, the informal food sector thrives within the city, with a majority of the residents relying on the sector for food provision, with formal supermarkets being utilized less frequently (Kiaka et al., 2021; Opiyo et al., 2018).

We selected Kisumu City due to its vibrant informal food sector and the overrepresentation of women vendors in the urban food space. This makes it a relevant case study area to understand the gendered implications of COVID-19-related policies in regards to secondary cities of the Global South (Opiyo et al., 2018). This study particularly focused on the markets, which are located within the informal settlements in the municipality, including Manyatta, Nyalenda, Obunga, Nyawita, and Bandani.

**Study design**

This cross-sectional study was implemented from July 10, 2024 to October 15, 2024. A cross-sectional study design was deemed appropriate as it allowed for a deeper understanding of the nuanced lived experiences of the vendors, highlighting the gendered aspects and gaps in the COVID-19 policy responses. We conducted a review of four main pandemic response policies and collected primary qualitative data from the informal food vendors in Kisumu City. The four pandemic response policies that were formulated by the national government and adopted by the county and city governments to curb the spread of COVID-19 and that were reviewed in this study include – Public Health (COVID-19 General Public Safety) (Laws of Kenya, 2020a); Public Order (State Curfew) (Laws of Kenya, 2020d); Public Health (COVID-19 Restriction of Movement of Persons and Related Measures) Rules (Laws of Kenya, 2020b); and Public Health (Prevention, Control and Suppression Of COVID-19) Rules (Laws of Kenya, 2020c). The primary data generation approaches included – Key Informant Interviews (KIIs), In-Depth Interviews (IDIs), and Focus Group Discussion (FGDs) with key informants and informal food vendors in the different informal settlements and markets in Kisumu City.

**Selection participants**

***Policy document sampling***

A total of four policy documents (*n* = 4) were purposefully selected for critical review. These included the Public Health (COVID-19 General Public Safety) (Laws of Kenya, 2020a); Public Order (State Curfew) (Laws of Kenya, 2020d); Public Health (COVID-19 Restriction of Movement of Persons and Related Measures) Rules (Laws of Kenya, 2020b); and Public Health (Prevention, Control and Suppression Of COVID-19) Rules (Laws of Kenya, 2020c). The policy documents were chosen because: (1) were related to government interventions to control and contain COVID-19 pandemic in Kisumu City, either formulated at the national or county level; (2) policies were implemented within Kisumu County; (3) policies impacted the informal food Sector. Details about the four selected COVID-19 control policies are shown below in Table 1:

**Table 1: Selected COVID-19 policy documents for review**

| **Policy document** | **Relevance to the study** |
| --- | --- |
| 1. Public Health (COVID-19 General Public Safety) Rules, 2020 (Laws of Kenya, 2020a) | Contained specific health protocols such as physical distancing, wearing of masks, sanitation procedures, COVID-19 screening and prohibition of public gatherings, which impacted business operations for informal food vendors. |
| 2. Public Order (State Curfew) Order, No. 36 of 2020 (Laws of Kenya, 2020d) | Imposition of a 7:00pm to 5:00am curfew, which affected business-operating hours. |
| 3. Public Health (COVID-19 Restriction of Movement of Persons and Related Measures) Rules, 2020 (Laws of Kenya, 2020b) | Set restriction of movement of people into and out of an infected area, curtailing the supply of goods, traders and customers into markets. |
| 4. Public Health (Prevention, Control and Suppression Of COVID-19) Rules, 2020 (Laws of Kenya, 2020c) | Outlined the powers of medical and clinical officers in relation to COVID-19 control and enforcement including search, inspection and decontamination, and closure of premises, with specified penalties for non-compliance. These led to a quarantine, decline in business profitability and closure of some premises where women vendors operated. |

***Participant sampling***

Participants for the primary data collection were selected through our connection with a local community partner – Pamoja Community Based Organisation (Pamoja CBO), a local entity that undertakes community-based initiatives in Kisumu. The organisation’s well-established networks were critical for identifying the potential participants for the study using purposive sampling and snowball sampling techniques.

A total of 80 participants were recruited between July 10, 2024 and October 15, 2024 to participate in this cross-sectional study and they were drawn from the five key markets (Manyatta, Nyalenda, Obunga, Nyawita, and Bandani) in the city. The participants sampled included female informal food vendors and some male vendors and key informants who work directly with the informal food vendors. The participants were deemed to hold the relevant knowledge and information to the study. In collaboration with Pamoja CBO, potential participants were invited to an information session through the market representatives. During the sessions, details about the study were shared to allow participants to make an informed decision about their participation. Individuals who gave a written or verbal informed consent took part in the IDIs (*n* = 20), KIIs (*n* = 20), and FGDs (*n* = 40).

The participants were distributed across the 5 key markets in the city with each market having at least 12 participants across the three data collection methods. Participants were included if they met the following criteria: (1) they had been operating food businesses in and had lived in Kisumu City for over five years; (2) were owners or operators of informal food business during and after the pandemic; (3) were willing to give informed consent to participate in the individual interviews or a group discussion. Participants who did not operate their businesses during and/or after the pandemic and those that did not give consent were excluded.

The key informants (*n* = 20) were selected across the 5 key markets with at least 1 to 2 participants from each market and others from the relevant government and city departments and community organizations. The key informants were included if they met the follow criteria: (1) they were market leaders or representatives of informal food vendors in the markets; (2) they were representatives from city government or community organizations such as the, community loaning and microfinance organizations, community health promoters and leaders; and (3) they were willing to consent to participate in a one-on-one interview with one of the research team members. Any potential key informants were excluded if they did not meet the above criteria.

**Data collection techniques**

**Policy document analysis**

The Public Health (COVID-19 General Public Safety) (Laws of Kenya, 2020a), Public Order (State Curfew) (Laws of Kenya, 2020d), Public Health (COVID-19 Restriction of Movement of Persons and Related Measures) Rules (Laws of Kenya, 2020b), and Public Health (Prevention, Control and Suppression Of COVID-19) Rules (Laws of Kenya, 2020c) were retrieved from the Kenya Law Reports portal. The review aimed to assess whether these pandemic control measures considered the diverse and unique needs of female informal food vendors. Guided by the principles of the IBPA framework, the review of these policies explored how the intersecting social locations of the women informal food vendors and other sociocultural factors were considered in the policy process. This was intended to demonstrate the gender-responsiveness of the policy interventions and identify gaps within the policies, and their implications to women-led in informal food businesses.

**Primary data collection**

Research assistants trained in qualitative research techniques and fluent in English, Swahili, and the local dialect Luo engaged in the recruitment of potential participants, and in the information session to facilitate primary data collection through face-to-face interviews. The choice of language used was determined by the participant’s preference with most key informants preferring English. Most of the IDI and FGD participants preferred Swahili and/or Luo. The interviews were audio-recorded with the consent of participants with those conducted in English transcribed verbatim and those conducted in Swahili/Luo translated in English and transcribed. The transcripts were then reviewed by the research assistance the two authors (PMO & EOO) who are fluent in all the three languages used. The reviews were done to ensure accuracy in the translation and transcription of the data.

We developed and used semi-structured interview guides to facilitate the KIIs, IDIs, and FGDs. The questions were intended to elicit discussions about specific key topics relevant to the experiences of the women vendors during and after implementation of COVID-19 pandemic policies. We explored the gendered considerations in the policies and the engagement women vendors in the formulation and implementation of such policies. We probed for the specific experiences with the implementation of the COVID-19 control measures and the direct and indirect impacts on the women-led businesses. Data collection was undertaken until saturation – the point at which no new ideas or themes were emerging from the interviews. We remained reflexive through out the research process and the research assistant kept field notes that were included in the data analysis. After every interview, the research assistants summarized the key insights from each interview and held a debrief meeting with the research team at the end of each day’s work.

***Key informant interviews*:** We conducted (*n* = 20) face-to-face interviews with representatives the County Government of Kisumu, market leaders, and women group leaders, community loaning and microfinance representatives, community health promoters and leaders. The interviews lasted approximately 30 – 60 minutes with the key informants including compliance experts, enforcement offices, revenue collectors, and municipal and city government officials. Based on the expertise of the different government and community officials, we obtained in-depth insights into the policy formulation and implementation as well as the implications of policy regulations to women informal food vendors. We also sought to gather information on the interaction of the different entities/organizations and their supports to women informal food vendors during and after the pandemic.

***In-depth interviews*** (*n* = 20) included female and some male food vendors who operated food businesses during and after the COVID-19 pandemic. The male participants were included to capture their perspectives of the experiences of female food vendor with the pandemic control measures. The recruitment was facilitated through our community partner – Pamoja CBO and in collaboration with the market leader. The market leaders invited potential participants to an information session and interested vendors gave informed consent and were invited to a face-to-face interview at a convenient place of their choice. The one-on-one interview participants were interviewed to understand the impacts of COVID-19-related policies on their business and household responsibilities, caregiving duties, interaction with customers and authority, food supply, physical and mental well-being, food security status, and the coping mechanisms they adopted. All the interviews were audio-recorded, translated, and transcribed to facilitate data analysis and interpretation.

***Focus group discussions*** (*n* = 40) include four FGDs, each comprising ten women informal food vendors who were recruited through our community partner and the women market leaders. The women market leaders organized for an information session with the potential participants and those who met the inclusion criteria and gave informed consent were invited to centralized location close to the various key markets, with most of the group discussions being conducted in the community halls close to the markets. The face-to-face group discussion facilitated a participatory space for the women vendors to reflect as a collective on the impact of COVID-19 policies on their social and economic spheres. The participants shared their experiences with the enforcement and compliance of curfews, physical-distancing, movement restrictions, market closures, and the health and safety protocols that were imposed after the implementation of the COVID-19 policies. They shared the gendered effects of the policies articulated some recommendations towards future policy reforms to limit the negative impacts of policies formulated with limited consultations.

**Data analysis**

Primary qualitative data (IDIs, KIIs, and FGDs were all audio-recorded with informed consent of the participants. All the KIIs were conducted in English and transcribed verbatim. The IDIs and FGDs were mostly facilitated in Swahili or Luo and were translated to English and transcribed for data analysis. The transcription and translation were conducted by the local research assistants who facilitated the interviews and the discussion and were reviewed by the authors PMO and EOO who are fluent in English, Swahili, and Luo. All the transcripts and the policy documents, field notes, reflexive journals, and interview summaries were imported in to NVivo QSR International, version 15 for analysis, coding and management.

***Policy document analysis***

The analysis of the four policy documents included in this study was guided by seven (7) core principles of the IBPA framework including power, equity, intersecting categories, multi-level analysis, social justice, diverse knowledges, and reflexivity (Hankivsky et al., 2014). The analysis adopted Braun and Clarke’s (Braun & Clarke, 2006) reflexive thematic analysis which involved reading and re-reading the four policy documents to familiarize with the key control measures, the objectives, the key areas of interventions for curbing the COVID-19 pandemic effects. Using both deductive (guided by IBPA framework) and inductive analysis, we developed a thematic code set allowing for pre-defined and new themes to emerge [45, 46]. The predefined themes were based on the IBPA principles of power and power dynamics, intersecting identities, social justice, equity, multi-level analysis, reflexivity, and time and space. The theme codes, transcripts, field notes, interview summaries, and the post-pandemic recovery policies were then imported and organized in NVivo QSR International, version 15, and the data code line by line. To validate the theme code sets and the coding and analysis process, an independent peer researcher performed an independent analysis and cross-checked the analysis for consistency and accuracy. This process of peer checking allowed for multiple interpretations and understanding of the experiences of women informal food vendors and the identification of gaps within the policy documents [47]. To ensure rigour and trustworthiness of the evidence, we triangulated evidence from the policy documents, the narratives from the key informants, and the informal food vendors.

***Primary data analysis***

The analysis of the primary data from the KII, IDI, and FGD were also condected inductively and deductively guided by the IBPA framework and following Braun and Clarke’s six-phase thematic analysis approach (Braun & Clarke, 2006; Hankivsky et al., 2014). Through prolonged immersion in the data by taking part in the transcription, reviewing the transcripts, and reading and re-reading of the transcripts, the research team identified the initial themes and codes. Key codes were then collated into potential themes and sub-themes, which were then refined to produce a theme code set. All the data – transcripts, field notes, interview summaries, and the theme code set were imported in NVivo QSR International Version 15 for line-by-line coding. Participant quotes and excerpts from the policies were used to convey the themes and to accurately present the participant perspectives (Tong et al., 2007). To ensure rigor, we engaged in peer checking of the theme codes and the line by line coding of the data. The evidence from the different data sources – primary data and policy documents were triangulated to enhance credibility and trustworthiness of the study findings.

**Quality & Rigor**

We adopted different approaches to achieve rigor in this study. First, we triangulated various sources of data including policy document analysis and primary sources of KIIs, FGDs and IDIs. We adopted inductive and deductive approaches to data analysis, which allowed for triangulation of the theoretical and lived experience perspectives. The use of purposive sampling technique also ensured that we selected the most suitable respondents to the study questions, who had the relevant lived experience and expertise (Johnson et al., 2020) with COVID-19 control policies, community leadership, governance, and informal food vending. We engaged local research assistants through Pamoja CBO who were part of the data collection team and engaged in peer review and peer debrief to ensure consistency in the data collection, analysis, and interpretation. Peer review and debrief was achieved through regular consultation with the researchers in the team – EOO, RG, and PMO.

**Ethical considerations**

Ethics approval for the study was granted by the University of Alberta Research Ethics Review Board (Protocol: Pro00138854) and Amref Research Ethics Board (ESRC P1670/2024). We also obtained written consent from all the participants engaged in the study. All participants received an honorarium of CAD $10 (KES 1,000), to honor their time and contributions to the study.

5. **What are some policy implications of this study? Covid policy is rightly critiqued in the paper, but the author/s could be more specific about policy recommendations of their own. are their frameworks to ensure better participation and inclusion in policy in the future? Are there action steps a governing body should be sensitive to make sure women informal vendors are included? Not just for the next emergency, but in general.**

Thanks for this valuable feedback. We have drawn some key conclusions from the study evidence and give some policy recommendations for consideration beyond the COVID-19 pandemic. The conclusion section now reads as follows:

**Conclusions**

In conclusion, this study revealed that the pandemic control measures were formulated and implemented following a top-down approach and that women informal food vendors who were directly impacted by the measures were not engaged in any of the processes. As such, the policies neither considered nor incorporated the unique needs and the diverse intersecting identities reflected within the informal food vendors’ population in secondary cities. Power imbalance and gender related vulnerabilities were reinforced and exacerbated with the control measures such as curfews, stay home orders, and social distancing regulations. While we acknowledge that the pandemic was a relatively new and urgent event that required immediate action through enforcement of public health orders inform of the control measures, we argue balancing of urgency with inclusive and equitable engagement of women by governments in public policy decision-making in times of emergencies is necessary to minimize the unintended impacts. This would enhances the gender responsiveness of the interventions and minimizes inequalities (Wenham et al., 2020). Policy interventions should be developed with the involvement of marginalized groups to account for the existing diversities and respond to their unique needs. In the case of women informal food vendors, future emergency policies should be designed guided by a gendered lens to recognize their unique roles and needs, and ensure they continue to play the vital role of urban household food security.

6. **I think the paper could have a little more theoretical engagement. COVID policy in Africa was harmful to informal actors. It 'justified' slum clearances and opened the door for authoritarian intervention. What this work shows is that COVID policy also reveals something about social and political life for female informal actors. These are lessons and insights beyond the pandemic that your scholarship can help reveal. I recommend reading and engaging with the following papers;**

   Cobbinah, P.B., 2022. Enabling urban planning action in Africa: The praxis and oddity of COVID-19 pandemic response. Journal of planning literature, 37(1), pp.83-87.

   Finn, B.M. and Kobayashi, L.C., 2020. Structural inequality in the time of COVID-19: Urbanization, segregation, and pandemic control in sub-Saharan Africa. Dialogues in Human Geography, 10(2), pp.217-220.

   Finn, B.M., 2023. Pandemic urbanization: How South Africa’s history of labor and disease control creates its current disparities. Journal of Urban Affairs, 45(3), pp.616-629.

   Sharifi, A. and Khavarian-Garmsir, A.R., 2020. The COVID-19 pandemic: Impacts on cities and major lessons for urban planning, design, and management. Science of the total environment, 749, p.142391.

Thanks for the recommendations. We have read an integrated the key findings in the papers into our paper.

6. PLOS authors have the option to publish the peer review history of their article ([what does this mean?](https://track.editorialmanager.com/CL0/https:%2F%2Fjournals.plos.org%2Fplosone%2Fs%2Feditorial-and-peer-review-process%23loc-peer-review-history/1/010f019b43f817ea-9d18d8dd-247e-4089-9d3a-4a384c351f9b-000000/N5dDMQgGKjV8pfZk0Tyc2yEZ2hHkh7OPZUElryZhWAA=242)). If published, this will include your full peer review and any attached files.

If you choose “no”, your identity will remain anonymous but your review may still be made public.

**Do you want your identity to be public for this peer review?** For information about this choice, including consent withdrawal, please see our [Privacy Policy](https://track.editorialmanager.com/CL0/https:%2F%2Fwww.plos.org%2Fprivacy-policy/1/010f019b43f817ea-9d18d8dd-247e-4089-9d3a-4a384c351f9b-000000/Jc3sCNPZLZlTDLXSLRlon_dYRvIANnO49XpDBnV0MgE=242).

Reviewer #1: **Yes:** Dr Johannes Bhanye

Reviewer #2: **Yes:** Brandon Marc Finn
