# Supplementary material for: An intersectionality-based policy analysis of COVID-19 measures and women informal food vendors in Kisumu, Kenya
Source: PLoS One. 2026 Oct 1;21(10):e0359620. doi: 10.1371/journal.pone.0359620 (PMC13630232; doi:10.1371/journal.pone.0359620)
Supplement: Supplementary file 2 [file pone.0359620.s003.docx]

The revised manuscript demonstrates substantial improvement in both scientific quality and presentation.

- The Introduction has been significantly strengthened, providing a clearer contextualization of COVID-19, gender inequalities, and the importance of secondary cities.
- The expanded description of the Intersectionality-Based Policy Analysis (IBPA) framework, together with the inclusion of its guiding principles, considerably improves the conceptual foundation of the study.
- The Methods section has also been enhanced through clearer descriptions of participant recruitment, data collection, analytical procedures, and ethical considerations.
- In addition, the Discussion now provides a more analytical interpretation of the findings by explicitly linking them to issues of power relations, structural inequalities, intersecting identities, and gender-responsive governance.

Despite these important improvements, several minor issues should still be addressed before the manuscript is suitable for publication.

1. Explicitly state the manuscript's contribution

Although the conclusion has been revised, the manuscript would benefit from a more explicit statement of its contributions. Rather than leaving these contributions implicit, consider clearly distinguishing the:

- theoretical contribution (advancing intersectionality-informed policy analysis in informal urban economies);
- methodological contribution (application of the IBPA framework integrated with qualitative inquiry);
- policy contribution (implications for inclusive urban governance and emergency policy development).

The authors should add a concise paragraph summarizing these three contributions in order to strengthen the manuscript's overall impact.

2. Clarify the research objective

- The Introduction has been substantially improved; however, it still concludes primarily with the rationale for the study rather than a clearly formulated research objective.
- The authors should add at the end of the Introduction one explicit sentence beginning, for example:
  - "This study aimed to...” or "The objective of this study was..."
  - This would improve the overall structure and clarity of the manuscript.

3. Strengthen the operational policy recommendations

- The revised Discussion and Conclusion now contain stronger policy implications. The policy implications have been substantially strengthened and are considerably more actionable than in the previous version.
- Nevertheless, a few recommendations remain relatively broad and could be further enhanced by briefly illustrating how the proposed governance measures could be operationalized in practice:
  - “… governments should adopt gender-responsive emergency governance measures that intentionally solicit and integrate the perspectives and lived realities of informal vendors." (lines 817-819). This recommendation is appropriate, but it remains relatively general. The manuscript could briefly indicate **how** governments could operationalize this recommendation (e.g., formal representation of women vendors on emergency advisory committees, mandatory stakeholder consultations before implementing market restrictions, or participatory policy forums).
  - "…. governments should institutionalize mechanisms for the meaningful participation of informal vendors in policy design through recognized market associations and community-based collectives." (lines 819-821). This is a stronger recommendation than in the previous version and directly addresses one of the reviewer's concerns. However, it could be strengthened further by briefly indicating **what these mechanisms might involve**, for example permanent consultative committees, representation within municipal emergency response structures, or structured feedback mechanisms during public health emergencies.
  - "To ensure efficacy, policy interventions must be designed in collaboration with marginalized groups to accommodate social diversity and respond to localized needs." (Lines 822-823). Although this recommendation is well justified, it remains somewhat conceptual. Providing one or two examples of concrete policy instruments (e.g., targeted financial assistance, flexible licensing arrangements, emergency income protection, or tailored communication strategies) would enhance its practical value for policymakers.
- Where appropriate, consider providing more concrete examples of how governments or local authorities could implement gender-responsive and intersectionality-informed policies (e.g., stakeholder consultation mechanisms, representation of women vendors in emergency planning, targeted support programmes, or inclusive communication strategies).

The Conclusion is much stronger than in the previous version.

- However, it could be made even more concise by focusing primarily on the principal findings, the study's contribution, and future research directions, while limiting discussion-style interpretation.

Although the Discussion is now more analytical, some repetition of empirical findings remains, particularly regarding exclusion from policy-making, enforcement-related harassment, and the blanket nature of COVID-19 policies.

- The authors should consider reducing descriptive repetition and using the Discussion primarily to interpret these findings through the IBPA framework:
  - The Results already present the issue of extortion/harassment: “The overlapping policy interventions created an avenue for extortion and harassment of the women vendors:” (lines 649-650).
  - The Discussion later repeats the same empirical finding: “Women vendors reported instances of inequitable treatment, including assault, harassment, and extortion by law enforcement officers.” (Lines 719-720). Suggestion: the Discussion could reduce the descriptive repetition and focus directly on interpretation: “*These enforcement practices illustrate how gendered power relations shaped the implementation of COVID-19 measures in informal urban economies*.”

The manuscript would benefit from one final careful language edit (for example, "venders" instead of "vendors" in the Abstract).
