# Supplementary material for: An intersectionality-based policy analysis of COVID-19 measures and women informal food vendors in Kisumu, Kenya
Source: PLoS One. 2026 Oct 1;21(10):e0359620. doi: 10.1371/journal.pone.0359620 (PMC13630232; doi:10.1371/journal.pone.0359620)
Supplement: Supplementary file 3 [file pone.0359620.s004.docx]

**Response to Editor and reviewers’ comments**

**NB: Highlighted in yellow are the edited/revised sections in the paper.**

**NB: Highlighted in blue are the editor/reviewers comments and suggested edits**

**NB: Highlighted in gray are our responses to the suggestions/comments**

Thank you for submitting your manuscript to PLOS ONE. After careful consideration, we feel that it has merit but does not fully meet PLOS ONE’s publication criteria as it currently stands. Therefore, we invite you to submit a revised version of the manuscript that addresses the points raised during the review process.

**Well noted and addressed as shown below!**

**Reviewers' comments:
Reviewer #1: Reviewer’s Comments**
Overall, the revision shows clear and substantial improvement. Several important issues raised during the previous review have been addressed. In particular:

- The Introduction has been expanded and now provides a broader contextualization of COVID-19 impacts, gender inequalities, and the relevance of secondary cities.
- The manuscript now includes a conceptual illustration of the IBPA framework, which improves the clarity of the analytical approach.
- The Methods section has been strengthened, with clearer explanations of participant recruitment, data collection procedures, and analytical methods.
- Additional information regarding ethical approval and research rigor has been provided.
- The Discussion section has been improved and more explicitly connects the findings with IBPA principles such as power relations, equity, and intersecting identities.
- A distinct Conclusion section has been included, which summarizes the main findings and highlights the policy relevance of the study.

Despite these improvements, several points still require further attention before the manuscript can be considered for publication.

**Thank you for acknowledging the improvements made in the paper following the incorporation of the 1^st^ round of reviewers’ comments. We appreciate your contribution to the work.**

**Conceptual positioning of IBPA**

• Although the manuscript explains the principles of Intersectionality-Based Policy Analysis (IBPA), it still does not clearly articulate how IBPA provides additional explanatory value compared with other gender or policy analysis frameworks. A clearer justification of the analytical advantage of IBPA would strengthen the theoretical contribution of the study.

**Thank you for this comment. We have extensively revised the introduction section to detail the application, relevance, and the rationale for the use of the IBPA framework in this research. A section of the introduction section now reads as follows:**

……………

This study extends our understanding of the extent to which the perspectives of women business-owners were considered in the formulation and implementation of the pandemic control policies in secondary cities in the Global South. Understanding the gendered dimensions of the COVID-19 policies on women engaged in informal food businesses is essential for advocating for equitable policy frameworks that safeguard the food and nutritional security and economic stability of urban households, particularly, in times of emergencies. The study adopted the intersectionality-based policy analysis (IBPA) framework (Hankivsky et al., 2014) to investigate the influence of power dynamics and systemic structures on the marginalization of women food vendors within secondary cities during the COVID-19 pandemic. The IBPA framework facilitated an analysis that extended beyond gender as an individual level factor, enabling an exploration of how intersecting identities, policies and regulations – such as the COVID-19 control measures construct social locations and inequities marginalize specific groups, such as the women food vendors.

We utilized seven of the eight key principles of the IBPA framework – the principles of equity, power, intersecting categories, multi-level analysis, reflexivity, social justice, and diverse knowledges (Hankivsky et al., 2014). These seven IBPA framework principles (See Figure 1 below) guided the analysis of the COVID-19-related policies, key informant interviews, focus group discussions and in-depth interview data as well as the interpretation of the findings.

The principle of intersecting categories was employed to examine the interactions between various overlapping social identities – including gender, age, sexual orientation, marital status, educational attainment, cultural orientation, and socio-economic standing—and pandemic-related control strategies in intensifying the marginalization of female vendors. Furthermore, the analysis assessed whether these policies acknowledged the heterogeneous identities of informal food vendors and the specific requirements arising from such positionalities. By applying an intersectional perspective to policy review and qualitative narrative analysis, this study identified the ways in which social locations influenced women’s experiences of the pandemic policies and how inequalities predicated upon the design, dissemination, and interpretation of (Kanenberg & Leal, 2019).

In integrating the principle of power and multi-level analysis, we sought to highlight how overlapping power either facilitated inclusion or exacerbated systemic inequities for female vendors within the informal sector. In the Kenyan context—where healthcare service delivery is devolved to county and municipal authorities while legislative mandate remains centralized federal mandate, complex hierarchies of power emerge. Within such complex governance environments, the management of a public health crisis such as the COVID-19 pandemic necessitated a multisectoral approach characterized by deliberate engagement of the diverse partners, particularly the disadvantaged populations – such as the informal food vendors.

Furthermore, our analytical framework integrated the gender equity lens and the principle of social justice to examine the extent of gender inclusivity with the COVID-19 control measures, while considering the overlapping layers of power in the policy processes. By applying the principle of diverse knowledge, the framework enabled us to interrogate whether the voices of women in informal food vending were integrated in policy development and how power structures influenced the inclusion or exclusion of their diverse perspectives in policy decision making. Whilst studies in other jurisdictions indicated that governments neglected the gender-specific requirements of women during COVID-19 policy responses (Chitando, 2020), the application of the IBPA framework in this study enabled the advancement of the analysis beyond gender to also consider other intersecting identities, tenets of power dynamics, promotion of a social justice lens, and appreciation of diverse knowledge in policy analysis. We applied the principles while acknowledging the relevance of being self-reflexive. Notably, the IBPA principle of time and space, which analyses the dynamics of policy inequalities over time and across historical and geographical spaces, was excluded from this study. This is because the research focussed on the gendered COVID-19 policy dynamics at a specified time and place.

**Explicit statement of the manuscript’s contribution**

• The conclusion would benefit from a clearer articulation of the manuscript’s contribution to theory, methodology, and policy practice. Currently, these contributions remain somewhat implicit:

o The authors are encouraged to explicitly state:

o the theoretical contribution, particularly how the study advances intersectionality-informed policy analysis in the context of urban informal economies;

o the methodological contribution, namely the application of the Intersectionality-Based Policy Analysis (IBPA) framework combined with qualitative methods to examine emergency governance measures;

o the policy contribution, including how the findings inform more inclusive urban governance and crisis-response policies affecting women informal food vendors.

**This has been addressed and integrated in the conclusion section which now includes specific policy interventions based on the discussed results.**

Policy implications

• The discussion and conclusion provide useful general recommendations regarding inclusive policymaking and engagement with women vendors. However, the policy implications remain relatively broad. The manuscript would benefit from more concrete and operational recommendations that could inform local governance or urban policy design.

**This has been addressed and integrated in the discussion and conclusion sections which now includes specific policy interventions based on the discussed results.**

o “Policy interventions should be developed with the involvement of marginalized groups to account for the existing diversities and respond to their unique needs.”. Suggestion: “The findings demonstrate the importance of integrating intersectionality-informed approaches into urban policy design to ensure that crisis response measures do not disproportionately marginalize women working in informal food systems.”

**This has been addressed and integrated in the discussion and conclusion sections which now includes specific policy interventions based on the discussed results.**

Language and editorial issues

• While the manuscript has improved stylistically, several minor grammatical and wording issues remain and should be corrected during the next revision.

o The term “IBPA fragment” is incorrect and unclear.

**Thank you! Corrected!**

o “The analysis of the primary data from the KII, IDI, and FGD were also condected inductively and deductively guided by the IBPA..”

**Thank you! Corrected!**

o “This would enhance the gender responsiveness of the interventions and minimizes inequalities…”

**Thank you! Corrected!**

• A careful proofreading is recommended to ensure clarity and consistency.

**Thank you! We have proof read and edited the entire manuscript for clarity!**

In addition, the authors should consider also the following recommendations:

• The introduction should end with clear research objectives or research questions.

**The introduction section has been extensively revised and a section that reflects the research question now reads as follows:**

Kisumu City is the economic hub for western Kenya and the city’s economy is largely driven by informal trade (Opiyo et al., 2018). Over 60% of the informal businesses in the city are female owned, the majority of whom reside in informal settlements and belong to the Luo or Luhya speaking communities (Kiplagat et al., 2025). In these communities, gender roles are strictly delineated, with domestic responsibilities and caregiving traditionally assigned to women and girls, whose social standing is often diminished by prevailing patriarchal systems. The implementation of pandemic containment measures disproportionately affected these women-led businesses, as the closure of educational institutions and the increased need for medical care for family members imposed substantial additional caregiving burdens (Kiplagat et al., 2025; Odhiambo et al., 2025). Additionally, systemic power imbalances and the predominance of men in leadership roles resulted in the marginalization of female perspectives, particularly those from the informal food sector, in the formulation of pandemic response strategies.

This study extends our understanding of the extent to which the perspectives of women business-owners were considered in the formulation and implementation of the pandemic control policies in secondary cities in the Global South. Understanding the gendered dimensions of the COVID-19 policies on women engaged in informal food businesses is essential for advocating for equitable policy frameworks that safeguard the food and nutritional security and economic stability of urban households, particularly, in times of emergencies. The study adopted the intersectionality-based policy analysis (IBPA) framework (Hankivsky et al., 2014) to investigate the influence of power dynamics and systemic structures on the marginalization of women food vendors within secondary cities during the COVID-19 pandemic. The IBPA framework facilitated an analysis that extended beyond gender as an individual level factor, enabling an exploration of how intersecting identities, policies and regulations – such as the COVID-19 control measures construct social locations and inequities marginalize specific groups, such as the women food vendors.

• In several sections, the Discussion reiterates findings already presented in the Results (e.g., the exclusion of women vendors from policy decision-making, experiences of harassment by enforcement officers, governance structure, and the blanket nature of COVID-19 policies):

o Results section: “Women vendors narrated their lived experiences of unfair treatment, extortion, assault and coercion from the enforcement authorities during the pandemic.” Discussion section: “In that regard, women vendors expressed their experiences with assault, harassment and extortion by law enforcement officers.”

**The discussion and the results section have been harmonized with the findings in the results section and the discussion having a more critical and analytical interpretation of the result/findings. See how the discussion now reads below.**

o The Discussion would benefit from stronger analytical interpretation linking these findings to the IBPA framework, particularly regarding power relations, intersecting identities, and structural inequalities in urban governance!

We have extensively revised the discussion section and incorporated the suggested changes. The discussion now reads as follows:

**Discussion**

This study aimed to examine the sociocultural and gender disparities inherent in the formulation and implementation of COVID-19 mitigation policies and the subsequent experiences of women within the informal food vending sector. The findings reveal that the formulation and execution of these pandemic mitigation measures were characterized by a top-down approach that limited the engagement of women food vendors. Furthermore, the policies were fragmented by varying levels of power and power dynamics, reinforced prevailing inequities, and neglected the diverse intersecting identities of informal food vendors. Women vendors were systematically excluded from policy-making processes due to existing governance structures wherein health policies are formulated at the national level while service delivery is delegated to county governments, with most of these roles being male dominated. At the city and community levels, women vendors possess limited power and influence; they are frequently excluded from key decision-making processes due to systemic barriers that reinforce power hierarchies and shape policy outcomes. Our research identified several critical barriers contributing to the restricted engagement of women in the COVID-19 policy-making process within Kisumu. The factors included time constraints necessitated by the urgency of the crisis, sociocultural and gender inequities, a lack of accessible information regarding participation opportunities, and a reliance on technocratic expertise within the fields of health, governance, and policy. Such findings correspond with the observations of Martínez and Young (2022), who argued that the prioritization of technical experts in policy development by governments limited the participation of street vendors in decision-making processes. Furthermore, the pervasive societal culture of patriarchy served as a significant impediment to women involvement in political and leadership roles. Despite constitutional provisions for affirmative action and the establishment of County Women Representative positions in Kenya, policy discussions and community leadership remain predominantly male-dominated. These structural barriers reinforce gender hierarchies and the exclusion of women from decision-making at multiple levels of governance and society. Consequently, this lack of representation diminished the responsiveness of policies to the specific circumstances of women in the informal food vending sector, thereby exacerbating existing socio-economic disparities. (Coalition on Violence Against Women & Making It Work, 2023).

Narratives from the vendors emphasized this systematic exclusion, with participants frequently employing the expression "forced on us" to describe their marginalization and the lack of prior consultation regarding policy initiatives. These exclusionary practices appear to deviate from Article 174 (c) f the Constitution of Kenya, which mandates the enhancement of public participation in decision-making processes affecting the citizens (Laws of Kenya, 2013). Although scholarly evidence indicates that the political empowerment of women in pandemic-related policy-making lead to improved gender-sensitive health and economic outcomes (Abras et al., 2021; Huang & Lin, 2022), the approach in Kisumu City and many other cities in the Global South such as Rangpur in Bangladesh and Rusizi and Rubavu in Rwanda, remained highly centralized within the national Ministry of Health. The development and implementation of mitigation measures were restricted to technical experts and government officials, failing to incorporate community engagement strategies, gender-based considerations, or formal feedback mechanisms for the public, particularly for women operating in the informal food economy.

Furthermore, our study findings indicate that these policies reinforced existing inequities by failing to account for the diverse intersecting identities of marginalized groups, such as women food vendors in secondary cities. The policies did not contextualize the lived realities of these women with reference to their intersecting identities or prevailing sociocultural practices. Women vendors reported instances of inequitable treatment, including assault, harassment, and extortion by law enforcement officers. Similarly, (Solymári et al., 2022) observed that the brutality associated with COVID-19 policy enforcement in Kenyan informal settlements infringed upon fundamental human rights. This occurred against the backdrop of a male-dominated county enforcement team in Kisumu, which contributed to biased regulation enforcement (Loo et al., 2021). Such male dominance in enforcement predisposes women to violence and physical abuse at their places of business, posing severe health and safety risks and curtailing their agency to support their families. These experiences of harassment and the application of excessive force demonstrate how established power structures directly impacted the gendered experiences of women in the informal food sector, with some vendors reporting being compelled to pay bribes of up to five thousand shillings to avoid arrest.

Likewise, research in Nairobi by Wandera et al. (2023) documented that women in the informal workforce were subjected to physical abuse by police and municipal officers during the enforcement of curfews. This indicates a consistent pattern of COVID-19 policy impacts on women vendors across both primary and secondary urban centers.  These findings are corroborated by Kiaka and colleagues who emphasized that the pandemic exacerbated instances of bribery and harassment in the Global South, particularly affecting informal vendors whose survival necessitated significant resilience. Exposure to such harassment and assault restricted women’s agency to facilitate urban household food provision and sustain their families during the pandemic. Consequently, it is argued that during periods of emergency, governments must implement gender-responsive governance systems to facilitate the engagement of women in the informal sector and ensure that policies address the specific requirements and experiences of this marginalized yet essential segment of the urban population.

Our study further provides evidence regarding the absence of an intersectional framework in the formulation of COVID-19 policies. Informal traders in Kisumu constitute a heterogeneous group characterized by diverse genders, ages, socio-economic statuses, cultural backgrounds, marital status, abilities, and religious affiliations, among other intersecting social identities. However, the pandemic mitigation strategies utilized a blanket policy approach that failed to account for the varied settings and specific identities of these vendors. This was also noted by Dutta et al. (2021) in India, where COVID-19 lockdown policies adopted a one-size-fits-all approach and overlooked existing gender differences.

These policy interventions did not consider the contextual factors such as the prevailing socio-economic situation in low- and middle-income countries (LMICs), and the pre-existing inequalities faced by women informal workers, including patriarchal structures and gender-based discrimination. Women vendors were already facing compounded challenges, including the precarity of their businesses and work, gender-based violence, discrimination, economic marginalization, and sexual harassment before the occurrence COVID-19 pandemic. The lack of gendered considerations in the policies worsened the prevailing inequalities creating an additional burden to the women vendors and influencing their resilience post-pandemic. While we acknowledge that the pandemic was a relatively new and urgent event that required immediate action through enforcement of public health orders inform of the control measures, we argue that governments must balance such urgency with inclusive and equitable engagement of women in public policy decision-making during emergencies to mitigate unintended negative impacts.

Women faced societal expectations to take up extra caregiving roles at the expense of paid work. Notably, findings from this study raise key questions regarding the value of unpaid household and caregiving duties undertaken by women. According to the United Nation’s Development Program-UN Women COVID-19 Global Gender Response Tracker, fewer than a third of the COVID-19 policy measures implemented in Kenya applied a gender perspective, with none accounting for unpaid care work (UN Women, 2021, 2022). Our findings extend this work by uncovering the experiences of women to the gender gaps in policies in localized settings of secondary cities.

Additionally, the COVID-19 policy measures introduced a significant number of new regulations with which traders were required to comply, potentially inducing "compliance fatigue" among vendors, considering the strict enforcement and use of force by the enforcement officers. This aligns with the findings of Martínez and Young (2022), which demonstrate that the exclusion of vulnerable groups in policy formulation and implementation undermines policy effectiveness and exacerbates marginalization. Such evidence may inform future policy decisions concerning the meaningful engagement of all stakeholders to achieve inclusive and more equitable policy outcomes, particularly during emergencies. As articulated by Cattapan et al., (2020), collaborative policy development should be guided by the principle of "nothing for us without us," which promotes the substantive participation of vulnerable groups in policy design and execution.

This study is not without limitations and it is important to recognize its limits. The choice of a cross-sectional study design meant that data was collected at a specific point in time, and in this case, between July 10, 2024 and October 15, 2024. The period during which data was collected was a few years after the COVID-19 pandemic, hence participants’ recollections could have omitted important details that could enrich the study findings. Additionally, this study only focused on one county out of the 47 counties in Kenya, and the outcome may not be generalized to the rest of the counties. However, the findings may be transferable to cities with similar characteristic like Kisumu City. Additionally, this study focused specifically on the informal food economy limiting the generalizability of the findings to other sectors of the informal economy. Lastly, the study relied on self reported data, which may be prone to social desirability, especially with reference to personalized experiences of the pandemic control measures, the impacts of the pandemic control measures on women-led informal food businesses, and the social and gender gaps in policies. Previous studies have adopted similar approaches where participants have shared their lived experiences with the COVID-19 control measures. Our findings are therefore comparable with those of past studies and this study makes important contributions to research on gendered dynamics of the informal food sector in secondary cities of the Global South.

• In Conclusion section:

o The policy recommendations in the conclusion are relevant but remain rather general (e.g., the call for ‘involvement of marginalized groups’ or ‘gender-responsive policy design’). The manuscript would benefit from more concrete and actionable policy suggestions, such as specific mechanisms for engaging women informal food vendors in policy processes or examples of gender-responsive emergency governance measures.

**Thank you for this suggestion! It has been incorporated into the conclusion.**

§ “Policy interventions should be developed with the involvement of marginalized groups to account for the existing diversities and respond to their unique needs”. Suggestion: “Policy interventions should recognize the unique roles of women informal food vendors by incorporating targeted economic support measures, flexible market regulations, and inclusive communication strategies during public health emergencies.”

**Thank you for this suggestion! It has been incorporated into the conclusion**

o “The Conclusion section currently includes extended discussion and policy interpretation The authors should avoid repeating discussion and focus on key takeaways:

§ “While we acknowledge that the pandemic was a relatively new and urgent event that required immediate action through enforcement of public health orders inform of the control measures, we argue balancing of urgency with inclusive and equitable engagement of women by governments in public policy decision-making in times of emergencies is necessary to minimize the unintended impacts.” This sentence provides extended interpretation and argumentation, which is typical of the Discussion, is very long, and introduces new reasoning rather than summarizing findings. The conclusion would be stronger if it focused more concisely on summarizing the main findings and the study’s contribution, while limiting extended interpretation and policy discussion.” Suggestion: “Although the pandemic required urgent public health responses, the findings highlight the importance of inclusive policy processes that engage women informal food vendors in emergency decision-making.”

**Thank you for this suggestion! It has been incorporated into the conclusion**

o The authors should highlight future research directions:

§ “Future emergency policies as well as other government policies and interventions should be designed guided by a gendered lens to recognize their unique roles and needs.” The sentence focuses on policy recommendations, but the Conclusion does not mention directions for future research. Suggestion: “Future research could extend this analysis by conducting comparative studies across different secondary cities in order to examine how intersectional inequalities shape the experiences of informal food vendors under varying governance and policy contexts.”

**Thank you for this suggestion! This has been integrated into to the conclusion section which now reads as follows:**

**Conclusions**

In conclusion, this research possesses significant scholarly and practical relevance, offering both methodological and substantive contributions. By utilizing an IBPA framework to examine urban informal economies and the experiences of informal food vendors, the study extends policy discourse beyond a singular focus on gender. It underscores the critical role of intersecting identities in shaping the lived experiences of women vendors and facilitates the integration of intersectional policy analysis within health research.

Substantively, this research indicates that pandemic containment measures were developed and enforced utilizing a top-down methodology, which effectively marginalized female informal food vendors and failed to account for their unique needs and diverse intersecting identities. Additionally, pre-existing power dynamics and gender-based vulnerabilities were reinforced and exacerbated by the regulatory measures, including curfews, stay-at-home mandates, and physical distancing protocols.

To address this gap, policy interventions should recognize the unique roles of women informal food vendors by incorporating targeted economic support measures, flexible market regulations, and inclusive communication strategies during public health emergencies. While the global health crisis necessitated immediate public health interventions, these findings emphasize the imperative for inclusive policy-making processes that incorporate female informal food vendors into the policy formulation and implementation processes. For example, governments should adopt gender-responsive emergency governance measures that intentionally solicit and integrate the perspectives and lived realities of informal vendors. Furthermore, governments should institutionalize mechanisms for the meaningful participation of informal vendors in policy design through recognized market associations and community-based collectives. To ensure efficacy, policy interventions must be designed in collaboration with marginalized groups to accommodate social diversity and respond to localized needs. In the context of female informal food vendors, future regulatory frameworks and emergency directives should adopt a gendered perspective to formally recognize their unique contributions and safeguard their role in maintaining urban food security.

Such integration would enhance the gender responsiveness of policy interventions and mitigate structural inequalities. Future scholarly inquiries should expand upon this analysis by conducting comparative studies across various secondary cities to evaluate how intersectional inequalities shape the experiences of informal food vendors under disparate governance and policy frameworks.

**Responses to Reviewer #2**

The manuscript addresses a relevant problem and provides empirical information on the effects of COVID-19 policies on a population that has been insufficiently studied. The revised version shows a clear effort to respond to previous comments, particularly in the expansion of the introduction, the development of the IBPA framework, and the extension of the discussion.

Even so, the manuscript maintains problems related to the formulation of the study, internal consistency, and methodological clarity that should be addressed before it can be considered in its current form.

**Thank you!**

RESPONSE TO PREVIOUS COMMENTS

The expansion of the introduction and the incorporation of additional literature are considered addressed. The context is now more solid and better articulated.

The development of the IBPA framework is considered partially addressed. Its description was expanded and a figure was incorporated, but its use is not consistent throughout the manuscript.

**Thank you for this observation! The IBPA framework and its use throughout the paper has been reviewed and areas of inconsistency revised.**

…….

This study extends our understanding of the extent to which the perspectives of women business-owners were considered in the formulation and implementation of the pandemic control policies in secondary cities in the Global South. Understanding the gendered dimensions of the COVID-19 policies on women engaged in informal food businesses is essential for advocating for equitable policy frameworks that safeguard the food and nutritional security and economic stability of urban households, particularly, in times of emergencies. The study adopted the intersectionality-based policy analysis (IBPA) framework (Hankivsky et al., 2014) to investigate the influence of power dynamics and systemic structures on the marginalization of women food vendors within secondary cities during the COVID-19 pandemic. The IBPA framework facilitated an analysis that extended beyond gender as an individual level factor, enabling an exploration of how intersecting identities, policies and regulations – such as the COVID-19 control measures construct social locations and inequities marginalize specific groups, such as the women food vendors.

We utilized seven of the eight key principles of the IBPA framework – the principles of equity, power, intersecting categories, multi-level analysis, reflexivity, social justice, and diverse knowledges (Hankivsky et al., 2014). These seven IBPA framework principles (See Figure 1 below) guided the analysis of the COVID-19-related policies, key informant interviews, focus group discussions and in-depth interview data as well as the interpretation of the findings.

The principle of intersecting categories was employed to examine the interactions between various overlapping social identities – including gender, age, sexual orientation, marital status, educational attainment, cultural orientation, and socio-economic standing—and pandemic-related control strategies in intensifying the marginalization of female vendors. Furthermore, the analysis assessed whether these policies acknowledged the heterogeneous identities of informal food vendors and the specific requirements arising from such positionalities. By applying an intersectional perspective to policy review and qualitative narrative analysis, this study identified the ways in which social locations influenced women’s experiences of the pandemic policies and how inequalities predicated upon the design, dissemination, and interpretation of (Kanenberg & Leal, 2019).

The inclusion of explicit objectives or research questions was not addressed. The purpose of the study continues to be distributed across the narrative and is not presented in a direct manner in the introduction.

**Thank you pointing this out! We have revised the introduction to accurately reflect the aim/objective of the paper. A section of the Introduction now reads as follows:**

The discussion was expanded and includes more bibliographic support. However, in several points, the statements are not directly supported by the results, and therefore this aspect is considered partially addressed.

**The discussion section has been extensively revised and the suggestions integrated to ensure that the claims are supported by the presented results. The Discussion Section now reads as follows:**

**Discussion**

This study aimed to examine the sociocultural and gender disparities inherent in the formulation and implementation of COVID-19 mitigation policies and the subsequent experiences of women within the informal food vending sector. The findings reveal that the formulation and execution of these pandemic mitigation measures were characterized by a top-down approach that limited the engagement of women food vendors. Furthermore, the policies were fragmented by varying levels of power and power dynamics, reinforced prevailing inequities, and neglected the diverse intersecting identities of informal food vendors. Women vendors were systematically excluded from policy-making processes due to existing governance structures wherein health policies are formulated at the national level while service delivery is delegated to county governments, with most of these roles being male dominated. At the city and community levels, women vendors possess limited power and influence; they are frequently excluded from key decision-making processes due to systemic barriers that reinforce power hierarchies and shape policy outcomes. Our research identified several critical barriers contributing to the restricted engagement of women in the COVID-19 policy-making process within Kisumu. The factors included time constraints necessitated by the urgency of the crisis, sociocultural and gender inequities, a lack of accessible information regarding participation opportunities, and a reliance on technocratic expertise within the fields of health, governance, and policy. Such findings correspond with the observations of Martínez and Young (2022), who argued that the prioritization of technical experts in policy development by governments limited the participation of street vendors in decision-making processes. Furthermore, the pervasive societal culture of patriarchy served as a significant impediment to women involvement in political and leadership roles. Despite constitutional provisions for affirmative action and the establishment of County Women Representative positions in Kenya, policy discussions and community leadership remain predominantly male-dominated. These structural barriers reinforce gender hierarchies and the exclusion of women from decision-making at multiple levels of governance and society. Consequently, this lack of representation diminished the responsiveness of policies to the specific circumstances of women in the informal food vending sector, thereby exacerbating existing socio-economic disparities. (Coalition on Violence Against Women & Making It Work, 2023).

Narratives from the vendors emphasized this systematic exclusion, with participants frequently employing the expression "forced on us" to describe their marginalization and the lack of prior consultation regarding policy initiatives. These exclusionary practices appear to deviate from Article 174 (c) f the Constitution of Kenya, which mandates the enhancement of public participation in decision-making processes affecting the citizens (Laws of Kenya, 2013). Although scholarly evidence indicates that the political empowerment of women in pandemic-related policy-making lead to improved gender-sensitive health and economic outcomes (Abras et al., 2021; Huang & Lin, 2022), the approach in Kisumu City and many other cities in the Global South such as Rangpur in Bangladesh and Rusizi and Rubavu in Rwanda, remained highly centralized within the national Ministry of Health. The development and implementation of mitigation measures were restricted to technical experts and government officials, failing to incorporate community engagement strategies, gender-based considerations, or formal feedback mechanisms for the public, particularly for women operating in the informal food economy.

Furthermore, our study findings indicate that these policies reinforced existing inequities by failing to account for the diverse intersecting identities of marginalized groups, such as women food vendors in secondary cities. The policies did not contextualize the lived realities of these women with reference to their intersecting identities or prevailing sociocultural practices. Women vendors reported instances of inequitable treatment, including assault, harassment, and extortion by law enforcement officers. Similarly, (Solymári et al., 2022) observed that the brutality associated with COVID-19 policy enforcement in Kenyan informal settlements infringed upon fundamental human rights. This occurred against the backdrop of a male-dominated county enforcement team in Kisumu, which contributed to biased regulation enforcement (Loo et al., 2021). Such male dominance in enforcement predisposes women to violence and physical abuse at their places of business, posing severe health and safety risks and curtailing their agency to support their families. These experiences of harassment and the application of excessive force demonstrate how established power structures directly impacted the gendered experiences of women in the informal food sector, with some vendors reporting being compelled to pay bribes of up to five thousand shillings to avoid arrest.

Likewise, research in Nairobi by Wandera et al. (2023) documented that women in the informal workforce were subjected to physical abuse by police and municipal officers during the enforcement of curfews. This indicates a consistent pattern of COVID-19 policy impacts on women vendors across both primary and secondary urban centers.  These findings are corroborated by Kiaka and colleagues who emphasized that the pandemic exacerbated instances of bribery and harassment in the Global South, particularly affecting informal vendors whose survival necessitated significant resilience. Exposure to such harassment and assault restricted women’s agency to facilitate urban household food provision and sustain their families during the pandemic. Consequently, it is argued that during periods of emergency, governments must implement gender-responsive governance systems to facilitate the engagement of women in the informal sector and ensure that policies address the specific requirements and experiences of this marginalized yet essential segment of the urban population.

Our study further provides evidence regarding the absence of an intersectional framework in the formulation of COVID-19 policies. Informal traders in Kisumu constitute a heterogeneous group characterized by diverse genders, ages, socio-economic statuses, cultural backgrounds, marital status, abilities, and religious affiliations, among other intersecting social identities. However, the pandemic mitigation strategies utilized a blanket policy approach that failed to account for the varied settings and specific identities of these vendors. This was also noted by Dutta et al. (2021) in India, where COVID-19 lockdown policies adopted a one-size-fits-all approach and overlooked existing gender differences.

These policy interventions did not consider the contextual factors such as the prevailing socio-economic situation in low- and middle-income countries (LMICs), and the pre-existing inequalities faced by women informal workers, including patriarchal structures and gender-based discrimination. Women vendors were already facing compounded challenges, including the precarity of their businesses and work, gender-based violence, discrimination, economic marginalization, and sexual harassment before the occurrence COVID-19 pandemic. The lack of gendered considerations in the policies worsened the prevailing inequalities creating an additional burden to the women vendors and influencing their resilience post-pandemic. While we acknowledge that the pandemic was a relatively new and urgent event that required immediate action through enforcement of public health orders inform of the control measures, we argue that governments must balance such urgency with inclusive and equitable engagement of women in public policy decision-making during emergencies to mitigate unintended negative impacts.

Women faced societal expectations to take up extra caregiving roles at the expense of paid work. Notably, findings from this study raise key questions regarding the value of unpaid household and caregiving duties undertaken by women. According to the United Nation’s Development Program-UN Women COVID-19 Global Gender Response Tracker, fewer than a third of the COVID-19 policy measures implemented in Kenya applied a gender perspective, with none accounting for unpaid care work (UN Women, 2021, 2022). Our findings extend this work by uncovering the experiences of women to the gender gaps in policies in localized settings of secondary cities.

Additionally, the COVID-19 policy measures introduced a significant number of new regulations with which traders were required to comply, potentially inducing "compliance fatigue" among vendors, considering the strict enforcement and use of force by the enforcement officers. This aligns with the findings of Martínez and Young (2022), which demonstrate that the exclusion of vulnerable groups in policy formulation and implementation undermines policy effectiveness and exacerbates marginalization. Such evidence may inform future policy decisions concerning the meaningful engagement of all stakeholders to achieve inclusive and more equitable policy outcomes, particularly during emergencies. As articulated by Cattapan et al., (2020), collaborative policy development should be guided by the principle of "nothing for us without us," which promotes the substantive participation of vulnerable groups in policy design and execution.

This study is not without limitations and it is important to recognize its limits. The choice of a cross-sectional study design meant that data was collected at a specific point in time, and in this case, between July 10, 2024 and October 15, 2024. The period during which data was collected was a few years after the COVID-19 pandemic, hence participants’ recollections could have omitted important details that could enrich the study findings. Additionally, this study only focused on one county out of the 47 counties in Kenya, and the outcome may not be generalized to the rest of the counties. However, the findings may be transferable to cities with similar characteristic like Kisumu City. Additionally, this study focused specifically on the informal food economy limiting the generalizability of the findings to other sectors of the informal economy. Lastly, the study relied on self reported data, which may be prone to social desirability, especially with reference to personalized experiences of the pandemic control measures, the impacts of the pandemic control measures on women-led informal food businesses, and the social and gender gaps in policies. Previous studies have adopted similar approaches where participants have shared their lived experiences with the COVID-19 control measures. Our findings are therefore comparable with those of past studies and this study makes important contributions to research on gendered dynamics of the informal food sector in secondary cities of the Global South.

A limitations section was included, but the analysis of its implications is limited. This point is considered partially addressed. Editorial revision was not addressed. Formal and structural issues remain that should have been corrected at this stage.

**The paper has been reviewed for any editorial errors and for better clarity of the key messages being communicated.**

GENERAL COMMENTS

The manuscript presents relevant empirical material and addresses an important topic. However, several aspects related to methodological transparency, internal consistency, and analytical clarity need to be strengthened.

In its current form, the manuscript does not clearly define its research objective, does not consistently apply the IBPA framework, and does not provide sufficient detail to reconstruct the methodological procedure. In addition, there are structural issues in the results and discussion sections, including lack of separation between evidence and interpretation, repetition of arguments, and insufficient analytical development in some parts.

These issues do not invalidate the study, but they do require substantial revision.

**Thanks for these valuable comments. The paper has been extensively revised and the areas of limitation/weaknesses pointed addressed.**

SPECIFIC COMMENTS

TITLE

The title reflects the content of the manuscript. It includes the analytical approach, the context of COVID-19 control measures, the study population, and the empirical case.

Even so, it requires revision.

**The title has been revised and made more concise to allow for clarity. This is the new title to the manuscript:**

**An Intersectionality-Based Policy Analysis of COVID-19 Measures and Women Informal Food Vendors in Kisumu, Kenya**

First, it is a long title. The number of elements included in a single structure reduces its clarity.

Second, it brings together two analytical levels, policy analysis and lived experiences, that are both part of the manuscript, but in the title, they appear accumulated, making it heavy.

Third, the expression “Secondary Cities” in plural does not fully correspond to the empirical scope of the study, which is based on a single case. Although the manuscript seeks to situate the analysis within that type of context, what is presented is a case study in Kisumu. The title therefore suggests a broader scope than what is actually developed.

**Well noted and revised as indicated above! Thank you!**

ABSTRACT

The abstract presents the topic and the general approach of the study, but the way in which the results are formulated requires adjustment.

Statements regarding the effects of the policies are written in conclusive terms, when they correspond to interpretations derived from qualitative analysis. This should be adjusted to reflect the type of evidence that supports the findings.

The description of the qualitative component is not precise. The use of “focus group discussions (n = 40)” may be interpreted as referring to the number of groups rather than the number of participants, which introduces ambiguity.

The final part of the abstract emphasizes general implications, but it does not clearly define the specific contribution of the study. The abstract should state more precisely what the study contributes.

In addition, there is an imbalance between the presentation of results and the formulation of implications, with greater emphasis on the latter.

**The abstract has been extensively revised and the raised points incorporated. We have also ensured consistency with the findings, discussion, and study implication. The Abstract now reads as follows:**

**Abstract**

**Introduction**: Women engaged in informal food vending in Kisumu faced precarious occupational conditions that were intensified by the COVID-19 pandemic. Notwithstanding their critical contribution to urban household food security, these vendors were marginalized from policy-making processes, which were predominantly restricted to technical experts and national government officials.

**Methods**: Utilizing the Intersectionality-Based Policy Analysis (IBPA) framework, this research investigated engagement of women informal food venders and the disparities within COVID-19 containment and control policies. Conducted from July 10, 2024 to October 15, 2024, the study employed qualitative methodologies, including key informant interviews (n=20), in-depth interviews (n=20), and four focus group discussions with ten participants each (n=40) to document the experiences of women vendors regarding pandemic-related regulations. Moreover, four policy documents were purposively selected and reviewed. Participants were selected through purposive sampling in partnership with Pamoja Community Based Organisation, and thematic analysis was performed according to IBPA principles.

**Results**: The findings indicate that the exclusion of women vendors from decision-making yielded gender-insensitive policies that exacerbated existing inequalities. The policy formulation process failed to account for the diverse and intersecting social identities of these vendors. Additionally, fragmented policy implementation subjected women to physical assault, harassment, and extortion, thereby increasing gender-based vulnerabilities.

**Discussion**: In the event of future emergencies, it is imperative that governments establish inclusive, gender-responsive policies that prioritize the perspectives of informal food vendors to ensure sustainable urban food systems. Governments should develop inclusive and gender-sensitive policies while centering the voices of women informal food vendors for a sustainable urban food system. This research advances the integration of intersectional policy analysis within health research, championing principles of equity and social justice to enhance the participation of marginalized groups in the development and execution of health-oriented regulations, particularly in emergency situations.

**Keywords:** Intersectionality-Based Policy Analysis; Gender-responsive policies; COVID-19 Control Measure; Informal Food Vendors; Secondary Cities; Global South

INTRODUCTION

The introduction presents a broader context than in the original version and improves the articulation of the problem.

However, it does not include an explicit statement of the objective or the research questions. The purpose of the study can be inferred from the text, but it is not presented directly, which affects the clarity of the manuscript.

The term “secondary cities” is used without an operational definition, which introduces ambiguity in the scope of the analysis.

The transition between the general contextualization of the problem and the specific delimitation of the study is not clearly established.

Conceptual Framework (IBPA)

The IBPA framework is developed in greater detail, but its application is not consistent.

The manuscript indicates that the “time and space” principle is not used, but it is later included in the description of the analysis. This inconsistency should be resolved.

In several parts of the manuscript, IBPA principles appear as already defined analytical categories, without showing clearly how they are constructed from the empirical material.

**The Introduction section has been extensively revised and the suggested comments integrated. The IBPA framework has been discussed more extensively to show its application in the study and the term “secondary cities” defined. A clear aim/objective of the paper is also included in the introduction section. The introduction now reads as follows:**

**Introduction**

In early 2020, the World Health Organization (WHO) characterized COVID-19 a global pandemic requiring immediate public health action in all fronts (World Health Organization (WHO), 2020). Governments across the world, subsequently, imposed mandatory containment measures such as lockdowns and curfews (Koh, 2020). Safety protocols such as the use of face masks, handwashing, sanitizing, and physical distancing were also introduced to curb the spread of the coronavirus (Mwakisha et al., 2024). These control measures coupled with the effects of the diseases – COVID-19 had devastating effects on global trade and markets, which affected both the formal and informal economies across the world (Alon et al., 2022; Alon et al., 2020; Mishra & Rampal, 2020). Within the informal sector, the control policies triggered events that shaped social and economic outcomes differently for men and women engaged in the sector (Kabeer et al., 2021); hence, exposing and exacerbating the existing socioeconomic and cultural inequalities, especially in urban centres (Finn, 2023; Finn & Kobayashi, 2020; Odhiambo et al., 2025; Onyango et al., 2021; Sharifi & Khavarian-Garmsir, 2020; Ukala et al., 2020).

In cities in the global South, women disproportionately bore the burden of the pandemic and its control measures, with women food vendors experiencing an array of systemic challenges (Afifu et al., 2021; Kiplagat et al., 2025; Onyango et al., 2021; Onyango et al., 2023). These challenges encompassed reduced market hours, restricted movement, disruption of food supply chains, increased household and caregiving responsibilities, alongside significant income loss and heightened food insecurity (Afifu et al., 2021; Kimani-Murage et al., 2022). The impact was particularly severe as many women-led informal food businesses that rely on robust social interaction and physical proximity their customers on the streets – elements that were curtailed by the pandemic control measures (Finn & Kobayashi, 2020). Such measures exacerbated extant inequalities, largely due to a failure to integrate the specific socio-cultural and economic exigencies faced by women into policy frameworks (Odhiambo et al., 2025; Ukala et al., 2020).

Furthermore, the absence of gender-sensitive considerations in policy initiatives introduced unique complications for women during the pandemic (UN Women, 2024). Women informal food vendors faced substantial barriers in securing financial assistance from both governmental and private financial institutions (Donga & Chimucheka, 2024; Sinyolo et al., 2022). In the African context, these difficulties were intensified by entrenched socio-cultural norms and patriarchal structures, which frequently undermine women's autonomy and ownership of assets required as collateral for credit access (Fletschner & Kenney, 2014; Kiplagat et al., 2025).

Across the majority of the cities within the Global South —and specifically across secondary cities, women are over-represented in the informal economic sector, with a heavy concentration in the food industry (Donga & Chimucheka, 2024). These secondary cities function as critical regional, administrative, and economic focal points and are characterized by a strong urban-rural connection and rapid urbanization (Pendras & Williams, 2021). Within the context of Kenya, Kisumu City stands as a major secondary city where women constitute approximately 60% of the total informal traders, primarily engaged in food vending (Kisumu, 2020; Opiyo et al., 2018). These women-led businesses play a key role in bolstering urban household food needs and livelihoods (Kiplagat et al., 2025; Odhiambo et al., 2025). Yet, pandemic-related policy frameworks failed to account for the distinct vulnerabilities of women in these informal food sectors. This neglect led to an escalation in gender-based and intimate partner violence, while simultaneously undermining the capacity of women informal enterprises to contribute to the social and economic fabric of the city (Afifu et al., 2021; John et al., 2021; Roever & Skinner, 2016; Smith, 2023). Simultaneously, increased unpaid care work and household responsibilities impeded women’s full engagement in paid work (Wenham et al., 2020).

However, limited studies have explored gender-specific gaps in COVID-19 control policies in secondary cities in the Global South. In particular, the impacts of the control measures on women-led informal food businesses in secondary cities remains an underexplored research area (Malou, 2024). Most existing studies have focused on the broader informal sector in major metropolitan cities of the Global South, such as Nairobi, Kampala, Karachi, West Bengal, and Johannesburg (Afifu et al., 2021; Anwar et al., 2023; Mondal & Chakraborty, 2022; Pinchoff et al., 2021; Rwafa-Ponela et al., 2022; Suubi et al., 2022). Policy analyses on COVID-19 in Durban, Nairobi and Kampala indicated that women operating within the urban informal economy were systematically excluded from COVID-19 policy processes (Afifu et al., 2021; Skinner et al., 2021; Suubi et al., 2022). Furthermore, intersectional analyses in Karachi revealed that lockdown mandates exacerbated gendered inequalities, manifesting in heightened domestic violence and intensified caregiving responsibilities, even as women maintained their critical role in household provisioning (Anwar et al., 2023). In context of West Benghal, Mondal and Chackraborty (2022) posited that women informal workers had limited influence on policy decisions during the pandemic. As emphasized by Martínez and Young (2022), there exists a critical dearth of research centred on secondary cities around the impacts of the pandemic of women in the informal food sector.

Secondary cities have a distinct role in fostering social, economic, and cultural connections between the rural and urban communities. These cities are often characterized by informality and are marginalized, especially during emergencies (Kiplagat et al., 2025). In the case of Kisumu City, COVID-19 policies were formulated at the national government level in Nairobi, a primary city in Kenya. Such centralized governance effectively excluded the lived realities of vulnerable populations in secondary cities, particularly, women.

Kisumu City is the economic hub for western Kenya and the city’s economy is largely driven by informal trade (Opiyo et al., 2018). Over 60% of the informal businesses in the city are female owned, the majority of whom reside in informal settlements and belong to the Luo or Luhya speaking communities (Kiplagat et al., 2025). In these communities, gender roles are strictly delineated, with domestic responsibilities and caregiving traditionally assigned to women and girls, whose social standing is often diminished by prevailing patriarchal systems. The implementation of pandemic containment measures disproportionately affected these women-led businesses, as the closure of educational institutions and the increased need for medical care for family members imposed substantial additional caregiving burdens (Kiplagat et al., 2025; Odhiambo et al., 2025). Additionally, systemic power imbalances and the predominance of men in leadership roles resulted in the marginalization of female perspectives, particularly those from the informal food sector, in the formulation of pandemic response strategies.

This study extends our understanding of the extent to which the perspectives of women business-owners were considered in the formulation and implementation of the pandemic control policies in secondary cities in the Global South. Understanding the gendered dimensions of the COVID-19 policies on women engaged in informal food businesses is essential for advocating for equitable policy frameworks that safeguard the food and nutritional security and economic stability of urban households, particularly, in times of emergencies. The study adopted the intersectionality-based policy analysis (IBPA) framework (Hankivsky et al., 2014) to investigate the influence of power dynamics and systemic structures on the marginalization of women food vendors within secondary cities during the COVID-19 pandemic. The IBPA framework facilitated an analysis that extended beyond gender as an individual level factor, enabling an exploration of how intersecting identities, policies and regulations – such as the COVID-19 control measures construct social locations and inequities marginalize specific groups, such as the women food vendors.

We utilized seven of the eight key principles of the IBPA framework – the principles of equity, power, intersecting categories, multi-level analysis, reflexivity, social justice, and diverse knowledges (Hankivsky et al., 2014). These seven IBPA framework principles (See Figure 1 below) guided the analysis of the COVID-19-related policies, key informant interviews, focus group discussions and in-depth interview data as well as the interpretation of the findings.

The principle of intersecting categories was employed to examine the interactions between various overlapping social identities – including gender, age, sexual orientation, marital status, educational attainment, cultural orientation, and socio-economic standing—and pandemic-related control strategies in intensifying the marginalization of female vendors. Furthermore, the analysis assessed whether these policies acknowledged the heterogeneous identities of informal food vendors and the specific requirements arising from such positionalities. By applying an intersectional perspective to policy review and qualitative narrative analysis, this study identified the ways in which social locations influenced women’s experiences of the pandemic policies and how inequalities predicated upon the design, dissemination, and interpretation of (Kanenberg & Leal, 2019).

In integrating the principle of power and multi-level analysis, we sought to highlight how overlapping power either facilitated inclusion or exacerbated systemic inequities for female vendors within the informal sector. In the Kenyan context—where healthcare service delivery is devolved to county and municipal authorities while legislative mandate remains centralized federal mandate, complex hierarchies of power emerge. Within such complex governance environments, the management of a public health crisis such as the COVID-19 pandemic necessitated a multisectoral approach characterized by deliberate engagement of the diverse partners, particularly the disadvantaged populations – such as the informal food vendors.

Furthermore, our analytical framework integrated the gender equity lens and the principle of social justice to examine the extent of gender inclusivity with the COVID-19 control measures, while considering the overlapping layers of power in the policy processes. By applying the principle of diverse knowledge, the framework enabled us to interrogate whether the voices of women in informal food vending were integrated in policy development and how power structures influenced the inclusion or exclusion of their diverse perspectives in policy decision making. Whilst studies in other jurisdictions indicated that governments neglected the gender-specific requirements of women during COVID-19 policy responses (Chitando, 2020), the application of the IBPA framework in this study enabled the advancement of the analysis beyond gender to also consider other intersecting identities, tenets of power dynamics, promotion of a social justice lens, and appreciation of diverse knowledge in policy analysis. We applied the principles while acknowledging the relevance of being self-reflexive. Notably, the IBPA principle of time and space, which analyses the dynamics of policy inequalities over time and across historical and geographical spaces, was excluded from this study. This is because the research focussed on the gendered COVID-19 policy dynamics at a specified time and place.

**<<<<Figure 1: The conceptual framework for the IBPA guiding principles for analysis of COVID-19 control policies & the experiences of female informal food vendors>>>**

MATERIALS AND METHODS

The section presents an expansion compared to the original version; even so, it does not allow a precise reconstruction of the procedure followed in the study. This limits the evaluation of the study’s rigor and the coherence between design, data generation, and analysis.

The text combines two levels that should be distinguished: the description of the design and the justification of that design. When both are presented together, it becomes difficult to identify what was actually done and why it was done in that way. In a methodological section, the priority is to make the procedure traceable; justification may be included, but it should not interfere with the operational description.

**Thank you for this observation. It has been considered in this revised version and the methodological section should now have more clear and detailed procedure that was adopted in this research.**

Participant selection is not sufficiently delimited. The groups included in the study are mentioned, but their distribution across techniques (interviews, focus groups, and other components) is not specified. The number of participants per technique and per market is also not indicated. In addition, it is not clear whether different inclusion criteria were applied across techniques. Without this information, it is not possible to evaluate the internal consistency of the design or its adequacy for the type of analysis conducted.

This has been addressed and the exact number of participants across the 5 markets and in the different data collection points presented in Table 1 in the paper.

The description of the sampling strategy includes statements that do not allow reconstruction of the selection logic. The indication that each market included “at least 12 participants distributed across techniques” does not clarify whether this refers to a minimum per technique, a cumulative total, or a distribution conditioned by fieldwork conditions. This point is relevant because it affects comparability across markets and the density of information generated by each technique.

**This has been addressed and the exact number of participants across the 5 markets and in the different data collection points presented in Table 1 in the paper.**

There are gaps in basic procedural aspects. The manuscript does not describe how focus groups were organized (group size, criteria for composition, number of sessions), who conducted them, or under what conditions they were carried out. It also does not specify the language in which interviews and focus groups were conducted, nor how translation was handled and incorporated into the analysis. These elements directly affect the interpretation of qualitative data and should be clearly described.

**Thank you for pointing this out. This has been addressed in the methods section. A section of the methods now reads as follows:**

**Primary data collection**

Research assistants trained in qualitative research techniques and fluent in English, Swahili, and the local Luo dialect, conducted participant recruitment and facilitated information sessions to support primary data collection through face-to-face interviews. The choice of language used was determined by the participant’s preference with most key informants preferring English. Most of the IDI and FGD participants preferred Swahili and/or Luo. The interviews were audio-recorded with the consent of participants. Sessions conducted in English were transcribed verbatim, while those conducted in Swahili or Luo were translated into English as they were being transcribed by the research assistant that had facilitated the sessions. The resulting transcripts were subsequently reviewed by the research assistants and two authors (PMO & EOO), who possess fluency in all three languages, to ensure the accuracy of the translations and data transcriptions.

Semi-structured interview guides were developed and utilized to facilitate KIIs, IDIs, and FGDs. These instruments were designed to elicit discussions regarding key topics relevant to the experiences of women vendors during and following the implementation of COVID-19 pandemic policies. We explored the gendered considerations in the policies and the engagement women vendors in the formulation and implementation of such policies. We probed for the specific experiences with the implementation of the COVID-19 control measures and the direct and indirect impacts on the women-led businesses. Data collection was undertaken until theoretical saturation was achieved– the point at which no new ideas or themes emerged from the interviews. The research team maintained reflexivity throughout the process, and field notes maintained by research assistants were involved in the data analysis. Following each interview, the research assistants summarized the key insights, and conducted daily debriefing meetings with the broader research team.

***Key informant interviews*:** We conducted (*n* = 20) face-to-face interviews with representatives the County Government of Kisumu, market leaders, and women group leaders, community loaning and microfinance representatives, community health promoters and leaders. The interviews lasted approximately 30 – 60 minutes with the key informants including compliance experts, enforcement offices, revenue collectors, and municipal and city government officials. Based on the expertise of the different government and community officials, we obtained in-depth insights into the policy formulation and implementation as well as the implications of policy regulations to women informal food vendors. We also sought to gather information on the interaction of the different entities/organizations and their supports to women informal food vendors during and after the pandemic.

***In-depth interviews*** (*n* = 20) included female and some male food vendors who operated food businesses during and after the COVID-19 pandemic. The male participants were included to capture their perspectives of the experiences of female food vendor with the pandemic control measures. The recruitment was facilitated through our community partner – Pamoja CBO and in collaboration with the market leader. The market leaders invited potential participants to an information session and interested vendors gave informed consent and were invited to a face-to-face interview at a convenient place of their choice. The one-on-one interview participants were interviewed to understand the impacts of COVID-19-related policies on their business and household responsibilities, caregiving duties, interaction with customers and authority, food supply, physical and mental well-being, food security status, and the coping mechanisms they adopted. All the interviews were audio-recorded, translated, and transcribed to facilitate data analysis and interpretation.

***Focus group discussions*** (*n* = 40) include four FGDs, each comprising ten women informal food vendors who were recruited through our community partner and the women market leaders. The women market leaders organized for an information session with the potential participants and those who met the inclusion criteria and gave informed consent were invited to centralized location close to the various key markets, with most of the group discussions being conducted in the community halls close to the markets. The face-to-face group discussion facilitated a participatory space for the women vendors to reflect as a collective on the impact of COVID-19 policies on their social and economic spheres. The participants shared their experiences with the enforcement and compliance of curfews, physical-distancing, movement restrictions, market closures, and the health and safety protocols that were imposed after the implementation of the COVID-19 policies. They shared the gendered effects of the policies articulated some recommendations towards future policy reforms to limit the negative impacts of policies formulated with limited consultations.

**Data analysis**

Primary qualitative data (IDIs, KIIs, and FGDs) were all audio-recorded following receipt of informed consent from all the participants. While KIIs were conducted in English and transcribed verbatim, IDIs and FGDs were primarily facilitated in Swahili or Luo, subsequently requiring translation into English and transcription for analysis. The transcription and translation were conducted by the local research assistants who facilitated the interviews and the discussion and were reviewed by the authors PMO and EOO who are fluent in English, Swahili, and Luo. Comprehensive data sets—comprising transcripts, policy documents, field notes, reflexive journals, and interview summaries—were imported into NVivo (QSR International, version 15) for systematic coding, management, and analysis.

The criterion used to determine that no new information was being generated is not defined. Saturation is mentioned, but the manuscript does not explain how it was established or at what point in the fieldwork it was considered to have been reached. Without this information, it is not possible to assess the sufficiency of the sample.

**Thanks for the comment. A section of the methods now reads as follows:**

**Primary data collection**

Research assistants trained in qualitative research techniques and fluent in English, Swahili, and the local Luo dialect, conducted participant recruitment and facilitated information sessions to support primary data collection through face-to-face interviews. The choice of language used was determined by the participant’s preference with most key informants preferring English. Most of the IDI and FGD participants preferred Swahili and/or Luo. The interviews were audio-recorded with the consent of participants. Sessions conducted in English were transcribed verbatim, while those conducted in Swahili or Luo were translated into English as they were being transcribed by the research assistant that had facilitated the sessions. The resulting transcripts were subsequently reviewed by the research assistants and two authors (PMO & EOO), who possess fluency in all three languages, to ensure the accuracy of the translations and data transcriptions.

Semi-structured interview guides were developed and utilized to facilitate KIIs, IDIs, and FGDs. These instruments were designed to elicit discussions regarding key topics relevant to the experiences of women vendors during and following the implementation of COVID-19 pandemic policies. We explored the gendered considerations in the policies and the engagement women vendors in the formulation and implementation of such policies. We probed for the specific experiences with the implementation of the COVID-19 control measures and the direct and indirect impacts on the women-led businesses. Data collection was undertaken until theoretical saturation was achieved– the point at which no new ideas or themes emerged from the interviews. The research team maintained reflexivity throughout the process, and field notes maintained by research assistants were involved in the data analysis. Following each interview, the research assistants summarized the key insights, and conducted daily debriefing meetings with the broader research team.

Regarding informed consent, the manuscript indicates that it was “written or verbal,” while other sections refer to written consent. This inconsistency should be resolved and presented in a uniform manner.

**This inconsistency has ben corrected to who that participants ether gave verbal or written consents depending on their ability to write.**

The document analysis component is mentioned as part of the design, but the procedure applied is not described. Criteria for selecting policies are indicated, but the manuscript does not explain how documents were analyzed, what categories were used, or how this component was integrated with interview and focus group data. Given that the study is presented as a policy analysis, this is a central element.

**Thank you for this valuable comment. The sub-sections of the methods have been revised and more details regarding this feedback added in the analysis and Quality &Rigor sections. The subsections now read as follows:**

***……***

***Policy document analysis***

The analysis of the four policy documents included in this study was guided by seven core principles of the Intersectionality-Based Policy Analysis (IBPA) framework: power, equity, intersecting categories, multi-level analysis, social justice, diverse knowledges, and reflexivity (Hankivsky et al., 2014). Adopting Braun and Clarke's(Braun & Clarke, 2006) reflexive thematic analysis, the researchers engaged in an iterative process of reading and re-reading the documents to gain familiarity with key control measures, objectives, and intervention areas designed to mitigate the effects of the COVID-19. Through a combination of deductive analysis, guided by the IBPA framework, and inductive analysis, a thematic code set was developed to facilitate the emergence of both pre-defined and new themes (Creswell, 2014; Strauss & Corbin, 1990). The predefined themes aligned with IBPA principles, encompassing power dynamics, intersecting identities, social justice, equity, multi-level analysis and reflexivity. The theme codes, transcripts, field notes, interview summaries, and the post-pandemic recovery policies were then imported and organized in NVivo QSR International, version 15, for line-by-line coding. To validate the theme code sets and the coding and analysis process, an independent peer researcher (GR) performed an independent analysis and cross-checked the analysis for consistency and accuracy. This process of peer checking allowed for multiple interpretations and understanding of the experiences of women informal food vendors and the identification of gaps within the policy documents (Hay, 2016). This peer-review process facilitated multiple interpretations of the experiences of women informal food vendors and assisted in identifying policy gaps. Rigor and trustworthiness were further established by triangulating evidence from policy documents, key informant narratives, and data from informal food vendors.

***Primary data analysis***

The analysis of the primary data from the KII, IDI, and FGD were also conducted inductively and deductively guided by the IBPA framework and following Braun and Clarke’s reflexive thematic analysis approach (Braun & Clarke, 2006; Hankivsky et al., 2014). The research team established initial themes and codes through prolonged immersion in the data, which included active participation in transcription and exhaustive review of the transcripts. These codes were subsequently collated into themes and sub-themes to establish a refined code set. All primary materials were imported into NVivo (QSR International, version 15) for comprehensive coding. Participant perspectives were accurately represented through the use of direct quotes (Tong et al., 2007). To ensure analytical rigor, peer debriefing was employed to verify the theme codes and the consistency of the line-by-line coding. Finally, data triangulation across primary sources and policy documents was utilized to enhance the credibility and trustworthiness of the finding.

**Quality & Rigor**

Various methodological strategies were employed to ensure the rigor of this study. First, data triangulation was achieved by synthesizing multiple sources, including policy document analysis and primary data from KIIs, FGDs, and IDIs. The integration of the policy document analysis and the qualitative evidence was an important element of triangulation to ensure rigor. The application of both inductive and deductive analytical approaches facilitated the triangulation of theoretical frameworks with lived experience perspectives. Furthermore, a purposive sampling technique was utilized to select respondents possessing specific expertise and lived experience (Johnson et al., 2020) relevant to COVID-19 control policies, community leadership, governance, and informal food vending Local research assistants from Pamoja CBO contributed to the data collection process and participated in peer review and debriefing sessions to maintain consistency in data collection, analysis, and interpretation. This peer debriefing process was supported through regular consultations among the research team members, specifically EOO, RG, and PMO.

**Ethical considerations**

Ethics approval for the study was granted by the University of Alberta Research Ethics Review Board (Protocol: Pro00138854) and AMREF Kenya Research Ethics Board (ESRC P1670/2024). We also obtained a verbal for participants that were unable to write a written consent if they were capable of writing. All participants gave informed consent (verbal or written) and received an honorarium of CAD $10 (KES 1,000), to honor their time and contributions to the study.

Finally, the relationship between the IBPA framework and the thematic analysis is not clearly described. The manuscript refers to both approaches, but it does not show how they were articulated in practice: whether IBPA guided coding, whether it was used to reorganize categories, or whether it was applied at a later interpretative stage. This lack of definition affects the methodological coherence of the study.

More details on IBPA framework and how it guided our analysis has been added in the introduction section and the use of both deductive (IBPA) and inductive in the analysis to develop themes included in the methods section. The Introduction section and the methods sections now reads as follows:

**Introduction**

…………….

This study extends our understanding of the extent to which the perspectives of women business-owners were considered in the formulation and implementation of the pandemic control policies in secondary cities in the Global South. Understanding the gendered dimensions of the COVID-19 policies on women engaged in informal food businesses is essential for advocating for equitable policy frameworks that safeguard the food and nutritional security and economic stability of urban households, particularly, in times of emergencies. The study adopted the intersectionality-based policy analysis (IBPA) framework (Hankivsky et al., 2014) to investigate the influence of power dynamics and systemic structures on the marginalization of women food vendors within secondary cities during the COVID-19 pandemic. The IBPA framework facilitated an analysis that extended beyond gender as an individual level factor, enabling an exploration of how intersecting identities, policies and regulations – such as the COVID-19 control measures construct social locations and inequities marginalize specific groups, such as the women food vendors.

We utilized seven of the eight key principles of the IBPA framework – the principles of equity, power, intersecting categories, multi-level analysis, reflexivity, social justice, and diverse knowledges (Hankivsky et al., 2014). These seven IBPA framework principles (See Figure 1 below) guided the analysis of the COVID-19-related policies, key informant interviews, focus group discussions and in-depth interview data as well as the interpretation of the findings.

The principle of intersecting categories was employed to examine the interactions between various overlapping social identities – including gender, age, sexual orientation, marital status, educational attainment, cultural orientation, and socio-economic standing—and pandemic-related control strategies in intensifying the marginalization of female vendors. Furthermore, the analysis assessed whether these policies acknowledged the heterogeneous identities of informal food vendors and the specific requirements arising from such positionalities. By applying an intersectional perspective to policy review and qualitative narrative analysis, this study identified the ways in which social locations influenced women’s experiences of the pandemic policies and how inequalities predicated upon the design, dissemination, and interpretation of (Kanenberg & Leal, 2019).

In integrating the principle of power and multi-level analysis, we sought to highlight how overlapping power either facilitated inclusion or exacerbated systemic inequities for female vendors within the informal sector. In the Kenyan context—where healthcare service delivery is devolved to county and municipal authorities while legislative mandate remains centralized federal mandate, complex hierarchies of power emerge. Within such complex governance environments, the management of a public health crisis such as the COVID-19 pandemic necessitated a multisectoral approach characterized by deliberate engagement of the diverse partners, particularly the disadvantaged populations – such as the informal food vendors.

Furthermore, our analytical framework integrated the gender equity lens and the principle of social justice to examine the extent of gender inclusivity with the COVID-19 control measures, while considering the overlapping layers of power in the policy processes. By applying the principle of diverse knowledge, the framework enabled us to interrogate whether the voices of women in informal food vending were integrated in policy development and how power structures influenced the inclusion or exclusion of their diverse perspectives in policy decision making. Whilst studies in other jurisdictions indicated that governments neglected the gender-specific requirements of women during COVID-19 policy responses (Chitando, 2020), the application of the IBPA framework in this study enabled the advancement of the analysis beyond gender to also consider other intersecting identities, tenets of power dynamics, promotion of a social justice lens, and appreciation of diverse knowledge in policy analysis. We applied the principles while acknowledging the relevance of being self-reflexive. Notably, the IBPA principle of time and space, which analyses the dynamics of policy inequalities over time and across historical and geographical spaces, was excluded from this study. This is because the research focussed on the gendered COVID-19 policy dynamics at a specified time and place.

**<<<<Figure 1: The conceptual framework for the IBPA guiding principles for analysis of COVID-19 control policies & the experiences of female informal food vendors>>>**

**Methods**

…………

***Policy document analysis***

The analysis of the four policy documents included in this study was guided by seven core principles of the Intersectionality-Based Policy Analysis (IBPA) framework: power, equity, intersecting categories, multi-level analysis, social justice, diverse knowledges, and reflexivity (Hankivsky et al., 2014). Adopting Braun and Clarke's(Braun & Clarke, 2006) reflexive thematic analysis, the researchers engaged in an iterative process of reading and re-reading the documents to gain familiarity with key control measures, objectives, and intervention areas designed to mitigate the effects of the COVID-19. Through a combination of deductive analysis, guided by the IBPA framework, and inductive analysis, a thematic code set was developed to facilitate the emergence of both pre-defined and new themes (Creswell, 2014; Strauss & Corbin, 1990). The predefined themes aligned with IBPA principles, encompassing power dynamics, intersecting identities, social justice, equity, multi-level analysis and reflexivity. The theme codes, transcripts, field notes, interview summaries, and the post-pandemic recovery policies were then imported and organized in NVivo QSR International, version 15, for line-by-line coding. To validate the theme code sets and the coding and analysis process, an independent peer researcher (GR) performed an independent analysis and cross-checked the analysis for consistency and accuracy. This process of peer checking allowed for multiple interpretations and understanding of the experiences of women informal food vendors and the identification of gaps within the policy documents (Hay, 2016). This peer-review process facilitated multiple interpretations of the experiences of women informal food vendors and assisted in identifying policy gaps. Rigor and trustworthiness were further established by triangulating evidence from policy documents, key informant narratives, and data from informal food vendors.

***Primary data analysis***

The analysis of the primary data from the KII, IDI, and FGD were also conducted inductively and deductively guided by the IBPA framework and following Braun and Clarke’s reflexive thematic analysis approach (Braun & Clarke, 2006; Hankivsky et al., 2014). The research team established initial themes and codes through prolonged immersion in the data, which included active participation in transcription and exhaustive review of the transcripts. These codes were subsequently collated into themes and sub-themes to establish a refined code set. All primary materials were imported into NVivo (QSR International, version 15) for comprehensive coding. Participant perspectives were accurately represented through the use of direct quotes (Tong et al., 2007). To ensure analytical rigor, peer debriefing was employed to verify the theme codes and the consistency of the line-by-line coding. Finally, data triangulation across primary sources and policy documents was utilized to enhance the credibility and trustworthiness of the finding.

**Quality & Rigor**

Various methodological strategies were employed to ensure the rigor of this study. First, data triangulation was achieved by synthesizing multiple sources, including policy document analysis and primary data from KIIs, FGDs, and IDIs. The integration of the policy document analysis and the qualitative evidence was an important element of triangulation to ensure rigor. The application of both inductive and deductive analytical approaches facilitated the triangulation of theoretical frameworks with lived experience perspectives. Furthermore, a purposive sampling technique was utilized to select respondents possessing specific expertise and lived experience (Johnson et al., 2020) relevant to COVID-19 control policies, community leadership, governance, and informal food vending Local research assistants from Pamoja CBO contributed to the data collection process and participated in peer review and debriefing sessions to maintain consistency in data collection, analysis, and interpretation. This peer debriefing process was supported through regular consultations among the research team members, specifically EOO, RG, and PMO.

**Ethical considerations**

Ethics approval for the study was granted by the University of Alberta Research Ethics Review Board (Protocol: Pro00138854) and AMREF Kenya Research Ethics Board (ESRC P1670/2024). We also obtained a verbal for participants that were unable to write a written consent if they were capable of writing. All participants gave informed consent (verbal or written) and received an honorarium of CAD $10 (KES 1,000), to honor their time and contributions to the study.

ANALYSIS

The manuscript mentions the participation of an independent researcher, but their role is not clearly defined.

**This has been addressed and details about the role of an independent research – peer reviewer added.**

Validation strategies are mentioned, including triangulation and related approaches, but they are not described in sufficient detail to understand how they were implemented.

**Quality and rigor section has been revised with more details on how the various strategies were applied in the study.**

RESULTS

The results are organized thematically and allow the reader to follow the general direction of the analysis; however, the section presents problems of structure, internal consistency, and differentiation between levels of information.

The section does not begin directly with the findings. The opening paragraphs return to methodological elements and describe again the organization of the analysis. This weakens the distinction between methods and results and delays the presentation of empirical evidence.

**Well noted and has been revised**

An inconsistency is observed in the characterization of the sample. The age range reported in the text does not match the one presented in the corresponding table. This type of discrepancy affects the internal coherence of the manuscript.

In several sections, there is no clear distinction between three levels of information: (i) evidence from analyzed documents, (ii) participant testimonies, and (iii) interpretation developed by the authors. This overlap makes it difficult to identify what corresponds to evidence and what corresponds to analysis.

Repetitions are observed across subthemes, particularly regarding exclusion of vendors, increased economic burden, and institutional control practices. These repetitions do not add new elements and fragment the argument, as the same idea appears in different sections without analytical progression.

The way some findings are introduced tends to be declarative. Repeated use of constructions such as “our analysis revealed” or similar expressions does not add information and can be replaced by direct presentation of the evidence.

**The results section has also been extensively revised and the suggestions incorporated. Much appreciation for all the great contributions and suggestions.**

A problem remains in table numbering, with duplication of “Table 1.”

**Corrected!**

The use of quotations requires adjustment. In some cases, the quotation repeats what has already been stated, without adding new information. In other cases, the quotation and the interpretation are not clearly separated, making it difficult to distinguish the participant’s voice from that of the authors.

**Thanks for this comment. Participants’ direct quotations are italicized in the results section. The unitalicized sections are the key highlights from the quotes. Hopefully this gives a distinction on what is said directly by the participants vs what the authors are hearing or reflecting on from the quotes.**

Additionally, in some sections, results are presented together with immediate interpretation, without clearly delimiting the descriptive level. This is not necessarily incorrect, but it should be handled carefully to ensure that evidence is not subordinated to interpretation from the outset.

**The results section has also been extensively revised and the suggestions incorporated. Much appreciation for all the great contributions and suggestions.**

DISCUSSION

The discussion presents a clear expansion compared to the original version and shows an effort to connect the findings with additional literature and with the principles of the IBPA framework. However, the section still presents problems in the articulation between results, interpretation, and theoretical argument.

The first issue is analytical. IBPA principles are mentioned repeatedly, but it is not always clear how each of them emerges from the empirical material. In several points, principles such as power, equity, social justice, or intersecting categories are introduced as interpretative axes, but the manuscript does not consistently show the transition from the evidence presented in the results to the assignment of that evidence to those principles.

The second issue concerns argumentative sequence. In several parts, the discussion moves from findings to broader claims without sufficient intermediate development, particularly when linking results to normative or constitutional frameworks.

There is also repetition of arguments already presented in the results.

Comparative literature is incorporated, but the criteria for comparison are not always clearly established.

The concept of “compliance fatigue” is introduced without sufficient development.

Some statements adopt a stronger tone than what the qualitative design can support.

Language issues remain and require editorial revision.

**The discussion section has been revised extensively to ensure clear and coherent flow of the key findings in relation to existing literature. The discussion now reads as follows:**

**Discussion**

This study aimed to examine the sociocultural and gender disparities inherent in the formulation and implementation of COVID-19 mitigation policies and the subsequent experiences of women within the informal food vending sector. The findings reveal that the formulation and execution of these pandemic mitigation measures were characterized by a top-down approach that limited the engagement of women food vendors. Furthermore, the policies were fragmented by varying levels of power and power dynamics, reinforced prevailing inequities, and neglected the diverse intersecting identities of informal food vendors. Women vendors were systematically excluded from policy-making processes due to existing governance structures wherein health policies are formulated at the national level while service delivery is delegated to county governments, with most of these roles being male dominated. At the city and community levels, women vendors possess limited power and influence; they are frequently excluded from key decision-making processes due to systemic barriers that reinforce power hierarchies and shape policy outcomes. Our research identified several critical barriers contributing to the restricted engagement of women in the COVID-19 policy-making process within Kisumu. The factors included time constraints necessitated by the urgency of the crisis, sociocultural and gender inequities, a lack of accessible information regarding participation opportunities, and a reliance on technocratic expertise within the fields of health, governance, and policy. Such findings correspond with the observations of Martínez and Young (2022), who argued that the prioritization of technical experts in policy development by governments limited the participation of street vendors in decision-making processes. Furthermore, the pervasive societal culture of patriarchy served as a significant impediment to women involvement in political and leadership roles. Despite constitutional provisions for affirmative action and the establishment of County Women Representative positions in Kenya, policy discussions and community leadership remain predominantly male-dominated. These structural barriers reinforce gender hierarchies and the exclusion of women from decision-making at multiple levels of governance and society. Consequently, this lack of representation diminished the responsiveness of policies to the specific circumstances of women in the informal food vending sector, thereby exacerbating existing socio-economic disparities. (Coalition on Violence Against Women & Making It Work, 2023).

Narratives from the vendors emphasized this systematic exclusion, with participants frequently employing the expression "forced on us" to describe their marginalization and the lack of prior consultation regarding policy initiatives. These exclusionary practices appear to deviate from Article 174 (c) f the Constitution of Kenya, which mandates the enhancement of public participation in decision-making processes affecting the citizens (Laws of Kenya, 2013). Although scholarly evidence indicates that the political empowerment of women in pandemic-related policy-making lead to improved gender-sensitive health and economic outcomes (Abras et al., 2021; Huang & Lin, 2022), the approach in Kisumu City and many other cities in the Global South such as Rangpur in Bangladesh and Rusizi and Rubavu in Rwanda, remained highly centralized within the national Ministry of Health. The development and implementation of mitigation measures were restricted to technical experts and government officials, failing to incorporate community engagement strategies, gender-based considerations, or formal feedback mechanisms for the public, particularly for women operating in the informal food economy.

Furthermore, our study findings indicate that these policies reinforced existing inequities by failing to account for the diverse intersecting identities of marginalized groups, such as women food vendors in secondary cities. The policies did not contextualize the lived realities of these women with reference to their intersecting identities or prevailing sociocultural practices. Women vendors reported instances of inequitable treatment, including assault, harassment, and extortion by law enforcement officers. Similarly, (Solymári et al., 2022) observed that the brutality associated with COVID-19 policy enforcement in Kenyan informal settlements infringed upon fundamental human rights. This occurred against the backdrop of a male-dominated county enforcement team in Kisumu, which contributed to biased regulation enforcement (Loo et al., 2021). Such male dominance in enforcement predisposes women to violence and physical abuse at their places of business, posing severe health and safety risks and curtailing their agency to support their families. These experiences of harassment and the application of excessive force demonstrate how established power structures directly impacted the gendered experiences of women in the informal food sector, with some vendors reporting being compelled to pay bribes of up to five thousand shillings to avoid arrest.

Likewise, research in Nairobi by Wandera et al. (2023) documented that women in the informal workforce were subjected to physical abuse by police and municipal officers during the enforcement of curfews. This indicates a consistent pattern of COVID-19 policy impacts on women vendors across both primary and secondary urban centers.  These findings are corroborated by Kiaka and colleagues who emphasized that the pandemic exacerbated instances of bribery and harassment in the Global South, particularly affecting informal vendors whose survival necessitated significant resilience. Exposure to such harassment and assault restricted women’s agency to facilitate urban household food provision and sustain their families during the pandemic. Consequently, it is argued that during periods of emergency, governments must implement gender-responsive governance systems to facilitate the engagement of women in the informal sector and ensure that policies address the specific requirements and experiences of this marginalized yet essential segment of the urban population.

Our study further provides evidence regarding the absence of an intersectional framework in the formulation of COVID-19 policies. Informal traders in Kisumu constitute a heterogeneous group characterized by diverse genders, ages, socio-economic statuses, cultural backgrounds, marital status, abilities, and religious affiliations, among other intersecting social identities. However, the pandemic mitigation strategies utilized a blanket policy approach that failed to account for the varied settings and specific identities of these vendors. This was also noted by Dutta et al. (2021) in India, where COVID-19 lockdown policies adopted a one-size-fits-all approach and overlooked existing gender differences.

These policy interventions did not consider the contextual factors such as the prevailing socio-economic situation in low- and middle-income countries (LMICs), and the pre-existing inequalities faced by women informal workers, including patriarchal structures and gender-based discrimination. Women vendors were already facing compounded challenges, including the precarity of their businesses and work, gender-based violence, discrimination, economic marginalization, and sexual harassment before the occurrence COVID-19 pandemic. The lack of gendered considerations in the policies worsened the prevailing inequalities creating an additional burden to the women vendors and influencing their resilience post-pandemic. While we acknowledge that the pandemic was a relatively new and urgent event that required immediate action through enforcement of public health orders inform of the control measures, we argue that governments must balance such urgency with inclusive and equitable engagement of women in public policy decision-making during emergencies to mitigate unintended negative impacts.

Women faced societal expectations to take up extra caregiving roles at the expense of paid work. Notably, findings from this study raise key questions regarding the value of unpaid household and caregiving duties undertaken by women. According to the United Nation’s Development Program-UN Women COVID-19 Global Gender Response Tracker, fewer than a third of the COVID-19 policy measures implemented in Kenya applied a gender perspective, with none accounting for unpaid care work (UN Women, 2021, 2022). Our findings extend this work by uncovering the experiences of women to the gender gaps in policies in localized settings of secondary cities.

Additionally, the COVID-19 policy measures introduced a significant number of new regulations with which traders were required to comply, potentially inducing "compliance fatigue" among vendors, considering the strict enforcement and use of force by the enforcement officers. This aligns with the findings of Martínez and Young (2022), which demonstrate that the exclusion of vulnerable groups in policy formulation and implementation undermines policy effectiveness and exacerbates marginalization. Such evidence may inform future policy decisions concerning the meaningful engagement of all stakeholders to achieve inclusive and more equitable policy outcomes, particularly during emergencies. As articulated by Cattapan et al., (2020), collaborative policy development should be guided by the principle of "nothing for us without us," which promotes the substantive participation of vulnerable groups in policy design and execution.

CONCLUSIONS

The conclusions summarize the general topic of the study but do not clearly delimit its specific contribution.

The section moves toward policy recommendations without first clearly establishing the main findings and their analytical contribution.

There is no clear distinction between findings, interpretation, and implications.

Some statements go beyond what can be supported by the study design.

The contribution is present, but not clearly articulated.

**The conclusion has also been revised and now reads as follows:**

**Conclusions**

In conclusion, this research possesses significant scholarly and practical relevance, offering both methodological and substantive contributions. By utilizing an IBPA framework to examine urban informal economies and the experiences of informal food vendors, the study extends policy discourse beyond a singular focus on gender. It underscores the critical role of intersecting identities in shaping the lived experiences of women vendors and facilitates the integration of intersectional policy analysis within health research.

Substantively, this research indicates that pandemic containment measures were developed and enforced utilizing a top-down methodology, which effectively marginalized female informal food vendors and failed to account for their unique needs and diverse intersecting identities. Additionally, pre-existing power dynamics and gender-based vulnerabilities were reinforced and exacerbated by the regulatory measures, including curfews, stay-at-home mandates, and physical distancing protocols.

To address this gap, policy interventions should recognize the unique roles of women informal food vendors by incorporating targeted economic support measures, flexible market regulations, and inclusive communication strategies during public health emergencies. While the global health crisis necessitated immediate public health interventions, these findings emphasize the imperative for inclusive policy-making processes that incorporate female informal food vendors into the policy formulation and implementation processes. For example, governments should adopt gender-responsive emergency governance measures that intentionally solicit and integrate the perspectives and lived realities of informal vendors. Furthermore, governments should institutionalize mechanisms for the meaningful participation of informal vendors in policy design through recognized market associations and community-based collectives. To ensure efficacy, policy interventions must be designed in collaboration with marginalized groups to accommodate social diversity and respond to localized needs. In the context of female informal food vendors, future regulatory frameworks and emergency directives should adopt a gendered perspective to formally recognize their unique contributions and safeguard their role in maintaining urban food security.

Such integration would enhance the gender responsiveness of policy interventions and mitigate structural inequalities. Future scholarly inquiries should expand upon this analysis by conducting comparative studies across various secondary cities to evaluate how intersectional inequalities shape the experiences of informal food vendors under disparate governance and policy frameworks.

ETHICS AND DATA AVAILABILITY

Data availability is not clearly described within the manuscript.

Thank you for this! Revised for clarity.

REFERENCES

Formatting is not consistent.

**We appreciate this observation. The reference have been cross checked for consistency.**

LANGUAGE

The manuscript is understandable, but it presents issues in writing and consistency.

A professional language revision is recommended.

**Thank you for this observation. Extensive editorial review of the manuscript has been done to enhance readability and clarity of the key messages.**
