# Supplementary material for: An intersectionality-based policy analysis of COVID-19 measures and women informal food vendors in Kisumu, Kenya
Source: PLoS One. 2026 Oct 1;21(10):e0359620. doi: 10.1371/journal.pone.0359620 (PMC13630232; doi:10.1371/journal.pone.0359620)
Supplement: Supplementary file 4 [file pone.0359620.s005.docx]

**Response to Editor’s comments**

**NB: Highlighted in yellow are the edited/revised sections in the paper.**

**NB: Highlighted in blue are the editor/reviewers comments and suggested edits**

**NB: Highlighted in gray are our responses to the suggestions/comments**

The revised manuscript demonstrates substantial improvement in both scientific quality and presentation.

- The Introduction has been significantly strengthened, providing a clearer contextualization of COVID-19, gender inequalities, and the importance of secondary cities.
- The expanded description of the Intersectionality-Based Policy Analysis (IBPA) framework, together with the inclusion of its guiding principles, considerably improves the conceptual foundation of the study.
- The Methods section has also been enhanced through clearer descriptions of participant recruitment, data collection, analytical procedures, and ethical considerations.
- In addition, the Discussion now provides a more analytical interpretation of the findings by explicitly linking them to issues of power relations, structural inequalities, intersecting identities, and gender-responsive governance.

**Thank you for acknowledging the improvements made in the paper!**

Despite these important improvements, several minor issues should still be addressed before the manuscript is suitable for publication.

1. Explicitly state the manuscript's contribution

Although the conclusion has been revised, the manuscript would benefit from a more explicit statement of its contributions. Rather than leaving these contributions implicit, consider clearly distinguishing the:

- theoretical contribution (advancing intersectionality-informed policy analysis in informal urban economies);
- methodological contribution (application of the IBPA framework integrated with qualitative inquiry);
- policy contribution (implications for inclusive urban governance and emergency policy development).

The authors should add a concise paragraph summarizing these three contributions in order to strengthen the manuscript's overall impact.

**Thanks for this valuable observation. The comment has been incorporated in the discussion and conclusion section which now reads as follows:**

**Discussion**

………………

This study is not without limitations and it is important to recognize its limits. First, the utilization of a cross-sectional research design restricted data collection to a specific timeframe, specifically between July 10, 2024, and October 15, 2024. Because this period occurred a few years after the COVID-19 pandemic, participant recollections may have lacked certain details that could further enrich the findings. Second, the geographic focus was confined to a single county out of Kenya's 47 counties, which may constrain the generalizability of the outcomes nationwide; nevertheless, these findings may possess transferability to urban centers exhibiting comparable characteristics with Kisumu City. Third, the scope was strictly limited to the informal food economy, thereby restricting generalizability to other segments of the informal sector. Furthermore, the reliance on self-reported data introduces potential social desirability bias, particularly regarding personal reflections on pandemic control measures, their impacts on women-led informal food enterprises, and identified socio-gender policy disparities. Notwithstanding these limitations, prior scholarship has successfully utilized analogous methodological approaches to document lived experiences under pandemic restrictions. Consequently, the findings of this study remain comparable to preceding research and offer significant contributions to the literature and practice regarding the gendered dynamics of the informal food sector in secondary cities within the Global South.

**Conclusions**

In conclusion, this study offers substantial theoretical, methodological, and practical contributions to the literature on urban informal economies, particularly regarding the management of public health emergencies. From a theoretical perspective, by utilizing the IBPA framework, this research advances intersectionality-informed policy analysis in the informal economies. It systematically reveals how blanket COVID-19 mitigation policies failed to account for the heterogeneous, intersecting identities of women food vendors, thereby reinforcing pre-existing socio-economic and cultural disparities. By documenting these gendered experiences of exclusion in both policy formulation and enforcement during emergencies, the study provides a critical empirical basis for shifting governance toward gender-responsive, inclusive frameworks that prioritize the substantive engagement of marginalized populations.

While the COVID-19 pandemic may necessitate immediate public health interventions, findings from the current study emphasize the imperative for inclusive policy-making processes that incorporate female informal food vendors into the policy formulation and implementation processes. Governments must adopt gender-responsive emergency governance measures that intentionally solicit the formal representation of women vendors on emergency advisory committees coupled with mandatory consultation of partners before implementing market restrictions. Through these deliberate policy participatory initiatives that engages the female informal vendors, governments will be able to accommodate social diversity and respond to localized needs while safeguarding their unique contributions and role in maintaining urban food security.

Additionally, the study makes a methodological contribution by demonstrating the relevance of integrating IBPA framework, review of policy documents, and qualitative evidence. In so doing, the study reveals how policies intersect with lived experiences and underscores the significance of qualitative evidence in capturing the human elements of policy impact, specifically, the socioeconomic and gender disparities in secondary cities. Substantively, this research indicates that pandemic containment measures were developed and enforced utilizing a top-down methodology, which effectively marginalized female informal food vendors and failed to account for their unique needs and diverse intersecting identities. Additionally, pre-existing power dynamics and gender-based vulnerabilities were reinforced and exacerbated by the regulatory measures, including curfews, stay-at-home mandates, and physical distancing protocols.

To address this gap, policy interventions should recognize the unique roles of women informal food vendors by incorporating targeted economic support measures (e.g., targeted financial assistance, flexible licensing arrangements, and emergency income protection), flexible market regulations, and inclusive and tailored communication strategies during public health emergencies. Such integration would enhance the gender responsiveness of policy interventions and mitigate structural inequalities. Future scholarly inquiries should expand upon this analysis by conducting comparative studies across various secondary cities to evaluate how intersectional inequalities shape the experiences of informal food vendors under disparate governance and policy frameworks.

2. Clarify the research objective

- The Introduction has been substantially improved; however, it still concludes primarily with the rationale for the study rather than a clearly formulated research objective.
- The authors should add at the end of the Introduction one explicit sentence beginning, for example:
  - "This study aimed to...” or "The objective of this study was..."
  - This would improve the overall structure and clarity of the manuscript.

**We appreciate this feedback. It has been addressed and the introduction to the paper now reads as follows:**

**Introduction**

In early 2020, the World Health Organization (WHO) designated COVID-19 as a global pandemic, necessitating immediate and comprehensive public health interventions (World Health Organization (WHO), 2020). Consequently, governments worldwide implemented mandatory containment measures, including lockdowns and curfews (Koh, 2020). Furthermore, safety protocols such as the utilization of face masks, regular hand hygiene, sanitization, and physical distancing were established to mitigate the transmission of the coronavirus (Mwakisha et al., 2024). These control measures coupled with the effects of the diseases – COVID-19 had devastating effects on global trade and markets, which affected both the formal and informal economies across the world (Alon et al., 2022; Alon et al., 2020; Mishra & Rampal, 2020). Within the informal sector, the control policies triggered events that shaped social and economic outcomes differently for men and women engaged in the sector (Kabeer et al., 2021); hence, exposing and exacerbating the existing socioeconomic and cultural inequalities, especially in urban centres (Finn, 2023; Finn & Kobayashi, 2020; Odhiambo et al., 2025; Onyango et al., 2021; Sharifi & Khavarian-Garmsir, 2020; Ukala et al., 2020).

In cities in the global South, women disproportionately bore the burden of the pandemic and its control measures, with women food vendors experiencing an array of systemic challenges (Afifu et al., 2021; Kiplagat et al., 2025; Onyango et al., 2021; Onyango et al., 2023). These challenges encompassed reduced market hours, restricted movement, disruption of food supply chains, increased household and caregiving responsibilities, alongside significant income loss and heightened food insecurity (Afifu et al., 2021; Kimani-Murage et al., 2022). The impact was particularly severe for many women-led informal food businesses which depend fundamentally on robust social interaction and close physical proximity to their clientele in public spaces—operational dimensions that were drastically restricted by pandemic containment protocols (Finn & Kobayashi, 2020). Such measures exacerbated extant inequalities, largely due to failure to integrate the specific socio-cultural and economic exigencies faced by women into policy frameworks (Odhiambo et al., 2025; Ukala et al., 2020).

Furthermore, the absence of gender-sensitive considerations in policy initiatives introduced unique complications for women during the pandemic (UN Women, 2024). Women informal food vendors faced substantial barriers in securing financial assistance from both governmental and private financial institutions (Donga & Chimucheka, 2024; Sinyolo et al., 2022). In the African context, these difficulties were intensified by entrenched socio-cultural norms and patriarchal structures, which frequently undermine women's autonomy and ownership of assets required as collateral for credit access (Fletschner & Kenney, 2014; Kiplagat et al., 2025).

Across the majority of the cities within the Global South —and specifically across secondary cities, women are over-represented in the informal economic sector, with a heavy concentration in the food industry (Donga & Chimucheka, 2024). These secondary cities function as critical regional, administrative, and economic focal points and are characterized by a strong urban-rural connection and rapid urbanization (Pendras & Williams, 2021). Within the context of Kenya, Kisumu City stands as a major secondary city where women constitute approximately 60% of the total informal traders, primarily engaged in food vending (Kisumu, 2020; Opiyo et al., 2018). These women-led businesses play a key role in bolstering urban household food needs and livelihoods (Kiplagat et al., 2025; Odhiambo et al., 2025). Yet, pandemic-related policy frameworks failed to account for the distinct vulnerabilities of women in these informal food sectors. This neglect led to an escalation in gender-based and intimate partner violence, while simultaneously undermining the capacity of women informal enterprises to contribute to the social and economic fabric of the city (Afifu et al., 2021; John et al., 2021; Roever & Skinner, 2016; Smith, 2023). Simultaneously, increased unpaid care work and household responsibilities during the pandemic impeded women’s full engagement in paid work and in their businesses (Wenham et al., 2020).

However, limited studies have explored gender-specific gaps in COVID-19 control policies in secondary cities in the Global South. In particular, the impacts of the control measures on women-led informal food businesses in secondary cities remains an underexplored research area (Malou, 2024). Most existing studies have focused on the broader informal sector in major metropolitan cities of the Global South, such as Nairobi, Kampala, Karachi, West Bengal, and Johannesburg (Afifu et al., 2021; Anwar et al., 2023; Mondal & Chakraborty, 2022; Pinchoff et al., 2021; Rwafa-Ponela et al., 2022; Suubi et al., 2022). Policy analyses on COVID-19 in Durban, Nairobi and Kampala indicated that women operating within the urban informal economy were systematically excluded from COVID-19 policy processes (Afifu et al., 2021; Skinner et al., 2021; Suubi et al., 2022). Furthermore, intersectional analyses of the COVID-19 control measures in Karachi revealed that lockdown mandates exacerbated gendered inequalities, manifesting in heightened domestic violence and intensified caregiving responsibilities, even as women maintained their critical role in household provisioning (Anwar et al., 2023). Within the context of West Benghal, Mondal and Chackraborty (2022) posited that women informal workers had limited influence on policy decisions during the pandemic. Furthermore, as underscored by Martínez and Young (2022), there exists a critical dearth of research centred on secondary cities around the impacts of the pandemic of women in the informal food sector.

Secondary cities have a distinct role in fostering social, economic, and cultural connections between the rural and urban communities. These cities are often characterized by informality and are marginalized, especially during emergencies (Kiplagat et al., 2025). In the case of Kisumu City, COVID-19 policies were formulated at the national government level in Nairobi, a primary city in Kenya. Such centralized governance effectively excluded the lived realities of vulnerable populations in secondary cities, particularly, women.

Kisumu City is the economic hub for western Kenya and the city’s economy is largely driven by informal trade (Opiyo et al., 2018). Over 60% of the informal businesses in the city are female owned, the majority of whom reside in informal settlements and belong to the Luo or Luhya speaking communities (Kiplagat et al., 2025). In these communities, gender roles are strictly delineated, with domestic responsibilities and caregiving traditionally assigned to women and girls, whose social standing is often diminished by prevailing patriarchal systems. The implementation of pandemic containment measures disproportionately affected these women-led businesses, as the closure of educational institutions and the increased need for medical care for family members imposed substantial additional caregiving burdens (Kiplagat et al., 2025; Odhiambo et al., 2025). Additionally, systemic power imbalances and the predominance of men in leadership roles resulted in the marginalization of female perspectives, particularly those from the informal food sector, in the formulation of pandemic response strategies.

It is against this backdrop that the current study aimed to examine how COVID-19 containment policies impacted women-led informal food businesses in Kisumu City, Kenya. Specifically, it addresses how centralized governance and prevailing gender norms affected operational resilience and excluded female perspectives and lived experiences from pandemic policy formulation. By analyzing the specific gender gaps confronting female informal food vendors, this research expands the understanding of how the perspectives of women entrepreneurs were accounted for in the design and implementation of pandemic control policies within secondary cities of the Global South. Elucidating the gendered dimensions of COVID-19 policy frameworks on women in the informal food sector is imperative for advocating for equitable policy frameworks that safeguard the food and nutritional security and economic stability of urban households, particularly, in times of emergencies.

3. Strengthen the operational policy recommendations

- The revised Discussion and Conclusion now contain stronger policy implications. The policy implications have been substantially strengthened and are considerably more actionable than in the previous version.
- Nevertheless, a few recommendations remain relatively broad and could be further enhanced by briefly illustrating how the proposed governance measures could be operationalized in practice:
  - “… governments should adopt gender-responsive emergency governance measures that intentionally solicit and integrate the perspectives and lived realities of informal vendors." (lines 817-819). This recommendation is appropriate, but it remains relatively general. The manuscript could briefly indicate **how** governments could operationalize this recommendation (e.g., formal representation of women vendors on emergency advisory committees, mandatory stakeholder consultations before implementing market restrictions, or participatory policy forums).
  - "…. governments should institutionalize mechanisms for the meaningful participation of informal vendors in policy design through recognized market associations and community-based collectives." (lines 819-821). This is a stronger recommendation than in the previous version and directly addresses one of the reviewer's concerns. However, it could be strengthened further by briefly indicating **what these mechanisms might involve**, for example permanent consultative committees, representation within municipal emergency response structures, or structured feedback mechanisms during public health emergencies.
  - "To ensure efficacy, policy interventions must be designed in collaboration with marginalized groups to accommodate social diversity and respond to localized needs." (Lines 822-823). Although this recommendation is well justified, it remains somewhat conceptual. Providing one or two examples of concrete policy instruments (e.g., targeted financial assistance, flexible licensing arrangements, emergency income protection, or tailored communication strategies) would enhance its practical value for policymakers.
- Where appropriate, consider providing more concrete examples of how governments or local authorities could implement gender-responsive and intersectionality-informed policies (e.g., stakeholder consultation mechanisms, representation of women vendors in emergency planning, targeted support programmes, or inclusive communication strategies).

**Thank you for these specific suggestions on the recommendations. These have been incorporated in the conclusion section which now reads as follows: See highlighted sections.**

**Conclusions**

In conclusion, this study offers substantial theoretical, methodological, and practical contributions to the literature on urban informal economies, particularly regarding the management of public health emergencies. From a theoretical perspective, by utilizing the IBPA framework, this research advances intersectionality-informed policy analysis in the informal economies. It systematically reveals how blanket COVID-19 mitigation policies failed to account for the heterogeneous, intersecting identities of women food vendors, thereby reinforcing pre-existing socio-economic and cultural disparities. By documenting these gendered experiences of exclusion in both policy formulation and enforcement during emergencies, the study provides a critical empirical basis for shifting governance toward gender-responsive, inclusive frameworks that prioritize the substantive engagement of marginalized populations.

While the COVID-19 pandemic may necessitate immediate public health interventions, findings from the current study emphasize the imperative for inclusive policy-making processes that incorporate female informal food vendors into the policy formulation and implementation processes. Governments must adopt gender-responsive emergency governance measures that intentionally solicit the formal representation of women vendors on emergency advisory committees coupled with mandatory consultation of partners before implementing market restrictions. Through these deliberate policy participatory initiatives that engages the female informal vendors, governments will be able to accommodate social diversity and respond to localized needs while safeguarding their unique contributions and role in maintaining urban food security.

Additionally, the study makes a methodological contribution by demonstrating the relevance of integrating IBPA framework, review of policy documents, and qualitative evidence. In so doing, the study reveals how policies intersect with lived experiences and underscores the significance of qualitative evidence in capturing the human elements of policy impact, specifically, the socioeconomic and gender disparities in secondary cities. Substantively, this research indicates that pandemic containment measures were developed and enforced utilizing a top-down methodology, which effectively marginalized female informal food vendors and failed to account for their unique needs and diverse intersecting identities. Additionally, pre-existing power dynamics and gender-based vulnerabilities were reinforced and exacerbated by the regulatory measures, including curfews, stay-at-home mandates, and physical distancing protocols.

To address this gap, policy interventions should recognize the unique roles of women informal food vendors by incorporating targeted economic support measures (e.g., targeted financial assistance, flexible licensing arrangements, and emergency income protection), flexible market regulations, and inclusive and tailored communication strategies during public health emergencies. Such integration would enhance the gender responsiveness of policy interventions and mitigate structural inequalities. Future scholarly inquiries should expand upon this analysis by conducting comparative studies across various secondary cities to evaluate how intersectional inequalities shape the experiences of informal food vendors under disparate governance and policy frameworks.

The Conclusion is much stronger than in the previous version.

- However, it could be made even more concise by focusing primarily on the principal findings, the study's contribution, and future research directions, while limiting discussion-style interpretation.

Although the Discussion is now more analytical, some repetition of empirical findings remains, particularly regarding exclusion from policy-making, enforcement-related harassment, and the blanket nature of COVID-19 policies.

- The authors should consider reducing descriptive repetition and using the Discussion primarily to interpret these findings through the IBPA framework:
  - The Results already present the issue of extortion/harassment: “The overlapping policy interventions created an avenue for extortion and harassment of the women vendors:” (lines 649-650).
  - The Discussion later repeats the same empirical finding: “Women vendors reported instances of inequitable treatment, including assault, harassment, and extortion by law enforcement officers.” (Lines 719-720). Suggestion: the Discussion could reduce the descriptive repetition and focus directly on interpretation: “*These enforcement practices illustrate how gendered power relations shaped the implementation of COVID-19 measures in informal urban economies*.”

The manuscript would benefit from one final careful language edit (for example, "venders" instead of "vendors" in the Abstract).

**We have revised the discussion section which now reads as follows:**

**Discussion**

This study aimed to examine the sociocultural and gender disparities inherent in the formulation and implementation of COVID-19 mitigation policies and the subsequent experiences of women within the informal food vending sector. The findings reveal that the formulation and execution of these pandemic mitigation measures were characterized by a top-down approach that limited the engagement of women food vendors. Furthermore, the policies were fragmented by varying levels of power and power dynamics, reinforced prevailing inequities, and neglected the diverse intersecting identities of informal food vendors. Women vendors were systematically excluded from policy-making processes due to existing governance structures wherein health policies are formulated at the national level while service delivery is delegated to county governments, with most of these roles being male dominated. At the city and community levels, women vendors possess limited power and influence; they are frequently excluded from key decision-making processes due to systemic barriers that reinforce power hierarchies and shape policy outcomes. Our research identified several critical barriers contributing to the restricted engagement of women in the COVID-19 policy-making process within Kisumu. The factors included time constraints necessitated by the urgency of the crisis, sociocultural and gender inequities, a lack of accessible information regarding participation opportunities, and a reliance on technocratic expertise within the fields of health, governance, and policy. Such findings correspond with the observations of Martínez and Young (2022), who argued that the prioritization of technical experts in policy development by governments limited the participation of street vendors in decision-making processes. Furthermore, the pervasive societal culture of patriarchy served as a significant impediment to women involvement in political and leadership roles. Despite constitutional provisions for affirmative action and the establishment of County Women Representative positions in Kenya, policy discussions and community leadership remain predominantly male-dominated. These structural barriers reinforce gender hierarchies and the exclusion of women from decision-making at multiple levels of governance and society. Consequently, this lack of representation diminished the responsiveness of policies to the specific circumstances of women in the informal food vending sector, thereby exacerbating existing socio-economic disparities. (Coalition on Violence Against Women & Making It Work, 2023).

Narratives from the vendors emphasized this systematic exclusion, with participants frequently employing the expression "forced on us" to describe their marginalization and the lack of prior consultation regarding policy initiatives. These exclusionary practices appear to deviate from Article 174 (c) f the Constitution of Kenya, which mandates the enhancement of public participation in decision-making processes affecting the citizens (Laws of Kenya, 2013). Although scholarly evidence indicates that the political empowerment of women in pandemic-related policy-making lead to improved gender-sensitive health and economic outcomes (Abras et al., 2021; Huang & Lin, 2022), the approach in Kisumu City and many other cities in the Global South such as Rangpur in Bangladesh and Rusizi and Rubavu in Rwanda, remained highly centralized within the national Ministry of Health. The development and implementation of mitigation measures were restricted to technical experts and government officials, failing to incorporate community engagement strategies, gender-based considerations, or formal feedback mechanisms for the public, particularly for women operating in the informal food economy.

Furthermore, our study findings indicate that these policies reinforced existing inequities by failing to account for the diverse intersecting identities of marginalized groups, such as women food vendors in secondary cities. The policies did not contextualize the lived realities of these women with reference to their intersecting identities or prevailing sociocultural practices. These enforcement practices illustrate how gendered power relations shaped the implementation of COVID-19 measures in informal urban economies. Similarly, Solymári et al., (2022) observed that the brutality associated with COVID-19 policy enforcement in Kenyan informal settlements infringed upon fundamental human rights. This occurred against the backdrop of a male-dominated county enforcement team in Kisumu, which contributed to biased regulation enforcement (Loo et al., 2021). Such male dominance in enforcement predisposes women to violence and physical abuse at their places of business, posing severe health and safety risks and curtailing their agency to support their families. These experiences of harassment and the application of excessive force demonstrate how established power structures directly impacted the gendered experiences of women in the informal food sector, with some vendors reporting being compelled to pay bribes of up to five thousand shillings to avoid arrest.

Likewise, research in Nairobi by Wandera et al. (2023) documented that women in the informal workforce were subjected to physical abuse by police and municipal officers during the enforcement of curfews. This indicates a consistent pattern of COVID-19 policy impacts on women vendors across both primary and secondary urban centers.  These findings are corroborated by Kiaka and colleagues (2021) who emphasized that the pandemic exacerbated instances of bribery and harassment in the Global South, particularly affecting informal vendors whose survival necessitated significant resilience. Exposure to such harassment and assault restricted women’s agency to facilitate urban household food provision and sustain their families during the pandemic. Consequently, we argued that during periods of emergency, governments must implement gender-responsive governance systems to facilitate the engagement of women in the informal sector and ensure that policies address the specific requirements and experiences of this marginalized yet essential segment of the urban population.

Our study further provides evidence regarding the absence of an intersectional perspectives in the formulation of COVID-19 policies. Informal traders in Kisumu constitute a heterogeneous group characterized by diverse genders, ages, socio-economic statuses, cultural backgrounds, marital status, abilities, and religious affiliations, among other intersecting social identities. However, the pandemic mitigation strategies utilized a blanket policy approach that failed to account for the varied settings and specific identities of these vendors. This was also noted by Dutta et al., (2021) in India, where COVID-19 lockdown policies adopted a one-size-fits-all approach and overlooked existing gender differences.

These policy interventions did not consider the contextual factors such as the prevailing socio-economic situation in low- and middle-income countries (LMICs), and the pre-existing inequalities faced by women informal workers, including patriarchal structures and gender-based discrimination. Women vendors were already facing compounded challenges, including the precarity of their businesses and work, gender-based violence, discrimination, economic marginalization, and sexual harassment before the occurrence COVID-19 pandemic. The lack of gendered considerations in the policies worsened the prevailing inequalities creating an additional burden to the women vendors and influencing their resilience post-pandemic. While we acknowledge that the pandemic was a relatively new and urgent event that required immediate action through enforcement of public health orders inform of the control measures, we argue that governments must balance such urgency with inclusive and equitable engagement of women in public policy decision-making during emergencies to mitigate unintended negative impacts.

Women faced societal expectations to take up extra caregiving roles at the expense of paid work. Notably, findings from this study raise key questions regarding the value of unpaid household and caregiving duties undertaken by women. According to the United Nation’s Development Program-UN Women COVID-19 Global Gender Response Tracker, fewer than a third of the COVID-19 policy measures implemented in Kenya applied a gender perspective, with none accounting for unpaid care work (UN Women, 2021, 2022). Our findings extend this work by uncovering the experiences of women to the gender gaps in policies in localized settings of secondary cities.

Additionally, the COVID-19 policy measures introduced a significant number of new regulations with which traders were required to comply, potentially inducing "compliance fatigue" among vendors, considering the strict enforcement and use of force by the enforcement officers. This aligns with the findings of Martínez and Young (2022), which demonstrate that the exclusion of vulnerable groups in policy formulation and implementation undermines policy effectiveness and exacerbates marginalization. Such evidence may inform future policy decisions concerning the meaningful engagement of all partners to achieve inclusive and more equitable policy outcomes, particularly during emergencies. As articulated by Cattapan et al., (2020), collaborative policy development should be guided by the principle of "nothing for us without us," which promotes the substantive participation of vulnerable groups in policy design and execution.

This study is not without limitations and it is important to recognize its limits. First, the utilization of a cross-sectional research design restricted data collection to a specific timeframe, specifically between July 10, 2024, and October 15, 2024. Because this period occurred a few years after the COVID-19 pandemic, participant recollections may have lacked certain details that could further enrich the findings. Second, the geographic focus was confined to a single county out of Kenya's 47 counties, which may constrain the generalizability of the outcomes nationwide; nevertheless, these findings may possess transferability to urban centers exhibiting comparable characteristics with Kisumu City. Third, the scope was strictly limited to the informal food economy, thereby restricting generalizability to other segments of the informal sector. Furthermore, the reliance on self-reported data introduces potential social desirability bias, particularly regarding personal reflections on pandemic control measures, their impacts on women-led informal food enterprises, and identified socio-gender policy disparities. Notwithstanding these limitations, prior scholarship has successfully utilized analogous methodological approaches to document lived experiences under pandemic restrictions. Consequently, the findings of this study remain comparable to preceding research and offer significant contributions to the literature and practice regarding the gendered dynamics of the informal food sector in secondary cities within the Global South.
