# Supplementary material for: An intersectionality-based policy analysis of COVID-19 measures and women informal food vendors in Kisumu, Kenya
Source: PLoS One. 2026 Oct 1;21(10):e0359620. doi: 10.1371/journal.pone.0359620 (PMC13630232; doi:10.1371/journal.pone.0359620)
Supplement: Supplementary file 5 [file pone.0359620.s006.docx]

**Response to Editor’s comments**

**NB: Highlighted in yellow are the edited/revised sections in the paper.**

**NB: Highlighted in blue are the editor/reviewers’ comments and suggested edits**

**NB: Highlighted in gray are our responses to the suggestions/comments**

The manuscript does not appear to have received the careful final language edit requested by Reviewer 3. Numerous grammatical errors, typographical mistakes, sentence fragments, and unclear expressions remain throughout the manuscript. Examples include:

**Thanks for these valuable observations. We have done a comprehensive review of the manuscript and revised sentence and paragraphs in the paper to ensure clarity and succinct communication of the key messages. W hope the revisions addressed the grammatical, typological, fragmented sentences, and unclear expressions that were previously in the paper.**

**For example, the below highlighted grammatical/typological edited section now read as follows:**

“West Benghal” rather than “West Bengal” (line 102).

…..

However, research on gender-specific gaps in COVID-19 control policies remains limited, particularly regarding the impact on women-led informal food businesses in secondary cities of the Global South (Malou, 2024). Most existing literature focuses on the broader informal sector in major metropolitan hubs such as Nairobi, Kampala, Karachi, West Bengal, and Johannesburg (Afifu et al., 2021; Anwar et al., 2023; Mondal & Chakraborty, 2022; Pinchoff et al., 2021; Rwafa-Ponela et al., 2022; Suubi et al., 2022). Previous analyses in Durban, Nairobi, and Kampala indicate that women in the urban informal economy were systematically excluded from COVID-19 policy processes (Afifu et al., 2021; Skinner et al., 2021; Suubi et al., 2022). Additionally, studies in Karachi demonstrate that lockdown mandates intensified gendered inequalities—increasing domestic violence and caregiving burdens—even as women remained essential to household food security (Anwar et al., 2023). Similarly, research in West Bengal suggests that women informal workers had little influence on pandemic policy decisions (Mondal & Chakraborty, 2022). Overall, as emphasized by Martínez and Young (2022), there is a critical need for more research focused specifically on how the pandemic affected women in the informal food sector within secondary cities.

“the impacts of the pandemic of women” instead of “on women” (line 105).

**Thanks for highlighting this. It has been revised!**

“K With a population…” (line 198).

**Thanks for highlighting this. It has been revised!**

“Selection participants” as a heading (line 229).

**Thanks for highlighting this. It has been revised!**

“with at least 1 to 2 participants…” beginning as a sentence fragment (line 273).

**Thanks for highlighting this. It has been revised!**

“representatives the County Government” (line 321).

**Thanks for highlighting this. It has been revised!**

“Article 174 (c) f the Constitution” rather than “of the Constitution” (line 715).

**Thanks for highlighting this. It has been revised!**

Finally, the cover letter concludes with the statement, “We look forward to sharing our work in the Health & Place Journal.” Please ensure that all submission materials and correspondence correctly identify PLOS ONE as the journal to which the manuscript is being submitted.

**Very much appreciate this highlight! A revised letter has been submitted.**

**NB: Overall, we have done a comprehensive review of the manuscript to enhance clarity and succinct communication.**
