# Supplementary material for: An intersectionality-based policy analysis of COVID-19 measures and women informal food vendors in Kisumu, Kenya
Source: PLoS One. 2026 Oct 1;21(10):e0359620. doi: 10.1371/journal.pone.0359620 (PMC13630232; doi:10.1371/journal.pone.0359620)
Supplement: Peer review history

# Peer review: Decision Letter 0

**DOI:** 10.1371/journal.pone.0359620.r001

**Authors:** Helen Onyeaka

21 Dec 2025

Dear Dr. Onyango,

Thank you for submitting your manuscript to PLOS ONE. After careful consideration, we feel that it has merit but does not fully meet PLOS ONE’s publication criteria as it currently stands. Therefore, we invite you to submit a revised version of the manuscript that addresses the points raised during the review process.

Please submit your revised manuscript by Feb 04 2026 11:59PM. If you will need more time than this to complete your revisions, please reply to this message or contact the journal office at plosone@plos.org. When you're ready to submit your revision, log on to https://www.editorialmanager.com/pone/ and select the 'Submissions Needing Revision' folder to locate your manuscript file.

- A letter that responds to each point raised by the academic editor and reviewer(s). You should upload this letter as a separate file labeled 'Response to Reviewers'.
- A marked-up copy of your manuscript that highlights changes made to the original version. You should upload this as a separate file labeled 'Revised Manuscript with Track Changes'.
- An unmarked version of your revised paper without tracked changes. You should upload this as a separate file labeled 'Manuscript'.

If applicable, we recommend that you deposit your laboratory protocols in protocols.io to enhance the reproducibility of your results. Protocols.io assigns your protocol its own identifier (DOI) so that it can be cited independently in the future. For instructions see: https://journals.plos.org/plosone/s/submission-guidelines#loc-laboratory-protocols. Additionally, PLOS ONE offers an option for publishing peer-reviewed Lab Protocol articles, which describe protocols hosted on protocols.io. Read more information on sharing protocols at https://plos.org/protocols?utm_medium=editorial-email&utm_source=authorletters&utm_campaign=protocols.

We look forward to receiving your revised manuscript.

Kind regards,

Helen Onyeaka, PhD

Academic Editor

PLOS One

Journal Requirements:

When submitting your revision, we need you to address these additional requirements.

1. Please ensure that your manuscript meets PLOS ONE's style requirements, including those for file naming. The PLOS ONE style templates can be found at

https://journals.plos.org/plosone/s/file?id=wjVg/PLOSOne_formatting_sample_main_body.pdf and

https://journals.plos.org/plosone/s/file?id=ba62/PLOSOne_formatting_sample_title_authors_affiliations.pdf

2. Please include a complete copy of PLOS’ questionnaire on inclusivity in global research in your revised manuscript. Our policy for research in this area aims to improve transparency in the reporting of research performed outside of researchers’ own country or community. The policy applies to researchers who have travelled to a different country to conduct research, research with Indigenous populations or their lands, and research on cultural artefacts. The questionnaire can also be requested at the journal’s discretion for any other submissions, even if these conditions are not met.  Please find more information on the policy and a link to download a blank copy of the questionnaire here: https://journals.plos.org/plosone/s/best-practices-in-research-reporting. Please upload a completed version of your questionnaire as Supporting Information when you resubmit your manuscript.

3. PLOS requires an ORCID iD for the corresponding author in Editorial Manager on papers submitted after December 6th, 2016. Please ensure that you have an ORCID iD and that it is validated in Editorial Manager. To do this, go to ‘Update my Information’ (in the upper left-hand corner of the main menu), and click on the Fetch/Validate link next to the ORCID field. This will take you to the ORCID site and allow you to create a new iD or authenticate a pre-existing iD in Editorial Manager.

4. Thank you for stating the following financial disclosure:

This research was funded by SSHRC-IDG Grant # 430-2023-00856, and a sub-grant from the Social Sciences and Humanities Research Council of Canada (SSHRC grant no. 895-2021-1004) from Wilfrid Laurier University.

Please state what role the funders took in the study. If the funders had no role, please state: "The funders had no role in study design, data collection and analysis, decision to publish, or preparation of the manuscript."

If this statement is not correct you must amend it as needed.

Please include this amended Role of Funder statement in your cover letter; we will change the online submission form on your behalf.

5. Please expand the acronym “SSHRC-IDG” (as indicated in your financial disclosure) so that it states the name of your funders in full.

This information should be included in your cover letter; we will change the online submission form on your behalf.

6. Thank you for stating the following in the Competing Interests section:

The authors declare no conflicts of interest with this paper’s research, authorship, and/or publication.

Please confirm that this does not alter your adherence to all PLOS ONE policies on sharing data and materials, by including the following statement: "This does not alter our adherence to PLOS ONE policies on sharing data and materials.” (as detailed online in our guide for authors http://journals.plos.org/plosone/s/competing-interests). If there are restrictions on sharing of data and/or materials, please state these. Please note that we cannot proceed with consideration of your article until this information has been declared.

Please include your updated Competing Interests statement in your cover letter; we will change the online submission form on your behalf.

7. In the online submission form, you indicated that data supporting reported results in this study are hoisted on the University Of Alberta Google Drive and are not publicly available currently but are available on request.

All PLOS journals now require all data underlying the findings described in their manuscript to be freely available to other researchers, either a. In a public repository, b. Within the manuscript itself, or c. Uploaded as supplementary information.

This policy applies to all data except where public deposition would breach compliance with the protocol approved by your research ethics board. If your data cannot be made publicly available for ethical or legal reasons (e.g., public availability would compromise patient privacy), please explain your reasons on resubmission and your exemption request will be escalated for approval.

8. Please amend your authorship list in your manuscript file to include all authors.

9. Please amend your list of authors on the manuscript to ensure that each author is linked to an affiliation. Authors’ affiliations should reflect the institution where the work was done (if authors moved subsequently, you can also list the new affiliation stating “current affiliation:….” as necessary).

10. Please include captions for your Supporting Information files at the end of your manuscript, and update any in-text citations to match accordingly. Please see our Supporting Information guidelines for more information: http://journals.plos.org/plosone/s/supporting-information.

11. Please remove all personal information, ensure that the data shared are in accordance with participant consent, and re-upload a fully anonymized data set.

Note: spreadsheet columns with personal information must be removed and not hidden as all hidden columns will appear in the published file.

Additional guidance on preparing raw data for publication can be found in our Data Policy (https://journals.plos.org/plosone/s/data-availability#loc-human-research-participant-data-and-other-sensitive-data) and in the following article: http://www.bmj.com/content/340/bmj.c181.long.

12. If the reviewer comments include a recommendation to cite specific previously published works, please review and evaluate these publications to determine whether they are relevant and should be cited. There is no requirement to cite these works unless the editor has indicated otherwise.

13. Please review your reference list to ensure that it is complete and correct. If you have cited papers that have been retracted, please include the rationale for doing so in the manuscript text, or remove these references and replace them with relevant current references. Any changes to the reference list should be mentioned in the rebuttal letter that accompanies your revised manuscript. If you need to cite a retracted article, indicate the article’s retracted status in the References list and also include a citation and full reference for the retraction notice.

Additional Editor Comments:

The manuscript requires a little bit of work to put it in a better form for publication, this work is majorly some additions to the body of the literature of the paper, to make it more engaging and also improve its structure. Writers are encouraged to adhere to the recent review comments and make the necessary adjustments.

Reviewers' comments:

Reviewer's Responses to Questions

**Comments to the Author**

1. Is the manuscript technically sound, and do the data support the conclusions?

Reviewer #1: Yes

Reviewer #2: Yes

**********

2. Has the statistical analysis been performed appropriately and rigorously?

Reviewer #1: N/A

Reviewer #2: N/A

**********

3. Have the authors made all data underlying the findings in their manuscript fully available?

The PLOS Data policy

Reviewer #1: Yes

Reviewer #2: Yes

**********

4. Is the manuscript presented in an intelligible fashion and written in standard English?

Reviewer #1: Yes

Reviewer #2: Yes

**********

Reviewer #1: Reviewer’s Comments

This is a well-written, relevant, and timely manuscript that provides valuable insights into how COVID-19 control measures intersected with gender and informality in the Global South, focusing on women informal food vendors in Kisumu, Kenya. The authors make an important contribution by applying the Intersectionality-Based Policy Analysis (IBPA) framework to an under-researched context, i.e. secondary African cities.

The authors can consider the following comments to improve their work:

• In the introduction Clarify how the IBPA framework adds explanatory power compared to other gender-policy frameworks. Also, include a brief statement of objectives or research questions at the end of the introduction section for clearer reader orientation.

• Also consider adding a concise literature review section just after the introduction that synthesizes global/African evidence on COVID-19, gender, and informality, intersectionality-based policy analyses, and prior IBPA applications to anchor the study’s concepts and contributions. Emphasize the documented gaps in secondary cities and in women vendors’ participation to justify Kisumu’s focus and show how your IBPA approach advances this literature.

• The IBPA framework is described well, but a short conceptual figure or table summarizing the seven principles and how they guided analysis would improve clarity.

• The discussion convincingly interprets findings but could more explicitly link empirical themes to IBPA dimensions (power, equity, social justice, etc.).

• In the discussion also strengthen the comparative insight by briefly noting how Kisumu’s experience relates to other secondary cities.

• The limitations section is honest, but you might add a note on potential recall bias mitigation strategies (e.g., triangulation). Or maybe invert that last paragraph of the paper into areas of further research rather than being too honest with limitations, which might dilute the essence of this paper.

• Include a separate conclusion section that explicitly summarizes the paper’s unique contributions to theory, methodology, and practice. The authors should clearly state how the study advances the application of the Intersectionality-Based Policy Analysis (IBPA) framework in African urban contexts, contributes to gender-responsive policy discourse in secondary cities, and provides actionable insights for inclusive pandemic and post-crisis policymaking

• The paper also need a final proof reading after revision..consider for example some details on Ethics not added on…pg….291 – 294 [Ethics approval for the study was granted by 290 the University of ...... Research Ethics Review Board (Protocol: Pro00138854) and ….a Research Ethics Board (ESRC P1670/2024). We also obtained written consent from all the participants engaged in the study. All participants received an honorarium of CAD $10 (KES 1000), to honor their time and contributions to the study.

• Also check areas still highlighted in Yellow.

Reviewer #2: There is a significant time period between when COVID policies impacted the interviewees, and when the fieldwork was conducted. How might this time difference impacted them and their recall? Has it enabled time for respondents to reflect on their exclusion from policy? Worth addressing this point and reflecting on it in the paper.

I like the methodology, and applaud the efforts of the auhtor/s. The 'key informant interviews (n=20),182 (2) focus group discussions (n= 40) 10 women-only participants across four focus groups, and183 (3) in-depth interview participants (n=20, four men and 16 women)' represents a solid undertaking. Good use of quotes in the results.

What are soem policy implications of this study? Covid policy is rightly critiqued in the paper, but the author/s could be more specific about policy recommendations of their own. are their frameworks to ensure better participation and inclusion in policy in the future? Are there action steps a governing body should be sensitive to to make sure women informal vendors are included? Not just for the next emergency, but in general.

I think the paper could have a little more theoretical engagement. COVID policy in Africa was harmful to informal actors. It 'justified' slum clearances and opened the door for authoritarian intervention. What this work shows is that COVID policy also reveals something about social and political life for female informal actors. These are lessons and insights beyond the pandemic that your scholarship can help reveal. I recommend reading and engaging with the following papers;

Cobbinah, P.B., 2022. Enabling urban planning action in Africa: The praxis and oddity of COVID-19 pandemic response. Journal of planning literature, 37(1), pp.83-87.

Finn, B.M. and Kobayashi, L.C., 2020. Structural inequality in the time of COVID-19: Urbanization, segregation, and pandemic control in sub-Saharan Africa. Dialogues in Human Geography, 10(2), pp.217-220.

Finn, B.M., 2023. Pandemic urbanization: How South Africa’s history of labor and disease control creates its current disparities. Journal of Urban Affairs, 45(3), pp.616-629.

Sharifi, A. and Khavarian-Garmsir, A.R., 2020. The COVID-19 pandemic: Impacts on cities and major lessons for urban planning, design, and management. Science of the total environment, 749, p.142391.

**********

what does this mean?). If published, this will include your full peer review and any attached files.

If you choose “no”, your identity will remain anonymous but your review may still be made public.

**Do you want your identity to be public for this peer review?**  For information about this choice, including consent withdrawal, please see our Privacy Policy

Reviewer #1: **Yes:**  Dr Johannes Bhanye

Reviewer #2: **Yes:**  Brandon Marc Finn

**********

[NOTE: If reviewer comments were submitted as an attachment file, they will be attached to this email and accessible via the submission site. Please log into your account, locate the manuscript record, and check for the action link "View Attachments". If this link does not appear, there are no attachment files.]

To ensure your figures meet our technical requirements, please review our figure guidelines: https://journals.plos.org/plosone/s/figures

You may also use PLOS’s free figure tool, NAAS, to help you prepare publication quality figures: https://journals.plos.org/plosone/s/figures#loc-tools-for-figure-preparation.

NAAS will assess whether your figures meet our technical requirements by comparing each figure against our figure specifications.

# Author comment: Author response to Decision Letter 1

**DOI:** 10.1371/journal.pone.0359620.r002

28 Jan 2026

Response to Editor and reviewers comments

NB: Highlighted in yellow are the edited/revised sections in the paper.

NB: Highlighted in blue are the editor/reviewers comments and suggested edits

NB: Highlighted in gray are our responses to the suggestions/comments

1. When submitting your revision, we need you to address these additional requirements.

Please ensure that your manuscript meets PLOS ONE's style requirements, including those for file naming. The PLOS ONE style templates can be found at

https://journals.plos.org/plosone/s/file?id=wjVg/PLOSOne_formatting_sample_main_body.pdf and

https://journals.plos.org/plosone/s/file?id=ba62/PLOSOne_formatting_sample_title_authors_affiliations.pdf

Checked and edits incorporated

2. Please include a complete copy of PLOS’ questionnaire on inclusivity in global research in your revised manuscript. Our policy for research in this area aims to improve transparency in the reporting of research performed outside of researchers’ own country or community. The policy applies to researchers who have travelled to a different country to conduct research, research with Indigenous populations or their lands, and research on cultural artefacts. The questionnaire can also be requested at the journal’s discretion for any other submissions, even if these conditions are not met. Please find more information on the policy and a link to download a blank copy of the questionnaire here: https://journals.plos.org/plosone/s/best-practices-in-research-reporting. Please upload a completed version of your questionnaire as Supporting Information when you resubmit your manuscript.

• Included as an attachment!

3. PLOS requires an ORCID iD for the corresponding author in Editorial Manager on papers submitted after December 6th, 2016. Please ensure that you have an ORCID iD and that it is validated in Editorial Manager. To do this, go to ‘Update my Information’ (in the upper left-hand corner of the main menu), and click on the Fetch/Validate link next to the ORCID field. This will take you to the ORCID site and allow you to create a new iD or authenticate a pre-existing iD in Editorial Manager.

ORCID iD

• Joyce Kiplagat - No account

• Patrick Mbullo Owuor - https://orcid.org/0000-0002-4550-2667

• Rebecca Gokiert - https://orcid.org/0000-0002-7472-9545

• Elizabeth Opiyo Onyango - https://orcid.org/0000-0002-2736-0108

4. Thank you for stating the following financial disclosure:

This research was funded by SSHRC-IDG Grant # 430-2023-00856, and a sub-grant from the Social Sciences and Humanities Research Council of Canada (SSHRC grant no. 895-2021-1004) from Wilfrid Laurier University. Please state what role the funders took in the study. If the funders had no role, please state: "The funders had no role in study design, data collection and analysis, decision to publish, or preparation of the manuscript."

If this statement is not correct you must amend it as needed.

Please include this amended Role of Funder statement in your cover letter; we will change the online submission form on your behalf.

"The funders had no role in study design, data collection and analysis, decision to publish, or preparation of the manuscript."

5. Please expand the acronym “SSHRC-IDG” (as indicated in your financial disclosure) so that it states the name of your funders in full.

This information should be included in your cover letter; we will change the online submission form on your behalf.

Social Science and Humanities Council – Insight Development Grant (SSHRC –IDG). Is spelt out in the financial disclosure section.

6. Thank you for stating the following in the Competing Interests section:

The authors declare no conflicts of interest with this paper’s research, authorship, and/or publication.

Please confirm that this does not alter your adherence to all PLOS ONE policies on sharing data and materials, by including the following statement: "This does not alter our adherence to PLOS ONE policies on sharing data and materials.” (as detailed online in our guide for authors http://journals.plos.org/plosone/s/competing-interests). If there are restrictions on sharing of data and/or materials, please state these. Please note that we cannot proceed with consideration of your article until this information has been declared.

Please include your updated Competing Interests statement in your cover letter; we will change the online submission form on your behalf.

The statement is included! Thank you!

7. In the online submission form, you indicated that data supporting reported results in this study are hoisted on the University of Alberta Google Drive and are not publicly available currently but are available on request.

All PLOS journals now require all data underlying the findings described in their manuscript to be freely available to other researchers, either a. In a public repository, b. Within the manuscript itself, or c. Uploaded as supplementary information.

This policy applies to all data except where public deposition would breach compliance with the protocol approved by your research ethics board. If your data cannot be made publicly available for ethical or legal reasons (e.g., public availability would compromise patient privacy), please explain your reasons on resubmission and your exemption request will be escalated for approval.

Vcjk;lx]mlnData Availability Statement: Data supporting reported results in this study are hosted on the University Of Alberta Google Drive and are available on request. This is because of the REB approval in which participants were assured that the raw data will not be publicly available and will only be accessed by the research team.

8. Please amend your authorship list in your manuscript file to include all authors.

Noted!

9. Please amend your list of authors on the manuscript to ensure that each author is linked to an affiliation. Authors’ affiliations should reflect the institution where the work was done (if authors moved subsequently, you can also list the new affiliation stating “current affiliation:….” as necessary).

Amended!

10. Please include captions for your Supporting Information files at the end of your manuscript, and update any in-text citations to match accordingly. Please see our Supporting Information guidelines for more information: http://journals.plos.org/plosone/s/supporting-information.

Done!

11. 11. Please remove all personal information, ensure that the data shared are in accordance with participant consent, and re-upload a fully anonymized data set. Note: spreadsheet columns with personal information must be removed and not hidden as all hidden columns will appear in the published file. Additional guidance on preparing raw data for publication can be found in our Data Policy (https://journals.plos.org/plosone/s/data-availability#loc-human-research-participant-data-and-other-sensitive-data) and in the following article: http://www.bmj.com/content/340/bmj.c181.long.

The data in this study is available but give the REB approval in which we assured the study participants that the raw data will only be available to the research team and only the summaries and de-identified quotes would be shared publicly in project report and other publication, we are unable to share the raw data publicly.

12. If the reviewer comments include a recommendation to cite specific previously published works, please review and evaluate these publications to determine whether they are relevant and should be cited. There is no requirement to cite these works unless the editor has indicated otherwise.

Noted!

13. Please review your reference list to ensure that it is complete and correct. If you have cited papers that have been retracted, please include the rationale for doing so in the manuscript text, or remove these references and replace them with relevant current references. Any changes to the reference list should be mentioned in the rebuttal letter that accompanies your revised manuscript. If you need to cite a retracted article, indicate the article’s retracted status in the References list and also include a citation and full reference for the retraction notice.

Noted! And revised accordingly!

Additional Editor Comments:

The manuscript requires a little bit of work to put it in a better form for publication, this work is majorly some additions to the body of the literature of the paper, to make it more engaging and also improve its structure. Writers are encouraged to adhere to the recent review comments and make the necessary adjustments.

Well noted and addressed as shown bellow!

Reviewers' comments:

Reviewer's Responses to Questions

Comments to the Author

1. Is the manuscript technically sound, and do the data support the conclusions?

The manuscript must describe a technically sound piece of scientific research with data that supports the conclusions. Experiments must have been conducted rigorously, with appropriate controls, replication, and sample sizes. The conclusions must be drawn appropriately based on the data presented.

Reviewer #1: Yes

Reviewer #2: Yes

________________________________________

2. Has the statistical analysis been performed appropriately and rigorously?

Reviewer #1: N/A

Reviewer #2: N/A

________________________________________

3. Have the authors made all data underlying the findings in their manuscript fully available?

The PLOS Data policy requires authors to make all data underlying the findings described in their manuscript fully available without restriction, with rare exception (please refer to the Data Availability Statement in the manuscript PDF file). The data should be provided as part of the manuscript or its supporting information, or deposited to a public repository. For example, in addition to summary statistics, the data points behind means, medians and variance measures should be available. If there are restrictions on publicly sharing data—e.g. participant privacy or use of data from a third party—those must be specified.

Reviewer #1: Yes

Reviewer #2: Yes

________________________________________

4. Is the manuscript presented in an intelligible fashion and written in standard English?

PLOS ONE does not copyedit accepted manuscripts, so the language in submitted articles must be clear, correct, and unambiguous. Any typographical or grammatical errors should be corrected at revision, so please note any specific errors here.

Reviewer #1: Yes

Reviewer #2: Yes

________________________________________

5. Review Comments to the Author

Please use the space provided to explain your answers to the questions above. You may also include additional comments for the author, including concerns about dual publication, research ethics, or publication ethics. (Please upload your review as an attachment if it exceeds 20,000 characters)

Reviewer #1: Reviewer’s Comments

This is a well-written, relevant, and timely manuscript that provides valuable insights into how COVID-19 control measures intersected with gender and informality in the Global South, focusing on women informal food vendors in Kisumu, Kenya. The authors make an important contribution by applying the Intersectionality-Based Policy Analysis (IBPA) framework to an under-researched context, i.e. secondary African cities.

The authors can consider the following comments to improve their work:

1. In the introduction Clarify how the IBPA framework adds explanatory power compared to other gender-policy frameworks. Also, include a brief statement of objectives or research questions at the end of the introduction section for clearer reader orientation.

Thank you for this valuable feedback. We have incorporated this and a section of the introduction now reads as follows:

.....

To explore how COVID-19 control policies accounted for and addressed the unique challenges and needs of women working in informal food businesses and whether their perspectives were considered in policy formulation and implementation in Kisumu City, this study adopted the intersectionality-based policy analysis framework (IBPA) (Hankivsky et al., 2014). The study utilized the seven principles of the IBPA framework including equity, power, intersecting categories, multi-level analysis, reflexivity, social justice, and diverse knowledges (Hankivsky et al., 2014). These guided the analysis of the COVID-19-related policies, key informant interviews, focus group discussions and in-depth interview transcripts, as well as the interpretation of the findings

2. Also consider adding a concise literature review section just after the introduction that synthesizes global/African evidence on COVID-19, gender, and informality, intersectionality-based policy analyses, and prior IBPA applications to anchor the study’s concepts and contributions. Emphasize the documented gaps in secondary cities and in women vendors’ participation to justify Kisumu’s focus and show how your IBPA approach advances this literature.

I appreciate this feedback. We have incorporated additional context literature to strengthen the introduction and create some foundation for the IBP framework and the subsequent sections of the paper. The introduction now reads as follows:

Introduction

The COVID-19 pandemic had devastating effects on global trade and markets, which affected both the formal and informal economies (Alon et al., 2022; Alon et al., 2020; Mishra & Rampal, 2020). Following the WHO declaration of the global pandemic, governments across the world imposed mandatory containment measures such as lockdowns and curfews (Koh, 2020). Additionally, safety protocols such as the use of face masks, handwashing, sanitizing, and physical distancing were introduced to curb the spread of coronavirus (Mwakisha et al., 2024). The pandemic control policies triggered events that shaped social and economic outcomes differently for men and women engaged in the informal economy (Kabeer et al., 2021).

As pointed out in most COVID-19 related literature, the pandemic exposed and exacerbated socioeconomic and cultural inequalities in most urban populations with women bearing a disproportionate burden (Finn, 2023; Finn & Kobayashi, 2020; Odhiambo et al., 2025; Onyango et al., 2021; Sharifi & Khavarian-Garmsir, 2020; Ukala et al., 2020). Women food vendors experienced varied challenges including reduced market hours, restricted movement, disruption of food supply chains, increased household and caregiving responsibilities, loss of income, and food insecurity (Afifu et al., 2021; Kimani-Murage et al., 2022). This is because most of the women-led informal food businesses rely on rich social interactions and close proximity engagements and yet these were restricted with the pandemic control measures (Finn & Kobayashi, 2020). The measures deepened existing inequalities due to the lack of consideration of socio-cultural and economic challenges faced by women (Odhiambo et al., 2025; Ukala et al., 2020). Additionally, lack of consideration of gender inequities in policy responses presented unique problems for women during the pandemic (UN Women, 2024). Economically, women informal food vendors faced challenges in accessing financial support from government and financial institutions (Donga & Chimucheka, 2024; Sinyolo et al., 2022). In the African context, these were particularly pronounced due to embedded socio-cultural practices and the dominance of patriarchy, which undermined women’s possession and authority over assets that could act as collateral in financial access (Fletschner & Kenney, 2014; Kiplagat et al., 2025).

In most urban centers of the Global South, women are over-represented in the informal economy, particularly within the food sector (Donga & Chimucheka, 2024). In Kisumu City for example, women make up to 60% of all the informal traders, majorly engaged in food vending (Kisumu, 2020; Opiyo et al., 2018). Yet, the COVID-19 policies did not consider the unique challenges of women in the informal food sectors leading to in increased gender and intimate partner violence, hindering the contributions of women-led informal food businesses in supporting urban food needs and livelihoods (Afifu et al., 2021; John et al., 2021; Roever & Skinner, 2016; Smith, 2023). Simultaneously, increased unpaid care work and household responsibilities impeded women’s full engagement in paid work (Wenham et al., 2020).

In secondary cities in the global South such as Kisumu City, the experiences with the pandemic control measures were not any different, yet limited studies have explored gender-specific gaps in COVID-19 control policies in such contexts. In particular, the impacts of the control measures on women-led informal food businesses in secondary cities remains an underexplored research area (Malou, 2024). Most existing studies have focused on the broader informal sector in major cities of the global South, including Nairobi, Kampala, Karachi, West Bengal, and Johannesburg (Afifu et al., 2021; Anwar et al., 2023; Mondal & Chakraborty, 2022; Pinchoff et al., 2021; Rwafa-Ponela et al., 2022; Suubi et al., 2022). Policy analyses on COVID-19 in Durban, Nairobi and Kampala showed that women in the urban informal economy were excluded from COVID-19 policy processes (Afifu et al., 2021; Skinner et al., 2021; Suubi et al., 2022). In Karachi, the findings of an intersectional analysis revealed that lockdowns resulted in gendered inequalities with women experiencing increased domestic violence and care responsibilities while also maintaining provisioning role to their households (Anwar et al., 2023). In West Benghal, Mondal and Chackraborty (2022) argued that women informal workers had limited influence on policy decisions during the pandemic. As Martínez and Young (2022) note, there is a dearth of research in secondary cities on the impacts of pandemic-related policies on women in the informal food sector.

Secondary cities have unique roles in fostering urban-rural linkages within the social, economic, and cultural spheres. In terms of policy development, these cities are often characterized by informality and are marginalized, especially during emergencies (Kiplagat et al., 2025). In the case of Kisumu City, COVID-19 policies were formulated at the national government level in Nairobi, a primary city, excluding the lived realities of vulnerable populations in secondary cities, particularly women.

Kisumu City is the economic hub for western Kenya and the city’s economy is largely driven by informal trade (Opiyo et al., 2018). Over 60% of the informal businesses in the city are female owned with majority living in the informal settlements and identifying as being from the Luo or the Luhya speaking communities (Kiplagat et al., 2025). In these communities, gender roles and responsibilities are well-defined with care work and household chore relegated to women and girls who are less-valued due to patriarchy. With the pandemic control measure, the women-led businesses in the city were significantly impacted as additional care responsibilities emerged with closure of schools and sick family members requiring care (Kiplagat et al., 2025; Odhiambo et al., 2025). Additionally, with the existing power imbalance between the males and females in the communities and the over representation of males in decision-making positions, the female voices, particularly those in the informal food sector were less likely to be reflected within the pandemic control measures.

This paper extends and enables our understanding of the extent to which the perspectives of women business-owners were considered in the formulation and implementation of the pandemic control policies in small-to-medium sized cities in the global South. Understanding the gendered dimensions of the COVID-19 policies on women informal food vendors secondary cities is key in recommending inclusive policy responses that supports the food security needs and livelihoods of urban households in policy formulation, and more so, in times of emergencies.

3. The IBPA framework is described well, but a short conceptual figure or table summarizing the seven principles and how they guided analysis would improve clarity.

Thanks for this suggestion! A conceptual framework for the IBPA framework guiding principles to the study is included in the paper. A section of the introduction now reads as follows and with the conceptual framework reflected below.

………

The study adopted the intersectionality-based policy analysis framework (IBPA), utilizing the principles of equity, power, intersecting categories, multi-level analysis, reflexivity, social justice, and diverse knowledges (Hankivsky et al., 2014). These seven IBPA framework principles (See Figure 1 below) guided the analysis of the COVID-19-related policies, key informant interviews, focus group discussions and in-depth interview data as well as the interpretation of the findings. We applied the principle of intersecting categories to explore their various overlapping social identities, such as gender, age, sexual orientation, and marital status, level of education, cultural orientation, and socio-economic status. The intersectional lens was meant to identify how the social locations influenced the women experiences with the policies and understand how inequalities are shaped by how policies are designed, disseminated and understood (Kanenberg & Leal, 2019).

Considering the principle of power and multi-level analysis, we sought to highlight how overlapping power structures fostered inclusion or reinforced inequality for the informal women food vendors. The gender equity lens and the principle of social justice was also applied to understand the gender inclusivity of the COVID-19 policies while considering the overlapping layers of power in policy processes. Furthermore, by applying the principle of diverse knowledge, the framework enabled us to interrogate whether the voices of women in informal food vending were integrated in policy development and how power dynamics shaped the inclusion or exclusion of their diverse perspectives in policy decision making. Whilst studies in other jurisdictions showed that governments overlooked gender-specific needs of women in COVID-19 policy responses (Chitando, 2020), the IBPA framework allowed for the advancing of the principle of social justice. The principles were applied to the analysis by the authors while acknowledging self-reflexivity. However, the IBPA principle of time and space, which analyses the dynamics of policy inequalities over time and across historical and geographical spaces, was not utilized in the study. This is because the research focussed on the gendered COVID-19 policy dynamics at a specified time and place.

4. The discussion convincingly interprets findings but could more explicitly link empirical themes to IBPA dimensions (power, equity, social justice, etc.). In the discussion also strengthen the comparative insight by briefly noting how Kisumu’s experience relates to other secondary cities.

We appreciate this valuable feedback. We have revised the discussion to explicitly link the themes to the IBPA principles the guided our interpretation of the findings and linked the experiences of Kisumu City to other secondary cities. The discussion now reads as follows:

This study aimed to explore the sociocultural and gender gaps in the formulation and implementation of the COVID-19 pandemic control policies and the experiences of women informal food vendors in Kisumu City, Kenya. The study adopted the IBPA fragment to reveal the prevailing gaps within the policies and to incorporate and intersectionality lens in the assessment of the policies and the lived experience narratives from the vendors and key informants. Seven key IBPA principles of power, intersecting identities, multilevel analysis, equity, social justice, multiple knowledges, and reflexivity guided our critical analysis of the data – the policy document and the narratives from the study participants.

Our analysis of the data uncovered that women informal food vendors in Kisumu City were not engaged in the formulation and implementation of the COVID-19 policies. Women vendor narratives alluded to this exclusion, while using the term ‘forced on us’ to show non-participation and lack of prior information of COVID-19 policy processes. This contravenes the provision of the Constitution of Kenya Article 174 (c) which gives powers of self-governance to the people to enhance their participation in making decisions of issues affecting them (Kenya., 2013). Previous research has shown that political empowerment of women and their participation in COVID-19 policy processes produced better gendered outcomes in the health and economic spheres (Abras et al., 2021; Huang & Lin, 2022). However, in the case of Kisumu City and many other cities in the Global South, policy formulation during the pandemic was undertaken at the national government through the Ministry of Health and in collaboration with technical experts and government officials. From our qualitative research, the policy texts did not allude to any community engagement mechanisms and gender considerations. Neither was there any kind of feedback channels provided for the public, and especially women, specifically those in informal food vending.

We identified several key barriers to the limited engagement of women in COVID-19 policy process in Kisumu, such as time limitations and the urgency of response to the crisis, unavailability of information on the need to participate, and the use of technocrats specialized in health, governance and policy matters. Our findings align with previous work by Martínez and Young (2022), who noted that the use of technical expertise in policy development by governments limited the participation of street vendors in decision-making. Another barrier to participation was the societal culture of patriarchy that impeded women participation in politics and key decision-making roles. Although Kenya’s constitution stipulates an affirmative action and creates the position of County Women Representatives, men often dominate the policy discussion tables and community level leadership (Kenyatta, 2023). These structural barriers have consistently fostered women exclusion and gender hierarchies in policy formulation and decision-making both at different levels of government and at societal level (Kenyatta, 2023). It is worth noting that this exclusion affected the responsiveness of the policies to the lived realities of women informal food vendors, worsening the existing social and economic inequalities (Women. & Work., 2023).

Furthermore, our study findings indicate that power dynamics influenced the enforcement of the COVID-19 policies. In that regard, women vendors expressed their experiences with assault, harassment and extortion by law enforcement officers. Similarly, (Solymári et al., 2022) indicated that the brutality that came with COVID-19 policy enforcement in Kenyan slum dwellings infringed on fundamental human rights. This comes against the backdrop of another study conducted in Kisumu City, where outcomes indicated that the county enforcement team was male-dominated, contributing to a biased enforcement of regulations (Loo et al., 2021). The male-dominance in enforcement predisposes women to violence and physical abuse at their places of business, which is a health and safety risk.

Similarly, in Nairobi, Wandera and colleagues (2023) indicated that women informal workers faced physical abuse from police and municipal officers during curfew implementation. This shows a commonality of the COVID-19 policy experiences by women vendors in major cities and secondary cities. This exposure to harassment and assault curtailed women’s agency in undertaking urban household food provision and fending for their families during a pandemic. Our study also provides evidence of the lack of an intersectional approach in the design of the COVID-19 policies. As in the case of the City of Cali, Colombia (Martínez & Young, 2022), informal traders in Kisumu are a heterogeneous group, of different genders, ages, socio-economic status, cultural backgrounds, religion, marital status, abilities, sexual orientation, among others. However, the policies employed a blanket policy approach and failed to consider the different settings and intersecting social identities of the vendors. This was found by Dutta et al. (Dutta et al., 2021) in India, where COVID-19 lockdown policies adopted a one-size-fits-all approach and overlooked existing gender differences.

Other findings suggest that there is need to enhance social protection mechanisms for women in the informal sector to safeguard them from gender-related exploitations (Wandera et al., 2023). The use of excessive force to enforce policies reveal how structures of power impacted the gendered experiences of women informal food vendors. Our research therefore corroborates the findings of Kiaka and colleagues (Kiaka et al., 2021) who highlighted that the COVID-19 pandemic intensified bribery and harassment in cities in the Global South.

The overlapping social locations of the women food vendors in our study highlight the diversity in ways that our study participants experienced the pandemic. The policy interventions did not consider the contextual factors such as the prevailing socio-economic situation in secondary cities in LMICs, and the pre-existing inequalities faced by women informal workers such as the issues of patriarchy and gender inequality. Women vendors were already facing compounded challenges, including the precarity of their businesses and work, gender-based violence, discrimination, economic marginalization, and sexual harassment before the occurrence COVID-19 pandemic. The lack of gendered considerations in the policies worsened the prevailing inequalities creating an additional burden to the women vendors and influencing their resilience post-pandemic.

Women faced societal expectations to take up extra caregiving roles at the expense of paid work. Notably, findings from this study raise key questions regarding the value of unpaid household and caregiving duties undertaken by women. According to the United Nation’s Development Program-UN Women COVID-19 Global Gender Response Tracker, fewer than a third of the COVID-19 policy measures implemented in Kenya applied a gender perspective, with none accounting for unpaid care work (UN Women, 2021, 2022). Our findings extend this work by uncovering the experiences of women to the gender gaps in policies in localized settings of secondary cities.

This study established that governance structures in policy formulation and implementation continued to worsen the existing gender inequalities during and post pandemic. The policies were formulated at the national government level and implemented at the county and city levels. This did not take into account the lived realities that existed in the local level of governance. In the context of IBPA principle of equity, this scenario reveals how power hierarchies shape policy decision making. We noted that women were organized into groups at the market level for collective bargaining, psychosocial and mental wellbeing, and networks for financial support. However, the representatives of these women groups were not engaged in policy processes. In their own words, the women vendors felt that the policies were “forced” on them, and they incurred additional costs in complying while also experiencing reduced business profitability. The cost of purchasing masks and sanitizers, cost of licensing and permits, compounded with the punitive penalties and fines for non-compliance further strained the women vendors economically.

Additionally, the COVID-19 policy measures added to the long list of regulations that the traders needed to comply with, and this had the possibility to cause “compliance fatigue” among the vendors, considering the strict enforcement and use of force by the enforcement officers. This is congruent to the findings of Martínez and Young (2022) that revealed that the exclusion of vulnerable groups in policy formulation and implementation hinders the effectiveness of policies and exacerbates marginalization. This evidence could benefit future policy decisions regarding the meaningful engagement of all stakeholders for inclusive and more equitable policy options, particularly during emergencies. As Cattapan et al., (2020) states, collaborative policy development should be guided by the principle of “nothing for us without us”, which fosters the meaningful participation of vulnerable groups in policy design and implementation.

5. The limitations section is honest, but you might add a note on potential recall bias mitigation strategies (e.g., triangulation). Or maybe invert that last paragraph of the paper into areas of further research rather than being too honest with limitations, which might dilute the essence of this paper.

We have revised the last paragraphs of the discussion section, which now reads as follows:

Additionally, the COVID-19 policy measures added to the long list of regulations that the traders needed to comply with, and this had the possibility to cause “compliance fatigue” among the vendors, considering the strict enforcement and use of force by the enforcement officers. This is congruent to the findings of Martínez and Young (2022) that revealed that the exclusion of vulnerable groups in policy formulation and implementation hinders the effectiveness of policies and exacerbates marginalization. This evidence could benefit future policy decisions regarding the meaningful engagement of all stakeholders for inclusive and more equitable policy options, particularly during emergencies. As Cattapan et al., (2020) states, collaborative policy development should be guided by the principle of “nothing for us without us”, which fosters the meaningful participation of vulnerable groups in policy design and implementation.

This study is not without limitations and it is important to recognize its limits. The choice of a cross-sectional study design meant that data was collected at a specific point in time, and in this case, between July 10, 2024 and October 15, 2024. The period during which data was collected was a few years after the COVID-19 pandemic, hence participants’ recollections could have omitted important details that could enrich the study findings. Additionally, this study only focused on one county out of the 47 counties in Kenya, and the outcome may not be generalized to the rest of the counties. However, the findings may be transferable to cities with similar characteristic like Kisumu City. Additionally, this study focused specifically on the informal food economy limiting the generalizability of the findings to other sectors of the informal economy. Lastly, the study relied on self reported data, which may be prone to social desirability, especially with reference to personalized experiences of the pandemic control measures, the impacts of the pandemic control measures on women-led informal food businesses, and the social and gender gaps in policies. Previous studies have adopted similar approaches where participants have shared their lived experiences with the COVID-19 control measures. Our findings are therefore comparable with those of past studies and this study makes important contributions to research on gendered dynamics of the informal food sector in secondary cities of the Global South.

6. Include a separate conclusion section that explicitly summarizes the paper’s unique contributions to theory, methodology, and practice. The authors should clearly state how the study advances the application of the Intersectionality-Based Policy Analysis (IBPA) framework in African urban contexts, contributes to gender-responsive policy discourse in secondary cities, and provides actionable insights for inclusive pandemic and post-crisis policymaking

Thanks for this excellent suggestion. We have separated and revised the conclusion section which now reads as follow:

Conclusions

In conclusion, this study revealed that the pandemic control measures were formulated and implemented following a top-down approach and that women informal food vendors who were directly impacted by the measures were not engaged in any of the processes. As such, the policies neither considered nor incorporated the unique needs and the diverse intersecting identities reflected within the informal food vendors’ population in secondary cities. Power imbalance and gender related vulnerabilities were reinforced and exacerbated with the control measures such as curfews, stay home orders, and social distancing regulations. While we acknowledge that the pandemic was a relatively new and urgent event that required immediate action through enforcement of public health orders inform of the control measures, we argue balancing of urgency with inclusive and equitable engagement of women by governments in public policy decision-making in times of emergencies is necessary to minimize the unintended impacts. This would enhances the gender responsiveness of the interventions and minimizes inequalities (Wenham et al., 2020). Policy interventions should be developed with the involvement of marginalized groups to account for the existing diversities and respond to their unique needs. In the case of women informal food vendors, future emergency policies should be designed guided by a gendered lens to recognize their unique roles and needs, and ensure they continue to play the vital role of urban household food security.

7. The paper also need a final proof reading after revision. consider for example some details on Ethics not added on…pg….291 – 294 [Ethics approval for the study was granted by 290 the University of ...... Research Ethics Review Board (Protocol: Pro00138854) and ….a Research Ethics Board (ESRC P1670/2024). We also obtained written consent from all the participants engaged in the study. All participants received an honorarium of CAD $10 (KES 1000), to honor their time and contributions to the study.

We have proof read the paper and corrected any editorial areas. The missing information in the paper are for the purpose of blinding as was requested by the editor.

8. Also check areas still highlighted in Yellow.

The areas highlighted in yellow are the sections that have been edited/revised following the recommendations of the editors and the reviewers.

Responses to Reviewer #2

1. Reviewer #2: There is a significant time period between when COVID policies impacted the interviewees, and when the fieldwork was conducted. How might this time difference impacted them and their recall? Has it enabled time for respondents to reflect on their exclusion from policy? Worth addressing this point and reflecting on it in the paper.

Thanks for this great observation. We have reflected on this in the study limitations section. The section now reads as follows:

This study is not without limitations and it is important to recognize its limits. The choice of a cross-sectional study design meant that data was collected at a specific point in time, and in this case, between July 10, 2024 and October 15, 2024. The period during which data was collected was a few years after the COVID-19 pandemic, hence participants’ recollections could have omitted important details that could enrich the study findings. Additionally, this study only focused on one county out of the 47 counties in Kenya, and the outcome may not be generalized to the rest of the counties. However, the findings may be transferable to cities with similar characteristic like Kisumu City. Additionally, this study focused specifically on the informal food economy limiting the generalizability of the findings to other sectors of the informal economy. Lastly, the study relied on self reported data, which may be prone to social desirability, especially with reference to personalized experiences of the pandemic control measures, the impacts of the pandemic control measures on women-led informal food businesses, and the social and gender gaps in policies. Previous studies have adopted similar approaches where participants have shared their lived experiences with the COVID-19 control measures. Our findings are therefore comparable with those of past studies and this study makes important contributions to research on gendered dynamics of the informal food sector in secondary cities of the Global South.

2. I like the methodology, and applaud the efforts of the author/s. The 'key informant interviews (n=20),182 (2) focus group discussions (n= 40) 10 women-only participants across four focus groups, and183 (3) in-depth interview participants (n=20, four men and 16 women)' represents a solid undertaking. Good use of quotes in the results.

Thanks for your positive feedback. We have added details in the methods section to enhance transparency and replicability. The methods section now read as follows:

Methods

Study setting

This study was conducted in Kisumu City, located within the county of Kisumu in western Kenya. It’s proximity to the Lake Victoria, world’s second largest freshwater lake, provides economic benefits of fishing resources, trade, and transport (Opiyo et al., 2018). Kenya has a devolved system of government comprising 47 counties, which was established after the promulgation of the constitution in 2010 (Chau, 2021). In the health sector, the national government oversees policy formulation while the county governments manage the delivery of health services (Chau, 2021). The dominant economic activities carried out in Kisumu include crop and livestock farming, fisheries, trade, manufacturing, construction, and mining (County Government of Kisumu, 2020; Owuor et al., 2024). Kisumu City has an approximate population of 600,000 inhabitants (KNBS, 2020) and has experienced an influx of people from the neighboring rural areas due to its rapidly urbanizing potential (Simiyu et al., 2019). Approximately 64% of the city’s inhabitants reside within the informal settlements (UN-Habitat, 2020), which experience high levels of poverty (Opiyo et al., 2018). These informal settlements include Manyatta A, Manyatta B, Obunga, Bandani, Kaloleni, Nyalenda A, Nyalenda B and Kibos (Simiyu et al., 2019). Additionally, the informal food sector thrives within the city, with a majority of the residents relying on the sector for food provision, with formal supermarkets being utilized less frequently (Kiaka et al., 2021; Opiyo et al., 2018).

We selected Kisumu City due to its vibrant informal food sector and the overrepresentation of women vendors in the urban food space. This makes it a relevant case study area to understand the gendered implications of COVID-19-related policies in regards to secondary cities of the Global South (Opiyo et al., 2018). This study particularly focused on the markets, which are located within the informal settlements in the municipality, including Manyatta, Nyalenda, Obunga, Nyawita, and Bandani.

Study design

This cross-sectional study was implemented from July 10, 2024 to October 15, 2024. A cross-sectional study design was deemed appropriate as it allowed for a deeper understanding of the nuanced lived experiences of the vendors, highlighting the gendered aspects and gaps in the COVID-19 policy responses. We conducted a review of four main pandemic response policies and collected primary qualitative data from the informal food vendors in Kisumu City. The four pandemic response policies that were formulated by the national government and adopted by the county and city governments to curb the spread of COVID-19 and that were reviewed in this study include – Public Health (COVID-19 General Public Safety) (Laws of Kenya, 2020a); Public Order (State Curfew) (Laws of Kenya, 2020d); Public Health (COVID-19 Restriction of Movement of Persons and Related Measures) Rules (Laws of Kenya, 2020b); and Public Health (Prevention, Control and Suppression Of COVID-19) Rules (Laws of Kenya, 2020c). The primary data generation approaches included – Key Informant Interviews (KIIs), In-Depth Interviews (IDIs), and Focus Group Discussion (FGDs) with key informants and informal food vendors in the different informal settlements and markets in Kisumu City.

Selection participants

Policy document sampling

A total of four policy documents (n = 4) were purposefully selected for critical review. These included the Public Health (COVID-19 General Public Safety) (Laws of Kenya, 2020a); Public Order (State Curfew) (Laws of Kenya, 2020d); Public Health (COVID-19 Restriction of Movement of Persons and Related Measures) Rules (Laws of Kenya, 2020b); and Public Health (Prevention, Control and Suppression Of COVID-19) Rules (Laws of Kenya, 2020c). The policy documents were chosen because: (1) were related to government interventions to control and contain COVID-19 pandemic in Kisumu City, either formulated at the national or county level; (2) policies were implemented within Kisumu County; (3) policies impacted the informal food Sector. Details about the four selected COVID-19 control policies are shown below in Table 1:

Table 1: Selected COVID-19 policy documents for review

Policy document Relevance to the study

1. Public Health (COVID-19 General Public Safety) Rules, 2020 (Laws of Kenya, 2020a)

Contained specific health protocols such as physical distancing, wearing of masks, sanitation procedures, COVID-19 screening and prohibition of public gatherings, which impacted business operations for informal food vendors.

2. Public Order (State Curfew) Order, No. 36 of 2020 (Laws of Kenya, 2020d)

Imposition of a 7:00pm to 5:00am curfew, which affected business-operating hours.

3. Public Health (COVID-19 Restriction of Movement of Persons and Related Measures) Rules, 2020 (Laws of Kenya, 2020b)

Set restriction of movement of people into and out of an infected area, curtailing the supply of goods, traders and customers into markets.

4. Public Health (Prevention, Control and Suppression Of COVID-19) Rules, 2020 (Laws of Kenya, 2020c)

Outlined the powers of medical and clinical officers in relation to COVID-19 control and enforcement including search, inspection and decontamination, and closure of premises, with specified penalties for non-compliance. These led to a quarantine, decline in business profitability and closure of some premises where women vendors operated.

Participant sampling

Participants for the primary data collection were selected through our connection with a local community partner – Pamoja Community Based Organisation (Pamoja CBO), a local entity that undertakes community-based initiatives in Kisumu. The organisation’s well-established networks were critical for identifying the potential participants for the study using purposive sampling and snowball sampling techniques.

A total of 80 participants were recruited between July 10, 2024 and October 15, 2024 to participate in this cross-sectional study and they were drawn from the five key markets (Manyatta, Nyalenda, Obunga, Nyawita, and Bandani) in the city. The participants sampled included female informal food vendors and some male vendors and key informants who work directly with the informal food vendors. The participants were deemed to hold the relevant knowledge and information to the study. In collaboration with Pamoja CBO, potential participants were invited to an information session through the market representatives. During the sessions, details about the study were shared to allow participants to make an informed decision about their participation. Individuals who gave a written or verbal informed consent took part in the IDIs (n = 20), KIIs (n = 20), and FGDs (n = 40).

The participants were distributed across the 5 key markets in the city with each market having at least 12 participants across the three data collection methods. Participants were included if they met the following criteria: (1) they had been operating food businesses in and had lived in Kisumu City for over five years; (2) were owners or operators of informal food business during and after the pandemic; (3) were willing to give informed consent to participate in the individual interviews or a group discussion. Participants who did not operate their businesses during and/or after the pandemic and those that did not give consent were excluded.

The key informants (n = 20) were selected across the 5 key markets with at least 1 to 2 participants from each market and others from the relevant government and city departments and community organizations. The key informants were included if they met the follow criteria: (1) they were market leaders or representatives of informal food vendors in the markets; (2) they were representatives from city government or community organizations such as the, community loaning and microfinance organizations, community health promoters and leaders; and (3) they were willing to consent to participate in a one-on-one interview with one of the research team members. Any potential key informants were excluded if they did not meet the above criteria.

Data collection techniques

Policy document analysis

The Public Health (COVID-19 General Public Safety) (Laws of Kenya, 2020a), Public Order (State Curfew) (Laws of Kenya, 2020d), Public Health (COVID-19 Restriction of Movement of Persons and Related Measures) Rules (Laws of Kenya, 2020b), and Public Health (Prevention, Control and Suppression Of COVID-19) Rules (Laws of Kenya, 2020c) were retrieved from the Kenya Law Reports portal. The review aimed to assess whether these pandemic control measures considered the diverse and unique needs of female informal food vendors. Guided by the principles of the IBPA framework, the review of these policies explored how the intersecting social locations of the women informal food vendors and other sociocultural factors were considered in the policy process. This was intended to demonstrate the gender-responsiveness of the policy interventions and identify gaps within the policies, and their implications to women-led in informal food businesses.

Primary data collection

Research assistants trained in qualitative research techniques and fluent in English, Swahili, and the local dialect Luo engaged in the recruitment of potential participants, and in the information session to facilitate primary data collection through face-to-face interviews. The choice of language used was determined by the participant’s preference with most key informants preferring English. Most of the IDI and FGD participants preferred Swahili and/or Luo. The interviews were audio-recorded with the consent of participants with those conducted in English transcribed verbatim and those conducted in Swahili/Luo translated in English and transcribed. The transcripts were then reviewed by the research assistance the two authors (PMO & EOO) who are fluent in all the three languages used. The reviews were done to ensure accuracy in the translation and transcription of the data.

We developed and used semi-structured interview guides to facilitate the KIIs, IDIs, and FGDs. The questions were intended to elicit discussions about specific key topics relevant to the experiences of the women vendors during and after implementation of COVID-19 pandemic policies. We explored the gendered considerations in the policies and the engagement women vendors in the formulation and implementation of such policies. We probed for the specific experiences with the implementation of the COVID-19 control measures and the direct and indirect impacts on the women-led businesses. Data collection was undertaken until saturation – the point at which no new ideas or themes were emerging from the interviews. We remained reflexive through out the research process and the research assistant kept field notes that were included in the data analysis. After every interview, the research assistants summarized the key insights from each interview and held a debrief meeting with the research team at the end of each day’s work.

Key informant interviews: We conducted (n = 20) face-to-face interviews with representatives the County Government of Kisumu, market leaders, and women group leaders, community loaning and microfinance representatives, community health promoters and leaders. The interviews lasted approximately 30 – 60 minutes with the key informants including compliance experts, enforcement offices, revenue collectors, and municipal and city government officials. Based on the expertise of the different government and community officials, we obtained in-depth insights into the policy formulation and implementation as well as the implications of policy regulations to women informal food vendors. We also sought to gather information on the interaction of the different entities/organizations and their supports to women informal food vendors during and after the pandemic.

In-depth interviews (n = 20) included female and some male food vendors who operated food businesses during and after the COVID-19 pandemic. The male participants were included to capture their perspectives of the experiences of female food vendor with the pandemic control measures. The recruitment was facilitated through our community partner – Pamoja CBO and in collaboration with the market leader. The market leaders invited potential participants to an information session and interested vendors gave informed consent and were invited to a face-to-face interview at a convenient place of their choice. The one-on-one interview participants were interviewed to understand the impacts of COVID-19-related policies on their business and household responsibilities, caregiving duties, interaction with customers and authority, food supply, physical and mental well-being, food security status, and the coping mechanisms they adopted. All the interviews were audio-recorded, translated, and transcribed to facilitate data analysis and interpretation.

Focus group discussions (n = 40) include four FGDs, each comprising ten women informal food vendors who were recruited through our community partner and the women market leaders. The women market leaders organized for an information session with the potential participants and those who met the inclusion criteria and gave informed consent were invited to centralized location close to the various key markets, with most of the group discussions being conducted in the community halls close to the markets. The face-to-face group discussion facilitated a participatory space for the women vendors to reflect as a collective on the impact of COVID-19 policies on their social and economic spheres. The participants shared their experiences with the enforcement and compliance of curfews, physical-distancing, movement restrictions, market closures, and the health and safety protocols that were imposed after the implementation of the COVID-19 policies. They shared the gendered effects of the policies articulated some recommendations towards future policy reforms to limit the negative impacts of policies formulated with limited consultations.

Data analysis

Primary qualitative data (IDIs, KIIs, and FGDs were all audio-recorded with informed consent of the participants. All the KIIs were conducted in English and transcribed verbatim. The IDIs and FGDs were mostly facilitated in Swahili or Luo and were translated to English and transcribed for data analysis. The transcription and translation were conducted by the local research assistants who facilitated the interviews and the discussion and were reviewed by the authors PMO and EOO who are fluent in English, Swahili, and Luo. All the transcripts and the policy documents, field notes, reflexive journals, and interview summaries were imported in to NVivo QSR International, version 15 for analysis, coding and management.

Policy document analysis

The analysis of the four policy documents included in this study was guided by seven (7) core principles of the IBPA framework including power, equity, intersecting categories, multi-level analysis, social justice, diverse knowledges, and reflexivity (Hankivsky et al., 2014). The analysis adopted Braun and Clarke’s (Braun & Clarke, 2006) reflexive thematic analysis which involved reading and re-reading the four policy documents to familiarize with the key control measures, the objectives, the key areas of interventions for curbing the COVID-19 pandemic effects. Using both deductive (guided by IBPA framework) and inductive analysis, we developed a thematic code set allowing for pre-defined and new themes to emerge [45, 46]. The predefined themes were based on the IBPA principles of power and power dynamics, intersecting identities, social justice, equity, multi-level analysis, reflexivity, and time and space. The theme codes, transcripts, field notes, interview summaries, and the post-pandemic recovery policies were then imported and organized in NVivo QSR International, version 15, and the data code line by line. To validate the theme code sets and the coding and analysis process, an independent peer researcher performed an independent analysis and cross-checked the analysis for consistency and accuracy. This process of peer checking allowed for multiple interpretations and understanding of the experiences of women informal food vendors and the identification of gaps within the policy documents [47]. To ensure rigour and trustworthiness of the evidence, we triangulated evidence from the policy documents, the narratives from the key informants, and the informal food vendors.

Primary data analysis

The analysis of the primary data from the KII, IDI, and FGD were also condected inductively and deductively guided by the IBPA framework and following Braun and Clarke’s six-phase thematic analysis approach (Braun & Clarke, 2006; Hankivsky et al., 2014). Through prolonged immersion in the data by taking part in the transcription, reviewing the transcripts, and reading and re-reading of the transcripts, the research team identified the initial themes and codes. Key codes were then collated into potential themes and sub-themes, which were then refined to produce a theme code set. All the data – transcripts, field notes, interview summaries, and the theme code set were imported in NVivo QSR International Version 15 for line-by-line coding. Participant quotes and excerpts from the policies were used to convey the themes and to accurately present the participant perspectives (Tong et al., 2007). To ensure rigor, we engaged in peer checking of the theme codes and the line by line coding of the data. The evidence from the different data sources – primary data and policy documents were triangulated to enhance credibility and trustworthiness of the study findings.

Quality & Rigor

We adopted different approaches to achieve rigor in this study. First, we triangulated various sources of data including policy document analysis and primary sources of KIIs, FGDs and IDIs. We adopted inductive and deductive approaches to data analysis, which allowed for triangulation of the theoretical and lived experience perspectives. The use of purposive sampling technique also ensured that we selected the most suitable respondents to the study questions, who had the relevant lived experience and expertise (Johnson et al., 2020) with COVID-19 control policies, community leadership, governance, and informal food vending. We engaged local research assistants through Pamoja CBO who were part of the data collection team and engaged in peer review and peer debrief to ensure consistency in the data collection, analysis, and interpretation. Peer review and debrief was achieved through regular consultation with the researchers in the team – EOO, RG, and PMO.

Ethical considerations

Ethics approval for the study was granted by the University of Alberta Research Ethics Review Board (Protocol: Pro00138854) and Amref Research Ethics Board (ESRC P1670/2024). We also obtained written consent from all the participants engaged in the study. All participants received an honorarium of CAD $10 (KES 1,000), to honor their time and contributions to the study.

5. What are some policy implications of this study? Covid policy is rightly critiqued in the paper, but the author/s could be more specific about policy recommendations of their own. are their frameworks to ensure better participation and inclusion in policy in the future? Are there action steps a governing body should be sensitive to make sure women informal vendors are included? Not just for the next emergency, but in general.

Thanks for this valuable feedback. We have drawn some key conclusions from the study evidence and give some policy recommendations for consideration beyond the COVID-19 pandemic. The conclusion section now reads as follows:

Conclusions

In conclusion, this study revealed that the pandemic control measures were formulated and implemented following a top-down approach and that women informal food vendors who were directly impacted by the measures were not engaged in any of the processes. As such, the policies neither considered nor incorporated the unique needs and the diverse intersecting identities reflected within the informal food vendors’ population in secondary cities. Power imbalance and gender related vulnerabilities were reinforced and exacerbated with the control measures such as curfews, stay home orders, and social distancing regulations. While we acknowledge that the pandemic was a relatively new and urgent event that required immediate action through enforcement of public health orders inform of the control measures, we argue balancing of urgency with inclusive and equitable engagement of women by governments in public policy decision-making in times of emergencies is necessary to minimize the unintended impacts. This would enhances the gender responsiveness of the interventions and minimizes inequalities (Wenham et al., 2020). Policy interventions should be developed with the involvement of marginalized groups to account for the existing diversities and respond to their unique needs. In the case of women informal food vendors, future emergency policies should be designed guided by a gendered lens to recognize their unique roles and needs, and ensure they continue to play the vital role of urban household food security.

6. I think the paper could have a little more theoretical engagement. COVID policy in Africa was harmful to informal actors. It 'justified' slum clearances and opened the door for authoritarian intervention. What this work shows is that COVID policy also reveals something about social and political life for female informal actors. These are lessons and insights beyond the pandemic that your scholarship can help reveal. I recommend reading and engaging with the following papers;

Cobbinah, P.B., 2022. Enabling urban planning action in Africa: The praxis and oddity of COVID-19 pandemic response. Journal of planning literature, 37(1), pp.83-87.

Finn, B.M. and Kobayashi, L.C., 2020. Structural inequality in the time of COVID-19: Urbanization, segregation, and pandemic control in sub-Saharan Africa. Dialogues in Human Geography, 10(2), pp.217-220.

Finn, B.M., 2023. Pandemic urbanization: How South Africa’s history of labor and disease control creates its current disparities. Journal of Urban Affairs, 45(3), pp.616-629.

Sharifi, A. and Khavarian-Garmsir, A.R., 2020. The COVID-19 pandemic: Impacts on cities and major lessons for urban planning, design, and management. Science of the total environment, 749, p.142391.

Thanks for the recommendations. We have read an integrated the key findings in the papers into our paper.

Attachment Submitted filename: Rebuttal to Reviewers and Editors Comments - Jan 17, 2026.docx

# Peer review: Decision Letter 1

**DOI:** 10.1371/journal.pone.0359620.r003

**Authors:** Helen Onyeaka

23 Apr 2026

Dear Dr. Onyango,

Thank you for submitting your manuscript to PLOS ONE. After careful consideration, we feel that it has merit but does not fully meet PLOS ONE’s publication criteria as it currently stands. Therefore, we invite you to submit a revised version of the manuscript that addresses the points raised during the review process.

==============================

**ACADEMIC EDITOR:**

Thank you for submitting your manuscript to PLOS ONE. After careful consideration, we feel that it has merit but does not fully meet PLOS ONE’s publication criteria as it currently stands. Therefore, we invite you to submit a revised version of the manuscript that addresses the points raised during the review process.

Reviewer 1

Overall, the revision shows clear and substantial improvement. Several important issues raised during the previous review have been addressed. In particular:

• The Introduction has been expanded and now provides a broader contextualization of COVID-19 impacts, gender inequalities, and the relevance of secondary cities:

• The manuscript now includes a conceptual illustration of the IBPA framework, which improves the clarity of the analytical approach.

• The Methods section has been strengthened, with clearer explanations of participant recruitment, data collection procedures, and analytical methods.

• Additional information regarding ethical approval and research rigor has been provided.

• The Discussion section has been improved and more explicitly connects the findings with IBPA principles such as power relations, equity, and intersecting identities.

• A distinct Conclusion section has been included, which summarizes the main findings and highlights the policy relevance of the study.

Despite these improvements, several points still require further attention before the manuscript can be considered for publication.

Conceptual positioning of IBPA

• Although the manuscript explains the principles of Intersectionality-Based Policy Analysis (IBPA), it still does not clearly articulate how IBPA provides additional explanatory value compared with other gender or policy analysis frameworks. A clearer justification of the analytical advantage of IBPA would strengthen the theoretical contribution of the study.

Explicit statement of the manuscript’s contribution

• The conclusion would benefit from a clearer articulation of the manuscript’s contribution to theory, methodology, and policy practice. Currently, these contributions remain somewhat implicit:

o The authors are encouraged to explicitly state:

o the theoretical contribution, particularly how the study advances intersectionality-informed policy analysis in the context of urban informal economies;

o the methodological contribution, namely the application of the Intersectionality-Based Policy Analysis (IBPA) framework combined with qualitative methods to examine emergency governance measures;

o the policy contribution, including how the findings inform more inclusive urban governance and crisis-response policies affecting women informal food vendors.

Policy implications

• The discussion and conclusion provide useful general recommendations regarding inclusive policymaking and engagement with women vendors. However, the policy implications remain relatively broad. The manuscript would benefit from more concrete and operational recommendations that could inform local governance or urban policy design.

o “Policy interventions should be developed with the involvement of marginalized groups to account for the existing diversities and respond to their unique needs.”. Suggestion: “The findings demonstrate the importance of integrating intersectionality-informed approaches into urban policy design to ensure that crisis response measures do not disproportionately marginalize women working in informal food systems.”

Language and editorial issues

• While the manuscript has improved stylistically, several minor grammatical and wording issues remain and should be corrected during the next revision.

o The term “IBPA fragment” is incorrect and unclear.

o “The analysis of the primary data from the KII, IDI, and FGD were also condected inductively and deductively guided by the IBPA..”

o “This would enhances the gender responsiveness of the interventions and minimizes inequalities…”

• A careful proofreading is recommended to ensure clarity and consistency.

In addition, the authors should consider also the following recommendations:

• The introduction should end with clear research objectives or research questions.

• In several sections, the Discussion reiterates findings already presented in the Results (e.g., the exclusion of women vendors from policy decision-making, experiences of harassment by enforcement officers, governance structure, and the blanket nature of COVID-19 policies):

o Results section: “Women vendors narrated their lived experiences of unfair treatment, extortion, assault and coercion from the enforcement authorities during the pandemic.” Discussion section: “In that regard, women vendors expressed their experiences with assault, harassment and extortion by law enforcement officers.”

o The Discussion would benefit from stronger analytical interpretation linking these findings to the IBPA framework, particularly regarding power relations, intersecting identities, and structural inequalities in urban governance!

• In Conclusion section:

o The policy recommendations in the conclusion are relevant but remain rather general (e.g., the call for ‘involvement of marginalized groups’ or ‘gender-responsive policy design’). The manuscript would benefit from more concrete and actionable policy suggestions, such as specific mechanisms for engaging women informal food vendors in policy processes or examples of gender-responsive emergency governance measures.

§ “Policy interventions should be developed with the involvement of marginalized groups to account for the existing diversities and respond to their unique needs”. Suggestion: “Policy interventions should recognize the unique roles of women informal food vendors by incorporating targeted economic support measures, flexible market regulations, and inclusive communication strategies during public health emergencies.”

o “The Conclusion section currently includes extended discussion and policy interpretation The authors should avoid repeating discussion and focus on key takeaways:

§ “While we acknowledge that the pandemic was a relatively new and urgent event that required immediate action through enforcement of public health orders inform of the control measures, we argue balancing of urgency with inclusive and equitable engagement of women by governments in public policy decision-making in times of emergencies is necessary to minimize the unintended impacts.” This sentence provides extended interpretation and argumentation, which is typical of the Discussion, is very long, and introduces new reasoning rather than summarizing findings. The conclusion would be stronger if it focused more concisely on summarizing the main findings and the study’s contribution, while limiting extended interpretation and policy discussion.” Suggestion: “Although the pandemic required urgent public health responses, the findings highlight the importance of inclusive policy processes that engage women informal food vendors in emergency decision-making.”

o The authors should highlight future research directions:

§ “Future emergency policies as well as other government policies and interventions should be designed guided by a gendered lens to recognize their unique roles and needs.” The sentence focuses on policy recommendations, but the Conclusion does not mention directions for future research. Suggestion: “Future research could extend this analysis by conducting comparative studies across different secondary cities in order to examine how intersectional inequalities shape the experiences of informal food vendors under varying governance and policy contexts.”

Reviewer 2

The manuscript addresses a relevant problem and provides empirical information on the effects of COVID-19 policies on a population that has been insufficiently studied. The revised version shows a clear effort to respond to previous comments, particularly in the expansion of the introduction, the development of the IBPA framework, and the extension of the discussion.

Even so, the manuscript maintains problems related to the formulation of the study, internal consistency, and methodological clarity that should be addressed before it can be considered in its current form.

RESPONSE TO PREVIOUS COMMENTS

The expansion of the introduction and the incorporation of additional literature are considered addressed. The context is now more solid and better articulated.

The development of the IBPA framework is considered partially addressed. Its description was expanded and a figure was incorporated, but its use is not consistent throughout the manuscript.

The inclusion of explicit objectives or research questions was not addressed. The purpose of the study continues to be distributed across the narrative and is not presented in a direct manner in the introduction.

The discussion was expanded and includes more bibliographic support. However, in several points, the statements are not directly supported by the results, and therefore this aspect is considered partially addressed.

A limitations section was included, but the analysis of its implications is limited. This point is considered partially addressed.

Editorial revision was not addressed. Formal and structural issues remain that should have been corrected at this stage.

GENERAL COMMENTS

The manuscript presents relevant empirical material and addresses an important topic. However, several aspects related to methodological transparency, internal consistency, and analytical clarity need to be strengthened.

In its current form, the manuscript does not clearly define its research objective, does not consistently apply the IBPA framework, and does not provide sufficient detail to reconstruct the methodological procedure. In addition, there are structural issues in the results and discussion sections, including lack of separation between evidence and interpretation, repetition of arguments, and insufficient analytical development in some parts.

These issues do not invalidate the study, but they do require substantial revision.

SPECIFIC COMMENTS

TITLE

The title reflects the content of the manuscript. It includes the analytical approach, the context of COVID-19 control measures, the study population, and the empirical case.

Even so, it requires revision.

First, it is a long title. The number of elements included in a single structure reduces its clarity.

Second, it brings together two analytical levels, policy analysis and lived experiences, that are both part of the manuscript, but in the title, they appear accumulated, making it heavy.

Third, the expression “Secondary Cities” in plural does not fully correspond to the empirical scope of the study, which is based on a single case. Although the manuscript seeks to situate the analysis within that type of context, what is presented is a case study in Kisumu. The title therefore suggests a broader scope than what is actually developed.

ABSTRACT

The abstract presents the topic and the general approach of the study, but the way in which the results are formulated requires adjustment.

Statements regarding the effects of the policies are written in conclusive terms, when they correspond to interpretations derived from qualitative analysis. This should be adjusted to reflect the type of evidence that supports the findings.

The description of the qualitative component is not precise. The use of “focus group discussions (n = 40)” may be interpreted as referring to the number of groups rather than the number of participants, which introduces ambiguity.

The final part of the abstract emphasizes general implications, but it does not clearly define the specific contribution of the study. The abstract should state more precisely what the study contributes.

In addition, there is an imbalance between the presentation of results and the formulation of implications, with greater emphasis on the latter.

INTRODUCTION

The introduction presents a broader context than in the original version and improves the articulation of the problem.

However, it does not include an explicit statement of the objective or the research questions. The purpose of the study can be inferred from the text, but it is not presented directly, which affects the clarity of the manuscript.

The term “secondary cities” is used without an operational definition, which introduces ambiguity in the scope of the analysis.

The transition between the general contextualization of the problem and the specific delimitation of the study is not clearly established.

Conceptual Framework (IBPA)

The IBPA framework is developed in greater detail, but its application is not consistent.

The manuscript indicates that the “time and space” principle is not used, but it is later included in the description of the analysis. This inconsistency should be resolved.

In several parts of the manuscript, IBPA principles appear as already defined analytical categories, without showing clearly how they are constructed from the empirical material.

MATERIALS AND METHODS

The section presents an expansion compared to the original version; even so, it does not allow a precise reconstruction of the procedure followed in the study. This limits the evaluation of the study’s rigor and the coherence between design, data generation, and analysis.

The text combines two levels that should be distinguished: the description of the design and the justification of that design. When both are presented together, it becomes difficult to identify what was actually done and why it was done in that way. In a methodological section, the priority is to make the procedure traceable; justification may be included, but it should not interfere with the operational description.

Participant selection is not sufficiently delimited. The groups included in the study are mentioned, but their distribution across techniques (interviews, focus groups, and other components) is not specified. The number of participants per technique and per market is also not indicated. In addition, it is not clear whether different inclusion criteria were applied across techniques. Without this information, it is not possible to evaluate the internal consistency of the design or its adequacy for the type of analysis conducted.

The description of the sampling strategy includes statements that do not allow reconstruction of the selection logic. The indication that each market included “at least 12 participants distributed across techniques” does not clarify whether this refers to a minimum per technique, a cumulative total, or a distribution conditioned by fieldwork conditions. This point is relevant because it affects comparability across markets and the density of information generated by each technique.

There are gaps in basic procedural aspects. The manuscript does not describe how focus groups were organized (group size, criteria for composition, number of sessions), who conducted them, or under what conditions they were carried out. It also does not specify the language in which interviews and focus groups were conducted, nor how translation was handled and incorporated into the analysis. These elements directly affect the interpretation of qualitative data and should be clearly described.

The criterion used to determine that no new information was being generated is not defined. Saturation is mentioned, but the manuscript does not explain how it was established or at what point in the fieldwork it was considered to have been reached. Without this information, it is not possible to assess the sufficiency of the sample.

Regarding informed consent, the manuscript indicates that it was “written or verbal,” while other sections refer to written consent. This inconsistency should be resolved and presented in a uniform manner.

The document analysis component is mentioned as part of the design, but the procedure applied is not described. Criteria for selecting policies are indicated, but the manuscript does not explain how documents were analyzed, what categories were used, or how this component was integrated with interview and focus group data. Given that the study is presented as a policy analysis, this is a central element.

Finally, the relationship between the IBPA framework and the thematic analysis is not clearly described. The manuscript refers to both approaches, but it does not show how they were articulated in practice: whether IBPA guided coding, whether it was used to reorganize categories, or whether it was applied at a later interpretative stage. This lack of definition affects the methodological coherence of the study.

ANALYSIS

The manuscript mentions the participation of an independent researcher, but their role is not clearly defined.

Validation strategies are mentioned, including triangulation and related approaches, but they are not described in sufficient detail to understand how they were implemented.

RESULTS

The results are organized thematically and allow the reader to follow the general direction of the analysis; however, the section presents problems of structure, internal consistency, and differentiation between levels of information.

The section does not begin directly with the findings. The opening paragraphs return to methodological elements and describe again the organization of the analysis. This weakens the distinction between methods and results and delays the presentation of empirical evidence.

An inconsistency is observed in the characterization of the sample. The age range reported in the text does not match the one presented in the corresponding table. This type of discrepancy affects the internal coherence of the manuscript.

In several sections, there is no clear distinction between three levels of information: (i) evidence from analyzed documents, (ii) participant testimonies, and (iii) interpretation developed by the authors. This overlap makes it difficult to identify what corresponds to evidence and what corresponds to analysis.

Repetitions are observed across subthemes, particularly regarding exclusion of vendors, increased economic burden, and institutional control practices. These repetitions do not add new elements and fragment the argument, as the same idea appears in different sections without analytical progression.

The way some findings are introduced tends to be declarative. Repeated use of constructions such as “our analysis revealed” or similar expressions does not add information and can be replaced by direct presentation of the evidence.

A problem remains in table numbering, with duplication of “Table 1.”

The use of quotations requires adjustment. In some cases, the quotation repeats what has already been stated, without adding new information. In other cases, the quotation and the interpretation are not clearly separated, making it difficult to distinguish the participant’s voice from that of the authors.

Additionally, in some sections, results are presented together with immediate interpretation, without clearly delimiting the descriptive level. This is not necessarily incorrect, but it should be handled carefully to ensure that evidence is not subordinated to interpretation from the outset.

DISCUSSION

The discussion presents a clear expansion compared to the original version and shows an effort to connect the findings with additional literature and with the principles of the IBPA framework. However, the section still presents problems in the articulation between results, interpretation, and theoretical argument.

The first issue is analytical. IBPA principles are mentioned repeatedly, but it is not always clear how each of them emerges from the empirical material. In several points, principles such as power, equity, social justice, or intersecting categories are introduced as interpretative axes, but the manuscript does not consistently show the transition from the evidence presented in the results to the assignment of that evidence to those principles.

The second issue concerns argumentative sequence. In several parts, the discussion moves from findings to broader claims without sufficient intermediate development, particularly when linking results to normative or constitutional frameworks.

There is also repetition of arguments already presented in the results.

Comparative literature is incorporated, but the criteria for comparison are not always clearly established.

The concept of “compliance fatigue” is introduced without sufficient development.

Some statements adopt a stronger tone than what the qualitative design can support.

Language issues remain and require editorial revision.

CONCLUSIONS

The conclusions summarize the general topic of the study but do not clearly delimit its specific contribution.

The section moves toward policy recommendations without first clearly establishing the main findings and their analytical contribution.

There is no clear distinction between findings, interpretation, and implications.

Some statements go beyond what can be supported by the study design.

The contribution is present, but not clearly articulated.

ETHICS AND DATA AVAILABILITY

Data availability is not clearly described within the manuscript.

REFERENCES

Formatting is not consistent.

LANGUAGE

The manuscript is understandable, but it presents issues in writing and consistency.

A professional language revision is recommended.

==============================

Please submit your revised manuscript by Jun 07 2026 11:59PM. If you will need more time than this to complete your revisions, please reply to this message or contact the journal office at plosone@plos.org. When you're ready to submit your revision, log on to https://www.editorialmanager.com/pone/ and select the 'Submissions Needing Revision' folder to locate your manuscript file.

- A letter that responds to each point raised by the academic editor and reviewer(s). You should upload this letter as a separate file labeled 'Response to Reviewers'.
- A marked-up copy of your manuscript that highlights changes made to the original version. You should upload this as a separate file labeled 'Revised Manuscript with Track Changes'.
- An unmarked version of your revised paper without tracked changes. You should upload this as a separate file labeled 'Manuscript'.

If you would like to make changes to your financial disclosure, please include your updated statement in your cover letter. Guidelines for resubmitting your figure files are available below the reviewer comments at the end of this letter.

If applicable, we recommend that you deposit your laboratory protocols in protocols.io to enhance the reproducibility of your results. Protocols.io assigns your protocol its own identifier (DOI) so that it can be cited independently in the future. For instructions see: https://journals.plos.org/plosone/s/submission-guidelines#loc-laboratory-protocols. Additionally, PLOS ONE offers an option for publishing peer-reviewed Lab Protocol articles, which describe protocols hosted on protocols.io. Read more information on sharing protocols at https://plos.org/protocols?utm_medium=editorial-email&utm_source=authorletters&utm_campaign=protocols.

As the corresponding author, your ORCID iD is verified in the submission system and will appear in the published article. PLOS supports the use of ORCID, and we encourage all coauthors to register for an ORCID iD and use it as well. Please encourage your coauthors to verify their ORCID iD within the submission system before final acceptance, as unverified ORCID iDs will not appear in the published article. *Only*  the individual author can complete the verification step; PLOS staff *cannot*  verify ORCID iDs on behalf of authors.

We look forward to receiving your revised manuscript.

Kind regards,

Helen Onyeaka, PhD

Academic Editor

PLOS One

Journal Requirements:

If the reviewer comments include a recommendation to cite specific previously published works, please review and evaluate these publications to determine whether they are relevant and should be cited. There is no requirement to cite these works unless the editor has indicated otherwise.

[Note: HTML markup is below. Please do not edit.]

Reviewers' comments:

Reviewer's Responses to Questions

**Comments to the Author**

Reviewer #3: (No Response)

Reviewer #4: (No Response)

**********

2. Is the manuscript technically sound, and do the data support the conclusions?

Reviewer #3: Partly

Reviewer #4: Partly

**********

3. Has the statistical analysis been performed appropriately and rigorously?

Reviewer #3: Yes

Reviewer #4: No

**********

4. Have the authors made all data underlying the findings in their manuscript fully available?

The PLOS Data policy

Reviewer #3: Yes

Reviewer #4: No

**********

5. Is the manuscript presented in an intelligible fashion and written in standard English?

Reviewer #3: No

Reviewer #4: No

**********

Reviewer #3: Overall, the revision shows clear and substantial improvement. Several important issues raised during the previous review have been addressed. In particular:

• The Introduction has been expanded and now provides a broader contextualization of COVID-19 impacts, gender inequalities, and the relevance of secondary cities:

• The manuscript now includes a conceptual illustration of the IBPA framework, which improves the clarity of the analytical approach.

• The Methods section has been strengthened, with clearer explanations of participant recruitment, data collection procedures, and analytical methods.

• Additional information regarding ethical approval and research rigor has been provided.

• The Discussion section has been improved and more explicitly connects the findings with IBPA principles such as power relations, equity, and intersecting identities.

• A distinct Conclusion section has been included, which summarizes the main findings and highlights the policy relevance of the study.

Despite these improvements, several points still require further attention before the manuscript can be considered for publication.

Conceptual positioning of IBPA

• Although the manuscript explains the principles of Intersectionality-Based Policy Analysis (IBPA), it still does not clearly articulate how IBPA provides additional explanatory value compared with other gender or policy analysis frameworks. A clearer justification of the analytical advantage of IBPA would strengthen the theoretical contribution of the study.

Explicit statement of the manuscript’s contribution

• The conclusion would benefit from a clearer articulation of the manuscript’s contribution to theory, methodology, and policy practice. Currently, these contributions remain somewhat implicit:

o The authors are encouraged to explicitly state:

o the theoretical contribution, particularly how the study advances intersectionality-informed policy analysis in the context of urban informal economies;

o the methodological contribution, namely the application of the Intersectionality-Based Policy Analysis (IBPA) framework combined with qualitative methods to examine emergency governance measures;

o the policy contribution, including how the findings inform more inclusive urban governance and crisis-response policies affecting women informal food vendors.

Policy implications

• The discussion and conclusion provide useful general recommendations regarding inclusive policymaking and engagement with women vendors. However, the policy implications remain relatively broad. The manuscript would benefit from more concrete and operational recommendations that could inform local governance or urban policy design.

o “Policy interventions should be developed with the involvement of marginalized groups to account for the existing diversities and respond to their unique needs.”. Suggestion: “The findings demonstrate the importance of integrating intersectionality-informed approaches into urban policy design to ensure that crisis response measures do not disproportionately marginalize women working in informal food systems.”

Language and editorial issues

• While the manuscript has improved stylistically, several minor grammatical and wording issues remain and should be corrected during the next revision.

o The term “IBPA fragment” is incorrect and unclear.

o “The analysis of the primary data from the KII, IDI, and FGD were also condected inductively and deductively guided by the IBPA..”

o “This would enhances the gender responsiveness of the interventions and minimizes inequalities…”

• A careful proofreading is recommended to ensure clarity and consistency.

In addition, the authors should consider also the following recommendations:

• The introduction should end with clear research objectives or research questions.

• In several sections, the Discussion reiterates findings already presented in the Results (e.g., the exclusion of women vendors from policy decision-making, experiences of harassment by enforcement officers, governance structure, and the blanket nature of COVID-19 policies):

o Results section: “Women vendors narrated their lived experiences of unfair treatment, extortion, assault and coercion from the enforcement authorities during the pandemic.” Discussion section: “In that regard, women vendors expressed their experiences with assault, harassment and extortion by law enforcement officers.”

o The Discussion would benefit from stronger analytical interpretation linking these findings to the IBPA framework, particularly regarding power relations, intersecting identities, and structural inequalities in urban governance!

• In Conclusion section:

o The policy recommendations in the conclusion are relevant but remain rather general (e.g., the call for ‘involvement of marginalized groups’ or ‘gender-responsive policy design’). The manuscript would benefit from more concrete and actionable policy suggestions, such as specific mechanisms for engaging women informal food vendors in policy processes or examples of gender-responsive emergency governance measures.

� “Policy interventions should be developed with the involvement of marginalized groups to account for the existing diversities and respond to their unique needs”. Suggestion: “Policy interventions should recognize the unique roles of women informal food vendors by incorporating targeted economic support measures, flexible market regulations, and inclusive communication strategies during public health emergencies.”

o “The Conclusion section currently includes extended discussion and policy interpretation The authors should avoid repeating discussion and focus on key takeaways:

� “While we acknowledge that the pandemic was a relatively new and urgent event that required immediate action through enforcement of public health orders inform of the control measures, we argue balancing of urgency with inclusive and equitable engagement of women by governments in public policy decision-making in times of emergencies is necessary to minimize the unintended impacts.” This sentence provides extended interpretation and argumentation, which is typical of the Discussion, is very long, and introduces new reasoning rather than summarizing findings. The conclusion would be stronger if it focused more concisely on summarizing the main findings and the study’s contribution, while limiting extended interpretation and policy discussion.” Suggestion: “Although the pandemic required urgent public health responses, the findings highlight the importance of inclusive policy processes that engage women informal food vendors in emergency decision-making.”

o The authors should highlight future research directions:

� “Future emergency policies as well as other government policies and interventions should be designed guided by a gendered lens to recognize their unique roles and needs.” The sentence focuses on policy recommendations, but the Conclusion does not mention directions for future research. Suggestion: “Future research could extend this analysis by conducting comparative studies across different secondary cities in order to examine how intersectional inequalities shape the experiences of informal food vendors under varying governance and policy contexts.”

Reviewer #4: The manuscript addresses a relevant problem and provides empirical information on the effects of COVID-19 policies on a population that has been insufficiently studied. The revised version shows a clear effort to respond to previous comments, particularly in the expansion of the introduction, the development of the IBPA framework, and the extension of the discussion.

Even so, the manuscript maintains problems related to the formulation of the study, internal consistency, and methodological clarity that should be addressed before it can be considered in its current form.

RESPONSE TO PREVIOUS COMMENTS

The expansion of the introduction and the incorporation of additional literature are considered addressed. The context is now more solid and better articulated.

The development of the IBPA framework is considered partially addressed. Its description was expanded and a figure was incorporated, but its use is not consistent throughout the manuscript.

The inclusion of explicit objectives or research questions was not addressed. The purpose of the study continues to be distributed across the narrative and is not presented in a direct manner in the introduction.

The discussion was expanded and includes more bibliographic support. However, in several points, the statements are not directly supported by the results, and therefore this aspect is considered partially addressed.

A limitations section was included, but the analysis of its implications is limited. This point is considered partially addressed.

Editorial revision was not addressed. Formal and structural issues remain that should have been corrected at this stage.

GENERAL COMMENTS

The manuscript presents relevant empirical material and addresses an important topic. However, several aspects related to methodological transparency, internal consistency, and analytical clarity need to be strengthened.

In its current form, the manuscript does not clearly define its research objective, does not consistently apply the IBPA framework, and does not provide sufficient detail to reconstruct the methodological procedure. In addition, there are structural issues in the results and discussion sections, including lack of separation between evidence and interpretation, repetition of arguments, and insufficient analytical development in some parts.

These issues do not invalidate the study, but they do require substantial revision.

SPECIFIC COMMENTS

TITLE

The title reflects the content of the manuscript. It includes the analytical approach, the context of COVID-19 control measures, the study population, and the empirical case.

Even so, it requires revision.

First, it is a long title. The number of elements included in a single structure reduces its clarity.

Second, it brings together two analytical levels, policy analysis and lived experiences, that are both part of the manuscript, but in the title, they appear accumulated, making it heavy.

Third, the expression “Secondary Cities” in plural does not fully correspond to the empirical scope of the study, which is based on a single case. Although the manuscript seeks to situate the analysis within that type of context, what is presented is a case study in Kisumu. The title therefore suggests a broader scope than what is actually developed.

ABSTRACT

The abstract presents the topic and the general approach of the study, but the way in which the results are formulated requires adjustment.

Statements regarding the effects of the policies are written in conclusive terms, when they correspond to interpretations derived from qualitative analysis. This should be adjusted to reflect the type of evidence that supports the findings.

The description of the qualitative component is not precise. The use of “focus group discussions (n = 40)” may be interpreted as referring to the number of groups rather than the number of participants, which introduces ambiguity.

The final part of the abstract emphasizes general implications, but it does not clearly define the specific contribution of the study. The abstract should state more precisely what the study contributes.

In addition, there is an imbalance between the presentation of results and the formulation of implications, with greater emphasis on the latter.

INTRODUCTION

The introduction presents a broader context than in the original version and improves the articulation of the problem.

However, it does not include an explicit statement of the objective or the research questions. The purpose of the study can be inferred from the text, but it is not presented directly, which affects the clarity of the manuscript.

The term “secondary cities” is used without an operational definition, which introduces ambiguity in the scope of the analysis.

The transition between the general contextualization of the problem and the specific delimitation of the study is not clearly established.

Conceptual Framework (IBPA)

The IBPA framework is developed in greater detail, but its application is not consistent.

The manuscript indicates that the “time and space” principle is not used, but it is later included in the description of the analysis. This inconsistency should be resolved.

In several parts of the manuscript, IBPA principles appear as already defined analytical categories, without showing clearly how they are constructed from the empirical material.

MATERIALS AND METHODS

The section presents an expansion compared to the original version; even so, it does not allow a precise reconstruction of the procedure followed in the study. This limits the evaluation of the study’s rigor and the coherence between design, data generation, and analysis.

The text combines two levels that should be distinguished: the description of the design and the justification of that design. When both are presented together, it becomes difficult to identify what was actually done and why it was done in that way. In a methodological section, the priority is to make the procedure traceable; justification may be included, but it should not interfere with the operational description.

Participant selection is not sufficiently delimited. The groups included in the study are mentioned, but their distribution across techniques (interviews, focus groups, and other components) is not specified. The number of participants per technique and per market is also not indicated. In addition, it is not clear whether different inclusion criteria were applied across techniques. Without this information, it is not possible to evaluate the internal consistency of the design or its adequacy for the type of analysis conducted.

The description of the sampling strategy includes statements that do not allow reconstruction of the selection logic. The indication that each market included “at least 12 participants distributed across techniques” does not clarify whether this refers to a minimum per technique, a cumulative total, or a distribution conditioned by fieldwork conditions. This point is relevant because it affects comparability across markets and the density of information generated by each technique.

There are gaps in basic procedural aspects. The manuscript does not describe how focus groups were organized (group size, criteria for composition, number of sessions), who conducted them, or under what conditions they were carried out. It also does not specify the language in which interviews and focus groups were conducted, nor how translation was handled and incorporated into the analysis. These elements directly affect the interpretation of qualitative data and should be clearly described.

The criterion used to determine that no new information was being generated is not defined. Saturation is mentioned, but the manuscript does not explain how it was established or at what point in the fieldwork it was considered to have been reached. Without this information, it is not possible to assess the sufficiency of the sample.

Regarding informed consent, the manuscript indicates that it was “written or verbal,” while other sections refer to written consent. This inconsistency should be resolved and presented in a uniform manner.

The document analysis component is mentioned as part of the design, but the procedure applied is not described. Criteria for selecting policies are indicated, but the manuscript does not explain how documents were analyzed, what categories were used, or how this component was integrated with interview and focus group data. Given that the study is presented as a policy analysis, this is a central element.

Finally, the relationship between the IBPA framework and the thematic analysis is not clearly described. The manuscript refers to both approaches, but it does not show how they were articulated in practice: whether IBPA guided coding, whether it was used to reorganize categories, or whether it was applied at a later interpretative stage. This lack of definition affects the methodological coherence of the study.

ANALYSIS

The manuscript mentions the participation of an independent researcher, but their role is not clearly defined.

Validation strategies are mentioned, including triangulation and related approaches, but they are not described in sufficient detail to understand how they were implemented.

RESULTS

The results are organized thematically and allow the reader to follow the general direction of the analysis; however, the section presents problems of structure, internal consistency, and differentiation between levels of information.

The section does not begin directly with the findings. The opening paragraphs return to methodological elements and describe again the organization of the analysis. This weakens the distinction between methods and results and delays the presentation of empirical evidence.

An inconsistency is observed in the characterization of the sample. The age range reported in the text does not match the one presented in the corresponding table. This type of discrepancy affects the internal coherence of the manuscript.

In several sections, there is no clear distinction between three levels of information: (i) evidence from analyzed documents, (ii) participant testimonies, and (iii) interpretation developed by the authors. This overlap makes it difficult to identify what corresponds to evidence and what corresponds to analysis.

Repetitions are observed across subthemes, particularly regarding exclusion of vendors, increased economic burden, and institutional control practices. These repetitions do not add new elements and fragment the argument, as the same idea appears in different sections without analytical progression.

The way some findings are introduced tends to be declarative. Repeated use of constructions such as “our analysis revealed” or similar expressions does not add information and can be replaced by direct presentation of the evidence.

A problem remains in table numbering, with duplication of “Table 1.”

The use of quotations requires adjustment. In some cases, the quotation repeats what has already been stated, without adding new information. In other cases, the quotation and the interpretation are not clearly separated, making it difficult to distinguish the participant’s voice from that of the authors.

Additionally, in some sections, results are presented together with immediate interpretation, without clearly delimiting the descriptive level. This is not necessarily incorrect, but it should be handled carefully to ensure that evidence is not subordinated to interpretation from the outset.

DISCUSSION

The discussion presents a clear expansion compared to the original version and shows an effort to connect the findings with additional literature and with the principles of the IBPA framework. However, the section still presents problems in the articulation between results, interpretation, and theoretical argument.

The first issue is analytical. IBPA principles are mentioned repeatedly, but it is not always clear how each of them emerges from the empirical material. In several points, principles such as power, equity, social justice, or intersecting categories are introduced as interpretative axes, but the manuscript does not consistently show the transition from the evidence presented in the results to the assignment of that evidence to those principles.

The second issue concerns argumentative sequence. In several parts, the discussion moves from findings to broader claims without sufficient intermediate development, particularly when linking results to normative or constitutional frameworks.

There is also repetition of arguments already presented in the results.

Comparative literature is incorporated, but the criteria for comparison are not always clearly established.

The concept of “compliance fatigue” is introduced without sufficient development.

Some statements adopt a stronger tone than what the qualitative design can support.

Language issues remain and require editorial revision.

CONCLUSIONS

The conclusions summarize the general topic of the study but do not clearly delimit its specific contribution.

The section moves toward policy recommendations without first clearly establishing the main findings and their analytical contribution.

There is no clear distinction between findings, interpretation, and implications.

Some statements go beyond what can be supported by the study design.

The contribution is present, but not clearly articulated.

ETHICS AND DATA AVAILABILITY

Data availability is not clearly described within the manuscript.

REFERENCES

Formatting is not consistent.

LANGUAGE

The manuscript is understandable, but it presents issues in writing and consistency.

A professional language revision is recommended.

**********

what does this mean?). If published, this will include your full peer review and any attached files.

If you choose “no”, your identity will remain anonymous but your review may still be made public.

**Do you want your identity to be public for this peer review?**  For information about this choice, including consent withdrawal, please see our Privacy Policy

Reviewer #3: No

Reviewer #4: **Yes:**  Javier Castro Rosas

**********

[NOTE: If reviewer comments were submitted as an attachment file, they will be attached to this email and accessible via the submission site. Please log into your account, locate the manuscript record, and check for the action link "View Attachments". If this link does not appear, there are no attachment files.]

To ensure your figures meet our technical requirements, please review our figure guidelines: https://journals.plos.org/plosone/s/figures

You may also use PLOS’s free figure tool, NAAS, to help you prepare publication quality figures: https://journals.plos.org/plosone/s/figures#loc-tools-for-figure-preparation.

NAAS will assess whether your figures meet our technical requirements by comparing each figure against our figure specifications.

# Author comment: Author response to Decision Letter 2

**DOI:** 10.1371/journal.pone.0359620.r004

15 May 2026

Response to Editor and reviewers’ comments

NB: Highlighted in yellow are the edited/revised sections in the paper.

NB: Highlighted in blue are the editor/reviewers comments and suggested edits

NB: Highlighted in gray are our responses to the suggestions/comments

Thank you for submitting your manuscript to PLOS ONE. After careful consideration, we feel that it has merit but does not fully meet PLOS ONE’s publication criteria as it currently stands. Therefore, we invite you to submit a revised version of the manuscript that addresses the points raised during the review process.

Well noted and addressed as shown below!

Reviewers' comments:

Reviewer #1: Reviewer’s Comments

Overall, the revision shows clear and substantial improvement. Several important issues raised during the previous review have been addressed. In particular:

o The Introduction has been expanded and now provides a broader contextualization of COVID-19 impacts, gender inequalities, and the relevance of secondary cities.

o The manuscript now includes a conceptual illustration of the IBPA framework, which improves the clarity of the analytical approach.

o The Methods section has been strengthened, with clearer explanations of participant recruitment, data collection procedures, and analytical methods.

o Additional information regarding ethical approval and research rigor has been provided.

o The Discussion section has been improved and more explicitly connects the findings with IBPA principles such as power relations, equity, and intersecting identities.

o A distinct Conclusion section has been included, which summarizes the main findings and highlights the policy relevance of the study.

Despite these improvements, several points still require further attention before the manuscript can be considered for publication.

Thank you for acknowledging the improvements made in the paper following the incorporation of the 1st round of reviewers’ comments. We appreciate your contribution to the work.

Conceptual positioning of IBPA

• Although the manuscript explains the principles of Intersectionality-Based Policy Analysis (IBPA), it still does not clearly articulate how IBPA provides additional explanatory value compared with other gender or policy analysis frameworks. A clearer justification of the analytical advantage of IBPA would strengthen the theoretical contribution of the study.

Thank you for this comment. We have extensively revised the introduction section to detail the application, relevance, and the rationale for the use of the IBPA framework in this research. A section of the introduction section now reads as follows:

……………

This study extends our understanding of the extent to which the perspectives of women business-owners were considered in the formulation and implementation of the pandemic control policies in secondary cities in the Global South. Understanding the gendered dimensions of the COVID-19 policies on women engaged in informal food businesses is essential for advocating for equitable policy frameworks that safeguard the food and nutritional security and economic stability of urban households, particularly, in times of emergencies. The study adopted the intersectionality-based policy analysis (IBPA) framework (Hankivsky et al., 2014) to investigate the influence of power dynamics and systemic structures on the marginalization of women food vendors within secondary cities during the COVID-19 pandemic. The IBPA framework facilitated an analysis that extended beyond gender as an individual level factor, enabling an exploration of how intersecting identities, policies and regulations – such as the COVID-19 control measures construct social locations and inequities marginalize specific groups, such as the women food vendors.

We utilized seven of the eight key principles of the IBPA framework – the principles of equity, power, intersecting categories, multi-level analysis, reflexivity, social justice, and diverse knowledges (Hankivsky et al., 2014). These seven IBPA framework principles (See Figure 1 below) guided the analysis of the COVID-19-related policies, key informant interviews, focus group discussions and in-depth interview data as well as the interpretation of the findings.

The principle of intersecting categories was employed to examine the interactions between various overlapping social identities – including gender, age, sexual orientation, marital status, educational attainment, cultural orientation, and socio-economic standing—and pandemic-related control strategies in intensifying the marginalization of female vendors. Furthermore, the analysis assessed whether these policies acknowledged the heterogeneous identities of informal food vendors and the specific requirements arising from such positionalities. By applying an intersectional perspective to policy review and qualitative narrative analysis, this study identified the ways in which social locations influenced women’s experiences of the pandemic policies and how inequalities predicated upon the design, dissemination, and interpretation of (Kanenberg & Leal, 2019).

In integrating the principle of power and multi-level analysis, we sought to highlight how overlapping power either facilitated inclusion or exacerbated systemic inequities for female vendors within the informal sector. In the Kenyan context—where healthcare service delivery is devolved to county and municipal authorities while legislative mandate remains centralized federal mandate, complex hierarchies of power emerge. Within such complex governance environments, the management of a public health crisis such as the COVID-19 pandemic necessitated a multisectoral approach characterized by deliberate engagement of the diverse partners, particularly the disadvantaged populations – such as the informal food vendors.

Furthermore, our analytical framework integrated the gender equity lens and the principle of social justice to examine the extent of gender inclusivity with the COVID-19 control measures, while considering the overlapping layers of power in the policy processes. By applying the principle of diverse knowledge, the framework enabled us to interrogate whether the voices of women in informal food vending were integrated in policy development and how power structures influenced the inclusion or exclusion of their diverse perspectives in policy decision making. Whilst studies in other jurisdictions indicated that governments neglected the gender-specific requirements of women during COVID-19 policy responses (Chitando, 2020), the application of the IBPA framework in this study enabled the advancement of the analysis beyond gender to also consider other intersecting identities, tenets of power dynamics, promotion of a social justice lens, and appreciation of diverse knowledge in policy analysis. We applied the principles while acknowledging the relevance of being self-reflexive. Notably, the IBPA principle of time and space, which analyses the dynamics of policy inequalities over time and across historical and geographical spaces, was excluded from this study. This is because the research focussed on the gendered COVID-19 policy dynamics at a specified time and place.

Explicit statement of the manuscript’s contribution

• The conclusion would benefit from a clearer articulation of the manuscript’s contribution to theory, methodology, and policy practice. Currently, these contributions remain somewhat implicit:

o The authors are encouraged to explicitly state:

o the theoretical contribution, particularly how the study advances intersectionality-informed policy analysis in the context of urban informal economies;

o the methodological contribution, namely the application of the Intersectionality-Based Policy Analysis (IBPA) framework combined with qualitative methods to examine emergency governance measures;

o the policy contribution, including how the findings inform more inclusive urban governance and crisis-response policies affecting women informal food vendors.

This has been addressed and integrated in the conclusion section which now includes specific policy interventions based on the discussed results.

Policy implications

• The discussion and conclusion provide useful general recommendations regarding inclusive policymaking and engagement with women vendors. However, the policy implications remain relatively broad. The manuscript would benefit from more concrete and operational recommendations that could inform local governance or urban policy design.

This has been addressed and integrated in the discussion and conclusion sections which now includes specific policy interventions based on the discussed results.

o “Policy interventions should be developed with the involvement of marginalized groups to account for the existing diversities and respond to their unique needs.”. Suggestion: “The findings demonstrate the importance of integrating intersectionality-informed approaches into urban policy design to ensure that crisis response measures do not disproportionately marginalize women working in informal food systems.”

This has been addressed and integrated in the discussion and conclusion sections which now includes specific policy interventions based on the discussed results.

Language and editorial issues

• While the manuscript has improved stylistically, several minor grammatical and wording issues remain and should be corrected during the next revision.

o The term “IBPA fragment” is incorrect and unclear.

Thank you! Corrected!

o “The analysis of the primary data from the KII, IDI, and FGD were also condected inductively and deductively guided by the IBPA..”

Thank you! Corrected!

o “This would enhance the gender responsiveness of the interventions and minimizes inequalities…”

Thank you! Corrected!

• A careful proofreading is recommended to ensure clarity and consistency.

Thank you! We have proof read and edited the entire manuscript for clarity!

In addition, the authors should consider also the following recommendations:

• The introduction should end with clear research objectives or research questions.

The introduction section has been extensively revised and a section that reflects the research question now reads as follows:

Kisumu City is the economic hub for western Kenya and the city’s economy is largely driven by informal trade (Opiyo et al., 2018). Over 60% of the informal businesses in the city are female owned, the majority of whom reside in informal settlements and belong to the Luo or Luhya speaking communities (Kiplagat et al., 2025). In these communities, gender roles are strictly delineated, with domestic responsibilities and caregiving traditionally assigned to women and girls, whose social standing is often diminished by prevailing patriarchal systems. The implementation of pandemic containment measures disproportionately affected these women-led businesses, as the closure of educational institutions and the increased need for medical care for family members imposed substantial additional caregiving burdens (Kiplagat et al., 2025; Odhiambo et al., 2025). Additionally, systemic power imbalances and the predominance of men in leadership roles resulted in the marginalization of female perspectives, particularly those from the informal food sector, in the formulation of pandemic response strategies.

This study extends our understanding of the extent to which the perspectives of women business-owners were considered in the formulation and implementation of the pandemic control policies in secondary cities in the Global South. Understanding the gendered dimensions of the COVID-19 policies on women engaged in informal food businesses is essential for advocating for equitable policy frameworks that safeguard the food and nutritional security and economic stability of urban households, particularly, in times of emergencies. The study adopted the intersectionality-based policy analysis (IBPA) framework (Hankivsky et al., 2014) to investigate the influence of power dynamics and systemic structures on the marginalization of women food vendors within secondary cities during the COVID-19 pandemic. The IBPA framework facilitated an analysis that extended beyond gender as an individual level factor, enabling an exploration of how intersecting identities, policies and regulations – such as the COVID-19 control measures construct social locations and inequities marginalize specific groups, such as the women food vendors.

• In several sections, the Discussion reiterates findings already presented in the Results (e.g., the exclusion of women vendors from policy decision-making, experiences of harassment by enforcement officers, governance structure, and the blanket nature of COVID-19 policies):

o Results section: “Women vendors narrated their lived experiences of unfair treatment, extortion, assault and coercion from the enforcement authorities during the pandemic.” Discussion section: “In that regard, women vendors expressed their experiences with assault, harassment and extortion by law enforcement officers.”

The discussion and the results section have been harmonized with the findings in the results section and the discussion having a more critical and analytical interpretation of the result/findings. See how the discussion now reads below.

o The Discussion would benefit from stronger analytical interpretation linking these findings to the IBPA framework, particularly regarding power relations, intersecting identities, and structural inequalities in urban governance!

We have extensively revised the discussion section and incorporated the suggested changes. The discussion now reads as follows:

Discussion

This study aimed to examine the sociocultural and gender disparities inherent in the formulation and implementation of COVID-19 mitigation policies and the subsequent experiences of women within the informal food vending sector. The findings reveal that the formulation and execution of these pandemic mitigation measures were characterized by a top-down approach that limited the engagement of women food vendors. Furthermore, the policies were fragmented by varying levels of power and power dynamics, reinforced prevailing inequities, and neglected the diverse intersecting identities of informal food vendors. Women vendors were systematically excluded from policy-making processes due to existing governance structures wherein health policies are formulated at the national level while service delivery is delegated to county governments, with most of these roles being male dominated. At the city and community levels, women vendors possess limited power and influence; they are frequently excluded from key decision-making processes due to systemic barriers that reinforce power hierarchies and shape policy outcomes. Our research identified several critical barriers contributing to the restricted engagement of women in the COVID-19 policy-making process within Kisumu. The factors included time constraints necessitated by the urgency of the crisis, sociocultural and gender inequities, a lack of accessible information regarding participation opportunities, and a reliance on technocratic expertise within the fields of health, governance, and policy. Such findings correspond with the observations of Martínez and Young (2022), who argued that the prioritization of technical experts in policy development by governments limited the participation of street vendors in decision-making processes. Furthermore, the pervasive societal culture of patriarchy served as a significant impediment to women involvement in political and leadership roles. Despite constitutional provisions for affirmative action and the establishment of County Women Representative positions in Kenya, policy discussions and community leadership remain predominantly male-dominated. These structural barriers reinforce gender hierarchies and the exclusion of women from decision-making at multiple levels of governance and society. Consequently, this lack of representation diminished the responsiveness of policies to the specific circumstances of women in the informal food vending sector, thereby exacerbating existing socio-economic disparities. (Coalition on Violence Against Women & Making It Work, 2023).

Narratives from the vendors emphasized this systematic exclusion, with participants frequently employing the expression "forced on us" to describe their marginalization and the lack of prior consultation regarding policy initiatives. These exclusionary practices appear to deviate from Article 174 (c) f the Constitution of Kenya, which mandates the enhancement of public participation in decision-making processes affecting the citizens (Laws of Kenya, 2013). Although scholarly evidence indicates that the political empowerment of women in pandemic-related policy-making lead to improved gender-sensitive health and economic outcomes (Abras et al., 2021; Huang & Lin, 2022), the approach in Kisumu City and many other cities in the Global South such as Rangpur in Bangladesh and Rusizi and Rubavu in Rwanda, remained highly centralized within the national Ministry of Health. The development and implementation of mitigation measures were restricted to technical experts and government officials, failing to incorporate community engagement strategies, gender-based considerations, or formal feedback mechanisms for the public, particularly for women operating in the informal food economy.

Furthermore, our study findings indicate that these policies reinforced existing inequities by failing to account for the diverse intersecting identities of marginalized groups, such as women food vendors in secondary cities. The policies did not contextualize the lived realities of these women with reference to their intersecting identities or prevailing sociocultural practices. Women vendors reported instances of inequitable treatment, including assault, harassment, and extortion by law enforcement officers. Similarly, (Solymári et al., 2022) observed that the brutality associated with COVID-19 policy enforcement in Kenyan informal settlements infringed upon fundamental human rights. This occurred against the backdrop of a male-dominated county enforcement team in Kisumu, which contributed to biased regulation enforcement (Loo et al., 2021). Such male dominance in enforcement predisposes women to violence and physical abuse at their places of business, posing severe health and safety risks and curtailing their agency to support their families. These experiences of harassment and the application of excessive force demonstrate how established power structures directly impacted the gendered experiences of women in the informal food sector, with some vendors reporting being compelled to pay bribes of up to five thousand shillings to avoid arrest.

Likewise, research in Nairobi by Wandera et al. (2023) documented that women in the informal workforce were subjected to physical abuse by police and municipal officers during the enforcement of curfews. This indicates a consistent pattern of COVID-19 policy impacts on women vendors across both primary and secondary urban centers. These findings are corroborated by Kiaka and colleagues who emphasized that the pandemic exacerbated instances of bribery and harassment in the Global South, particularly affecting informal vendors whose survival necessitated significant resilience. Exposure to such harassment and assault restricted women’s agency to facilitate urban household food provision and sustain their families during the pandemic. Consequently, it is argued that during periods of emergency, governments must implement gender-responsive governance systems to facilitate the engagement of women in the informal sector and ensure that policies address the specific requirements and experiences of this marginalized yet essential segment of the urban population.

Our study further provides evidence regarding the absence of an intersectional framework in the formulation of COVID-19 policies. Informal traders in Kisumu constitute a heterogeneous group characterized by diverse genders, ages, socio-economic statuses, cultural backgrounds, marital status, abilities, and religious affiliations, among other intersecting social identities. However, the pandemic mitigation strategies utilized a blanket policy approach that failed to account for the varied settings and specific identities of these vendors. This was also noted by Dutta et al. (2021) in India, where COVID-19 lockdown policies adopted a one-size-fits-all approach and overlooked existing gender differences.

These policy interventions did not consider the contextual factors such as the prevailing socio-economic situation in low- and middle-income countries (LMICs), and the pre-existing inequalities faced by women informal workers, including patriarchal structures and gender-based discrimination. Women vendors were already facing compounded challenges, including the precarity of their businesses and work, gender-based violence, discrimination, economic marginalization, and sexual harassment before the occurrence COVID-19 pandemic. The lack of gendered considerations in the policies worsened the prevailing inequalities creating an additional burden to the women vendors and influencing their resilience post-pandemic. While we acknowledge that the pandemic was a relatively new and urgent event that required immediate action through enforcement of public health orders inform of the control measures, we argue that governments must balance such urgency with inclusive and equitable engagement of women in public policy decision-making during emergencies to mitigate unintended negative impacts.

Women faced societal expectations to take up extra caregiving roles at the expense of paid work. Notably, findings from this study raise key questions regarding the value of unpaid household and caregiving duties undertaken by women. According to the United Nation’s Development Program-UN Women COVID-19 Global Gender Response Tracker, fewer than a third of the COVID-19 policy measures implemented in Kenya applied a gender perspective, with none accounting for unpaid care work (UN Women, 2021, 2022). Our findings extend this work by uncovering the experiences of women to the gender gaps in policies in localized settings of secondary cities.

Additionally, the COVID-19 policy measures introduced a significant number of new regulations with which traders were required to comply, potentially inducing "compliance fatigue" among vendors, considering the strict enforcement and use of force by the enforcement officers. This aligns with the findings of Martínez and Young (2022), which demonstrate that the exclusion of vulnerable groups in policy formulation and implementation undermines policy effectiveness and exacerbates marginalization. Such evidence may inform future policy decisions concerning the meaningful engagement of all stakeholders to achieve inclusive and more equitable policy outcomes, particularly during emergencies. As articulated by Cattapan et al., (2020), collaborative policy development should be guided by the principle of "nothing for us without us," which promotes the substantive participation of vulnerable groups in policy design and execution.

This study is not without limitations and it is important to recognize its limits. The choice of a cross-sectional study design meant that data was collected at a specific point in time, and in this case, between July 10, 2024 and October 15, 2024. The period during which data was collected was a few years after the COVID-19 pandemic, hence participants’ recollections could have omitted important details that could enrich the study findings. Additionally, this study only focused on one county out of the 47 counties in Kenya, and the outcome may not be generalized to the rest of the counties. However, the findings may be transferable to cities with similar characteristic like Kisumu City. Additionally, this study focused specifically on the informal food economy limiting the generalizability of the findings to other sectors of the informal economy. Lastly, the study relied on self reported data, which may be prone to social desirability, especially with reference to personalized experiences of the pandemic control measures, the impacts of the pandemic control measures on women-led informal food businesses, and the social and gender gaps in policies. Previous studies have adopted similar approaches where participants have shared their lived experiences with the COVID-19 control measures. Our findings are therefore comparable with those of past studies and this study makes important contributions to research on gendered dynamics of the informal food sector in secondary cities of the Global South.

• In Conclusion section:

o The policy recommendations in the conclusion are relevant but remain rather general (e.g., the call for ‘involvement of marginalized groups’ or ‘gender-responsive policy design’). The manuscript would benefit from more concrete and actionable policy suggestions, such as specific mechanisms for engaging women informal food vendors in policy processes or examples of gender-responsive emergency governance measures.

Thank you for this suggestion! It has been incorporated into the conclusion.

§ “Policy interventions should be developed with the involvement of marginalized groups to account for the existing diversities and respond to their unique needs”. Suggestion: “Policy interventions should recognize the unique roles of women informal food vendors by incorporating targeted economic support measures, flexible market regulations, and inclusive communication strategies during public health emergencies.”

Thank you for this suggestion! It has been incorporated into the conclusion

o “The Conclusion section currently includes extended discussion and policy interpretation The authors should avoid repeating discussion and focus on key takeaways:

§ “While we acknowledge that the pandemic was a relatively new and urgent event that required immediate action through enforcement of public health orders inform of the control measures, we argue balancing of urgency with inclusive and equitable engagement of women by governments in public policy decision-making in times of emergencies is necessary to minimize the unintended impacts.” This sentence provides extended interpretation and argumentation, which is typical of the Discussion, is very long, and introduces new reasoning rather than summarizing findings. The conclusion would be stronger if it focused more concisely on summarizing the main findings and the study’s contribution, while limiting extended interpretation and policy discussion.” Suggestion: “Although the pandemic required urgent public health responses, the findings highlight the importance of inclusive policy processes that engage women informal food vendors in emergency decision-making.”

Thank you for this suggestion! It has been incorporated into the conclusion

o The authors should highlight future research directions:

§ “Future emergency policies as well as other government policies and interventions should be designed guided by a gendered lens to recognize their unique roles and needs.” The sentence focuses on policy recommendations, but the Conclusion does not mention directions for future research. Suggestion: “Future research could extend this analysis by conducting comparative studies across different secondary cities in order to examine how intersectional inequalities shape the experiences of informal food vendors under varying governance and policy contexts.”

Thank you for this suggestion! This has been integrated into to the conclusion section which now reads as follows:

Conclusions

In conclusion, this research possesses significant scholarly and practical relevance, offering both methodological and substantive contributions. By utilizing an IBPA framework to examine urban informal economies and the experiences of informal food vendors, the study extends policy discourse beyond a singular focus on gender. It underscores the critical role of intersecting identities in shaping the lived experiences of women vendors and facilitates the integration of intersectional policy analysis within health research.

Substantively, this research indicates that pandemic containment measures were developed and enforced utilizing a top-down methodology, which effectively marginalized female informal food vendors and failed to account for their unique needs and diverse intersecting identities. Additionally, pre-existing power dynamics and gender-based vulnerabilities were reinforced and exacerbated by the regulatory measures, including curfews, stay-at-home mandates, and physical distancing protocols.

To address this gap, policy interventions should recognize the unique roles of women informal food vendors by incorporating targeted economic support measures, flexible market regulations, and inclusive communication strategies during public health emergencies. While the global health crisis necessitated immediate public health interventions, these findings emphasize the imperative for inclusive policy-making processes that incorporate female informal food vendors into the policy formulation and implementation processes. For example, governments should adopt gender-responsive emergency governance measures that intentionally solicit and integrate the perspectives and lived realities of informal vendors. Furthermore, governments should institutionalize mechanisms for the meaningful participation of informal vendors in policy design through recognized market associations and community-based collectives. To ensure efficacy, policy interventions must be designed in collaboration with marginalized groups to accommodate social diversity and respond to localized needs. In the context of female informal food vendors, future regulatory frameworks and emergency directives should adopt a gendered perspective to formally recognize their unique contributions and safeguard their role in maintaining urban food security.

Such integration would enhance the gender responsiveness of policy interventions and mitigate structural inequalities. Future scholarly inquiries should expand upon this analysis by conducting comparative studies across various secondary cities to evaluate how intersectional inequalities shape the experiences of informal food vendors under disparate governance and policy frameworks.

Responses to Reviewer #2

The manuscript addresses a relevant problem and provides empirical information on the effects of COVID-19 policies on a population that has been insufficiently studied. The revised version shows a clear effort to respond to previous comments, particularly in the expansion of the introduction, the development of the IBPA framework, and the extension of the discussion.

Even so, the manuscript maintains problems related to the formulation of the study, internal consistency, and methodological clarity that should be addressed before it can be considered in its current form.

Thank you!

RESPONSE TO PREVIOUS COMMENTS

The expansion of the introduction and the incorporation of additional literature are considered addressed. The context is now more solid and better articulated.

The development of the IBPA framework is considered partially addressed. Its description was expanded and a figure was incorporated, but its use is not consistent throughout the manuscript.

Thank you for this observation! The IBPA framework and its use throughout the paper has been reviewed and areas of inconsistency revised.

…….

This study extends our understanding of the extent to which the perspectives of women business-owners were considered in the formulation and implementation of the pandemic control policies in secondary cities in the Global South. Understanding the gendered dimensions of the COVID-19 policies on women engaged in informal food businesses is essential for advocating for equitable policy frameworks that safeguard the food and nutritional security and economic stability of urban households, particularly, in times of emergencies. The study adopted the intersectionality-based policy analysis (IBPA) framework (Hankivsky et al., 2014) to investigate the influence of power dynamics and systemic structures on the marginalization of women food vendors within secondary cities during the COVID-19 pandemic. The IBPA framework facilitated an analysis that extended beyond gender as an individual level factor, enabling an exploration of how intersecting identities, policies and regulations – such as the COVID-19 control measures construct social locations and inequities marginalize specific groups, such as the women food vendors.

We utilized seven of the eight key principles of the IBPA framework – the principles of equity, power, intersecting categories, multi-level analysis, reflexivity, social justice, and diverse knowledges (Hankivsky et al., 2014). These seven IBPA framework principles (See Figure 1 below) guided the analysis of the COVID-19-related policies, key informant interviews, focus group discussions and in-depth interview data as well as the interpretation of the findings.

The principle of intersecting categories was employed to examine the interactions between various overlapping social identities – including gender, age, sexual orientation, marital status, educational attainment, cultural orientation, and socio-economic standing—and pandemic-related control strategies in intensifying the marginalization of female vendors. Furthermore, the analysis assessed whether these policies acknowledged the heterogeneous identities of informal food vendors and the specific requirements arising from such positionalities. By applying an intersectional perspective to policy review and qualitative narrative analysis, this study identified the ways in which social locations influenced women’s experiences of the pandemic policies and how inequalities predicated upon the design, dissemination, and interpretation of (Kanenberg & Leal, 2019).

The inclusion of explicit objectives or research questions was not addressed. The purpose of the study continues to be distributed across the narrative and is not presented in a direct manner in the introduction.

Thank you pointing this out! We have revised the introduction to accurately reflect the aim/objective of the paper. A section of the Introduction now reads as follows:

The discussion was expanded and includes more bibliographic support. However, in several points, the statements are not directly supported by the results, and therefore this aspect is considered partially addressed.

The discussion section has been extensively revised and the suggestions integrated to ensure that the claims are supported by the presented results. The Discussion Section now reads as follows:

Discussion

This study aimed to examine the sociocultural and gender disparities inherent in the formulation and implementation of COVID-19 mitigation policies and the subsequent experiences of women within the informal food vending sector. The findings reveal that the formulation and execution of these pandemic mitigation measures were characterized by a top-down approach that limited the engagement of women food vendors. Furthermore, the policies were fragmented by varying levels of power and power dynamics, reinforced prevailing inequities, and neglected the diverse intersecting identities of informal food vendors. Women vendors were systematically excluded from policy-making processes due to existing governance structures wherein health policies are formulated at the national level while service delivery is delegated to county governments, with most of these roles being male dominated. At the city and community levels, women vendors possess limited power and influence; they are frequently excluded from key decision-making processes due to systemic barriers that reinforce power hierarchies and shape policy outcomes. Our research identified several critical barriers contributing to the restricted engagement of women in the COVID-19 policy-making process within Kisumu. The factors included time constraints necessitated by the urgency of the crisis, sociocultural and gender inequities, a lack of accessible information regarding participation opportunities, and a reliance on technocratic expertise within the fields of health, governance, and policy. Such findings correspond with the observations of Martínez and Young (2022), who argued that the prioritization of technical experts in policy development by governments limited the participation of street vendors in decision-making processes. Furthermore, the pervasive societal culture of patriarchy served as a significant impediment to women involvement in political and leadership roles. Despite constitutional provisions for affirmative action and the establishment of County Women Representative positions in Kenya, policy discussions and community leadership remain predominantly male-dominated. These structural barriers reinforce gender hierarchies and the exclusion of women from decision-making at multiple levels of governance and society. Consequently, this lack of representation diminished the responsiveness of policies to the specific circumstances of women in the informal food vending sector, thereby exacerbating existing socio-economic disparities. (Coalition on Violence Against Women & Making It Work, 2023).

Narratives from the vendors emphasized this systematic exclusion, with participants frequently employing the expression "forced on us" to describe their marginalization and the lack of prior consultation regarding policy initiatives. These exclusionary practices appear to deviate from Article 174 (c) f the Constitution of Kenya, which mandates the enhancement of public participation in decision-making processes affecting the citizens (Laws of Kenya, 2013). Although scholarly evidence indicates that the political empowerment of women in pandemic-related policy-making lead to improved gender-sensitive health and economic outcomes (Abras et al., 2021; Huang & Lin, 2022), the approach in Kisumu City and many other cities in the Global South such as Rangpur in Bangladesh and Rusizi and Rubavu in Rwanda, remained highly centralized within the national Ministry of Health. The development and implementation of mitigation measures were restricted to technical experts and government officials, failing to incorporate community engagement strategies, gender-based considerations, or formal feedback mechanisms for the public, particularly for women operating in the informal food economy.

Furthermore, our study findings indicate that these policies reinforced existing inequities by failing to account for the diverse intersecting identities of marginalized groups, such as women food vendors in secondary cities. The policies did not contextualize the lived realities of these women with reference to their intersecting identities or prevailing sociocultural practices. Women vendors reported instances of inequitable treatment, including assault, harassment, and extortion by law enforcement officers. Similarly, (Solymári et al., 2022) observed that the brutality associated with COVID-19 policy enforcement in Kenyan informal settlements infringed upon fundamental human rights. This occurred against the backdrop of a male-dominated county enforcement team in Kisumu, which contributed to biased regulation enforcement (Loo et al., 2021). Such male dominance in enforcement predisposes women to violence and physical abuse at their places of business, posing severe health and safety risks and curtailing their agency to support their families. These experiences of harassment and the application of excessive force demonstrate how established power structures directly impacted the gendered experiences of women in the informal food sector, with some vendors reporting being compelled to pay bribes of up to five thousand shillings to avoid arrest.

Likewise, research in Nairobi by Wandera et al. (2023) documented that women in the informal workforce were subjected to physical abuse by police and municipal officers during the enforcement of curfews. This indicates a consistent pattern of COVID-19 policy impacts on women vendors across both primary and secondary urban centers. These findings are corroborated by Kiaka and colleagues who emphasized that the pandemic exacerbated instances of bribery and harassment in the Global South, particularly affecting informal vendors whose survival necessitated significant resilience. Exposure to such harassment and assault restricted women’s agency to facilitate urban household food provision and sustain their families during the pandemic. Consequently, it is argued that during periods of emergency, governments must implement gender-responsive governance systems to facilitate the engagement of women in the informal sector and ensure that policies address the specific requirements and experiences of this marginalized yet essential segment of the urban population.

Our study further provides evidence regarding the absence of an intersectional framework in the formulation of COVID-19 policies. Informal traders in Kisumu constitute a heterogeneous group characterized by diverse genders, ages, socio-economic statuses, cultural backgrounds, marital status, abilities, and religious affiliations, among other intersecting social identities. However, the pandemic mitigation strategies utilized a blanket policy approach that failed to account for the varied settings and specific identities of these vendors. This was also noted by Dutta et al. (2021) in India, where COVID-19 lockdown policies adopted a one-size-fits-all approach and overlooked existing gender differences.

These policy interventions did not consider the contextual factors such as the prevailing socio-economic situation in low- and middle-income countries (LMICs), and the pre-existing inequalities faced by women informal workers, including patriarchal structures and gender-based discrimination. Women vendors were already facing compounded challenges, including the precarity of their businesses and work, gender-based violence, discrimination, economic marginalization, and sexual harassment before the occurrence COVID-19 pandemic. The lack of gendered considerations in the policies worsened the prevailing inequalities creating an additional burden to the women vendors and influencing their resilience post-pandemic. While we acknowledge that the pandemic was a relatively new and urgent event that required immediate action through enforcement of public health orders inform of the control measures, we argue that governments must balance such urgency with inclusive and equitable engagement of women in public policy decision-making during emergencies to mitigate unintended negative impacts.

Women faced societal expectations to take up extra caregiving roles at the expense of paid work. Notably, findings from this study raise key questions regarding the value of unpaid household and caregiving duties undertaken by women. According to the United Nation’s Development Program-UN Women COVID-19 Global Gender Response Tracker, fewer than a third of the COVID-19 policy measures implemented in Kenya applied a gender perspective, with none accounting for unpaid care work (UN Women, 2021, 2022). Our findings extend this work by uncovering the experiences of women to the gender gaps in policies in localized settings of secondary cities.

Additionally, the COVID-19 policy measures introduced a significant number of new regulations with which traders were required to comply, potentially inducing "compliance fatigue" among vendors, considering the strict enforcement and use of force by the enforcement officers. This aligns with the findings of Martínez and Young (2022), which demonstrate that the exclusion of vulnerable groups in policy formulation and implementation undermines policy effectiveness and exacerbates marginalization. Such evidence may inform future policy decisions concerning the meaningful engagement of all stakeholders to achieve inclusive and more equitable policy outcomes, particularly during emergencies. As articulated by Cattapan et al., (2020), collaborative policy development should be guided by the principle of "nothing for us without us," which promotes the substantive participation of vulnerable groups in policy design and execution.

This study is not without limitations and it is important to recognize its limits. The choice of a cross-sectional study design meant that data was collected at a specific point in time, and in this case, between July 10, 2024 and October 15, 2024. The period during which data was collected was a few years after the COVID-19 pandemic, hence participants’ recollections could have omitted important details that could enrich the study findings. Additionally, this study only focused on one county out of the 47 counties in Kenya, and the outcome may not be generalized to the rest of the counties. However, the findings may be transferable to cities with similar characteristic like Kisumu City. Additionally, this study focused specifically on the informal food economy limiting the generalizability of the findings to other sectors of the informal economy. Lastly, the study relied on self reported data, which may be prone to social desirability, especially with reference to personalized experiences of the pandemic control measures, the impacts of the pandemic control measures on women-led informal food businesses, and the social and gender gaps in policies. Previous studies have adopted similar approaches where participants have shared their lived experiences with the COVID-19 control measures. Our findings are therefore comparable with those of past studies and this study makes important contributions to research on gendered dynamics of the informal food sector in secondary cities of the Global South.

A limitations section was included, but the analysis of its implications is limited. This point is considered partially addressed. Editorial revision was not addressed. Formal and structural issues remain that should have been corrected at this stage.

The paper has been reviewed for any editorial errors and for better clarity of the key messages being communicated.

GENERAL COMMENTS

The manuscript presents relevant empirical material and addresses an important topic. However, several aspects related to methodological transparency, internal consistency, and analytical clarity need to be strengthened.

In its current form, the manuscript does not clearly define its research objective, does not consistently apply the IBPA framework, and does not provide sufficient detail to reconstruct the methodological procedure. In addition, there are structural issues in the results and discussion sections, including lack of separation between evidence and interpretation, repetition of arguments, and insufficient analytical development in some parts.

These issues do not invalidate the study, but they do require substantial revision.

Thanks for these valuable comments. The paper has been extensively revised and the areas of limitation/weaknesses pointed addressed.

SPECIFIC COMMENTS

TITLE

The title reflects the content of the manuscript. It includes the analytical approach, the context of COVID-19 control measures, the study population, and the empirical case.

Even so, it requires revision.

The title has been revised and made more concise to allow for clarity. This is the new title to the manuscript:

An Intersectionality-Based Policy Analysis of COVID-19 Measures and Women Informal Food Vendors in Kisumu, Kenya

First, it is a long title. The number of elements included in a single structure reduces its clarity.

Second, it brings together two analytical levels, policy analysis and lived experiences, that are both part of the manuscript, but in the title, they appear accumulated, making it heavy.

Third, the expression “Secondary Cities” in plural does not fully correspond to the empirical scope of the study, which is based on a single case. Although the manuscript seeks to situate the analysis within that type of context, what is presented is a case study in Kisumu. The title therefore suggests a broader scope than what is actually developed.

Well noted and revised as indicated above! Thank you!

ABSTRACT

The abstract presents the topic and the general approach of the study, but the way in which the results are formulated requires adjustment.

Statements regarding the effects of the policies are written in conclusive terms, when they correspond to interpretations derived from qualitative analysis. This should be adjusted to reflect the type of evidence that supports the findings.

The description of the qualitative component is not precise. The use of “focus group discussions (n = 40)” may be interpreted as referring to the number of groups rather than the number of participants, which introduces ambiguity.

The final part of the abstract emphasizes general implications, but it does not clearly define the specific contribution of the study. The abstract should state more precisely what the study contributes.

In addition, there is an imbalance between the presentation of results and the formulation of implications, with greater emphasis on the latter.

The abstract has been extensively revised and the raised points incorporated. We have also ensured consistency with the findings, discussion, and study implication. The Abstract now reads as follows:

Abstract

Introduction: Women engaged in informal food vending in Kisumu faced precarious occupational conditions that were intensified by the COVID-19 pandemic. Notwithstanding their critical contribution to urban household food security, these vendors were marginalized from policy-making processes, which were predominantly restricted to technical experts and national government officials.

Methods: Utilizing the Intersectionality-Based Policy Analysis (IBPA) framework, this research investigated engagement of women informal food venders and the disparities within COVID-19 containment and control policies. Conducted from July 10, 2024 to October 15, 2024, the study employed qualitative methodologies, including key informant interviews (n=20), in-depth interviews (n=20), and four focus group discussions with ten participants each (n=40) to document the experiences of women vendors regarding pandemic-related regulations. Moreover, four policy documents were purposively selected and reviewed. Participants were selected through purposive sampling in partnership with Pamoja Community Based Organisation, and thematic analysis was performed according to IBPA principles.

Results: The findings indicate that the exclusion of women vendors from decision-making yielded gender-insensitive policies that exacerbated existing inequalities. The policy formulation process failed to account for the diverse and intersecting social identities of these vendors. Additionally, fragmented policy implementation subjected women to physical assault, harassment, and extortion, thereby increasing gender-based vulnerabilities.

Discussion: In the event of future emergencies, it is imperative that governments establish inclusive, gender-responsive policies that prioritize the perspectives of informal food vendors to ensure sustainable urban food systems. Governments should develop inclusive and gender-sensitive policies while centering the voices of women informal food vendors for a sustainable urban food system. This research advances the integration of intersectional policy analysis within health research, championing principles of equity and social justice to enhance the participation of marginalized groups in the development and execution of health-oriented regulations, particularly in emergency situations.

Keywords: Intersectionality-Based Policy Analysis; Gender-responsive policies; COVID-19 Control Measure; Informal Food Vendors; Secondary Cities; Global South

INTRODUCTION

The introduction presents a broader context than in the original version and improves the articulation of the problem.

However, it does not include an explicit statement of the objective or the research questions. The purpose of the study can be inferred from the text, but it is not presented directly, which affects the clarity of the manuscript.

The term “secondary cities” is used without an operational definition, which introduces ambiguity in the scope of the analysis.

The transition between the general contextualization of the problem and the specific delimitation of the study is not clearly established.

Conceptual Framework (IBPA)

The IBPA framework is developed in greater detail, but its application is not consistent.

The manuscript indicates that the “time and space” principle is not used, but it is later included in the description of the analysis. This inconsistency should be resolved.

In several parts of the manuscript, IBPA principles appear as already defined analytical categories, without showing clearly how they are constructed from the empirical material.

The Introduction section has been extensively revised and the suggested comments integrated. The IBPA framework has been discussed more extensively to show its application in the study and the term “secondary cities” defined. A clear aim/objective of the paper is also included in the introduction section. The introduction now reads as follows:

Introduction

In early 2020, the World Health Organization (WHO) characterized COVID-19 a global pandemic requiring immediate public health action in all fronts (World Health Organization (WHO), 2020). Governments across the world, subsequently, imposed mandatory containment measures such as lockdowns and curfews (Koh, 2020). Safety protocols such as the use of face masks, handwashing, sanitizing, and physical distancing were also introduced to curb the spread of the coronavirus (Mwakisha et al., 2024). These control measures coupled with the effects of the diseases – COVID-19 had devastating effects on global trade and markets, which affected both the formal and informal economies across the world (Alon et al., 2022; Alon et al., 2020; Mishra & Rampal, 2020). Within the informal sector, the control policies triggered events that shaped social and economic outcomes differently for men and women engaged in the sector (Kabeer et al., 2021); hence, exposing and exacerbating the existing socioeconomic and cultural inequalities, especially in urban centres (Finn, 2023; Finn & Kobayashi, 2020; Odhiambo et al., 2025; Onyango et al., 2021; Sharifi & Khavarian-Garmsir, 2020; Ukala et al., 2020).

In cities in the global South, women disproportionately bore the burden of the pandemic and its control measures, with women food vendors experiencing an array of systemic challenges (Afifu et al., 2021; Kiplagat et al., 2025; Onyango et al., 2021; Onyango et al., 2023). These challenges encompassed reduced market hours, restricted movement, disruption of food supply chains, increased household and caregiving responsibilities, alongside significant income loss and heightened food insecurity (Afifu et al., 2021; Kimani-Murage et al., 2022). The impact was particularly severe as many women-led informal food businesses that rely on robust social interaction and physical proximity their customers on the streets – elements that were curtailed by the pandemic control measures (Finn & Kobayashi, 2020). Such measures exacerbated extant inequalities, largely due to a failure to integrate the specific socio-cultural and economic exigencies faced by women into policy frameworks (Odhiambo et al., 2025; Ukala et al., 2020).

Furthermore, the absence of gender-sensitive considerations in policy initiatives introduced unique complications for women during the pandemic (UN Women, 2024). Women informal food vendors faced substantial barriers in securing financial assistance from both governmental and private financial institutions (Donga & Chimucheka, 2024; Sinyolo et al., 2022). In the African context, these difficulties were intensified by entrenched socio-cultural norms and patriarchal structures, which frequently undermine women's autonomy and ownership of assets required as collateral for credit access (Fletschner & Kenney, 2014; Kiplagat et al., 2025).

Across the majority of the cities within the Global South —and specifically across secondary cities, women are over-represented in the informal economic sector, with a heavy concentration in the food industry (Donga & Chimucheka, 2024). These secondary cities function as critical regional, administrative, and economic focal points and are characterized by a strong urban-rural connection and rapid urbanization (Pendras & Williams, 2021). Within the context of Kenya, Kisumu City stands as a major secondary city where women constitute approximately 60% of the total informal traders, primarily engaged in food vending (Kisumu, 2020; Opiyo et al., 2018). These women-led businesses play a key role in bolstering urban household food needs and livelihoods (Kiplagat et al., 2025; Odhiambo et al., 2025). Yet, pandemic-related policy frameworks failed to account for the distinct vulnerabilities of women in these informal food sectors. This neglect led to an escalation in gender-based and intimate partner violence, while simultaneously undermining the capacity of women informal enterprises to contribute to the social and economic fabric of the city (Afifu et al., 2021; John et al., 2021; Roever & Skinner, 2016; Smith, 2023). Simultaneously, increased unpaid care work and household responsibilities impeded women’s full engagement in paid work (Wenham et al., 2020).

However, limited studies have explored gender-specific gaps in COVID-19 control policies in secondary cities in the Global South. In particular, the impacts of the control measures on women-led informal food businesses in secondary cities remains an underexplored research area (Malou, 2024). Most existing studies have focused on the broader informal sector in major metropolitan cities of the Global South, such as Nairobi, Kampala, Karachi, West Bengal, and Johannesburg (Afifu et al., 2021; Anwar et al., 2023; Mondal & Chakraborty, 2022; Pinchoff et al., 2021; Rwafa-Ponela et al., 2022; Suubi et al., 2022). Policy analyses on COVID-19 in Durban, Nairobi and Kampala indicated that women operating within the urban informal economy were systematically excluded from COVID-19 policy processes (Afifu et al., 2021; Skinner et al., 2021; Suubi et al., 2022). Furthermore, intersectional analyses in Karachi revealed that lockdown mandates exacerbated gendered inequalities, manifesting in heightened domestic violence and intensified caregiving responsibilities, even as women maintained their critical role in household provisioning (Anwar et al., 2023). In context of West Benghal, Mondal and Chackraborty (2022) posited that women informal workers had limited influence on policy decisions during the pandemic. As emphasized by Martínez and Young (2022), there exists a critical dearth of research centred on secondary cities around the impacts of the pandemic of women in the informal food sector.

Secondary cities have a distinct role in fostering social, economic, and cultural connections between the rural and urban communities. These cities are often characterized by informality and are marginalized, especially during emergencies (Kiplagat et al., 2025). In the case of Kisumu City, COVID-19 policies were formulated at the national government level in Nairobi, a primary city in Kenya. Such centralized governance effectively excluded the lived realities of vulnerable populations in secondary cities, particularly, women.

Kisumu City is the economic hub for western Kenya and the city’s economy is largely driven by informal trade (Opiyo et al., 2018). Over 60% of the informal businesses in the city are female owned, the majority of whom reside in informal settlements and belong to the Luo or Luhya speaking communities (Kiplagat et al., 2025). In these communities, gender roles are strictly delineated, with domestic responsibilities and caregiving traditionally assigned to women and girls, whose social standing is often diminished by prevailing patriarchal systems. The implementation of pandemic containment measures disproportionately affected these women-led businesses, as the closure of educational institutions and the increased need for medical care for family members imposed substantial additional caregiving burdens (Kiplagat et al., 2025; Odhiambo et al., 2025). Additionally, systemic power imbalances and the predominance of men in leadership roles resulted in the marginalization of female perspectives, particularly those from the informal food sector, in the formulation of pandemic response strategies.

This study extends our understanding of the extent to which the perspectives of women business-owners were considered in the formulation and implementation of the pandemic control policies in secondary cities in the Global South. Understanding the gendered dimensions of the COVID-19 policies on women engaged in informal food businesses is essential for advocating for equitable policy frameworks that safeguard the food and nutritional security and economic stability of urban households, particularly, in times of emergencies. The study adopted the intersectionality-based policy analysis (IBPA) framework (Hankivsky et al., 2014) to investigate the influence of power dynamics and systemic structures on the marginalization of women food vendors within secondary cities during the COVID-19 pandemic. The IBPA framework facilitated an analysis that extended beyond gender as an individual level factor, enabling an exploration of how intersecting identities, policies and regulations – such as the COVID-19 control measures construct social locations and inequities marginalize specific groups, such as the women food vendors.

We utilized seven of the eight key principles of the IBPA framework – the principles of equity, power, intersecting categories, multi-level analysis, reflexivity, social justice, and diverse knowledges (Hankivsky et al., 2014). These seven IBPA framework principles (See Figure 1 below) guided the analysis of the COVID-19-related policies, key informant interviews, focus group discussions and in-depth interview data as well as the interpretation of the findings.

The principle of intersecting categories was employed to examine the interactions between various overlapping social identities – including gender, age, sexual orientation, marital status, educational attainment, cultural orientation, and socio-economic standing—and pandemic-related control strategies in intensifying the marginalization of female vendors. Furthermore, the analysis assessed whether these policies acknowledged the heterogeneous identities of informal food vendors and the specific requirements arising from such positionalities. By applying an intersectional perspective to policy review and qualitative narrative analysis, this study identified the ways in which social locations influenced women’s experiences of the pandemic policies and how inequalities predicated upon the design, dissemination, and interpretation of (Kanenberg & Leal, 2019).

In integrating the principle of power and multi-level analysis, we sought to highlight how overlapping power either facilitated inclusion or exacerbated systemic inequities for female vendors within the informal sector. In the Kenyan context—where healthcare service delivery is devolved to county and municipal authorities while legislative mandate remains centralized federal mandate, complex hierarchies of power emerge. Within such complex governance environments, the management of a public health crisis such as the COVID-19 pandemic necessitated a multisectoral approach characterized by deliberate engagement of the diverse partners, particularly the disadvantaged populations – such as the informal food vendors.

Furthermore, our analytical framework integrated the gender equity lens and the principle of social justice to examine the extent of gender inclusivity with the COVID-19 control measures, while considering the overlapping layers of power in the policy processes. By applying the principle of diverse knowledge, the framework enabled us to interrogate whether the voices of women in informal food vending were integrated in policy development and how power structures influenced the inclusion or exclusion of their diverse perspectives in policy decision making. Whilst studies in other jurisdictions indicated that governments neglected the gender-specific requirements of women during COVID-19 policy responses (Chitando, 2020), the application of the IBPA framework in this study enabled the advancement of the analysis beyond gender to also consider other intersecting identities, tenets of power dynamics, promotion of a social justice lens, and appreciation of diverse knowledge in policy analysis. We applied the principles while acknowledging the relevance of being self-reflexive. Notably, the IBPA principle of time and space, which analyses the dynamics of policy inequalities over time and across historical and geographical spaces, was excluded from this study. This is because the research focussed on the gendered COVID-19 policy dynamics at a specified time and place.

<<<<Figure 1: The conceptual framework for the IBPA guiding principles for analysis of COVID-19 control policies & the experiences of female informal food vendors>>>

MATERIALS AND METHODS

The section presents an expansion compared to the original version; even so, it does not allow a precise reconstruction of the procedure followed in the study. This limits the evaluation of the study’s rigor and the coherence between design, data generation, and analysis.

The text combines two levels that should be distinguished: the description of the design and the justification of that design. When both are presented together, it becomes difficult to identify what was actually done and why it was done in that way. In a methodological section, the priority is to make the procedure traceable; justification may be included, but it should not interfere with the operational description.

Thank you for this observation. It has been considered in this revised version and the methodological section should now have more clear and detailed procedure that was adopted in this research.

Participant selection is not sufficiently delimited. The groups included in the study are mentioned, but their distribution across techniques (interviews, focus groups, and other components) is not specified. The number of participants per technique and per market is also not indicated. In addition, it is not clear whether different inclusion criteria were applied across techniques. Without this information, it is not possible to evaluate the internal consistency of the design or its adequacy for the type of analysis conducted.

This has been addressed and the exact number of participants across the 5 markets and in the different data collection points presented in Table 1 in the paper.

The description of the sampling strategy includes statements that do not allow reconstruction of the selection logic. The indication that each market included “at least 12 participants distributed across techniques” does not clarify whether this refers to a minimum per technique, a cumulative total, or a distribution conditioned by fieldwork conditions. This point is relevant because it affects comparability across markets and the density of information generated by each technique.

This has been addressed and the exact number of participants across the 5 markets and in the different data collection points presented in Table 1 in the paper.

There are gaps in basic procedural aspects. The manuscript does not describe how focus groups were organized (group size, criteria for composition, number of sessions), who conducted them, or under what conditions they were carried out. It also does not specify the language in which interviews and focus groups were conducted, nor how translation was handled and incorporated into the analysis. These elements directly affect the interpretation of qualitative data and should be clearly described.

Thank you for pointing this out. This has been addressed in the methods section. A section of the methods now reads as follows:

Primary data collection

Research assistants trained in qualitative research techniques and fluent in English, Swahili, and the local Luo dialect, conducted participant recruitment and facilitated information sessions to support primary data collection through face-to-face interviews. The choice of language used was determined by the participant’s preference with most key informants preferring English. Most of the IDI and FGD participants preferred Swahili and/or Luo. The interviews were audio-recorded with the consent of participants. Sessions conducted in English were transcribed verbatim, while those conducted in Swahili or Luo were translated into English as they were being transcribed by the research assistant that had facilitated the sessions. The resulting transcripts were subsequently reviewed by the research assistants and two authors (PMO & EOO), who possess fluency in all three languages, to ensure the accuracy of the translations and data transcriptions.

Semi-structured interview guides were developed and utilized to facilitate KIIs, IDIs, and FGDs. These instruments were designed to elicit discussions regarding key topics relevant to the experiences of women vendors during and following the implementation of COVID-19 pandemic policies. We explored the gendered considerations in the policies and the engagement women vendors in the formulation and implementation of such policies. We probed for the specific experiences with the implementation of the COVID-19 control measures and the direct and indirect impacts on the women-led businesses. Data collection was undertaken until theoretical saturation was achieved– the point at which no new ideas or themes emerged from the interviews. The research team maintained reflexivity throughout the process, and field notes maintained by research assistants were involved in the data analysis. Following each interview, the research assistants summarized the key insights, and conducted daily debriefing meetings with the broader research team.

Key informant interviews: We conducted (n = 20) face-to-face interviews with representatives the County Government of Kisumu, market leaders, and women group leaders, community loaning and microfinance representatives, community health promoters and leaders. The interviews lasted approximately 30 – 60 minutes with the key informants including compliance experts, enforcement offices, revenue collectors, and municipal and city government officials. Based on the expertise of the different government and community officials, we obtained in-depth insights into the policy formulation and implementation as well as the implications of policy regulations to women informal food vendors. We also sought to gather information on the interaction of the different entities/organizations and their supports to women informal food vendors during and after the pandemic.

In-depth interviews (n = 20) included female and some male food vendors who operated food businesses during and after the COVID-19 pandemic. The male participants were included to capture their perspectives of the experiences of female food vendor with the pandemic control measures. The recruitment was facilitated through our community partner – Pamoja CBO and in collaboration with the market leader. The market leaders invited potential participants to an information session and interested vendors gave informed consent and were invited to a face-to-face interview at a convenient place of their choice. The one-on-one interview participants were interviewed to understand the impacts of COVID-19-related policies on their business and household responsibilities, caregiving duties, interaction with customers and authority, food supply, physical and mental well-being, food security status, and the coping mechanisms they adopted. All the interviews were audio-recorded, translated, and transcribed to facilitate data analysis and interpretation.

Focus group discussions (n = 40) include four FGDs, each comprising ten women informal food vendors who were recruited through our community partner and the women market leaders. The women market leaders organized for an information session with the potential participants and those who met the inclusion criteria and gave informed consent were invited to centralized location close to the various key markets, with most of the group discussions being conducted in the community halls close to the markets. The face-to-face group discussion facilitated a participatory space for the women vendors to reflect as a collective on the impact of COVID-19 policies on their social and economic spheres. The participants shared their experiences with the enforcement and compliance of curfews, physical-distancing, movement restrictions, market closures, and the health and safety protocols that were imposed after the implementation of the COVID-19 policies. They shared the gendered effects of the policies articulated some recommendations towards future policy reforms to limit the negative impacts of policies formulated with limited consultations.

Data analysis

Primary qualitative data (IDIs, KIIs, and FGDs) were all audio-recorded following receipt of informed consent from all the participants. While KIIs were conducted in English and transcribed verbatim, IDIs and FGDs were primarily facilitated in Swahili or Luo, subsequently requiring translation into English and transcription for analysis. The transcription and translation were conducted by the local research assistants who facilitated the interviews and the discussion and were reviewed by the authors PMO and EOO who are fluent in English, Swahili, and Luo. Comprehensive data sets—comprising transcripts, policy documents, field notes, reflexive journals, and interview summaries—were imported into NVivo (QSR International, version 15) for systematic coding, management, and analysis.

The criterion used to determine that no new information was being generated is not defined. Saturation is mentioned, but the manuscript does not explain how it was established or at what point in the fieldwork it was considered to have been reached. Without this information, it is not possible to assess the sufficiency of the sample.

Thanks for the comment. A section of the methods now reads as follows:

Primary data collection

Research assistants trained in qualitative research techniques and fluent in English, Swahili, and the local Luo dialect, conducted participant recruitment and facilitated information sessions to support primary data collection through face-to-face interviews. The choice of language used was determined by the participant’s preference with most key informants preferring English. Most of the IDI and FGD participants preferred Swahili and/or Luo. The interviews were audio-recorded with the consent of participants. Sessions conducted in English were transcribed verbatim, while those conducted in Swahili or Luo were translated into English as they were being transcribed by the research assistant that had facilitated the sessions. The resulting transcripts were subsequently reviewed by the research assistants and two authors (PMO & EOO), who possess fluency in all three languages, to ensure the accuracy of the translations and data transcriptions.

Semi-structured interview guides were developed and utilized to facilitate KIIs, IDIs, and FGDs. These instruments were designed to elicit discussions regarding key topics relevant to the experiences of women vendors during and following the implementation of COVID-19 pandemic policies. We explored the gendered considerations in the policies and the engagement women vendors in the formulation and implementation of such policies. We probed for the specific experiences with the implementation of the COVID-19 control measures and the direct and indirect impacts on the women-led businesses. Data collection was undertaken until theoretical saturation was achieved– the point at which no new ideas or themes emerged from the interviews. The research team maintained reflexivity throughout the process, and field notes maintained by research assistants were involved in the data analysis. Following each interview, the research assistants summarized the key insights, and conducted daily debriefing meetings with the broader research team.

Regarding informed consent, the manuscript indicates that it was “written or verbal,” while other sections refer to written consent. This inconsistency should be resolved and presented in a uniform manner.

This inconsistency has ben corrected to who that participants ether gave verbal or written consents depending on their ability to write.

The document analysis component is mentioned as part of the design, but the procedure applied is not described. Criteria for selecting policies are indicated, but the manuscript does not explain how documents were analyzed, what categories were used, or how this component was integrated with interview and focus group data. Given that the study is presented as a policy analysis, this is a central element.

Thank you for this valuable comment. The sub-sections of the methods have been revised and more details regarding this feedback added in the analysis and Quality &Rigor sections. The subsections now read as follows:

……

Policy document analysis

The analysis of the four policy documents included in this study was guided by seven core principles of the Intersectionality-Based Policy Analysis (IBPA) framework: power, equity, intersecting categories, multi-level analysis, social justice, diverse knowledges, and reflexivity (Hankivsky et al., 2014). Adopting Braun and Clarke's(Braun & Clarke, 2006) reflexive thematic analysis, the researchers engaged in an iterative process of reading and re-reading the documents to gain familiarity with key control measures, objectives, and intervention areas designed to mitigate the effects of the COVID-19. Through a combination of deductive analysis, guided by the IBPA framework, and inductive analysis, a thematic code set was developed to facilitate the emergence of both pre-defined and new themes (Creswell, 2014; Strauss & Corbin, 1990). The predefined themes aligned with IBPA principles, encompassing power dynamics, intersecting identities, social justice, equity, multi-level analysis and reflexivity. The theme codes, transcripts, field notes, interview summaries, and the post-pandemic recovery policies were then imported and organized in NVivo QSR International, version 15, for line-by-line coding. To validate the theme code sets and the coding and analysis process, an independent peer researcher (GR) performed an independent analysis and cross-checked the analysis for consistency and accuracy. This process of peer checking allowed for multiple interpretations and understanding of the experiences of women informal food vendors and the identification of gaps within the policy documents (Hay, 2016). This peer-review process facilitated multiple interpretations of the experiences of women informal food vendors and assisted in identifying policy gaps. Rigor and trustworthiness were further established by triangulating evidence from policy documents, key informant narratives, and data from informal food vendors.

Primary data analysis

The analysis of the primary data from the KII, IDI, and FGD were also conducted inductively and deductively guided by the IBPA framework and following Braun and Clarke’s reflexive thematic analysis approach (Braun & Clarke, 2006; Hankivsky et al., 2014). The research team established initial themes and codes through prolonged immersion in the data, which included active participation in transcription and exhaustive review of the transcripts. These codes were subsequently collated into themes and sub-themes to establish a refined code set. All primary materials were imported into NVivo (QSR International, version 15) for comprehensive coding. Participant perspectives were accurately represented through the use of direct quotes (Tong et al., 2007). To ensure analytical rigor, peer debriefing was employed to verify the theme codes and the consistency of the line-by-line coding. Finally, data triangulation across primary sources and policy documents was utilized to enhance the credibility and trustworthiness of the finding.

Quality & Rigor

Various methodological strategies were employed to ensure the rigor of this study. First, data triangulation was achieved by synthesizing multiple sources, including policy document analysis and primary data from KIIs, FGDs, and IDIs. The integration of the policy document analysis and the qualitative evidence was an important element of triangulation to ensure rigor. The application of both inductive and deductive analytical approaches facilitated the triangulation of theoretical frameworks with lived experience perspectives. Furthermore, a purposive sampling technique was utilized to select respondents possessing specific expertise and lived experience (Johnson et al., 2020) relevant to COVID-19 control policies, community leadership, governance, and informal food vending Local research assistants from Pamoja CBO contributed to the data collection process and participated in peer review and debriefing sessions to maintain consistency in data collection, analysis, and interpretation. This peer debriefing process was supported through regular consultations among the research team members, specifically EOO, RG, and PMO.

Ethical considerations

Ethics approval for the study was granted by the University of Alberta Research Ethics Review Board (Protocol: Pro00138854) and AMREF Kenya Research Ethics Board (ESRC P1670/2024). We also obtained a verbal for participants that were unable to write a written consent if they were capable of writing. All participants gave informed consent (verbal or written) and received an honorarium of CAD $10 (KES 1,000), to honor their time and contributions to the study.

Finally, the relationship between the IBPA framework and the thematic analysis is not clearly described. The manuscript refers to both approaches, but it does not show how they were articulated in practice: whether IBPA guided coding, whether it was used to reorganize categories, or whether it was applied at a later interpretative stage. This lack of definition affects the methodological coherence of the study.

More details on IBPA framework and how it guided our analysis has been added in the introduction section and the use of both deductive (IBPA) and inductive in the analysis to develop themes included in the methods section. The Introduction section and the methods sections now reads as follows:

Introduction

…………….

This study extends our understanding of the extent to which the perspectives of women business-owners were considered in the formulation and implementation of the pandemic control policies in secondary cities in the Global South. Understanding the gendered dimensions of the COVID-19 policies on women engaged in informal food businesses is essential for advocating for equitable policy frameworks that safeguard the food and nutritional security and economic stability of urban households, particularly, in times of emergencies. The study adopted the intersectionality-based policy analysis (IBPA) framework (Hankivsky et al., 2014) to investigate the influence of power dynamics and systemic structures on the marginalization of women food vendors within secondary cities during the COVID-19 pandemic. The IBPA framework facilitated an analysis that extended beyond gender as an individual level factor, enabling an exploration of how intersecting identities, policies and regulations – such as the COVID-19 control measures construct social locations and inequities marginalize specific groups, such as the women food vendors.

We utilized seven of the eight key principles of the IBPA framework – the principles of equity, power, intersecting categories, multi-level analysis, reflexivity, social justice, and diverse knowledges (Hankivsky et al., 2014). These seven IBPA framework principles (See Figure 1 below) guided the analysis of the COVID-19-related policies, key informant interviews, focus group discussions and in-depth interview data as well as the interpretation of the findings.

The principle of intersecting categories was employed to examine the interactions between various overlapping social identities – including gender, age, sexual orientation, marital status, educational attainment, cultural orientation, and socio-economic standing—and pandemic-related control strategies in intensifying the marginalization of female vendors. Furthermore, the analysis assessed whether these policies acknowledged the heterogeneous identities of informal food vendors and the specific requirements arising from such positionalities. By applying an intersectional perspective to policy review and qualitative narrative analysis, this study identified the ways in which social locations influenced women’s experiences of the pandemic policies and how inequalities predicated upon the design, dissemination, and interpretation of (Kanenberg & Leal, 2019).

In integrating the principle of power and multi-level analysis, we sought to highlight how overlapping power either facilitated inclusion or exacerbated systemic inequities for female vendors within the informal sector. In the Kenyan context—where healthcare service delivery is devolved to county and municipal authorities while legislative mandate remains centralized federal mandate, complex hierarchies of power emerge. Within such complex governance environments, the management of a public health crisis such as the COVID-19 pandemic necessitated a multisectoral approach characterized by deliberate engagement of the diverse partners, particularly the disadvantaged populations – such as the informal food vendors.

Furthermore, our analytical framework integrated the gender equity lens and the principle of social justice to examine the extent of gender inclusivity with the COVID-19 control measures, while considering the overlapping layers of power in the policy processes. By applying the principle of diverse knowledge, the framework enabled us to interrogate whether the voices of women in informal food vending were integrated in policy development and how power structures influenced the inclusion or exclusion of their diverse perspectives in policy decision making. Whilst studies in other jurisdictions indicated that governments neglected the gender-specific requirements of women during COVID-19 policy responses (Chitando, 2020), the application of the IBPA framework in this study enabled the advancement of the analysis beyond gender to also consider other intersecting identities, tenets of power dynamics, promotion of a social justice lens, and appreciation of diverse knowledge in policy analysis. We applied the principles while acknowledging the relevance of being self-reflexive. Notably, the IBPA principle of time and space, which analyses the dynamics of policy inequalities over time and across historical and geographical spaces, was excluded from this study. This is because the research focussed on the gendered COVID-19 policy dynamics at a specified time and place.

<<<<Figure 1: The conceptual framework for the IBPA guiding principles for analysis of COVID-19 control policies & the experiences of female informal food vendors>>>

Methods

…………

Policy document analysis

The analysis of the four policy documents included in this study was guided by seven core principles of the Intersectionality-Based Policy Analysis (IBPA) framework: power, equity, intersecting categories, multi-level analysis, social justice, diverse knowledges, and reflexivity (Hankivsky et al., 2014). Adopting Braun and Clarke's(Braun & Clarke, 2006) reflexive thematic analysis, the researchers engaged in an iterative process of reading and re-reading the documents to gain familiarity with key control measures, objectives, and intervention areas designed to mitigate the effects of the COVID-19. Through a combination of deductive analysis, guided by the IBPA framework, and inductive analysis, a thematic code set was developed to facilitate the emergence of both pre-defined and new themes (Creswell, 2014; Strauss & Corbin, 1990). The predefined themes aligned with IBPA principles, encompassing power dynamics, intersecting identities, social justice, equity, multi-level analysis and reflexivity. The theme codes, transcripts, field notes, interview summaries, and the post-pandemic recovery policies were then imported and organized in NVivo QSR International, version 15, for line-by-line coding. To validate the theme code sets and the coding and analysis process, an independent peer researcher (GR) performed an independent analysis and cross-checked the analysis for consistency and accuracy. This process of peer checking allowed for multiple interpretations and understanding of the experiences of women informal food vendors and the identification of gaps within the policy documents (Hay, 2016). This peer-review process facilitated multiple interpretations of the experiences of women informal food vendors and assisted in identifying policy gaps. Rigor and trustworthiness were further established by triangulating evidence from policy documents, key informant narratives, and data from informal food vendors.

Primary data analysis

The analysis of the primary data from the KII, IDI, and FGD were also conducted inductively and deductively guided by the IBPA framework and following Braun and Clarke’s reflexive thematic analysis approach (Braun & Clarke, 2006; Hankivsky et al., 2014). The research team established initial themes and codes through prolonged immersion in the data, which included active participation in transcription and exhaustive review of the transcripts. These codes were subsequently collated into themes and sub-themes to establish a refined code set. All primary materials were imported into NVivo (QSR International, version 15) for comprehensive coding. Participant perspectives were accurately represented through the use of direct quotes (Tong et al., 2007). To ensure analytical rigor, peer debriefing was employed to verify the theme codes and the consistency of the line-by-line coding. Finally, data triangulation across primary sources and policy documents was utilized to enhance the credibility and trustworthiness of the finding.

Quality & Rigor

Various methodological strategies were employed to ensure the rigor of this study. First, data triangulation was achieved by synthesizing multiple sources, including policy document analysis and primary data from KIIs, FGDs, and IDIs. The integration of the policy document analysis and the qualitative evidence was an important element of triangulation to ensure rigor. The application of both inductive and deductive analytical approaches facilitated the triangulation of theoretical frameworks with lived experience perspectives. Furthermore, a purposive sampling technique was utilized to select respondents possessing specific expertise and lived experience (Johnson et al., 2020) relevant to COVID-19 control policies, community leadership, governance, and informal food vending Local research assistants from Pamoja CBO contributed to the data collection process and participated in peer review and debriefing sessions to maintain consistency in data collection, analysis, and interpretation. This peer debriefing process was supported through regular consultations among the research team members, specifically EOO, RG, and PMO.

Ethical considerations

Ethics approval for the study was granted by the University of Alberta Research Ethics Review Board (Protocol: Pro00138854) and AMREF Kenya Research Ethics Board (ESRC P1670/2024). We also obtained a verbal for participants that were unable to write a written consent if they were capable of writing. All participants gave informed consent (verbal or written) and received an honorarium of CAD $10 (KES 1,000), to honor their time and contributions to the study.

ANALYSIS

The manuscript mentions the participation of an independent researcher, but their role is not clearly defined.

This has been addressed and details about the role of an independent research – peer reviewer added.

Validation strategies are mentioned, including triangulation and related approaches, but they are not described in sufficient detail to understand how they were implemented.

Quality and rigor section has been revised with more details on how the various strategies were applied in the study.

RESULTS

The results are organized thematically and allow the reader to follow the general direction of the analysis; however, the section presents problems of structure, internal consistency, and differentiation between levels of information.

The section does not begin directly with the findings. The opening paragraphs return to methodological elements and describe again the organization of the analysis. This weakens the distinction between methods and results and delays the presentation of empirical evidence.

Well noted and has been revised

An inconsistency is observed in the characterization of the sample. The age range reported in the text does not match the one presented in the corresponding table. This type of discrepancy affects the internal coherence of the manuscript.

In several sections, there is no clear distinction between three levels of information: (i) evidence from analyzed documents, (ii) participant testimonies, and (iii) interpretation developed by the authors. This overlap makes it difficult to identify what corresponds to evidence and what corresponds to analysis.

Repetitions are observed across subthemes, particularly regarding exclusion of vendors, increased economic burden, and institutional control practices. These repetitions do not add new elements and fragment the argument, as the same idea appears in different sections without analytical progression.

The way some findings are introduced tends to be declarative. Repeated use of constructions such as “our analysis revealed” or similar expressions does not add information and can be replaced by direct presentation of the evidence.

The results section has also been extensively revised and the suggestions incorporated. Much appreciation for all the great contributions and suggestions.

A problem remains in table numbering, with duplication of “Table 1.”

Corrected!

The use of quotations requires adjustment. In some cases, the quotation repeats what has already been stated, without adding new information. In other cases, the quotation and the interpretation are not clearly separated, making it difficult to distinguish the participant’s voice from that of the authors.

Thanks for this comment. Participants’ direct quotations are italicized in the results section. The unitalicized sections are the key highlights from the quotes. Hopefully this gives a distinction on what is said directly by the participants vs what the authors are hearing or reflecting on from the quotes.

Additionally, in some sections, results are presented together with immediate interpretation, without clearly delimiting the descriptive level. This is not necessarily incorrect, but it should be handled carefully to ensure that evidence is not subordinated to interpretation from the outset.

The results section has also been extensively revised and the suggestions incorporated. Much appreciation for all the great contributions and suggestions.

DISCUSSION

The discussion presents a clear expansion compared to the original version and shows an effort to connect the findings with additional literature and with the principles of the IBPA framework. However, the section still presents problems in the articulation between results, interpretation, and theoretical argument.

The first issue is analytical. IBPA principles are mentioned repeatedly, but it is not always clear how each of them emerges from the empirical material. In several points, principles such as power, equity, social justice, or intersecting categories are introduced as interpretative axes, but the manuscript does not consistently show the transition from the evidence presented in the results to the assignment of that evidence to those principles.

The second issue concerns argumentative sequence. In several parts, the discussion moves from findings to broader claims without sufficient intermediate development, particularly when linking results to normative or constitutional frameworks.

There is also repetition of arguments already presented in the results.

Comparative literature is incorporated, but the criteria for comparison are not always clearly established.

The concept of “compliance fatigue” is introduced without sufficient development.

Some statements adopt a stronger tone than what the qualitative design can support.

Language issues remain and require editorial revision.

The discussion section has been revised extensively to ensure clear and coherent flow of the key findings in relation to existing literature. The discussion now reads as follows:

Discussion

This study aimed to examine the sociocultural and gender disparities inherent in the formulation and implementation of COVID-19 mitigation policies and the subsequent experiences of women within the informal food vending sector. The findings reveal that the formulation and execution of these pandemic mitigation measures were characterized by a top-down approach that limited the engagement of women food vendors. Furthermore, the policies were fragmented by varying levels of power and power dynamics, reinforced prevailing inequities, and neglected the diverse intersecting identities of informal food vendors. Women vendors were systematically excluded from policy-making processes due to existing governance structures wherein health policies are formulated at the national level while service delivery is delegated to county governments, with most of these roles being male dominated. At the city and community levels, women vendors possess limited power and influence; they are frequently excluded from key decision-making processes due to systemic barriers that reinforce power hierarchies and shape policy outcomes. Our research identified several critical barriers contributing to the restricted engagement of women in the COVID-19 policy-making process within Kisumu. The factors included time constraints necessitated by the urgency of the crisis, sociocultural and gender inequities, a lack of accessible information regarding participation opportunities, and a reliance on technocratic expertise within the fields of health, governance, and policy. Such findings correspond with the observations of Martínez and Young (2022), who argued that the prioritization of technical experts in policy development by governments limited the participation of street vendors in decision-making processes. Furthermore, the pervasive societal culture of patriarchy served as a significant impediment to women involvement in political and leadership roles. Despite constitutional provisions for affirmative action and the establishment of County Women Representative positions in Kenya, policy discussions and community leadership remain predominantly male-dominated. These structural barriers reinforce gender hierarchies and the exclusion of women from decision-making at multiple levels of governance and society. Consequently, this lack of representation diminished the responsiveness of policies to the specific circumstances of women in the informal food vending sector, thereby exacerbating existing socio-economic disparities. (Coalition on Violence Against Women & Making It Work, 2023).

Narratives from the vendors emphasized this systematic exclusion, with participants frequently employing the expression "forced on us" to describe their marginalization and the lack of prior consultation regarding policy initiatives. These exclusionary practices appear to deviate from Article 174 (c) f the Constitution of Kenya, which mandates the enhancement of public participation in decision-making processes affecting the citizens (Laws of Kenya, 2013). Although scholarly evidence indicates that the political empowerment of women in pandemic-related policy-making lead to improved gender-sensitive health and economic outcomes (Abras et al., 2021; Huang & Lin, 2022), the approach in Kisumu City and many other cities in the Global South such as Rangpur in Bangladesh and Rusizi and Rubavu in Rwanda, remained highly centralized within the national Ministry of Health. The development and implementation of mitigation measures were restricted to technical experts and government officials, failing to incorporate community engagement strategies, gender-based considerations, or formal feedback mechanisms for the public, particularly for women operating in the informal food economy.

Furthermore, our study findings indicate that these policies reinforced existing inequities by failing to account for the diverse intersecting identities of marginalized groups, such as women food vendors in secondary cities. The policies did not contextualize the lived realities of these women with reference to their intersecting identities or prevailing sociocultural practices. Women vendors reported instances of inequitable treatment, including assault, harassment, and extortion by law enforcement officers. Similarly, (Solymári et al., 2022) observed that the brutality associated with COVID-19 policy enforcement in Kenyan informal settlements infringed upon fundamental human rights. This occurred against the backdrop of a male-dominated county enforcement team in Kisumu, which contributed to biased regulation enforcement (Loo et al., 2021). Such male dominance in enforcement predisposes women to violence and physical abuse at their places of business, posing severe health and safety risks and curtailing their agency to support their families. These experiences of harassment and the application of excessive force demonstrate how established power structures directly impacted the gendered experiences of women in the informal food sector, with some vendors reporting being compelled to pay bribes of up to five thousand shillings to avoid arrest.

Likewise, research in Nairobi by Wandera et al. (2023) documented that women in the informal workforce were subjected to physical abuse by police and municipal officers during the enforcement of curfews. This indicates a consistent pattern of COVID-19 policy impacts on women vendors across both primary and secondary urban centers. These findings are corroborated by Kiaka and colleagues who emphasized that the pandemic exacerbated instances of bribery and harassment in the Global South, particularly affecting informal vendors whose survival necessitated significant resilience. Exposure to such harassment and assault restricted women’s agency to facilitate urban household food provision and sustain their families during the pandemic. Consequently, it is argued that during periods of emergency, governments must implement gender-responsive governance systems to facilitate the engagement of women in the informal sector and ensure that policies address the specific requirements and experiences of this marginalized yet essential segment of the urban population.

Our study further provides evidence regarding the absence of an intersectional framework in the formulation of COVID-19 policies. Informal traders in Kisumu constitute a heterogeneous group characterized by diverse genders, ages, socio-economic statuses, cultural backgrounds, marital status, abilities, and religious affiliations, among other intersecting social identities. However, the pandemic mitigation strategies utilized a blanket policy approach that failed to account for the varied settings and specific identities of these vendors. This was also noted by Dutta et al. (2021) in India, where COVID-19 lockdown policies adopted a one-size-fits-all approach and overlooked existing gender differences.

These policy interventions did not consider the contextual factors such as the prevailing socio-economic situation in low- and middle-income countries (LMICs), and the pre-existing inequalities faced by women informal workers, including patriarchal structures and gender-based discrimination. Women vendors were already facing compounded challenges, including the precarity of their businesses and work, gender-based violence, discrimination, economic marginalization, and sexual harassment before the occurrence COVID-19 pandemic. The lack of gendered considerations in the policies worsened the prevailing inequalities creating an additional burden to the women vendors and influencing their resilience post-pandemic. While we acknowledge that the pandemic was a relatively new and urgent event that required immediate action through enforcement of public health orders inform of the control measures, we argue that governments must balance such urgency with inclusive and equitable engagement of women in public policy decision-making during emergencies to mitigate unintended negative impacts.

Women faced societal expectations to take up extra caregiving roles at the expense of paid work. Notably, findings from this study raise key questions regarding the value of unpaid household and caregiving duties undertaken by women. According to the United Nation’s Development Program-UN Women COVID-19 Global Gender Response Tracker, fewer than a third of the COVID-19 policy measures implemented in Kenya applied a gender perspective, with none accounting for unpaid care work (UN Women, 2021, 2022). Our findings extend this work by uncovering the experiences of women to the gender gaps in policies in localized settings of secondary cities.

Additionally, the COVID-19 policy measures introduced a significant number of new regulations with which traders were required to comply, potentially inducing "compliance fatigue" among vendors, considering the strict enforcement and use of force by the enforcement officers. This aligns with the findings of Martínez and Young (2022), which demonstrate that the exclusion of vulnerable groups in policy formulation and implementation undermines policy effectiveness and exacerbates marginalization. Such evidence may inform future policy decisions concerning the meaningful engagement of all stakeholders to achieve inclusive and more equitable policy outcomes, particularly during emergencies. As articulated by Cattapan et al., (2020), collaborative policy development should be guided by the principle of "nothing for us without us," which promotes the substantive participation of vulnerable groups in policy design and execution.

CONCLUSIONS

The conclusions summarize the general topic of the study but do not clearly delimit its specific contribution.

The section moves toward policy recommendations without first clearly establishing the main findings and their analytical contribution.

There is no clear distinction between findings, interpretation, and implications.

Some statements go beyond what can be supported by the study design.

The contribution is present, but not clearly articulated.

The conclusion has also been revised and now reads as follows:

Conclusions

In conclusion, this research possesses significant scholarly and practical relevance, offering both methodological and substantive contributions. By utilizing an IBPA framework to examine urban informal economies and the experiences of informal food vendors, the study extends policy discourse beyond a singular focus on gender. It underscores the critical role of intersecting identities in shaping the lived experiences of women vendors and facilitates the integration of intersectional policy analysis within health research.

Substantively, this research indicates that pandemic containment measures were developed and enforced utilizing a top-down methodology, which effectively marginalized female informal food vendors and failed to account for their unique needs and diverse intersecting identities. Additionally, pre-existing power dynamics and gender-based vulnerabilities were reinforced and exacerbated by the regulatory measures, including curfews, stay-at-home mandates, and physical distancing protocols.

To address this gap, policy interventions should recognize the unique roles of women informal food vendors by incorporating targeted economic support measures, flexible market regulations, and inclusive communication strategies during public health emergencies. While the global health crisis necessitated immediate public health interventions, these findings emphasize the imperative for inclusive policy-making processes that incorporate female informal food vendors into the policy formulation and implementation processes. For example, governments should adopt gender-responsive emergency governance measures that intentionally solicit and integrate the perspectives and lived realities of informal vendors. Furthermore, governments should institutionalize mechanisms for the meaningful participation of informal vendors in policy design through recognized market associations and community-based collectives. To ensure efficacy, policy interventions must be designed in collaboration with marginalized groups to accommodate social diversity and respond to localized needs. In the context of female informal food vendors, future regulatory frameworks and emergency directives should adopt a gendered perspective to formally recognize their unique contributions and safeguard their role in maintaining urban food security.

Such integration would enhance the gender responsiveness of policy interventions and mitigate structural inequalities. Future scholarly inquiries should expand upon this analysis by conducting comparative studies across various secondary cities to evaluate how intersectional inequalities shape the experiences of informal food vendors under disparate governance and policy frameworks.

ETHICS AND DATA AVAILABILITY

Data availability is not clearly described within the manuscript.

Thank you for this! Revised for clarity.

REFERENCES

Formatting is not consistent.

We appreciate this observation. The reference have been cross checked for consistency.

LANGUAGE

The manuscript is understandable, but it presents issues in writing and consistency.

A professional language revision is recommended.

Thank you for this observation. Extensive editorial review of the manuscript has been done to enhance readability and clarity of the key messages.

Attachment Submitted filename: Rebuttal to Reviewers and Editors Comments - May 15, 2026.docx

# Peer review: Decision Letter 2

**DOI:** 10.1371/journal.pone.0359620.r005

**Authors:** Wisarut Suwanprasert

14 Jul 2026

Dear Dr. Onyango,

Please submit your revised manuscript by Aug 28 2026 11:59PM. If you will need more time than this to complete your revisions, please reply to this message or contact the journal office at plosone@plos.org. When you're ready to submit your revision, log on to https://www.editorialmanager.com/pone/ and select the 'Submissions Needing Revision' folder to locate your manuscript file.

- A letter that responds to each point raised by the academic editor and reviewer(s). You should upload this letter as a separate file labeled 'Response to Reviewers'.
- A marked-up copy of your manuscript that highlights changes made to the original version. You should upload this as a separate file labeled 'Revised Manuscript with Track Changes'.
- An unmarked version of your revised paper without tracked changes. You should upload this as a separate file labeled 'Manuscript'.

If you would like to make changes to your financial disclosure, please include your updated statement in your cover letter. Guidelines for resubmitting your figure files are available below the reviewer comments at the end of this letter.

If applicable, we recommend that you deposit your laboratory protocols in protocols.io to enhance the reproducibility of your results. Protocols.io assigns your protocol its own identifier (DOI) so that it can be cited independently in the future. For instructions see: https://journals.plos.org/plosone/s/submission-guidelines#loc-laboratory-protocols. Additionally, PLOS ONE offers an option for publishing peer-reviewed Lab Protocol articles, which describe protocols hosted on protocols.io. Read more information on sharing protocols at https://plos.org/protocols?utm_medium=editorial-email&utm_source=authorletters&utm_campaign=protocols.

As the corresponding author, your ORCID iD is verified in the submission system and will appear in the published article. PLOS supports the use of ORCID, and we encourage all coauthors to register for an ORCID iD and use it as well. Please encourage your coauthors to verify their ORCID iD within the submission system before final acceptance, as unverified ORCID iDs will not appear in the published article. *Only*  the individual author can complete the verification step; PLOS staff *cannot*  verify ORCID iDs on behalf of authors.

We look forward to receiving your revised manuscript.

Kind regards,

Wisarut Suwanprasert, Ph.D.

Academic Editor

PLOS One

**Journal Requirements:**

1. If the reviewer comments include a recommendation to cite specific previously published works, please review and evaluate these publications to determine whether they are relevant and should be cited. There is no requirement to cite these works unless the editor has indicated otherwise.

2. Please review your reference list to ensure that it is complete and correct. If you have cited papers that have been retracted, please include the rationale for doing so in the manuscript text, or remove these references and replace them with relevant current references. Any changes to the reference list should be mentioned in the rebuttal letter that accompanies your revised manuscript. If you need to cite a retracted article, indicate the article’s retracted status in the References list and also include a citation and full reference for the retraction notice.

[Note: HTML markup is below. Please do not edit.]

Reviewers' comments:

Reviewer's Responses to Questions

**Comments to the Author**

Reviewer #3: (No Response)

**********

2. Is the manuscript technically sound, and do the data support the conclusions?

Reviewer #3: (No Response)

**********

3. Has the statistical analysis been performed appropriately and rigorously?

Reviewer #3: N/A

**********

4. Have the authors made all data underlying the findings in their manuscript fully available?

The PLOS Data policy

Reviewer #3: (No Response)

**********

5. Is the manuscript presented in an intelligible fashion and written in standard English?

Reviewer #3: Yes

**********

Reviewer #3: The revised manuscript demonstrates substantial improvement in both scientific quality and presentation.

• The Introduction has been significantly strengthened, providing a clearer contextualization of COVID-19, gender inequalities, and the importance of secondary cities.

• The expanded description of the Intersectionality-Based Policy Analysis (IBPA) framework, together with the inclusion of its guiding principles, considerably improves the conceptual foundation of the study.

• The Methods section has also been enhanced through clearer descriptions of participant recruitment, data collection, analytical procedures, and ethical considerations.

• In addition, the Discussion now provides a more analytical interpretation of the findings by explicitly linking them to issues of power relations, structural inequalities, intersecting identities, and gender-responsive governance.

Despite these important improvements, several minor issues should still be addressed before the manuscript is suitable for publication.

1. Explicitly state the manuscript's contribution

Although the conclusion has been revised, the manuscript would benefit from a more explicit statement of its contributions. Rather than leaving these contributions implicit, consider clearly distinguishing the:

• theoretical contribution (advancing intersectionality-informed policy analysis in informal urban economies);

• methodological contribution (application of the IBPA framework integrated with qualitative inquiry);

• policy contribution (implications for inclusive urban governance and emergency policy development).

The authors should add a concise paragraph summarizing these three contributions in order to strengthen the manuscript's overall impact.

2. Clarify the research objective

• The Introduction has been substantially improved; however, it still concludes primarily with the rationale for the study rather than a clearly formulated research objective.

• The authors should add at the end of the Introduction one explicit sentence beginning, for example:

o "This study aimed to...” or "The objective of this study was..."

o This would improve the overall structure and clarity of the manuscript.

3. Strengthen the operational policy recommendations

• The revised Discussion and Conclusion now contain stronger policy implications. The policy implications have been substantially strengthened and are considerably more actionable than in the previous version.

• Nevertheless, a few recommendations remain relatively broad and could be further enhanced by briefly illustrating how the proposed governance measures could be operationalized in practice:

o “… governments should adopt gender-responsive emergency governance measures that intentionally solicit and integrate the perspectives and lived realities of informal vendors." (lines 817-819). This recommendation is appropriate, but it remains relatively general. The manuscript could briefly indicate how governments could operationalize this recommendation (e.g., formal representation of women vendors on emergency advisory committees, mandatory stakeholder consultations before implementing market restrictions, or participatory policy forums).

o "…. governments should institutionalize mechanisms for the meaningful participation of informal vendors in policy design through recognized market associations and community-based collectives." (lines 819-821). This is a stronger recommendation than in the previous version and directly addresses one of the reviewer's concerns. However, it could be strengthened further by briefly indicating what these mechanisms might involve, for example permanent consultative committees, representation within municipal emergency response structures, or structured feedback mechanisms during public health emergencies.

o "To ensure efficacy, policy interventions must be designed in collaboration with marginalized groups to accommodate social diversity and respond to localized needs." (Lines 822-823). Although this recommendation is well justified, it remains somewhat conceptual. Providing one or two examples of concrete policy instruments (e.g., targeted financial assistance, flexible licensing arrangements, emergency income protection, or tailored communication strategies) would enhance its practical value for policymakers.

• Where appropriate, consider providing more concrete examples of how governments or local authorities could implement gender-responsive and intersectionality-informed policies (e.g., stakeholder consultation mechanisms, representation of women vendors in emergency planning, targeted support programmes, or inclusive communication strategies).

The Conclusion is much stronger than in the previous version.

• However, it could be made even more concise by focusing primarily on the principal findings, the study's contribution, and future research directions, while limiting discussion-style interpretation.

Although the Discussion is now more analytical, some repetition of empirical findings remains, particularly regarding exclusion from policy-making, enforcement-related harassment, and the blanket nature of COVID-19 policies.

• The authors should consider reducing descriptive repetition and using the Discussion primarily to interpret these findings through the IBPA framework:

o The Results already present the issue of extortion/harassment: “The overlapping policy interventions created an avenue for extortion and harassment of the women vendors:” (lines 649-650).

o The Discussion later repeats the same empirical finding: “Women vendors reported instances of inequitable treatment, including assault, harassment, and extortion by law enforcement officers.” (Lines 719-720). Suggestion: the Discussion could reduce the descriptive repetition and focus directly on interpretation: “These enforcement practices illustrate how gendered power relations shaped the implementation of COVID-19 measures in informal urban economies.”

The manuscript would benefit from one final careful language edit (for example, "venders" instead of "vendors" in the Abstract).

**********

what does this mean?). If published, this will include your full peer review and any attached files.

If you choose “no”, your identity will remain anonymous but your review may still be made public.

**Do you want your identity to be public for this peer review?**  For information about this choice, including consent withdrawal, please see our Privacy Policy

Reviewer #3: No

**********

[NOTE: If reviewer comments were submitted as an attachment file, they will be attached to this email and accessible via the submission site. Please log into your account, locate the manuscript record, and check for the action link "View Attachments". If this link does not appear, there are no attachment files.]

To ensure your figures meet our technical requirements, please review our figure guidelines: https://journals.plos.org/plosone/s/figures

You may also use PLOS’s free figure tool, NAAS, to help you prepare publication quality figures: https://journals.plos.org/plosone/s/figures#loc-tools-for-figure-preparation.

NAAS will assess whether your figures meet our technical requirements by comparing each figure against our figure specifications.

Attachment Submitted filename: Revision 05.07.2026.docx

# Author comment: Author response to Decision Letter 3

**DOI:** 10.1371/journal.pone.0359620.r006

24 Jul 2026

Response to Editor’s comments

NB: Highlighted in yellow are the edited/revised sections in the paper.

NB: Highlighted in blue are the editor/reviewers comments and suggested edits

NB: Highlighted in gray are our responses to the suggestions/comments

The revised manuscript demonstrates substantial improvement in both scientific quality and presentation.

• The Introduction has been significantly strengthened, providing a clearer contextualization of COVID-19, gender inequalities, and the importance of secondary cities.

• The expanded description of the Intersectionality-Based Policy Analysis (IBPA) framework, together with the inclusion of its guiding principles, considerably improves the conceptual foundation of the study.

• The Methods section has also been enhanced through clearer descriptions of participant recruitment, data collection, analytical procedures, and ethical considerations.

• In addition, the Discussion now provides a more analytical interpretation of the findings by explicitly linking them to issues of power relations, structural inequalities, intersecting identities, and gender-responsive governance.

Thank you for acknowledging the improvements made in the paper!

Despite these important improvements, several minor issues should still be addressed before the manuscript is suitable for publication.

1. Explicitly state the manuscript's contribution

Although the conclusion has been revised, the manuscript would benefit from a more explicit statement of its contributions. Rather than leaving these contributions implicit, consider clearly distinguishing the:

• theoretical contribution (advancing intersectionality-informed policy analysis in informal urban economies);

• methodological contribution (application of the IBPA framework integrated with qualitative inquiry);

• policy contribution (implications for inclusive urban governance and emergency policy development).

The authors should add a concise paragraph summarizing these three contributions in order to strengthen the manuscript's overall impact.

Thanks for this valuable observation. The comment has been incorporated in the discussion and conclusion section which now reads as follows:

Discussion

………………

This study is not without limitations and it is important to recognize its limits. First, the utilization of a cross-sectional research design restricted data collection to a specific timeframe, specifically between July 10, 2024, and October 15, 2024. Because this period occurred a few years after the COVID-19 pandemic, participant recollections may have lacked certain details that could further enrich the findings. Second, the geographic focus was confined to a single county out of Kenya's 47 counties, which may constrain the generalizability of the outcomes nationwide; nevertheless, these findings may possess transferability to urban centers exhibiting comparable characteristics with Kisumu City. Third, the scope was strictly limited to the informal food economy, thereby restricting generalizability to other segments of the informal sector. Furthermore, the reliance on self-reported data introduces potential social desirability bias, particularly regarding personal reflections on pandemic control measures, their impacts on women-led informal food enterprises, and identified socio-gender policy disparities. Notwithstanding these limitations, prior scholarship has successfully utilized analogous methodological approaches to document lived experiences under pandemic restrictions. Consequently, the findings of this study remain comparable to preceding research and offer significant contributions to the literature and practice regarding the gendered dynamics of the informal food sector in secondary cities within the Global South.

Conclusions

In conclusion, this study offers substantial theoretical, methodological, and practical contributions to the literature on urban informal economies, particularly regarding the management of public health emergencies. From a theoretical perspective, by utilizing the IBPA framework, this research advances intersectionality-informed policy analysis in the informal economies. It systematically reveals how blanket COVID-19 mitigation policies failed to account for the heterogeneous, intersecting identities of women food vendors, thereby reinforcing pre-existing socio-economic and cultural disparities. By documenting these gendered experiences of exclusion in both policy formulation and enforcement during emergencies, the study provides a critical empirical basis for shifting governance toward gender-responsive, inclusive frameworks that prioritize the substantive engagement of marginalized populations.

While the COVID-19 pandemic may necessitate immediate public health interventions, findings from the current study emphasize the imperative for inclusive policy-making processes that incorporate female informal food vendors into the policy formulation and implementation processes. Governments must adopt gender-responsive emergency governance measures that intentionally solicit the formal representation of women vendors on emergency advisory committees coupled with mandatory consultation of partners before implementing market restrictions. Through these deliberate policy participatory initiatives that engages the female informal vendors, governments will be able to accommodate social diversity and respond to localized needs while safeguarding their unique contributions and role in maintaining urban food security.

Additionally, the study makes a methodological contribution by demonstrating the relevance of integrating IBPA framework, review of policy documents, and qualitative evidence. In so doing, the study reveals how policies intersect with lived experiences and underscores the significance of qualitative evidence in capturing the human elements of policy impact, specifically, the socioeconomic and gender disparities in secondary cities. Substantively, this research indicates that pandemic containment measures were developed and enforced utilizing a top-down methodology, which effectively marginalized female informal food vendors and failed to account for their unique needs and diverse intersecting identities. Additionally, pre-existing power dynamics and gender-based vulnerabilities were reinforced and exacerbated by the regulatory measures, including curfews, stay-at-home mandates, and physical distancing protocols.

To address this gap, policy interventions should recognize the unique roles of women informal food vendors by incorporating targeted economic support measures (e.g., targeted financial assistance, flexible licensing arrangements, and emergency income protection), flexible market regulations, and inclusive and tailored communication strategies during public health emergencies. Such integration would enhance the gender responsiveness of policy interventions and mitigate structural inequalities. Future scholarly inquiries should expand upon this analysis by conducting comparative studies across various secondary cities to evaluate how intersectional inequalities shape the experiences of informal food vendors under disparate governance and policy frameworks.

2. Clarify the research objective

• The Introduction has been substantially improved; however, it still concludes primarily with the rationale for the study rather than a clearly formulated research objective.

• The authors should add at the end of the Introduction one explicit sentence beginning, for example:

o "This study aimed to...” or "The objective of this study was..."

o This would improve the overall structure and clarity of the manuscript.

We appreciate this feedback. It has been addressed and the introduction to the paper now reads as follows:

Introduction

In early 2020, the World Health Organization (WHO) designated COVID-19 as a global pandemic, necessitating immediate and comprehensive public health interventions (World Health Organization (WHO), 2020). Consequently, governments worldwide implemented mandatory containment measures, including lockdowns and curfews (Koh, 2020). Furthermore, safety protocols such as the utilization of face masks, regular hand hygiene, sanitization, and physical distancing were established to mitigate the transmission of the coronavirus (Mwakisha et al., 2024). These control measures coupled with the effects of the diseases – COVID-19 had devastating effects on global trade and markets, which affected both the formal and informal economies across the world (Alon et al., 2022; Alon et al., 2020; Mishra & Rampal, 2020). Within the informal sector, the control policies triggered events that shaped social and economic outcomes differently for men and women engaged in the sector (Kabeer et al., 2021); hence, exposing and exacerbating the existing socioeconomic and cultural inequalities, especially in urban centres (Finn, 2023; Finn & Kobayashi, 2020; Odhiambo et al., 2025; Onyango et al., 2021; Sharifi & Khavarian-Garmsir, 2020; Ukala et al., 2020).

In cities in the global South, women disproportionately bore the burden of the pandemic and its control measures, with women food vendors experiencing an array of systemic challenges (Afifu et al., 2021; Kiplagat et al., 2025; Onyango et al., 2021; Onyango et al., 2023). These challenges encompassed reduced market hours, restricted movement, disruption of food supply chains, increased household and caregiving responsibilities, alongside significant income loss and heightened food insecurity (Afifu et al., 2021; Kimani-Murage et al., 2022). The impact was particularly severe for many women-led informal food businesses which depend fundamentally on robust social interaction and close physical proximity to their clientele in public spaces—operational dimensions that were drastically restricted by pandemic containment protocols (Finn & Kobayashi, 2020). Such measures exacerbated extant inequalities, largely due to failure to integrate the specific socio-cultural and economic exigencies faced by women into policy frameworks (Odhiambo et al., 2025; Ukala et al., 2020).

Furthermore, the absence of gender-sensitive considerations in policy initiatives introduced unique complications for women during the pandemic (UN Women, 2024). Women informal food vendors faced substantial barriers in securing financial assistance from both governmental and private financial institutions (Donga & Chimucheka, 2024; Sinyolo et al., 2022). In the African context, these difficulties were intensified by entrenched socio-cultural norms and patriarchal structures, which frequently undermine women's autonomy and ownership of assets required as collateral for credit access (Fletschner & Kenney, 2014; Kiplagat et al., 2025).

Across the majority of the cities within the Global South —and specifically across secondary cities, women are over-represented in the informal economic sector, with a heavy concentration in the food industry (Donga & Chimucheka, 2024). These secondary cities function as critical regional, administrative, and economic focal points and are characterized by a strong urban-rural connection and rapid urbanization (Pendras & Williams, 2021). Within the context of Kenya, Kisumu City stands as a major secondary city where women constitute approximately 60% of the total informal traders, primarily engaged in food vending (Kisumu, 2020; Opiyo et al., 2018). These women-led businesses play a key role in bolstering urban household food needs and livelihoods (Kiplagat et al., 2025; Odhiambo et al., 2025). Yet, pandemic-related policy frameworks failed to account for the distinct vulnerabilities of women in these informal food sectors. This neglect led to an escalation in gender-based and intimate partner violence, while simultaneously undermining the capacity of women informal enterprises to contribute to the social and economic fabric of the city (Afifu et al., 2021; John et al., 2021; Roever & Skinner, 2016; Smith, 2023). Simultaneously, increased unpaid care work and household responsibilities during the pandemic impeded women’s full engagement in paid work and in their businesses (Wenham et al., 2020).

However, limited studies have explored gender-specific gaps in COVID-19 control policies in secondary cities in the Global South. In particular, the impacts of the control measures on women-led informal food businesses in secondary cities remains an underexplored research area (Malou, 2024). Most existing studies have focused on the broader informal sector in major metropolitan cities of the Global South, such as Nairobi, Kampala, Karachi, West Bengal, and Johannesburg (Afifu et al., 2021; Anwar et al., 2023; Mondal & Chakraborty, 2022; Pinchoff et al., 2021; Rwafa-Ponela et al., 2022; Suubi et al., 2022). Policy analyses on COVID-19 in Durban, Nairobi and Kampala indicated that women operating within the urban informal economy were systematically excluded from COVID-19 policy processes (Afifu et al., 2021; Skinner et al., 2021; Suubi et al., 2022). Furthermore, intersectional analyses of the COVID-19 control measures in Karachi revealed that lockdown mandates exacerbated gendered inequalities, manifesting in heightened domestic violence and intensified caregiving responsibilities, even as women maintained their critical role in household provisioning (Anwar et al., 2023). Within the context of West Benghal, Mondal and Chackraborty (2022) posited that women informal workers had limited influence on policy decisions during the pandemic. Furthermore, as underscored by Martínez and Young (2022), there exists a critical dearth of research centred on secondary cities around the impacts of the pandemic of women in the informal food sector.

Secondary cities have a distinct role in fostering social, economic, and cultural connections between the rural and urban communities. These cities are often characterized by informality and are marginalized, especially during emergencies (Kiplagat et al., 2025). In the case of Kisumu City, COVID-19 policies were formulated at the national government level in Nairobi, a primary city in Kenya. Such centralized governance effectively excluded the lived realities of vulnerable populations in secondary cities, particularly, women.

Kisumu City is the economic hub for western Kenya and the city’s economy is largely driven by informal trade (Opiyo et al., 2018). Over 60% of the informal businesses in the city are female owned, the majority of whom reside in informal settlements and belong to the Luo or Luhya speaking communities (Kiplagat et al., 2025). In these communities, gender roles are strictly delineated, with domestic responsibilities and caregiving traditionally assigned to women and girls, whose social standing is often diminished by prevailing patriarchal systems. The implementation of pandemic containment measures disproportionately affected these women-led businesses, as the closure of educational institutions and the increased need for medical care for family members imposed substantial additional caregiving burdens (Kiplagat et al., 2025; Odhiambo et al., 2025). Additionally, systemic power imbalances and the predominance of men in leadership roles resulted in the marginalization of female perspectives, particularly those from the informal food sector, in the formulation of pandemic response strategies.

It is against this backdrop that the current study aimed to examine how COVID-19 containment policies impacted women-led informal food businesses in Kisumu City, Kenya. Specifically, it addresses how centralized governance and prevailing gender norms affected operational resilience and excluded female perspectives and lived experiences from pandemic policy formulation. By analyzing the specific gender gaps confronting female informal food vendors, this research expands the understanding of how the perspectives of women entrepreneurs were accounted for in the design and implementation of pandemic control policies within secondary cities of the Global South. Elucidating the gendered dimensions of COVID-19 policy frameworks on women in the informal food sector is imperative for advocating for equitable policy frameworks that safeguard the food and nutritional security and economic stability of urban households, particularly, in times of emergencies.

3. Strengthen the operational policy recommendations

• The revised Discussion and Conclusion now contain stronger policy implications. The policy implications have been substantially strengthened and are considerably more actionable than in the previous version.

• Nevertheless, a few recommendations remain relatively broad and could be further enhanced by briefly illustrating how the proposed governance measures could be operationalized in practice:

o “… governments should adopt gender-responsive emergency governance measures that intentionally solicit and integrate the perspectives and lived realities of informal vendors." (lines 817-819). This recommendation is appropriate, but it remains relatively general. The manuscript could briefly indicate how governments could operationalize this recommendation (e.g., formal representation of women vendors on emergency advisory committees, mandatory stakeholder consultations before implementing market restrictions, or participatory policy forums).

o "…. governments should institutionalize mechanisms for the meaningful participation of informal vendors in policy design through recognized market associations and community-based collectives." (lines 819-821). This is a stronger recommendation than in the previous version and directly addresses one of the reviewer's concerns. However, it could be strengthened further by briefly indicating what these mechanisms might involve, for example permanent consultative committees, representation within municipal emergency response structures, or structured feedback mechanisms during public health emergencies.

o "To ensure efficacy, policy interventions must be designed in collaboration with marginalized groups to accommodate social diversity and respond to localized needs." (Lines 822-823). Although this recommendation is well justified, it remains somewhat conceptual. Providing one or two examples of concrete policy instruments (e.g., targeted financial assistance, flexible licensing arrangements, emergency income protection, or tailored communication strategies) would enhance its practical value for policymakers.

• Where appropriate, consider providing more concrete examples of how governments or local authorities could implement gender-responsive and intersectionality-informed policies (e.g., stakeholder consultation mechanisms, representation of women vendors in emergency planning, targeted support programmes, or inclusive communication strategies).

Thank you for these specific suggestions on the recommendations. These have been incorporated in the conclusion section which now reads as follows: See highlighted sections.

Conclusions

In conclusion, this study offers substantial theoretical, methodological, and practical contributions to the literature on urban informal economies, particularly regarding the management of public health emergencies. From a theoretical perspective, by utilizing the IBPA framework, this research advances intersectionality-informed policy analysis in the informal economies. It systematically reveals how blanket COVID-19 mitigation policies failed to account for the heterogeneous, intersecting identities of women food vendors, thereby reinforcing pre-existing socio-economic and cultural disparities. By documenting these gendered experiences of exclusion in both policy formulation and enforcement during emergencies, the study provides a critical empirical basis for shifting governance toward gender-responsive, inclusive frameworks that prioritize the substantive engagement of marginalized populations.

While the COVID-19 pandemic may necessitate immediate public health interventions, findings from the current study emphasize the imperative for inclusive policy-making processes that incorporate female informal food vendors into the policy formulation and implementation processes. Governments must adopt gender-responsive emergency governance measures that intentionally solicit the formal representation of women vendors on emergency advisory committees coupled with mandatory consultation of partners before implementing market restrictions. Through these deliberate policy participatory initiatives that engages the female informal vendors, governments will be able to accommodate social diversity and respond to localized needs while safeguarding their unique contributions and role in maintaining urban food security.

Additionally, the study makes a methodological contribution by demonstrating the relevance of integrating IBPA framework, review of policy documents, and qualitative evidence. In so doing, the study reveals how policies intersect with lived experiences and underscores the significance of qualitative evidence in capturing the human elements of policy impact, specifically, the socioeconomic and gender disparities in secondary cities. Substantively, this research indicates that pandemic containment measures were developed and enforced utilizing a top-down methodology, which effectively marginalized female informal food vendors and failed to account for their unique needs and diverse intersecting identities. Additionally, pre-existing power dynamics and gender-based vulnerabilities were reinforced and exacerbated by the regulatory measures, including curfews, stay-at-home mandates, and physical distancing protocols.

To address this gap, policy interventions should recognize the unique roles of women informal food vendors by incorporating targeted economic support measures (e.g., targeted financial assistance, flexible licensing arrangements, and emergency income protection), flexible market regulations, and inclusive and tailored communication strategies during public health emergencies. Such integration would enhance the gender responsiveness of policy interventions and mitigate structural inequalities. Future scholarly inquiries should expand upon this analysis by conducting comparative studies across various secondary cities to evaluate how intersectional inequalities shape the experiences of informal food vendors under disparate governance and policy frameworks.

The Conclusion is much stronger than in the previous version.

• However, it could be made even more concise by focusing primarily on the principal findings, the study's contribution, and future research directions, while limiting discussion-style interpretation.

Although the Discussion is now more analytical, some repetition of empirical findings remains, particularly regarding exclusion from policy-making, enforcement-related harassment, and the blanket nature of COVID-19 policies.

• The authors should consider reducing descriptive repetition and using the Discussion primarily to interpret these findings through the IBPA framework:

o The Results already present the issue of extortion/harassment: “The overlapping policy interventions created an avenue for extortion and harassment of the women vendors:” (lines 649-650).

o The Discussion later repeats the same empirical finding: “Women vendors reported instances of inequitable treatment, including assault, harassment, and extortion by law enforcement officers.” (Lines 719-720). Suggestion: the Discussion could reduce the descriptive repetition and focus directly on interpretation: “These enforcement practices illustrate how gendered power relations shaped the implementation of COVID-19 measures in informal urban economies.”

The manuscript would benefit from one final careful language edit (for example, "venders" instead of "vendors" in the Abstract).

We have revised the discussion section which now reads as follows:

Discussion

This study aimed to examine the sociocultural and gender disparities inherent in the formulation and implementation of COVID-19 mitigation policies and the subsequent experiences of women within the informal food vending sector. The findings reveal that the formulation and execution of these pandemic mitigation measures were characterized by a top-down approach that limited the engagement of women food vendors. Furthermore, the policies were fragmented by varying levels of power and power dynamics, reinforced prevailing inequities, and neglected the diverse intersecting identities of informal food vendors. Women vendors were systematically excluded from policy-making processes due to existing governance structures wherein health policies are formulated at the national level while service delivery is delegated to county governments, with most of these roles being male dominated. At the city and community levels, women vendors possess limited power and influence; they are frequently excluded from key decision-making processes due to systemic barriers that reinforce power hierarchies and shape policy outcomes. Our research identified several critical barriers contributing to the restricted engagement of women in the COVID-19 policy-making process within Kisumu. The factors included time constraints necessitated by the urgency of the crisis, sociocultural and gender inequities, a lack of accessible information regarding participation opportunities, and a reliance on technocratic expertise within the fields of health, governance, and policy. Such findings correspond with the observations of Martínez and Young (2022), who argued that the prioritization of technical experts in policy development by governments limited the participation of street vendors in decision-making processes. Furthermore, the pervasive societal culture of patriarchy served as a significant impediment to women involvement in political and leadership roles. Despite constitutional provisions for affirmative action and the establishment of County Women Representative positions in Kenya, policy discussions and community leadership remain predominantly male-dominated. These structural barriers reinforce gender hierarchies and the exclusion of women from decision-making at multiple levels of governance and society. Consequently, this lack of representation diminished the responsiveness of policies to the specific circumstances of women in the informal food vending sector, thereby exacerbating existing socio-economic disparities. (Coalition on Violence Against Women & Making It Work, 2023).

Narratives from the vendors emphasized this systematic exclusion, with participants frequently employing the expression "forced on us" to describe their marginalization and the lack of prior consultation regarding policy initiatives. These exclusionary practices appear to deviate from Article 174 (c) f the Constitution of Kenya, which mandates the enhancement of public participation in decision-making processes affecting the citizens (Laws of Kenya, 2013). Although scholarly evidence indicates that the political empowerment of women in pandemic-related policy-making lead to improved gender-sensitive health and economic outcomes (Abras et al., 2021; Huang & Lin, 2022), the approach in Kisumu City and many other cities in the Global South such as Rangpur in Bangladesh and Rusizi and Rubavu in Rwanda, remained highly centralized within the national Ministry of Health. The development and implementation of mitigation measures were restricted to technical experts and government officials, failing to incorporate community engagement strategies, gender-based considerations, or formal feedback mechanisms for the public, particularly for women operating in the informal food economy.

Furthermore, our study findings indicate that these policies reinforced existing inequities by failing to account for the diverse intersecting identities of marginalized groups, such as women food vendors in secondary cities. The policies did not contextualize the lived realities of these women with reference to their intersecting identities or prevailing sociocultural practices. These enforcement practices illustrate how gendered power relations shaped the implementation of COVID-19 measures in informal urban economies. Similarly, Solymári et al., (2022) observed that the brutality associated with COVID-19 policy enforcement in Kenyan informal settlements infringed upon fundamental human rights. This occurred against the backdrop of a male-dominated county enforcement team in Kisumu, which contributed to biased regulation enforcement (Loo et al., 2021). Such male dominance in enforcement predisposes women to violence and physical abuse at their places of business, posing severe health and safety risks and curtailing their agency to support their families. These experiences of harassment and the application of excessive force demonstrate how established power structures directly impacted the gendered experiences of women in the informal food sector, with some vendors reporting being compelled to pay bribes of up to five thousand shillings to avoid arrest.

Likewise, research in Nairobi by Wandera et al. (2023) documented that women in the informal workforce were subjected to physical abuse by police and municipal officers during the enforcement of curfews. This indicates a consistent pattern of COVID-19 policy impacts on women vendors across both primary and secondary urban centers. These findings are corroborated by Kiaka and colleagues (2021) who emphasized that the pandemic exacerbated instances of bribery and harassment in the Global South, particularly affecting informal vendors whose survival necessitated significant resilience. Exposure to such harassment and assault restricted women’s agency to facilitate urban household food provision and sustain their families during the pandemic. Consequently, we argued that during periods of emergency, governments must implement gender-responsive governance systems to facilitate the engagement of women in the informal sector and ensure that policies address the specific requirements and experiences of this marginalized yet essential segment of the urban population.

Our study further provides evidence regarding the absence of an intersectional perspectives in the formulation of COVID-19 policies. Informal traders in Kisumu constitute a heterogeneous group characterized by diverse genders, ages, socio-economic statuses, cultural backgrounds, marital status, abilities, and religious affiliations, among other intersecting social identities. However, the pandemic mitigation strategies utilized a blanket policy approach that failed to account for the varied settings and specific identities of these vendors. This was also noted by Dutta et al., (2021) in India, where COVID-19 lockdown policies adopted a one-size-fits-all approach and overlooked existing gender differences.

These policy interventions did not consider the contextual factors such as the prevailing socio-economic situation in low- and middle-income countries (LMICs), and the pre-existing inequalities faced by women informal workers, including patriarchal structures and gender-based discrimination. Women vendors were already facing compounded challenges, including the precarity of their businesses and work, gender-based violence, discrimination, economic marginalization, and sexual harassment before the occurrence COVID-19 pandemic. The lack of gendered considerations in the policies worsened the prevailing inequalities creating an additional burden to the women vendors and influencing their resilience post-pandemic. While we acknowledge that the pandemic was a relatively new and urgent event that required immediate action through enforcement of public health orders inform of the control measures, we argue that governments must balance such urgency with inclusive and equitable engagement of women in public policy decision-making during emergencies to mitigate unintended negative impacts.

Women faced societal expectations to take up extra caregiving roles at the expense of paid work. Notably, findings from this study raise key questions regarding the value of unpaid household and caregiving duties undertaken by women. According to the United Nation’s Development Program-UN Women COVID-19 Global Gender Response Tracker, fewer than a third of the COVID-19 policy measures implemented in Kenya applied a gender perspective, with none accounting for unpaid care work (UN Women, 2021, 2022). Our findings extend this work by uncovering the experiences of women to the gender gaps in policies in localized settings of secondary cities.

Additionally, the COVID-19 policy measures introduced a significant number of new regulations with which traders were required to comply, potentially inducing "compliance fatigue" among vendors, considering the strict enforcement and use of force by the enforcement officers. This aligns with the findings of Martínez and Young (2022), which demonstrate that the exclusion of vulnerable groups in policy formulation and implementation undermines policy effectiveness and exacerbates marginalization. Such evidence may inform future policy decisions concerning the meaningful engagement of all partners to achieve inclusive and more equitable policy outcomes, particularly during emergencies. As articulated by Cattapan et al., (2020), collaborative policy development should be guided by the principle of "nothing for us without us," which promotes the substantive participation of vulnerable groups in policy design and execution.

This study is not without limitations and it is important to recognize its limits. First, the utilization of a cross-sectional research design restricted data collection to a specific timeframe, specifically between July 10, 2024, and October 15, 2024. Because this period occurred a few years after the COVID-19 pandemic, participant recollections may have lacked certain details that could further enrich the findings. Second, the geographic focus was confined to a single county out of Kenya's 47 counties, which may constrain the generalizability of the outcomes nationwide; nevertheless, these findings may possess transferability to urban centers exhibiting comparable characteristics with Kisumu City. Third, the scope was strictly limited to the informal food economy, thereby restricting generalizability to other segments of the informal sector. Furthermore, the reliance on self-reported data introduces potential social desirability bias, particularly regarding personal reflections on pandemic control measures, their impacts on women-led informal food enterprises, and identified socio-gender policy disparities. Notwithstanding these limitations, prior scholarship has successfully utilized analogous methodological approaches to document lived experiences under pandemic restrictions. Consequently, the findings of this study remain comparable to preceding research and offer significant contributions to the literature and practice regarding the gendered dynamics of the informal food sector in secondary cities within the Global South.

Attachment Submitted filename: Revision 05.07.2026_Response to Editors comments.docx

# Editor report: Decision Letter 3

**DOI:** 10.1371/journal.pone.0359620.r007

**Authors:** Wisarut Suwanprasert

11 Aug 2026

Dear Dr. Onyango,

Please submit your revised manuscript by Sep 25 2026 11:59PM. If you will need more time than this to complete your revisions, please reply to this message or contact the journal office at plosone@plos.org. When you're ready to submit your revision, log on to https://www.editorialmanager.com/pone/ and select the 'Submissions Needing Revision' folder to locate your manuscript file.

- A letter that responds to each point raised by the academic editor and reviewer(s). You should upload this letter as a separate file labeled 'Response to Reviewers'.
- A marked-up copy of your manuscript that highlights changes made to the original version. You should upload this as a separate file labeled 'Revised Manuscript with Track Changes'.
- An unmarked version of your revised paper without tracked changes. You should upload this as a separate file labeled 'Manuscript'.

If you would like to make changes to your financial disclosure, please include your updated statement in your cover letter. Guidelines for resubmitting your figure files are available below the reviewer comments at the end of this letter.

If applicable, we recommend that you deposit your laboratory protocols in protocols.io to enhance the reproducibility of your results. Protocols.io assigns your protocol its own identifier (DOI) so that it can be cited independently in the future. For instructions see: https://journals.plos.org/plosone/s/submission-guidelines#loc-laboratory-protocols. Additionally, PLOS ONE offers an option for publishing peer-reviewed Lab Protocol articles, which describe protocols hosted on protocols.io. Read more information on sharing protocols at https://plos.org/protocols?utm_medium=editorial-email&utm_source=authorletters&utm_campaign=protocols.

As the corresponding author, your ORCID iD is verified in the submission system and will appear in the published article. PLOS supports the use of ORCID, and we encourage all coauthors to register for an ORCID iD and use it as well. Please encourage your coauthors to verify their ORCID iD within the submission system before final acceptance, as unverified ORCID iDs will not appear in the published article. *Only*  the individual author can complete the verification step; PLOS staff *cannot*  verify ORCID iDs on behalf of authors.

We look forward to receiving your revised manuscript.

Kind regards,

Wisarut Suwanprasert, Ph.D.

Academic Editor

PLOS One

Journal Requirements:

1. If the reviewer comments include a recommendation to cite specific previously published works, please review and evaluate these publications to determine whether they are relevant and should be cited. There is no requirement to cite these works unless the editor has indicated otherwise.

2. Please review your reference list to ensure that it is complete and correct. If you have cited papers that have been retracted, please include the rationale for doing so in the manuscript text, or remove these references and replace them with relevant current references. Any changes to the reference list should be mentioned in the rebuttal letter that accompanies your revised manuscript. If you need to cite a retracted article, indicate the article’s retracted status in the References list and also include a citation and full reference for the retraction notice.

[Note: HTML markup is below. Please do not edit.]

[NOTE: If reviewer comments were submitted as an attachment file, they will be attached to this email and accessible via the submission site. Please log into your account, locate the manuscript record, and check for the action link "View Attachments". If this link does not appear, there are no attachment files.]

To ensure your figures meet our technical requirements, please review our figure guidelines: https://journals.plos.org/plosone/s/figures

You may also use PLOS’s free figure tool, NAAS, to help you prepare publication quality figures: https://journals.plos.org/plosone/s/figures#loc-tools-for-figure-preparation.

NAAS will assess whether your figures meet our technical requirements by comparing each figure against our figure specifications.

# Author comment: Author response to Decision Letter 4

**DOI:** 10.1371/journal.pone.0359620.r008

12 Sep 2026

Response to Editor’s comments

NB: Highlighted in yellow are the edited/revised sections in the paper.

NB: Highlighted in blue are the editor/reviewers’ comments and suggested edits

NB: Highlighted in gray are our responses to the suggestions/comments

The manuscript does not appear to have received the careful final language edit requested by Reviewer 3. Numerous grammatical errors, typographical mistakes, sentence fragments, and unclear expressions remain throughout the manuscript. Examples include:

Thanks for these valuable observations. We have done a comprehensive review of the manuscript and revised sentence and paragraphs in the paper to ensure clarity and succinct communication of the key messages. W hope the revisions addressed the grammatical, typological, fragmented sentences, and unclear expressions that were previously in the paper.

For example, the below highlighted grammatical/typological edited section now read as follows:

“West Benghal” rather than “West Bengal” (line 102).

…..

However, research on gender-specific gaps in COVID-19 control policies remains limited, particularly regarding the impact on women-led informal food businesses in secondary cities of the Global South (Malou, 2024). Most existing literature focuses on the broader informal sector in major metropolitan hubs such as Nairobi, Kampala, Karachi, West Bengal, and Johannesburg (Afifu et al., 2021; Anwar et al., 2023; Mondal & Chakraborty, 2022; Pinchoff et al., 2021; Rwafa-Ponela et al., 2022; Suubi et al., 2022). Previous analyses in Durban, Nairobi, and Kampala indicate that women in the urban informal economy were systematically excluded from COVID-19 policy processes (Afifu et al., 2021; Skinner et al., 2021; Suubi et al., 2022). Additionally, studies in Karachi demonstrate that lockdown mandates intensified gendered inequalities—increasing domestic violence and caregiving burdens—even as women remained essential to household food security (Anwar et al., 2023). Similarly, research in West Bengal suggests that women informal workers had little influence on pandemic policy decisions (Mondal & Chakraborty, 2022). Overall, as emphasized by Martínez and Young (2022), there is a critical need for more research focused specifically on how the pandemic affected women in the informal food sector within secondary cities.

“the impacts of the pandemic of women” instead of “on women” (line 105).

Thanks for highlighting this. It has been revised!

“K With a population…” (line 198).

Thanks for highlighting this. It has been revised!

“Selection participants” as a heading (line 229).

Thanks for highlighting this. It has been revised!

“with at least 1 to 2 participants…” beginning as a sentence fragment (line 273).

Thanks for highlighting this. It has been revised!

“representatives the County Government” (line 321).

Thanks for highlighting this. It has been revised!

“Article 174 (c) f the Constitution” rather than “of the Constitution” (line 715).

Thanks for highlighting this. It has been revised!

Finally, the cover letter concludes with the statement, “We look forward to sharing our work in the Health & Place Journal.” Please ensure that all submission materials and correspondence correctly identify PLOS ONE as the journal to which the manuscript is being submitted.

Very much appreciate this highlight! A revised letter has been submitted.

NB: Overall, we have done a comprehensive review of the manuscript to enhance clarity and succinct communication.

Attachment Submitted filename: Revision 03.09.2026_Response to Editors comments.docx

# Editor report: Decision Letter 4

**DOI:** 10.1371/journal.pone.0359620.r009

**Authors:** Wisarut Suwanprasert

16 Sep 2026

An Intersectionality-Based Policy Analysis of COVID-19 Measures and Women Informal Food Vendors in Kisumu, Kenya

PONE-D-25-46140R4

Dear Dr. Onyango,

We’re pleased to inform you that your manuscript has been judged scientifically suitable for publication and will be formally accepted for publication once it meets all outstanding technical requirements.

Within one week, you’ll receive an e-mail detailing the required amendments. When these have been addressed, you’ll receive a formal acceptance letter and your manuscript will be scheduled for publication.

An invoice will be generated when your article is formally accepted. Please note, if your institution has a publishing partnership with PLOS and your article meets the relevant criteria, all or part of your publication costs will be covered. Please make sure your user information is up-to-date by logging into Editorial Manager at Editorial Manager® and clicking the ‘Update My Information' link at the top of the page. For questions related to billing, please contact billing support.

If your institution or institutions have a press office, please notify them about your upcoming paper to help maximize its impact. If they’ll be preparing press materials, please inform our press team as soon as possible -- no later than 48 hours after receiving the formal acceptance. Your manuscript will remain under strict press embargo until 2 pm Eastern Time on the date of publication. For more information, please contact onepress@plos.org.

Kind regards,

Wisarut Suwanprasert, Ph.D.

Academic Editor

PLOS One

Additional Editor Comments (optional):

Reviewers' comments:

# Editor report: Acceptance letter

**DOI:** 10.1371/journal.pone.0359620.r010

**Authors:** Wisarut Suwanprasert

PONE-D-25-46140R4

PLOS One

Dear Dr. Onyango,

I'm pleased to inform you that your manuscript has been deemed suitable for publication in PLOS One. Congratulations! Your manuscript is now being handed over to our production team.

At this stage, our production department will prepare your paper for publication. This includes ensuring the following:

* All references, tables, and figures are properly cited

* All relevant supporting information is included in the manuscript submission,

* There are no issues that prevent the paper from being properly typeset

You will receive further instructions from the production team, including instructions on how to review your proof when it is ready. Please keep in mind that we are working through a large volume of accepted articles, so please give us a few days to review your paper and let you know the next and final steps.

Lastly, if your institution or institutions have a press office, please let them know about your upcoming paper now to help maximize its impact. If they'll be preparing press materials, please inform our press team within the next 48 hours. Your manuscript will remain under strict press embargo until 2 pm Eastern Time on the date of publication. For more information, please contact onepress@plos.org.

You will receive an invoice from PLOS for your publication fee after your manuscript has reached the completed accept phase. If you receive an email requesting payment before acceptance or for any other service, this may be a phishing scheme. Learn how to identify phishing emails and protect your accounts at https://explore.plos.org/phishing.

If we can help with anything else, please email us at customercare@plos.org.

Thank you for submitting your work to PLOS One and supporting open access.

Kind regards,

PLOS One Editorial Office Staff

on behalf of

Dr. Wisarut Suwanprasert

Academic Editor

PLOS One
